# Mechanistic insight into anaphase bridge signaling to the abscission checkpoint

**DOI:** 10.1038/s44318-025-00453-w

**Published:** 2025-05-12

**Authors:** Manika I Singh, Girish Rajendraprasad, Vasileios Katopodis, Rui Cui, Marin Barisic, Rahul Bhowmick, Ian D Hickson

**Affiliations:** 1 Center for Chromosome Stability, Department of Cellular and Molecular Medicine, University of Copenhagen, Blegdamsvej 3B, 2200 Copenhagen N, Denmark; 2 Danish Cancer Society Research Center, Strandboulevarden 49, 2100 Copenhagen N, Denmark; 3 Department of Cellular and Molecular Medicine, University of Copenhagen, Blegdamsvej 3B, 2200 Copenhagen N, Denmark; 4 Department of Biochemistry, Vanderbilt University, Nashville, TN 37232 USA; 5 Present Address: Centre for Genomic Medicine, Rigshospitalet, Blegdamsvej 9, 2100 Copenhagen, Denmark

**Keywords:** ATR-CHK1 Signaling, Chromosome Missegregation, Aneuploidy, Cytokinesis Failure, Anchorage Independent Growth, Cell Cycle, DNA Replication, Recombination & Repair

## Abstract

During cytokinesis in human cells, a failure to resolve persistent DNA bridges that span the cell-division plane maintains the Aurora B-dependent abscission checkpoint in an active state. However, the molecular mechanism by which unresolved sister-chromatid bridging signals to this checkpoint is poorly defined. Here, we define an essential role for the Bloom’s syndrome helicase, BLM, in signaling to the abscission-checkpoint machinery in response to replication stress through the conversion of dsDNA bridges into RPA-coated ssDNA. RPA then promotes ATR-CHK1 signaling to Aurora B, utilizing a kinase cascade shared with the S-phase checkpoint. BLM-deficient cells ultimately abandon cytokinesis in response to replication stress, which promotes binucleation and hence aneuploidy. Considering that aneuploidy is a hallmark of cancer, we propose that this role for BLM in cytokinesis is a plausible reason for cancer predisposition in Bloom’s syndrome individuals. Consistent with this, BLM deficiency promotes anchorage-independent growth of non-cancer cells.

## Synopsis

How unresolved sister-chromatid bridges maintain abscission checkpoint activity during cytokinesis remains unclear. This work shows that in response to DNA replication stress, the Bloom’s syndrome helicase (BLM) signals to the checkpoint machinery and prevents binucleation and aneuploidy, consistent with BLM acting as tumor suppressor.

- BLM and RPA regulate abscission-checkpoint activation.
- The RPA-ATR-Chk1 S-phase checkpoint axis ‘moonlights’ in mitosis.
- Perturbation of BLM-RPA function in mitosis induces cytokinesis failure, a driver of aneuploidy.
- Inactivation of BLM promotes anchorage-independent growth in non-cancer cells.

## Abstract (web summary)

The Bloom’s syndrome helicase BLM is essential for blocking cytokinetic abscission and preventing binucleation in response to DNA replication stress, explaining its role as a tumor suppressor.

## Graphical abstract

**Figure:**
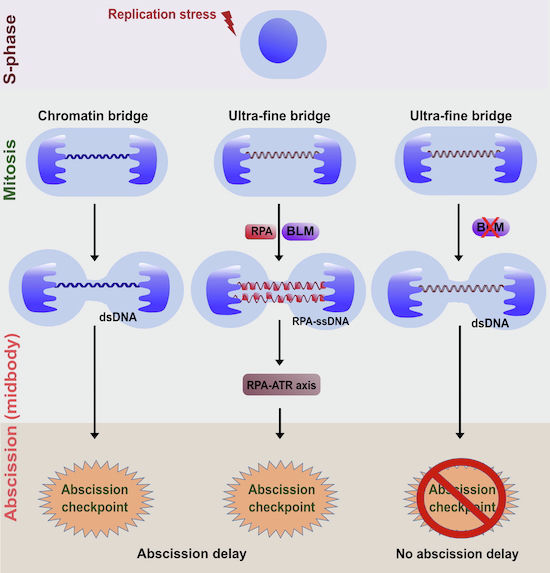

**Figure:**
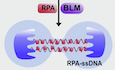

## Introduction

The human genome is packaged into 23 pairs of chromosomes. A chromosome number differing from the canonical 46 per cell is referred to as aneuploidy and is a characteristic feature of most solid tumors in humans (Gordon et al, 2012; Li and Zhu, 2022; Sansregret and Swanton, 2017). Aneuploidy in tumor cells is generally associated with a poor disease prognosis and the development of resistance to therapeutic drugs and radiation (Mahadevan and Rogers, 2020; Replogle et al, 2020; Taylor et al, 2018; Vasudevan et al, 2021; Weaver and Cleveland, 2006). There are several potential ways by which cells can acquire aneuploidy, but the most common are through perturbation of DNA replication (replication stress; RS) or *via* chromosome segregation errors during mitosis (Bach et al, 2019; Böhly et al, 2019; Gordon et al, 2012; Zhu et al, 2018). These drivers of aneuploidy are often functionally interconnected in tumors, since the activation of oncogenes such as *MYC* generates RS that can lead to chromosome mis-segregation. Similarly, mitotic errors can activate RS in subsequent cell cycles (Halazonetis et al, 2008; Santaguida and Amon, 2015; Zhu et al, 2018). Indeed, it is recognized that activated oncogenes promote and cooperate with aneuploidy for inducing Chromosome Instability (CIN) and neoplastic transformation (Orr and Compton, 2013; Woo and Poon, 2004).

Aneuploidy often arises from a tetraploid/binucleated precursor cell generated following cytokinesis failure in the previous cell division cycle (Hayashi and Karlseder, 2013). Perhaps the most common (patho)physiological driver of cytokinesis failure is the presence of unresolved chromosomal DNA lying in the cell division plane (Mendoza et al, 2009; Steigemann et al, 2009). Such unresolved DNA prevents the execution of the final stage of cytokinesis known as abscission through a prolonged activation of the abscission checkpoint, which depends on an Aurora B kinase-dependent signaling cascade centered on the midbody (Petsalaki and Zachos, 2016). Aurora B kinase is activated and triggers the abscission checkpoint even in an unperturbed mitosis. When no DNA bridges are present, the checkpoint is satisfied by PP1-mediated dephosphorylation, which is the trigger for abscission to occur (Bhowmick et al, 2019; Petsalaki and Zachos, 2016; Steigemann et al, 2009). In the presence of DNA bridges, Aurora B remains persistently active until the bridge is resolved. Hence, the abscission checkpoint allows sufficient time for the cells to promote the resolution of the DNA in the abscission plane and hence to continue with faithful cytokinesis (Petsalaki and Zachos, 2021a). Ultimately, if bridge resolution is impossible, the persistent activation of the abscission checkpoint leads to one of two undesirable outcomes: (i) Cleavage furrow regression and the abortion of cytokinesis, with the subsequent generation of binucleated progeny (Steigemann et al, 2009). (ii) Physical rupture of the bridge, which can generate micronuclei and chromosomal instability (Carlton et al, 2012; Petsalaki and Zachos, 2021a). The activation of the checkpoint depends upon phosphorylation of the Aurora B kinase on Thr-232, which subsequently phosphorylates several other proteins at the midbody, including CHMP4C, an ESCRT-III subunit that interacts with the chromosome passenger complex (Carlton et al, 2012; Petsalaki et al, 2018; Yasui et al, 2004). Phosphorylated CHMP4C interacts with and restricts VPS4 from localizing to the midbody core, preventing abscission until the RIF1-PP1 complex dephosphorylates Aurora B (Bhowmick et al, 2019; Thoresen et al, 2014).

It is well established that unresolved anaphase DNA bridges are potent activators of the abscission checkpoint (Bhowmick et al, 2019; Steigemann et al, 2009). These bridges can be bulky and chromatinized, and likely are formed from whole or fused chromosomes, but the frequency of these bridges is low in normally growing human cell lines. More commonly, the bridges comprise thin, chromatin-free DNA threads termed ultra-fine DNA bridges (UFBs), and these structures arise in virtually all mitoses in cultured cancer cell lines (Liu et al, 2014). The composition of UFBs is not entirely clear, although it is likely that they represent regions of catenated or entangled DNA linking the separating sister chromatids (Liu et al, 2014). UFBs do not stain clearly with DNA dyes such as DAPI and are generally visualized using immunofluorescence for bridge-associated proteins (Bizard et al, 2018). Commonly used markers of UFBs are the SNF2 family translocase PICH, RIF1, and members of the BTRR complex comprising BLM, TOP3A, RMI1 and RMI2 (Liu et al, 2014). Cells defective in any of these factors show a delay in the resolution of UFBs, such that bridges can persist beyond the end of anaphase (Hengeveld et al, 2015). PICH appears to play several non-catalytic roles on UFBs, including recognition of UFB DNA and recruitment of other factors to UFBs, including the BTRR proteins (Bizard et al, 2019; Sarlós et al, 2018). However, it is not known how the catalytic activity of the PICH and BTRR enzymes is utilized during UFB processing. The BTRR complex, by virtue of containing a topoisomerase (TOP3A), is implicated in decatenating/disentangling intertwined DNA elements in UFBs that prevent their timely separation, but direct evidence for this role is currently lacking.

BLM is a DNA helicase that belongs to the RecQ family. Cells lacking BLM show aneuploidy and many other features of chromosomal instability, including excessive anaphase bridging and micronucleus formation (Chan et al, 2007). Germline mutations in the *BLM* gene in humans give rise to Bloom’s syndrome (BS), an autosomal recessive condition characterized by pre- and post-natal growth retardation, microcephaly, skin pigmentation abnormalities, and a strong predisposition to the development of cancer (Cunniff et al, 2017; Ellis et al, 1995). BS is of particular interest because affected individuals succumb to the full range of cancers seen in the general population, albeit at an unusually early age. This suggests that, unlike many other cancer predisposition disorders where a very limited range of cancers is observed, the functional defect in BS is likely to impact on the basic biology underlying tumorigenesis. Hence, understanding the ways in which cellular processes become deranged in BLM-defective cells might enrich our knowledge of some of the fundamental drivers of tumorigenesis.

In this study, we set out to define the consequences of BLM deficiency for cytokinesis and cell ploidy. We show that BLM is required to prolong the abscission checkpoint in response to the presence of an unresolved UFB during late mitosis and hence suppresses cytokinesis failure and prevents tetraploidization. We also confirm that this role is mediated by UFB unwinding (Chan et al, 2018) and reveal that the ssDNA generated by BLM promotes RPA-mediated signaling through the ATR-CHK1 axis to the Aurora B kinase. Consistent with a role for BLM in preventing aneuploidy, we show that BLM deficiency combined with p53 deficiency can activate a degree of anchorage-independent growth in immortalized, but untransformed, RPE1 cells, particularly in cells constitutively expressing the *MYC* oncogene. Taken together, our data not only define a novel role for BLM in cytokinesis but also establish that human cells utilize the same machinery to activate checkpoints during S-phase and mitosis. We propose a model in which cytokinesis failure in BLM-deficient cells serves as a mechanism to generate aneuploidy and hence predisposes Bloom’s syndrome sufferers to the development of cancers of many types.

## Results and discussion

### BLM influences the activation of the abscission checkpoint

**Figure 1:**
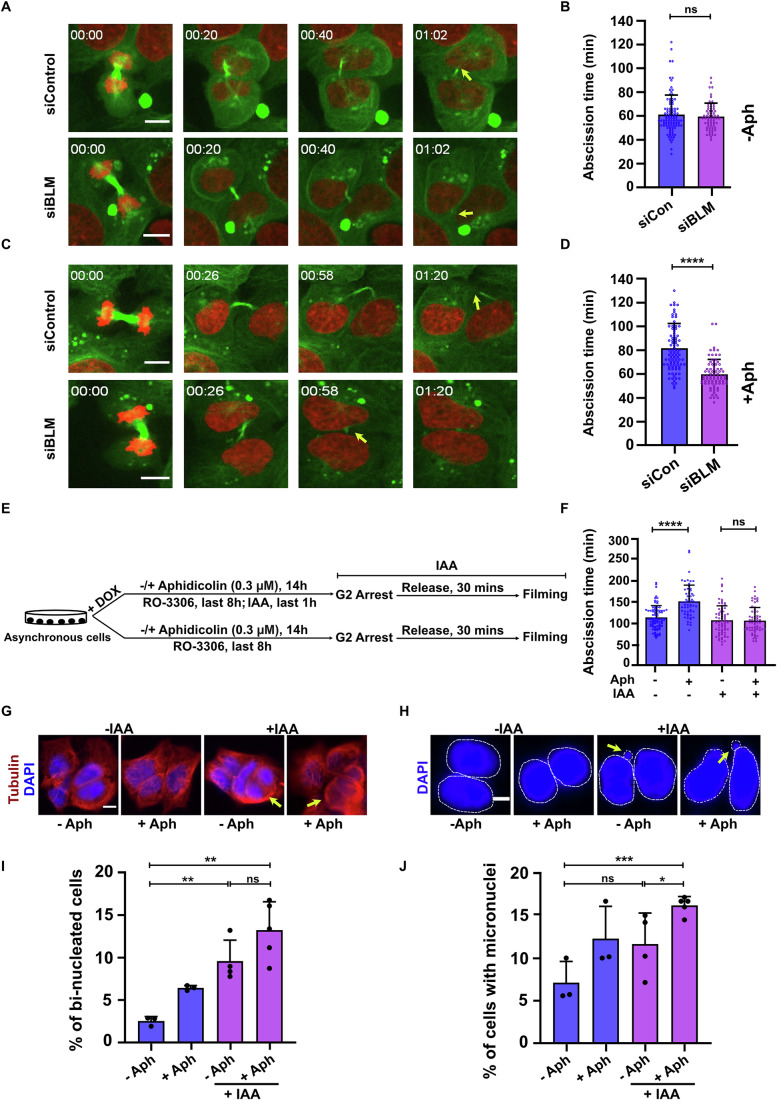
Abscission delay triggered by UFBs requires BLM. (**A**) Representative still pictures derived from live-cell imaging of U2OS cells (with no visible chromatin bridge) stably expressing fluorescently tagged histone H2B (red) and α-tubulin (green) and treated with either siControl or siBLM for 48 h. The yellow arrows indicate the site of abscission. Scale bar, 10 µm. (**B**) Quantification of abscission time derived from the live-cell imaging (*n* = >60) described in (**A**). The data are an average of three independent biological replicates with error bars representing the standard deviation. The significance was calculated using a two-tailed Mann–Whitney test. No significant difference was observed between siCon and siBLM. Exact *p* value is 0.92 (n.s.). (**C**) Representative still pictures derived from live-cell imaging of U2OS cells (with no visible chromatin bridge) stably expressing fluorescently tagged histone H2B (red) and α-tubulin (green) and treated with either siControl or siBLM for 48 h in presence of RS (0.3 µM Aph). The yellow arrows indicate the site of abscission. Scale bar, 10 µm. (**D**) Quantification of abscission time derived from the live-cell imaging (*n* = >60) described in (**C**). The data are an average of three independent biological replicates with error bars representing the standard deviation. The significance was calculated using a two-tailed Mann-Whitney test. Exact *p* value is <0.0001 (****). (**E**) Experimental workflow of assessing abscission time in BLM degron cells treated or not with IAA (to induce BLM degradation) exclusively during mitosis in the presence or absence of RS (0.3 µM Aph). (**F**) Quantification of abscission time derived from live-cell imaging (*n* = >60) of BLM degron cells as described in (**E**). The data are an average of three independent biological replicates with error bars representing the standard deviation. The significance was calculated using a two-tailed Mann–Whitney test. Exact *p* values are <0.0001 (****) and 0.95 (n.s.). (**G**, **H**) Representative images of BLM degron cells treated or not with IAA (to induce BLM degradation) exclusively during mitosis in presence (+Aph) or absence (-Aph) of RS (0.3 µM Aph) depicting formation of bi-nucleated cells (**G**) and micronuclei (**H**). Scale bars are 10 µm (**G**) and 4 µm (**H**). The yellow arrows indicate the bi-nucleated cells (**G**) and micronuclei (**H**). The nucleus and micronuclei are encircled using a dotted lines for the ease of visualization in (**H**). (**I**) Quantification of bi-nucleated cells (*n* = >300) from the experiment in (**G**). The data are an average of three or more independent biological replicates with error bars representing the standard deviation. The significance was calculated using a two-tailed t-test. Exact *p* values are 0.005 (**), 0.114 (n.s.) and 0.002 (**) between –Aph/ –Aph +IAA, -Aph+IAA/+Aph+IAA and –Aph/+Aph+IAA, respectively. (**J**) Quantification of micronucleated cells (*n* = >300) from the experiment in (**H**). The data are an average of three or more independent biological replicates with error bars representing the standard deviation. The significance was calculated using a two-tailed t-test. Exact *p* values are 0.127 (n.s.), 0.031 (*) and 0.0003 (***) between –Aph/ –Aph +IAA, -Aph+IAA/+Aph+IAA and –Aph/+Aph+IAA, respectively. Source data are available online for this figure.

In a recent study, we established that unresolved UFBs maintain the abscission checkpoint in an active state and delay cytokinesis (Bhowmick et al, 2019). This finding suggested that UFBs must in some way generate a signal that is ultimately received by the abscission checkpoint machinery. To define the molecular mechanism of this checkpoint signaling from UFBs in human cells, we tested the hypothesis that one or more UFB-associated proteins might play the role of a UFB ‘sensor’ and hence be required for maintaining the checkpoint. More specifically, we surmised that, in the absence of a protein that acts as a bridge sensor, cells would proceed with abscission even if a UFB was still present in the cell division plane and would do so with similar kinetics to a cell that lacks UFBs. To investigate this, we depleted either BLM or PICH and determined abscission timing (Fig. EV1A,B), which we define here as the time between complete cleavage furrow ingression and midbody cleavage. It is important to note that we excluded from this analysis any cell that contained a chromatin bridge. We observed that depletion of PICH increased the time required for abscission (Fig. EV1C,D), as would be expected of a protein required for UFB resolution but not for signaling the presence of bridge DNA to the checkpoint machinery. This phenotype is analogous to that seen in cells lacking RIF1 (Bhowmick et al, 2019). In contrast, upon BLM depletion, despite there being a delay in the resolution of UFBs (Fig. EV1E), cells failed to undergo any abscission delay (Fig. 1A,B and Movie EV1). These data suggest that BLM might play a role in maintaining activation of the abscission checkpoint. Although inhibition of the abscission checkpoint is known to reduce abscission time even in cells lacking DNA bridges (Mackay and Ullman, 2015; Steigemann et al, 2009), we did not observe any reduction in abscission timing in BLM depleted cells with persistent UFBs compared to control cells having no persistent UFBs. This suggests that rather than activating the abscission checkpoint directly upon mitotic entry, BLM acts as a UFB signaling factor that can maintain the checkpoint in an activated state, leading to abscission delay. During the live cell imaging we observed atypical aggregates in the mCherry-tubulin channel. Through performing immunofluorescence with an anti-tubulin antibody, we confirmed that these aggregates represent free mCherry and not mCherry-tubulin (Appendix Fig. S1A).

**Figure EV1:**

The absence of PICH induces a delay in the timing of abscission. (**A**) Representative western blot of a whole cell lysate derived from U2OS cells treated with siControl, siBLM or siPICH for 48 h. (**B**) Experimental workflow for live-cell imaging experiments depicted in Fig. 1A–D. (**C**, **D**) Representative stills (**C**) and quantification (**D**) derived from live-cell imaging of U2OS cells (with no visible chromatin bridges) stably expressing fluorescently tagged histone H2B (red) and α-tubulin (green), treated with either siControl or siPICH for 48 h (*n* = >60). The yellow arrows indicate the site of abscission. Scale bar, 10 µm. The data are an average of three independent biological replicates with error bars representing the standard deviation. A Mann–Whitney test was performed to derive significance. Exact *p* value is <0.0001 (****). The data used for siCon in (**D**) are the same as used in Fig. 1B, as these experiments were performed together. (**E**) Representative immunofluorescence images and quantification of the total number of UFBs in U2OS cells during anaphase after treatment with siControl or siBLM for 48 h (*n* = >50). Scale bar, 5 µm. Significance was derived using a t-test. Exact *p* values are 0.0062 (**) between siCon/sicon+Aph, 0.012 (*) between siCon/siBLM, 0.008 (**) between siBLM/siBLM+Aph and 0.0002 (***) between siCon/siBLM+Aph. Source data are available online for this figure.

To study this putative role of BLM as a DNA bridge sensor in more detail, particularly in relation to cancer cells, we induced persistent UFBs by treating cells with a low dose of aphidicolin (Aph; which slows the progression of replicative DNA polymerases) (Chan et al, 2009). This treatment mimics the RS that arises during tumorigenesis due to oncogene activation. Upon RS, cells progress into mitosis with under-replicated loci that ultimately appear as UFBs in anaphase (Chan et al, 2009). Although this treatment can also generate chromatin bridges in a small percentage of cells, we focused on UFBs and only scored cells that lack chromatin bridges. We observed that, in the presence of RS-induced UFBs, control cells triggered an abscission delay but cells in which BLM was depleted with siRNAs did not (Fig. 1C,D and Movie EV2). To investigate this further, we first considered whether BLM’s apparent role in abscission might be due to a role conducted in interphase, rather than in mitosis, such as in replication fork re-start (Davies et al, 2007). To address this, we developed an auxin-inducible ‘degron’ system (Nishimura et al, 2009) (Fig. EV2A) where we could deplete BLM protein by >90% in only 45 min following addition of indole acetic acid (IAA) to the culture medium (Fig. EV2B/C). Using this degron cell line, we depleted BLM at the G2/M boundary and then assessed abscission timing in the presence or absence of Aph (Figs. 1E and EV2D). We observed that, in absence of Aph, degradation of BLM at the G2/M boundary did not significantly affect abscission timing. In contrast, in the presence of Aph, BLM degron cells failed to induce an abscission delay, unlike control cells in which BLM was not degraded (Figs. 1F and EV2E). Taken together, these observations suggest that BLM plays a critical role in mitosis itself in signaling to the abscission checkpoint. Further, they suggest that this role is required only in those mitoses where UFBs persist into late mitosis, as occurs frequently following RS.

**Figure EV2:**

Mitotic degradation of BLM prevents replication stress-induced abscission delay. (**A**) Depiction of the procedure for generating a BLM degron in DLD1 cells (left) and a graphical overview of the mechanism by which BLM undergoes degradation (right). (**B**) Experimental workflow for assessing BLM degradation over time in BLM degron cells upon IAA addition. (**C**) A representative western blot of whole cell lysates derived from BLM degron cells treated with IAA for the indicated times. The data are representative of two independent biological replicates. (**D**) A representative western blot of BLM degron cells, arrested in G2-M checkpoint with RO-3306 for 8 h before degrading the BLM by adding IAA (auxin). BLM depletion was monitored for up to 6 h after adding IAA. Cells were then washed and released with fresh warm media and BLM recovery was monitored for up to 48 h. (**E**) Representative still pictures derived from live-cell imaging of BLM degron cells (with no visible chromatin bridges) stably expressing fluorescently tagged α-tubulin (green) and histone H2B (red) treated with (+IAA) or without (-IAA). BLM degradation was induced exclusively in mitosis following exposure or not of cells to RS (0.3 µM Aph), as indicated in Fig. 1E. The yellow arrows indicate the site of abscission. Scale bar, 10 µm. Source data are available online for this figure.

To provide evidence that it is the persistence of UFBs that promotes abscission delay following RS, we overexpressed a GFP-tagged version of the human Ankle1 nuclease and monitored abscission duration with and without RS (Fig. EV3A). The Ankle1 nuclease migrates to the midbody during cytokinesis and is believed to cut unresolved or partially resolved DNA junctions during late mitosis (Brachner et al, 2012; Jiang et al, 2023; Song et al, 2020). Thus, Ankle1 is a good candidate for the cleavage of DNA bridges during abscission. We observed that, in absence of RS (-Aph), there was no change in abscission timing upon GFP-Ankle1 overexpression. However, through comparing abscission timing in those cells expressing GFP-Ankle1 or not (absence of green fluorescence at the midbody) in the same population, we observed that the abscission delay induced by Aph was overcome upon expression of GFP-Ankle1 (Fig. EV3B). To substantiate these findings, we utilized DLD1 cells expressing Neon-PICH and mCherry-Tubulin and analyzed the duration of abscission in cells treated with or without Aph. In almost all mitoses (even without RS) multiple PICH-coated bridges were visible during anaphase A, but these were largely resolved prior to the point where the cells progressed into anaphase B. These bridges generally arise from centromeres and are a common feature of mitosis in human cancer cells (Liu et al, 2014). In contrast, following exposure to Aph, we observed persistent bridges in some anaphase B cells and those cells exhibited a significant delay in completing abscission (Fig. EV3C). In addition, we observed a slight increase in abscission timing in cells that lacked a visible bridge following treatment with Aph, suggesting that RS might also have a limited effect on the checkpoint, perhaps through the generation of DNA lesions (Fig. EV3C). Nevertheless, this effect was much less pronounced than the effect of a persistent UFB on the checkpoint. Taken together, these observations strongly suggest that UFBs induced by RS cause a delay in abscission and that BLM plays a critical role in the maintenance of the abscission checkpoint. Recent studies (Lussi et al, 2010) have demonstrated that nucleoporins, such as NUP153, regulate cytokinesis by modulating spindle assembly checkpoint activity. Our findings indicate that BLM does not contribute to nucleoporin-triggered cytokinesis regulation (Appendix Fig. S1B), suggesting a specific role for BLM in regulating UFB-induced cytokinetic delay.

**Figure EV3:**

Expression of Ankle1 nuclease reduces the incidence of DNA bridges and prevents RS-induced abscission delay. (**A**) Representative still pictures derived from live-cell imaging of U2OS cells (with no visible chromatin bridges) stably expressing fluorescently tagged histone H2B (green) and α-tubulin (red) and transiently expressing GFP-Ankel protein (which can be seen to localize to the midbody; in panels +Ankle1), in the presence (+Aph) or absence (-Aph) of RS (0.3 µM Aph). The yellow arrows indicate the site of abscission. Scale bar, 10 µm. (**B**) Quantification of abscission time derived from the live-cell imaging described in (**A**) (*n* => 10). The data are an average of two independent biological replicates with error bars representing the standard deviation. Mann-Whitney test was performed to derive significance. Exact *p* values are 0.89 (n.s.) and 0.014 (*). (**C**) Quantification of abscission time derived from the live-cell imaging of DLD1 cells expressing neon-PICH and mCherry-Tubulin along with SiR-DNA (*n* => 10). Mitotic cells under RS (APH), but having no visible PICH bridge, were analyzed separately from mitotic cells with visible PICH bridges and cells treated with RPAi (discussed later in the text). The data are an average of three independent biological replicates with error bars representing the standard deviation. Mann-Whitney test was performed to derive significance. Exact *p* values are 0.0016 (**) between UT/Aph (no bridge), <0.0001 (****) between UT/Aph (with bridge) and 0.289 (ns) between UT/Aph+RPAi. (**D**) Experimental workflow for assessing bi-nucleation and micronuclei formation with BLM degron cells with (+IAA) or without (-IAA) BLM degradation exclusively in mitosis in presence or absence of RS (0.3 µM Aph). Source data are available online for this figure.

It has been proposed previously that a delay in abscission allows cells sufficient time to resolve DNA bridges (Bhowmick et al, 2019; Steigemann et al, 2009). In the absence of such a delay, cells proceed with abscission even though unresolved DNA bridges persist, resulting in one or more of the following aberrant cytokinetic outcomes; bi-nucleation, micronucleus formation or aneuploidy (Chan et al, 2007; Chan et al, 2018; Hengeveld et al, 2015; Jallepalli and Lengauer, 2001; Nielsen et al, 2015). These chromosomal abnormalities are proposed to be important contributors to tumorigenesis (Levine and Holland, 2018). Therefore, we assessed the frequency of bi-nucleation and micronucleus formation following exposure to Aph in cells in which BLM was degraded only in mitosis (Fig. EV3D). We observed that BLM-depleted cells displayed increased frequencies of both bi-nucleation and micronuclei (Fig. 1G–J). These data indicate that BLM’s role in suppression of gross chromosomal abnormalities must also require a function undertaken specifically in mitosis.

### RPA acts as a central signaling molecule to activate the abscission checkpoint

**Figure 2:**
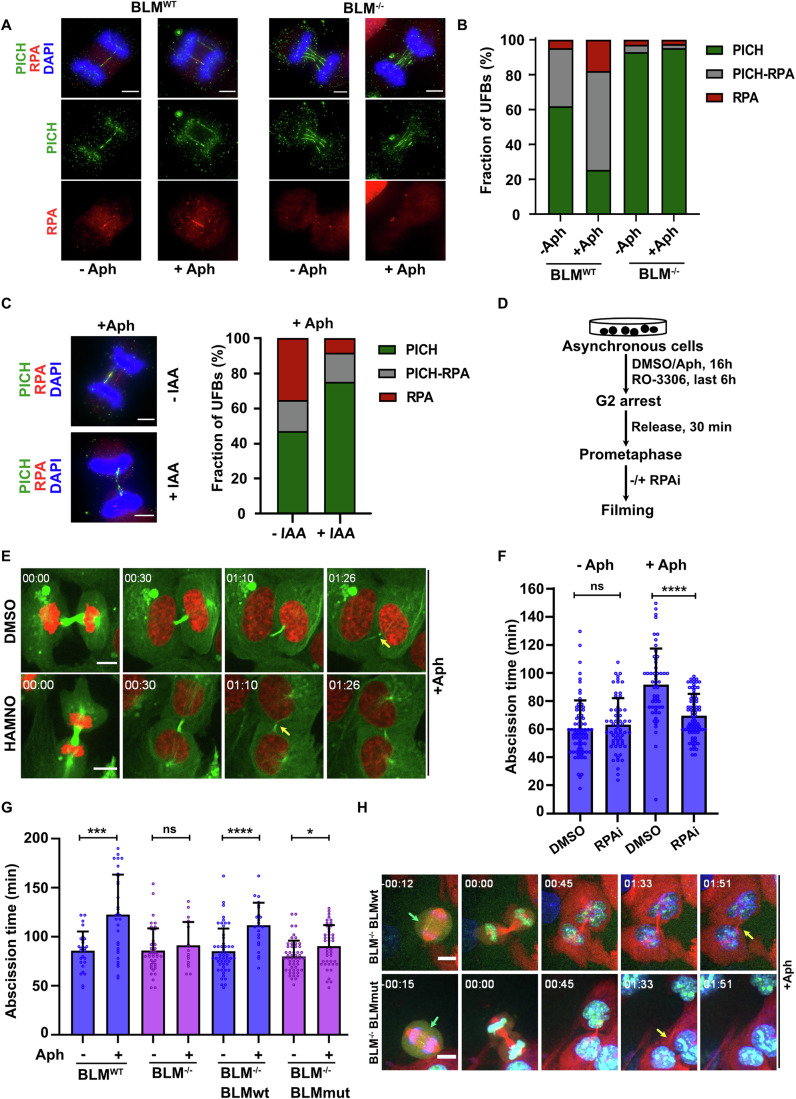
RPA activates the abscission checkpoint. (**A**, **B**) Representative images (**A**) and quantification (**B**) of PICH coated, RPA-coated and dual PICH-RPA-coated UFBs in the presence (BLM^WT^) or absence of BLM (BLM^−/−^), and with (+Aph) or without (−Aph) RS (0.3 µM Aph) for 48 h (*n* = >50). The data are an average of three independent biological replicates. Scale bar, 5 µm. (**C**) Representative images and quantification of PICH coated, RPA-coated and dual PICH-RPA-coated UFBs in BLM degron cells with (+IAA) or without (−IAA) BLM degradation exclusively during mitosis in the presence of RS (0.3 µM Aph) (*n* = >50). The data are an average of two independent biological replicates. Scale bar, 5 µm. (**D**) Experimental workflow for assessing abscission time in U2OS cells with and without RS and in presence or absence of an RPA inhibitor (RPAi) during mitosis. (**E**, **F**) Representative still pictures (**E**) and quantification (**F**) of live-cell imaging (*n* = >50) of U2OS cells (with no chromatin bridges) stably expressing fluorescently tagged histone H2B (red) and α-tubulin (green) treated with either DMSO or RPA inhibitor (HAMNO, 50 µM) during mitosis in presence (+Aph) and absence (-Aph) of RS (0.3 µM Aph). The yellow arrows indicate the site of abscission. The data are an average of three independent biological replicates with error bars representing the standard deviation. The significance was calculated using a two-tailed Mann–Whitney test. Exact *p* values are 0.31 (n.s.) and <0.0001 (****). Scale bar, 10 µm (**E**). (**G**) Quantification of abscission time derived from live-cell imaging (*n* =>15) of RPE cells (with no chromatin bridges). BLM^−/−^ cells expressing GFP-BLM-WT (BLMwt) or GFP-BLM-ATPase-dead (BLMmut) were compared with and without RS. The data are an average of three independent biological replicates with error bars representing the standard deviation. The significance was calculated using a two-tailed Mann-Whitney test. Exact *p* values between –Aph and +Aph are 0.0007 (***) for BLM^WT^, 0.506 (ns) for BLM^−/−^, < 0.0001 (****) for BLM^−/−^ BLMwt and 0.014 (*) for BLM^−/−^ BLMmut. (**H**) Representative still pictures of live-cell imaging of RPE cells stably expressing GFP tagged BLM (green) and fluorescently tagged α-tubulin (red) in presence of Sir-DNA (blue) to visualize chromatin. BLM^−/−^ cells expressing GFP-BLM-WT (BLMwt) or GFP-BLM-ATPase-dead (BLMmut) were compared in presence of RS. The green arrows indicated the GFP-BLM bridges during anaphase and yellow arrows indicate the site of abscission. Scale bar, 10 µm. Source data are available online for this figure.

Next, we addressed how the presence of BLM on UFBs might signal to the abscission checkpoint machinery. Since BLM is a DNA helicase and has been shown to mediate the conversion of double-stranded UFBs (coated by PICH) to single-stranded UFBs (coated by RPA) (Chan et al, 2018), we hypothesized that RPA might play a key role in abscission checkpoint signaling. RPA was an attractive candidate because of its established role as a checkpoint mediator during S-phase (Cimprich and Cortez, 2008). To investigate this, we first sought to verify BLM’s role in unwinding PICH-coated UFBs in our cell lines following Aph treatment, which was not studied as a form of genotoxic stress in the previously published study (Chan et al, 2018). For this, we determined the frequency of PICH- and RPA-coated UFBs in control U2OS cells (designated BLM^WT^) and in U2OS cells in which the *BLM* gene was inactivated using CRISPR-Cas9-mediated gene targeting (designated BLM^−/−^) (Loe et al, 2020). Consistent with previously published data (Barroso-González et al, 2021), we observed that Aph induced both PICH- and RPA-coated UFBs in BLM^WT^ cells. In contrast, Aph induced almost exclusively PICH-coated UFBs in BLM^−/−^ cells (Fig. 2A,B). We confirmed this observation using the BLM degron system (Fig. 2C), indicating that the ability of BLM to modulate the structure of UFBs requires its action only in mitosis. Given that the proteins required for abscission checkpoint signaling coalesce on the midbody in late mitosis (Mierzwa and Gerlich, 2014), we also confirmed that control cells exposed to Aph displayed RPA-coated UFBs that in some cases persisted until very late in mitosis when midbody formation was already evident (Fig. EV4A).

**Figure EV4:**

Mitotic inhibition of RPA prevents replication stress-induced abscission delay. (**A**) Representative immunofluorescence images (top) and bright field images (bottom) of the same U2OS cell (with visible midbody formation), treated with low dose Aph (0.3 µM during interphase) and stained with an anti-RPA antibody and DAPI. Scale bar, 10 µm. (**B**) Representative still pictures of live-cell imaging (*n* = >60) of U2OS cells (with no visible chromatin bridges) stably expressing fluorescently tagged histone H2B (red) and α-tubulin (green) treated with either DMSO or an RPA inhibitor (HAMNO, 50 µM) during mitosis in the absence of RS. The yellow arrows indicate the site of abscission. Scale bar, 10 µm. (**C**, **D**) Representative still pictures (**C**) and quantification (**D**) of live-cell imaging (*n* = >30) of U2OS cells (with no visible chromatin bridges) stably expressing fluorescently tagged histone H2B (red) and α-tubulin (green) treated with either DMSO or an RPA inhibitor (TDRL-505, 50 µM) during mitosis in the presence (+Aph) or absence (-Aph) of RS (0.3 µM Aph). The yellow arrows in (**C**), indicate the site of abscission. Scale bar, 10 µm. The data are an average of two independent biological replicates with error bars representing the standard deviation. A Mann-Whitney test was performed to derive significance. Exact *p* values are 0.490 (ns) and 0.0028 (**). Source data are available online for this figure.

To test the hypothesis that RPA signals the presence of UFBs to the abscission checkpoint machinery, we exposed cells to either of two previously validated RPA inhibitors, HAMNO (which obstructs RPA’s downstream function by inhibiting its interaction with ATRIP/ATR) (Glanzer et al, 2014) and TDRL-505 (which prevents DNA binding of RPA protein) (Shuck and Turchi, 2010). These inhibitors were only added to cells from the time of metaphase through to abscission. We then determined abscission timing using live cell imaging (Fig. 2D). We observed that treatment of U2OS cells with either HAMNO or TDRL-505 was sufficient to prevent the characteristic Aph-induced abscission delay (Figs. EV4B–D, 2E,F and Movie EV3). Importantly, these RPA inhibitors, which have different modes of action, did not affect abscission timing in unperturbed cells (Figs. 2F and EV4D). These data suggest that BLM’s role in abscission is associated with the formation of single-stranded UFBs coated with RPA.

Next, we analyzed the role of BLM’s helicase activity in the abscission checkpoint. Since the unwinding of dsDNA bridges to form RPA-coated ssDNA bridges would require BLM’s helicase activity, we reasoned that a helicase-dead mutant of BLM shall fail to signal to the abscission machinery during cytokinesis. To address this, we conducted analysis of abscission timing by live cell imaging using derivatives of RPE BLM^−/−^ cells (Tsukada et al, 2024) ectopically expressing either GFP-BLM or a GFP-BLM helicase-dead variant (K695R). We also used parental RPE cells (BLM^WT^) and BLM-inactivated RPE cells (BLM^−/−^) as controls. As predicted, BLM^WT^ cells and BLM^−/−^ cells expressing the BLM gene (BLM^−/−^ BLMwt) showed a delay in the timing of abscission following RS (Fig. 2G,H). In contrast, RPE BLM^−/−^ cells, and the derivative expressing only ATPase-dead BLM (BLM^−/−^ BLMmut) failed to display any abscission delay (Fig. 2G,H). We confirmed that this failure of the ATPase-dead variant to function in abscission regulation was not because it was unable to localize to UFBs (Fig. 2H). These data confirm a role for the helicase activity of BLM in triggering abscission delay.

### The role of RPA in regulating abscission does not directly require BLM

**Figure 3:**
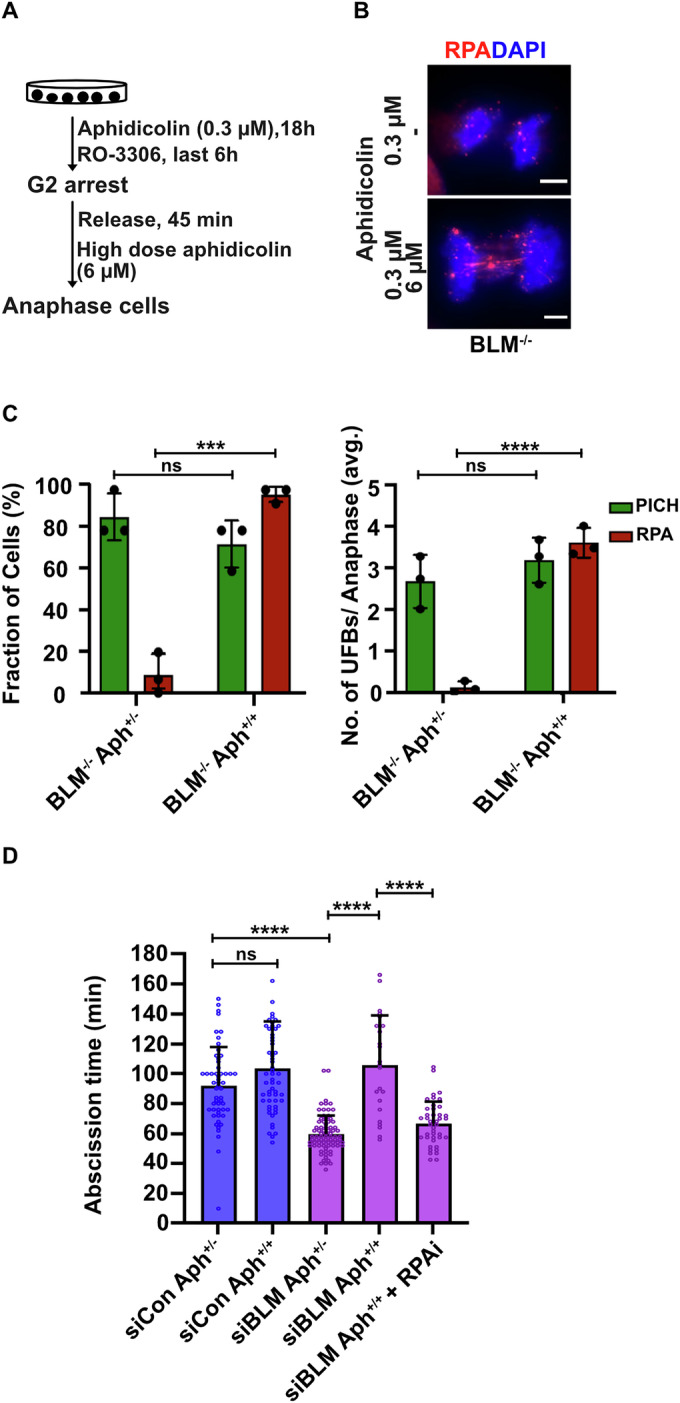
RPA-coated UFBs generated by BLM delay abscission. (**A**–**C**) Experimental workflow (**A**), representative images (**B**) and quantification (**C**) of anaphase cells with at least one PICH or RPA UFB (*n* = >50) (left) and average number of PICH and RPA UFBs (*n* = >50) (right) in BLM^−/−^ cells under the indicated conditions. The cells were treated with low dose Aph (0.3 µM) during interphase and then exposed or not to high dose Aph (6 µM) during mitosis. The data are an average of three independent biological replicates with error bars representing the standard deviation. The significance was calculated using a t-test. Exact *p* values in (**C**) are 0.230 (ns), 0.0001 (***) in the graph on the left and 0.35 (n.s.), <0.0001 (****) in the graph on the right. Scale bar, 5 µm (**B**). (**D**) Quantification of abscission time derived from live-cell imaging of siCon or siBLM treated U2OS cells (with no visible chromatin bridges). Cells were exposed to low dose Aph during interphase and then exposed (Aph^+/+^) or not (Aph^+/−^) to high dose Aph during mitosis in the presence or absence of RPA inhibitor (HAMNO, 50 µM) (*n* = >30). The data used for siCon Aph^+/−^ and siBLM Aph^+/−^ are the same as those used in Fig. 2F and Fig. 1D, respectively, as these experiments were all performed together. The data are an average of three independent experiments with error bars representing the standard deviation and Mann–Whitney test was performed to get the significance. Exact *p* values are 0.089 (n.s.) and <0.0001 (****). Source data are available online for this figure.

We investigated whether RPA bound to ssDNA might act autonomously as a signaling platform and only require BLM in cases where a UFB formed in early anaphase comprises dsDNA (which is the case for the vast majority of UFBs). To address this, we sought a means of generating RPA-coated UFBs in a BLM-independent manner. Following exposure to Aph in interphase (RS), cancer cells often fail to complete replication at common fragile site loci (Minocherhomji et al, 2015). Upon mitotic entry, these under-replicated regions activate a recombination-based DNA repair pathway called MiDAS that displays hallmarks of break-induced replication (BIR) (Bhowmick et al, 2016). The DNA synthesis step of MiDAS requires DNA polymerase δ and hence can be inhibited by a high dose of Aph (2–4 µM) applied to cells in mitosis (Macheret et al, 2020), as is routinely used to arrest S-phase replication (Krokan et al, 1981). We observed that this treatment generated numerous RPA-coated UFBs in U2OS cells (Appendix Fig. S2A). Importantly for our purposes, these ssDNA bridges still arose in BLM-depleted cells (Fig. 3A–C). Hence, we addressed whether inducing the formation of RPA-coated UFBs in BLM-depleted cells in this way could bypass the usual requirement of BLM for inducing abscission delay. As expected, BLM-proficient cells showed an abscission delay with or without inhibition of MiDAS following RS. However, we observed that inhibition of MiDAS was also able to induce abscission delay (following RS) in BLM-depleted cells, and that this delay could be prevented by treating cells with an RPA inhibitor during late mitosis (Fig. 3D and Appendix Fig. S2B). It should be noted that the overall abscission delay upon MiDAS inhibition is similar in BLM-proficient and -deficient cells. These findings indicate that the presence of an RPA-coated ssDNA bridge is sufficient to induce abscission delay and strongly suggest that BLM’s role is predominantly (or perhaps exclusively) to convert dsDNA bridges to ssDNA bridges through its DNA unwinding activity.

Because BLM is known to be recruited by PICH to centromeric anaphase bridges (the most common variety), it might have been expected that PICH depletion would phenocopy BLM depletion. However, contrary to this view, we observed an extension in the period of cytokinesis in Aph treated cells in which PICH was depleted using siRNAs. However, it has been shown previously that PICH is not needed for BLM to be recruited to all types of bridges, and hence PICH-depleted cells can still contain BLM-coated bridges (Rouzeau et al, 2012). To investigate this issue directly using our cell treatment conditions, we analyzed whether PICH-depleted cells might display RPA-coated bridges. We observed that, in contrast to BLM-depleted cells, RPA bridges were still present in PICH-depleted cells (Appendix Fig. S2C). We propose that BLM could either bind directly to certain ssDNA regions on Aph-induced bridges in a PICH-independent manner (such as sites of incomplete replication) or might be recruited by RPA, as proposed previously (Shorrocks et al, 2021). Either way, this would permit BLM to unwind the bridge DNA and would provide a plausible means for the generation of RPA-coated bridges that trigger the checkpoint and cause abscission delay in PICH-depleted cells.

### RPA signals to Aurora B through CHK1

**Figure 4:**
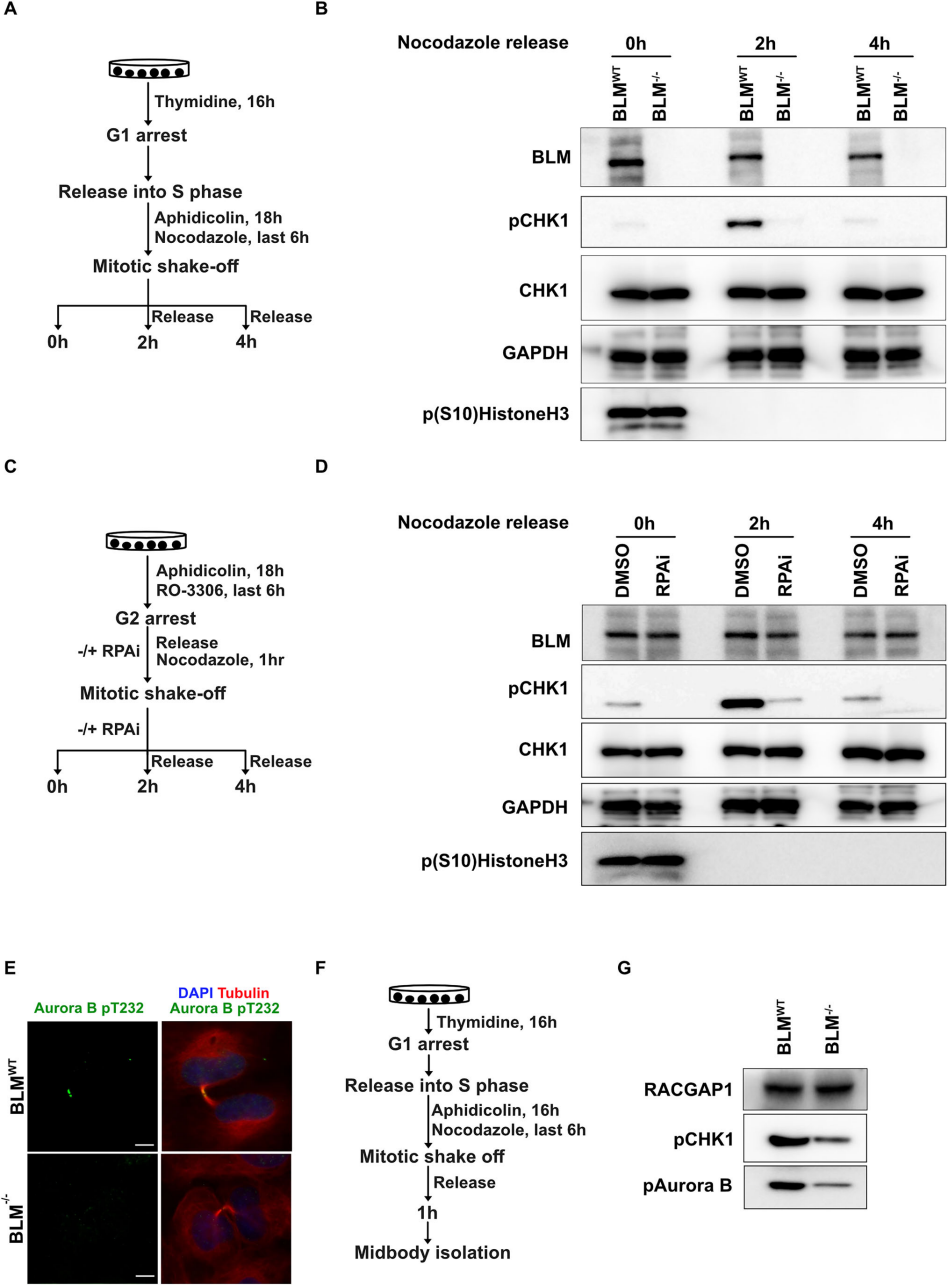
RPA signals to Aurora B through ChK1. (**A**, **B**) Experimental workflow (**A**) and representative western blots with the indicated antibodies (**B**) of whole cell lysates derived from BLM^WT^ and BLM^−/−^ U2OS cells, arrested with nocodazole and released for the indicated time, in presence of RS. The data are representative of two independent experiments. (**C**, **D**) As per panels (**A**, **B**), except that BLM^WT^ cells were treated with DMSO or RPA inhibitor (50 µM, HAMNO) exclusively during mitosis. The data are representative of two independent experiments. (**E**) Representative immunofluorescence image of cytokinetic cells (treated with Aph during interphase) with antibodies for phosphorylated (T232) Aurora B (green), Tubulin (red). DNA was stained with DAPI (blue). Scale bar, 10 µm. (**F**, **G**) Experimental workflow (**F**) and representative western blot (**G**) of midbody lysates derived from BLM^WT^ and BLM^−/−^ U2OS cells with the indicated antibodies. The data are representative of three independent experiments. Source data are available online for this figure.

During DNA replication, RPA activates the intra-S-phase checkpoint by promoting the ATR/ATRIP-dependent phosphorylation of checkpoint protein kinase 1 (CHK1) (Cortez et al, 2001). Interestingly, CHK1 is also important for cytokinesis, and inhibition of CHK1 induces cytokinesis failure and chromosomal instability (Mackay and Ullman, 2015). To investigate whether the ATR-CHK1 axis might perform a role in the UFB-activated abscission checkpoint that is analogous to its role in S-phase, we released BLM^WT^ and BLM^−/−^ cells from an arrest in prometaphase (induced by Nocodazole) and then assessed CHK1 activation throughout the course of cytokinesis (Fig. 4A; Appendix Fig. S3A). In the presence of RS, BLM^WT^ cells showed strong CHK1 activation 2 h after release from Nocodazole arrest (discussed further in the Methods). In contrast, BLM^−/−^ cells failed to fully activate CHK1 over a 6-h period after release (Fig. 4B). To study the connection between UFBs and CHK1 activation, we addressed whether the ability of BLM^WT^ cells to activate CHK1 in response to RS was driven by RPA-coated ssDNA bridges. For this, we applied an RPA inhibitor during mitosis and evaluated CHK1 activation during cytokinesis (Fig. 4C; Appendix Fig. S3B). We observed that the addition of an RPA inhibitor in prometaphase was sufficient to inhibit CHK1 activation during cytokinesis even in BLM-proficient cells (Fig. 4D). As a control, we analyzed CHK1 phosphorylation in BLM^WT^ and BLM^−/−^ cells in absence of RS but failed to detect any activation of CHK1 (Appendix Fig. S3C).

**Figure EV5:**
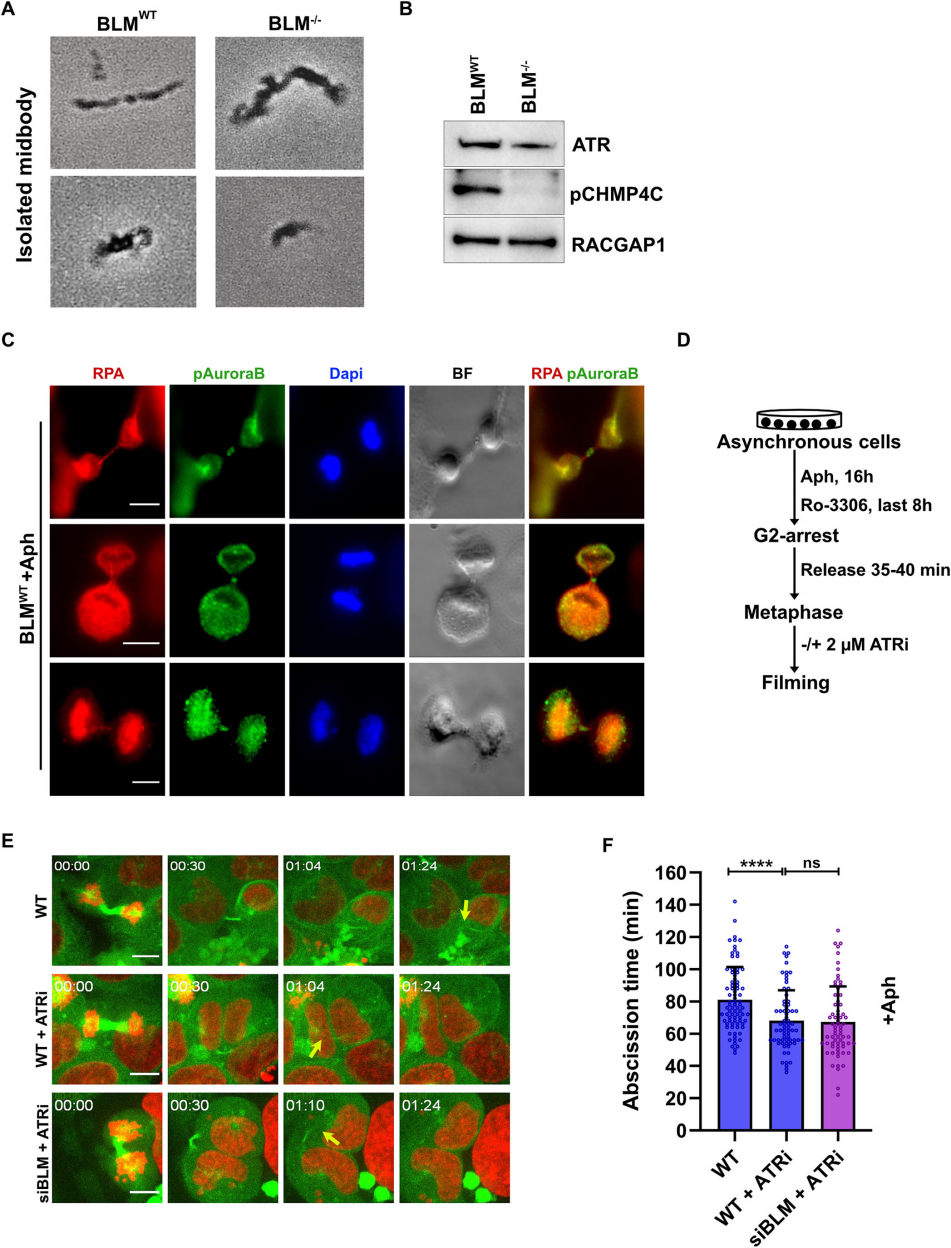
Mitotic inhibition of ATR prevents replication stress-induced abscission delay. (**A**) Images of purified midbodies from BLM^WT^ and BLM^−/−^ cells. (**B**) A representative western blot of midbody lysates derived from BLM^WT^ and BLM^−/−^ U2OS cells with the indicated antibodies. The data are an average of two independent experiments. (**C**) Representative immunofluorescence images of BLM^WT^ cells showing an RPA coated persistent bridge passing through the midbody depicted through bright field (BF) microscopy, with active Aurora B (pThr-232) at the midbody and following replication stress. Scale bar, 10 µm. (**D**–**F**) Experimental workflow (**D**), still pictures (**E**) and quantification (**F**) of live-cell imaging (*n* = >60) of U2OS cells (with no visible chromatin bridges) stably expressing fluorescently tagged histone H2B (green) and α-tubulin (red) treated with either DMSO or an ATR inhibitor (2 µM, VE-822) during mitosis following exposure to RS (0.3 µM Aph) in interphase. The yellow arrows in (**E**) indicate the site of abscission. The data are an average of three independent experiments with error bars representing the standard deviation. A Mann-Whitney test was performed to derive significance. Exact *p* values are <0.0001 (****) and 0.554 (n.s.). Scale bar in (**E**), 10 µm. Source data are available online for this figure.

CHK1 is proposed to act upstream of Aurora B, which then governs the activation of the abscission checkpoint (Mackay and Ullman, 2015). Phosphorylation of Aurora B on Thr-232 is known to be a mediator of checkpoint activation (Yasui et al, 2004), and therefore we assessed whether this specific phosphorylation event occurs normally or not in cells lacking BLM. We observed that, as expected, BLM^WT^ cells treated with Aph showed an accumulation of pThr-232 Aurora B at the midbody, especially within the so called midbody arms. In contrast, BLM-deficient cells showed only mild activation of Aurora B even in the presence of Aph (Fig. 4E). Since the pThr-232 signal was present on the arms of the midbody and not at its center, it was difficult to quantify this difference by reference to a control protein (such as RACGAP1) that lies at the midbody center. For this reason, we used an orthogonal method to quantify the extent to which pThr-232 levels were altered. We purified a midbody fraction from Aph-treated BLM^WT^ and BLM^−/−^ cells and assessed the level of pThr-232 Aurora B and pSer-345 CHK1 using western blotting (Figs. 4F,G and EV5A). Consistent with the immunofluorescence data, we observed a significantly reduced accumulation of pThr-232 Aurora B in the midbody fraction in BLM^−/−^ cells (Fig. 4G).

A recent study showed that ATR localizes constitutively to the midbody in HeLa cells, even in the absence of RS (Luessing et al, 2022). Hence, we investigated whether ATR is present in the midbody fraction alongside pSer-210 CHMP4C protein because association of ATR with RPA-coated ssDNA in S-phase triggers the replication checkpoint upstream of CHK1, and CHMP4C is known to influence abscission timing in an Aurora B-dependent manner (Fig. EV5B). We observed that the level of ATR in the midbody fraction in BLM^−/−^ cells was reduced compared to that in BLM^WT^ cells, suggesting that at least some fraction of ATR is recruited to the midbody upon association with RPA-coated ssDNA bridges. Based on an analogy with the S-phase checkpoint, we would suggest that ATR activation most likely occurs directly on RPA-coated UFBs, which then triggers CHK1 activation in the midbody. In this regard, it might be significant that CHK1 possesses a KA1 domain, which is known to target protein kinases to the site of cytokinesis (bud-neck) in budding yeast (Hadders and Williams, 2010). We propose that midbody-associated CHK1 then phosphorylates Aurora B, as seen in Fig. EV5C which in-turn phosphorylates CHMP4C, leading to abscission checkpoint activation. Thus, BLM^−/−^ cells exhibit very limited phosphorylation of CHMP4C in response to Aph (Luessing et al, 2022). This reduction in the level of activated Aurora B and CHMP4C in BLM^−/−^ cells can explain the lack of a delay in abscission timing in BLM^−/−^ cells even in presence of Aph (Figs. 4G and EV5B). Also, BLM is known to be phosphorylated by CHK1 (Kharat et al, 2016). It is possible that this serves as a positive feedback loop for activation of abscission checkpoint.

To provide additional evidence for a critical role of the RPA-ATR-CHK1 axis in the abscission checkpoint, we analyzed the effect of addition of a validated ATR inhibitor exclusively during mitosis (Fig. EV5D). We observed that the ATR inhibitor also prevented abscission delay induced by Aph (Fig. EV5E,F). Inhibiting ATR in this way in BLM-depleted cells did not produce any additive effect on abscission duration, suggesting that ATR and BLM are likely to operate in the same pathway (Fig. EV5F). Taken together, these data not only define a mechanistic role for RPA in activation of the abscission checkpoint but also reveal that human cells use a similar checkpoint activation pathway (the ATR-CHK1 axis) to regulate cell cycle transitions in both S-phase and mitosis.

### Evidence that aneuploidy in BLM-deficient cells is dependent upon BLM’s role in cytokinesis

**Figure 5:**
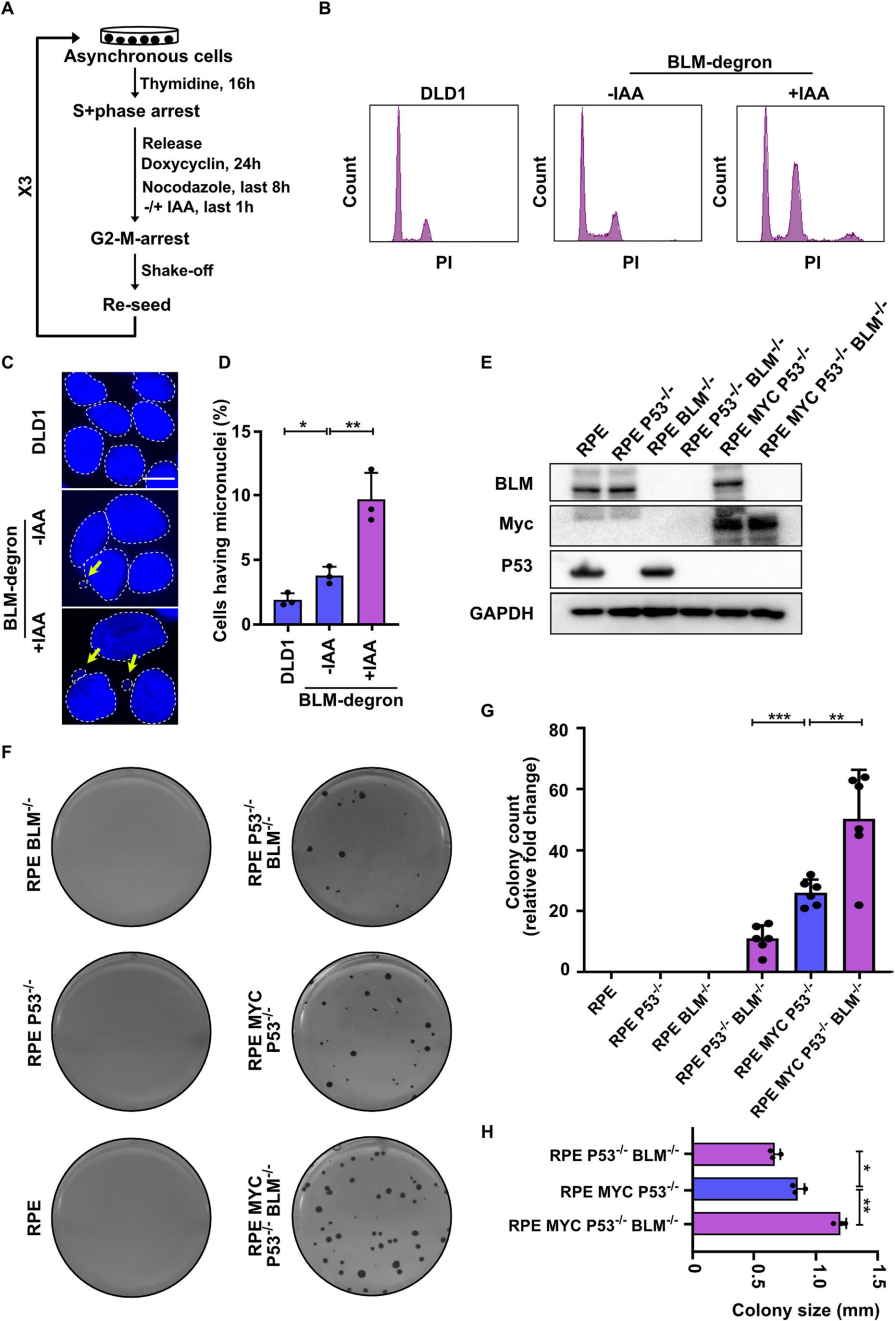
Abscission checkpoint override generates aneuploidy. (**A**) Experimental workflow for analyzing the cell cycle profile of BLM degron cells after degradation of BLM over three generations exclusively during mitosis. (**B**) Representative cell cycle profile of BLM degron cells after the indicated treatment. The data are representative of two independent experiments. (**C**, **D**) Representative immunofluorescence images (**C**) and quantification (**D**) of micronuclei in asynchronously growing BLM degron cells after being treated as indicated in (**A**). Scale bar is 5 µm and the yellow arrows indicate micronuclei. The nucleus and micronucleus are encircled using a dotted lines for the ease of visualization in (**C**). The data in (**D**) are an average of three independent experiments (*n* => 300) and error bars represent standard deviation. Significance was derived using a t-test. Exact *p* values are 0.018 (*) and 0.009 (**). (**E**) Representative western blot of whole cell lysate from indicated cell lines with antibodies against BLM, MYC, P53, GAPDH antibody. The data are representative of two independent biological replicates. (**F**) Representative images of a soft agar assay with the indicated different cell lines. The data are representative of six independent biological replicates. (**G**) Number (fold change compared to RPE) of colonies grown on soft agar generated by the indicated cell lines. The data are an average of six independent biological replicates and error bars represent standard deviation. Statistics were calculated using a two-tailed t-test. Exact *p* values are 0.0001 (***) and 0.005 (**). (**H**) Size (mm) of colonies grown on soft agar generated by the indicated cell lines. The data are an average of three independent biological replicates and error bars represent standard deviation. Statistics were calculated using a two-tailed t-test. Exact *p* values are 0.010 (*) and 0.001 (**). Source data are available online for this figure.

One of the main features of cells from BS individuals is an increased frequency of aneuploidy and micronuclei (Chester et al, 2006). BLM is known to play crucial roles in DNA replication and repair (Manthei and Keck, 2013), and we have shown here a new role in promoting the abscission checkpoint. Given that binucleation resulting from aberrant cytokinesis might underlie the generation of aneuploidy in BS cells, we investigated whether it might be possible to induce aneuploidy in a diploid cell line through inhibiting the function of BLM only in mitosis. For this, we used the BLM degron system described above that was created in DLD1 cells, a diploid line lacking functional p53 (Leroy et al, 2014). DLD1 cells were used because the continued survival of binucleated cells is generally prevented by p53, which activates senescence or apoptosis in response to abnormalities in ploidy (Andreassen et al, 2001; Horii et al, 2015). We degraded BLM only during mitosis for three generations before assessing aneuploidy/polyploidy and levels of micronuclei (Fig. 5A). To achieve this, we arrested cells in prometaphase and degraded BLM for an hour before releasing cells in IAA free media. Since removal of auxin should allow restoration of BLM levels in the next cell cycle. We observed that degradation of BLM during mitosis was sufficient to increase the frequency of aneuploidy/polyploidy from 2–4% prior to BLM degradation to 10–12% after three cycles of degradation (Fig. 5B). A similar increase was observed in the frequency of micronuclei (Fig. 5C,D). These data are consistent with the cytokinetic function of BLM making a significant contribution to the generation of aneuploidy in BS patients. However, we cannot completely exclude the possibility of a role for BLM in interphase because we observed that, following mitotic degradation of BLM, cells only partially recovered BLM expression by the time of entry into the subsequent S phase (Fig. EV2D).

### BLM deficiency promotes anchorage-independent growth of non-cancer cells

BLM patients are known to be predisposed to cancer (Cunniff et al, 2017). A relationship between aneuploidy and tumorigenesis is well established, since aneuploidy is able to stimulate cancer evolution by increasing the probability that pro-neoplastic genetic changes (driver mutations) are tolerated (Holland and Cleveland, 2009). We therefore analyzed whether BLM’s absence might confer upon untransformed cells an ability to proliferate in an anchorage-independent manner, which is a key feature of neoplastic transformation. For this, we analyzed the ability of RPE cells (an immortalized, but untransformed, human epithelial cell line) to grow in soft agar. To address whether BLM deficiency alone could promote anchorage-independent growth or whether it could potentiate the ability of a classical oncogene to promote such growth, we utilized a derivative of RPE cells in which p53 was inactivated (which would allow any BLM deficiency-driven aneuploid cells to survive) and the *MYC* oncogene was overexpressed in an inducible manner. We then compared BLM^WT^ and BLM^−/−^ derivatives of these cells in a soft agar colony formation assay, which has been used extensively in previous studies to monitor cellular transformation (Borowicz et al, 2014) (Fig. 5E,F). As expected, we observed that RPE cells, RPE-p53^−/−^ cells and RPE-BLM^−/−^ cells failed to form visible colonies in soft agar (Fig. 5G,H). In contrast, RPE-p53^−/−^-BLM^−/−^ cells generated a limited number of slow growing colonies, suggesting that BLM deficiency alone can trigger some degree of cellular transformation in a p53-deficient background (Fig. 5G,H). As reported previously (Facchini and Penn, 1998), we observed that overexpression of *MYC* in combination with p53 deficiency led to the formation of numerous, fast-growing, transformed colonies. Importantly, however, both the average size and the number of these colonies was increased significantly in cells additionally lacking functional BLM (Fig. 5G,H).

**Figure 6:**
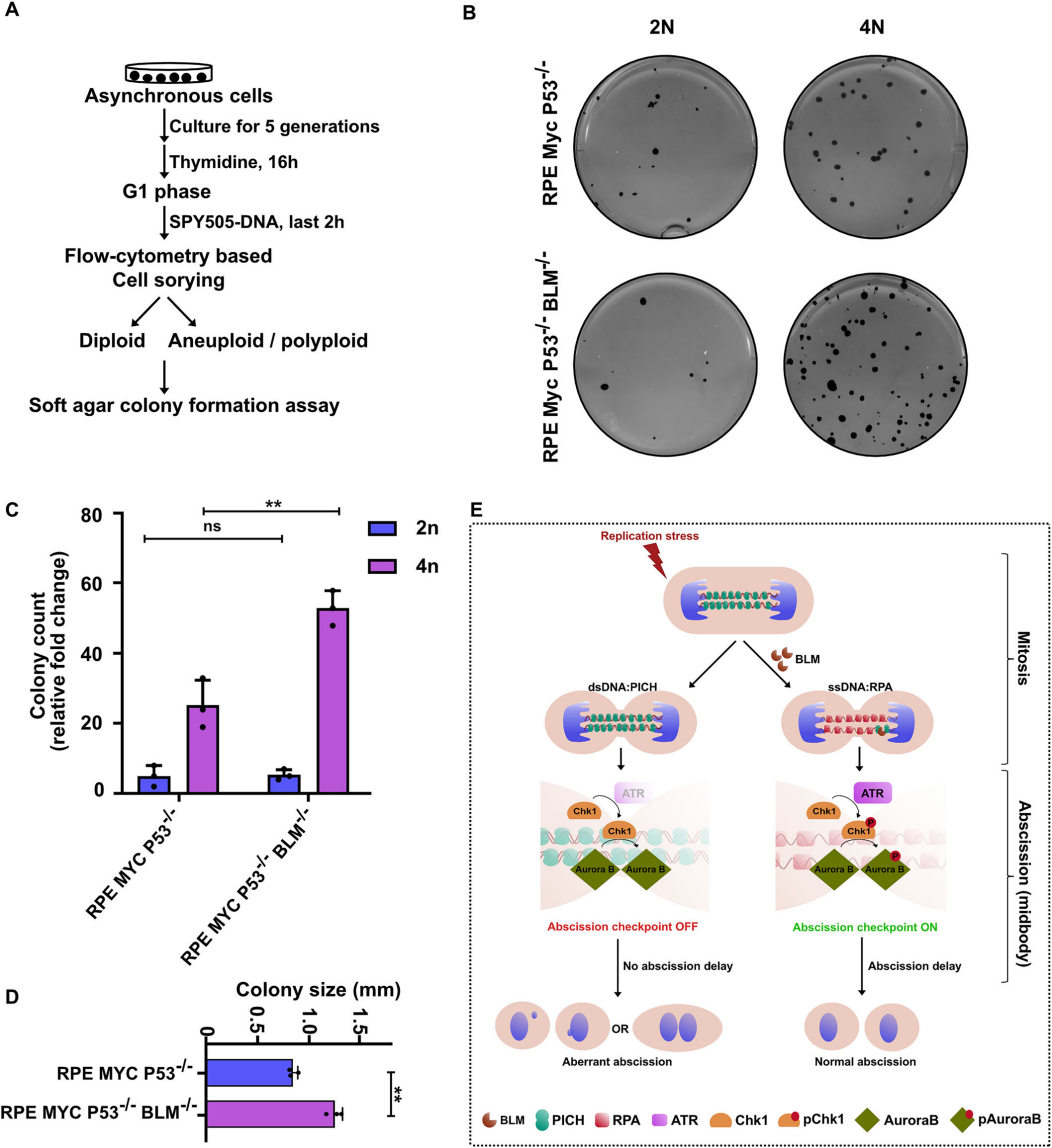
Aneuploidy assists cellular transformation. (**A**) Experimental workflow for sorting diploid and aneuploid/polyploid cell populations and performing soft agar colony formation assays. (**B**) Representative images of a soft agar assay with either diploid or aneuploid/polyploid cells. (**C**, **D**) Fold increase (compared to RPE) (**C**) and size (in mm) of colonies (**D**) grown in soft agar generated by the indicated cell lines. The data are an average of three independent biological replicates and error bars represent standard deviation. Statistics were calculated using a two-tailed t-test. Exact *p* values are 0.468 (n.s.) and 0.007 (**) in (**C**) and 0.003 (**) in (**D**). (**E**) Working model describing BLM’s cytokinetic role in activating the abscission checkpoint by the ATR-CHK1-Aurora B axis in the presence of persistent UFBs to maintain chromosome stability. Source data are available online for this figure.

To strengthen the connection between aneuploidy generated by BLM deficiency and the promotion of anchorage-independent growth, we overexpressed MYC in either RPE-p53^−/−^ or RPE-p53^−/−^BLM^−/−^ cells for 5 generations to promote the development of aneuploidy. We then flow-sorted the diploid and aneuploid populations from these cultures and performed soft agar colony formation assays (Fig. 6A). We observed that the percentage of cells from the aneuploid, MYC-overexpressing, cell population that could form colonies was significantly higher than that from the corresponding diploid population (Fig. 6B,C). More significantly, BLM deficiency was found to increase the number and size of the colonies developed by the aneuploid population of RPE-p53^−/−^ cells overexpressing MYC (Fig. 6C,D). Taken together, these data indicate that BLM’s cytokinetic role may make an important contribution to the suppression of aneuploidy and suggest that a key driver of cancer predisposition in BS patients might be aneuploidy. While we cannot rule out the possibility that the interphase role of BLM acts to suppress anchorage-independent growth, we propose that a more plausible role for BLM in this regard is through a mitotic role that suppresses aneuploidy. We have outlined these proposals in a model presented in Fig. 6E.

### Concluding remarks

#### Moonlighting of the S phase checkpoint in mitosis

One of the most critical checkpoints utilized during the human cell division cycle is the intra-S phase checkpoint. This checkpoint, which is governed by RPA-ATR-CHK1 axis, is triggered by the presence of ssDNA generated by different types of replication perturbation such as the uncoupling of the replicative helicase and polymerase. Here, we provide evidence that the same RPA-ATR-CHK1 axis is playing an important role in mitosis by maintaining the abscission checkpoint in an activated state in response to defects in sister chromatid disjunction. This checkpoint allows cells to resolve problematic anaphase DNA bridge structures prior to activating the ‘final cut’ (abscission) to complete cytokinesis. This ‘moonlighting’ role of the RPA-ATR-CHK1 axis in mitosis indicates that cells have evolved a mechanism to efficiently use the same proteins to maintain genome stability at different stages of the cell cycle.

#### Comparison of bulky chromatin bridges and UFBs

Given their radically different structure and composition, the processing of bulky chromatin bridges and UFBs would likely require different factors in anaphase. A recent study (Petsalaki and Zachos, 2021b) demonstrated that the ATM-CHK2 pathway activates the abscission checkpoint. We found that UFB-induced abscission delay is dependent instead on ATR-CHK1. The low dose Aph treatment used in our experiments primarily induces UFBs, while chromatin bridges are only observed in a very small fraction of the cells. It has been reported by others (Mackay and Ullman, 2015) that low-dose Aph-induced abscission delay is dependent on ATR-CHK1 and is independent of ATM, as we observed. One explanation for these apparently contradictory results is that the BLM-RPA-ATR axis controls a checkpoint that responds to UFBs, whereas ATM-CHK2 promotes checkpoint activation in response to chromatin bridges. Consistent with this, a role for Topoisomerase IIa-associated cleavage complexes (which are associated with dsDNA breaks) in the activation of the abscission checkpoint by chromatin bridges was identified recently (Petsalaki et al, 2023). Whether this mechanism for influencing the checkpoint operates with some forms of UFBs will require future studies. While Topoisomerase IIa is implicated in processing UFBs arising from centromeres, it is much less likely to be involved in the types of RS-induced UFBs that we have studied here, which probably represent under-replicated regions of genomic DNA.

#### BLM deficiency-driven aneuploidy assists tumorigenesis

It is well known that oncogene activation in the absence of the p53 tumor suppressor protein can trigger cellular transformation. In our study, we demonstrated that BLM deficiency acted additively with oncogene activation to promote the transformation of non-cancer cells. More surprisingly, we observed that the absence of BLM alone when combined with p53 deficiency could trigger a limited level of cellular transformation. These findings offer a potential explanation for the observation that BS patients are predisposed to a very wide range of different cancers, a phenotype that most likely requires a defect that impacts on the suppression of tumorigenesis in all tissue types. The role of BLM in prevention of aneuploidy would fulfill just such a fundamental tumor suppressor role.

## Methods

**Table:** Reagents and tools table

Reagent/Resource	Reference or Source	Identifier or CatalogNumber
**Experimental models**		
U2OS	ATCC	Cat# HTB-96
U2OS H2B-GFP mCherry-Tubulin	(Bhowmick et al, 2019)	N/A
U2OS BLM^−/−^	Gift from Dr. E. L. Denchi	N/A
DLD1-TET-OsTIR1	This study	N/A
DLD1 BLM degron	This study	N/A
DLD1 BLM degron H2B-mCherry Tubulin-GFP	This study	N/A
hTERT-RPE-1-MycER	Gift from Dr Ingram Iaccarino	N/A
hTERT-RPE-1	(Tsukada et al, 2024)	N/A
hTERT-RPE-1-BLM^−/−^	(Tsukada et al, 2024)	N/A
hTERT-RPE-1-BLM^−/−^-GFP-BLM WT	(Tsukada et al, 2024)	N/A
hTERT-RPE-1-BLM^−/−^-GFP-BLM K695R (ATPase dead)	(Tsukada et al, 2024)	N/A
hTERT-RPE-1 mScarlet-I-Tubulin	This study	N/A
hTERT-RPE-1-BLM^−/−^ mScarlet-I-Tubulin	This study	N/A
hTERT-RPE-1-BLM^−/−^-GFP-BLM WT mScarlet-I-Tubulin	This study	N/A
hTERT-RPE-1-BLM^−/−^-GFP-BLM K695R (ATPase dead) mScarlet-I-Tubulin	This study	N/A
hTERT-RPE-1-MycER-p53^−/−^	(Bhowmick et al, 2022)	N/A
hTERT-RPE-1-p53^−/−^	(Bhowmick et al, 2022)	N/A
hTERT-RPE-1-MycER-p53^−/−^ BLM^−/−^	This study	N/A
hTERT-RPE-1-p53^−/−^ BLM^−/−^	This study	N/A
DLD1-PICHNeon	Gift from Drs Y. Azuma and D. J. Clarke	N/A
**Recombinant DNA**		
pSpCas9(BB)-2A-GFP (PX458)	Addgene	Cat# 48138
pMK287	Addgene	Cat# 72825
pMK288	Addgene	Cat# 72826
pENTR4-GFP-C1	Addgene	Cat# 17396
pLenti-CMV/TO-Hygro DEST	Addgene	Cat# 17291
pLenti-CMV/TO-Hygro-GFP-Tubulin	This study	N/A
pLenti6-H2B-mCherry	Addgene	Cat# 89766
psPAX2	Addgene	Cat# 12259
pMD2.G	Addgene	Cat# 12260
pFA6a-mScarlet-mNeonGreen-kanMX	Addgene	Cat# 173454
pENTR4 no ccdB (686-1)	Addgene	Cat# 17424
pLenti CMV Puro DEST (w118-1)	Addgene	Cat# 17452
pLenti CMV Puro DEST (w118-1) mScarlet-I-Tubulin	This study	N/A
peGFP-C1-Ankle1	(Brachner et al, 2012)	N/A
pLVpuro-CMV-N-mCherry	Addgene	Cat# 123221
**Antibodies**		
All antibodies	N/A	Appendix Table S2
**Oligonucleotides and other sequence-based reagents**		
PCR Primers	This Study	Appendix Table S1
BLM-Linker-mAID-3UTR (Linear dsDNA)	This study	Appendix Table S1
P53 KO RNA guide: 5’ CAGAATGCAAGAAGCCCAGA 3’	(Zimmermann et al, 2018)	N/A
BLM KO RNA guide: 5’ GGGGACTGTTTACTGACTAC 3’	(Loe et al, 2020)	N/A
mScarlet-I_F primer: 5’ CCGCTCGAGCGATGG TGAGCAAGGGCGAG 3’	This study	N/A
mScarlet-I_R primer: 5’ ATGGACGAGCTGTACAAGTAATCTAGAGC 3’	This study	N/A
siBLM-1: 5′-CUAAAUCUGUGGAGGGUUA-3′	Dharmacon	L-007287-00-0005
siBLM-2: 5′-GCAACUAGAACGUCACUCA-3′	Dharmacon	L-007287-00-0005
siBLM-3: 5′-GAGAAACUCACUUCAAUAA-3′	Dharmacon	L-007287-00-0005
siBLM-4: 5′-GGAUGACUCAGAAUGGUUA-3′	Dharmacon	L-007287-00-0005
siPICH: 5′-AGUAGGUGGUGUCGGUUUA-3′	Dharmacon	Cat# L-031851-01
siNUP153: 5’-AAGGCAGACUCUACCAAAUGUTT-3’	(Zhou and Pante, 2010)	N/A
Non-targeting control siRNA: 5′-UGGUUUACAUGUCGACUAA-3′	Dharmacon Inc.	Cat# D-001810-01-05
**Chemicals, Enzymes and other reagents**		
Hygromycin B	Thermo Fisher Scientific	Cat# 10687010
Blasticidin	Thermo Fisher Scientific	Cat# R21001
Puromycin	Sigma Aldrich	Cat# P8833
4-hydroxy-tamoxifen	Merck	Cat# H7904
Verapamil	Spirochrome	N/A
SiR-DNA	Spirochrome	Cat# SC007
TDRL-505	Sigma-Aldrich	Cat# 5305350001
HAMNO	Axon Medchem	cat# NSC 111847
Berzosertib (VE-822)	Sellekchem	Cat.# S7102
Doxycycline	Thermo Fisher Scientific	Cat# 10687010
Auxin (IAA)	Santa Cruz Biotech	Cat# CAS 6505-45-9
RO-3306	Merck	Cat# SML0569
Thymidine	Sigma Aldrich	Cat# T1895
Nocodazole	Sigma Aldrich	Cat# M1404
Benzonase	Sigma Aldrich	Cat# E1014
**Software**		
Prism	Graphpad	https://www.graphpad.com/scientific-software/prism/
Fiji (ImageJ)	NIH	https://imagej.net/software/fiji/downloads
CellSens	Olympus	https://www.olympus-lifescience.com/en/software/cellsens/?gclid=EAIaIQobChMIkJXdwquT9QIVLI1oCR19FAUOEAAYASAAEgLtmPD_BwE
Affinity designer	Affinity	https://affinity.serif.com/en-gb/designer/
**Other**		
GeneJuice transfection reagent	Merck	Cat# 70967
Luminata Forte HRP substrate	Millipore	Cat# ELLUF0100
ECL	Thermo Fischer Scientific	Cat# 34580
OptiMEM	GIBCO	Cat# 31985062
Lipofectamine RNAiMAX	Thermo Fisher Scientific	Cat# 13778150
VectaShield mounting medium containing DAPI	Vector Laboratories	Cat# H-1200-10
Nitroblue tetrazolium chloride solution	Sigma-Aldrich	Cat# T4375

### Cell culture, cloning, and plasmids

U2OS (osteosarcoma; human female origin, ATCC Cat# HTB-96), U2OS H2B-GFP mCherry-Tubulin (Bhowmick et al, 2019), U2OS BLM^−/−^ cells (a gift from Dr. E. L. Denchi, National Cancer Institute, USA) (Loe et al, 2020) were maintained in DMEM media supplemented with 10% FBS and 1% penicillin-streptomycin-glutamine.

To generate a BLM degron in the DLD1 cell line (Cat# CCL-221, ATCC), pMK243 TET-OsTIR1-Puro and AAVS1 T2 CRIPR in pX330 (a kind gift from M. Kanemaki; Addgene, Cat# 72835 and Cat# 72833) (Natsume et al, 2016) plasmids were co-transfected using GeneJuice transfection reagent (Merck, Cat# 70967) according to manufacturer’s protocol and DLD1-TET-OsTIR1 cell line was generated. The clonal selection was done in presence of Puromycin (Invitrogen, Cat# ant-pr-1). Further, for endogenous tagging, the guide DNAs (gDNAs) targeting the 3′ UTR of the BLM gene were designed using Zhang Lab design resources (https://zlab.squarespace.com/guide-design-resources; MIT 2013). gDNA oligos were synthesized with BbsI/BpiI overhangs, which were then annealed and ligated into pSpCas9(BB)-2A-GFP (PX458) (Addgene, Cat# 48138) as described previously (Ran et al, 2013). Fragment of DNA containing 30 bp BLM (3′ end of BLM excluding the stop codon), FLAG tag, linker sequence, mAID sequence, and 3’UTR was commercially synthesized (Thermo Fisher Scientific) as a linear DNA (BLM-Linker-mAID-3UTR). The 855 bp (5′) and 951 bp (3′) homology arms flanking the BLM 3′UTR were generated by PCR using Q5 Hot start high-fidelity polymerase (NEB) with the “BLM-5′Arm_For” (forward)/“BLM-5′Arm_Rev” (reverse) and “BLM-3′Arm_For” (forward)/“BLM-3′Arm_Rev” (reverse) primers, respectively. The homology arms had 30 bp overlaps to both hygromycin gene (pMK287, Addgene, Cat# 72825) or blasticidin (BSR) gene (pMK288, Addgene, Cat# 72826) fragments cut by BamHI and pSK_BS as backbone cut by EcoRI. These fragments were assembled using Hifi Gibson Assembly (NEB). Assembled plasmid together with gDNA containing pSpCas9(BB)-2A-GFP plasmid were transfected into DLD1-TET-OsTIR1 cells using Neon transfection system (Thermo Fisher Scientific) according to manufacturer’s instructions. After 24 h of transfection, cells were selected with 250 µg/ml hygromycin and 7.5 µg/ml blasticidin for at least 12 days before colony selection. Correct clones were confirmed by PCR using BLM-EXT_For and BLM-EXT_Rev2 primers and western blotting. For live cell imaging of BLM degron cells, Tubulin A1B cDNA (MyBioSource, Cat# MBS1274063) was amplified by PCR and cloned into pENTR4-GFP-C1 vector (a kind gift from E. Campeau, Addgene, Cat# 17396) (Campeau et al, 2009) using Kpn1-TUB-F and BamH1-TUB-R primers. Following verification by sequencing, GFP-Tubulin was sub-cloned into pLenti-CMV/TO-Hygro DEST plasmid (a kind gift from E. Campeau, Addgene, Cat# 17291) (Campeau et al, 2009) by LR recombination (Invitrogen) according to manufacturer’s protocol to synthesize pLenti-CMV/TO-Hygro-GFP-Tubulin. BLM degron cells were transduced with pLenti6-H2B-mCherry (Addgene, Cat# 89766), pLenti-CMV/TO-Hygro-GFP-Tubulin, psPAX2 and pMD2.G using Metafectene (cat# T020-1.0, Biontex) following standard lentiviral transduction protocol. BLM degron cells were cultured in RPMI medium supplemented with 10% FBS and 1% penicillin-streptomycin-glutamine. All oligonucleotides used in this study are listed in Appendix Table S1.

hTERT-RPE-1-MycER cells were a gift from Dr Ingram Iaccarino, Institute of Genetics and Biophysics, Italy. hTERT-RPE-1-BLM^−/−^, hTERT-RPE-1, hTERT-RPE-1-BLM^−/−^-GFP-BLM WT and hTERT-RPE-1-BLM^−/−^-GFP-BLM K695R (ATPase dead) cells were a gift from Dr Andrew Blackford, University of Copenhagen, Denmark (Tsukada et al, 2024). To generate cell lines stably expressing fluorescent tubulin, mScarlet-I was amplified by PCR from pFA6a-mScarlet-mNeonGreen-kanMX (Addgene plasmid #173454) using the primers F-CCGCTCGAGCGATGG TGAGCAAGGGCGAG and R-ATGGACGAGCTGTACAA GTAATCTAGAGC and cloned into pENTR4 no ccdB (686-1) (Addgene plasmid #17424). Subsequently, the human Tubulin A1B cDNA was cloned in frame with mSacrlet-I to generate GATEWAY compatible entry vector encoding mScarlet-I TUBA1B and was confirmed by sequencing. Using LR recombination, mScarlet-I TUBA1B was subcloned into pLenti CMV Puro DEST (w118-1) (Addgene plasmid #17452). Stable cell lines expressing mScarlet-I-Tubulin were generated by lentiviral infection followed by selection with Puromycin (1 μg/ml).

The p53 gene was inactivated in hTERT-RPE-1-MycER and hTERT-RPE-1 cells using a specific guide RNA (5’ CAGAATGCAAGAAGCCCAGA3’) as described previously (Zimmermann et al, 2018). BLM was inactivated in hTERT-RPE-1-MycER-p53^−/−^ cells using a specific guide RNA (5’ GGGGACTGTTTACTGACTAC 3’) as described previously (Loe et al, 2020). All the RPE-1 derived cells were maintained in DMEM media supplemented with 10% FBS and 1% penicillin-streptomycin-glutamine. For overexpression of MYC, hTERT-RPE-1-MycER-p53KO (RPE p53^−/−^ MYC) cells were treated for 48 h with 50 nM 4-hydroxy-tamoxifen (MERCK, Cat. # H7904).

The Ankle1 cDNA cloned into a peGFP-C1 vector (a kind gift from Dr. Roland Foisner, Max F. Perutz Laboratories, Medical University, Vienna (Brachner et al, 2012)), was transiently transfected into U2OS H2B-GFP mCherry-Tubulin cells for analysis of abscission duration through live cell imaging.

DLD1 cells expressing PICH fused to a Neon fluorescent tag were a kind gift from Drs Y. Azuma and D. J. Clarke. These cells were transduced with lentivirus plasmid, pLVpuro-CMV-N-mCherry (gift from R. Ketteler, Addgene plasmid #123221), expressing mCherry-tubulin. Following viral transduction, single cell clones were isolated and were selected in the presence of 2 μg/ml puromycin (ant-pr-1, Invivogen). Unless indicated otherwise, all cell lines were obtained from the ATCC and are not among those listed as commonly misidentified by the International Cell Line Authentication Committee. All cell lines were subjected to karyotyping and STR profiling (ATCC) to confirm their identity and were routinely tested for the absence of mycoplasma contamination (using MycoAlert; Lonza Group Ltd, Cat. # LT07-318).

### Live-cell imaging

An incubation chamber with the temperature maintained at 37 °C and a controlled humidified atmosphere with 5% CO_2_ was used for live-cell imaging. A Plan-Apochromat 63x/1.4NA oil objective with differential interference contrast mounted on an inverted Zeiss Axio Observer Z1 microscope (Marianas Imaging Workstation [3i—Intelligent Imaging Innovations, Inc., Denver, CO, USA]), equipped with a CSU-X1 spinning disk confocal head (Yokogawa Corporation of America) and four laser lines (405, 488, 561, and 640 nm) was used. Images were acquired using an iXon Ultra 888 EM-CCD Camera (Andor Technology). Fifteen 1 μm-separated z-planes were collected every 2 min for 3–5 h. Cells with any visible chromatin bridges were not analyzed when determining abscission time, and only those cells that completed mitosis without inducing apoptosis were included in the analysis. Representative snapshots of live cell imaging with U2OS H2B-GFP mCherry-Tubulin were pseudo-colored (H2B-GFP; red, RFP-Tubulin; green) for visual clarity.

Analysis with hTERT-RPE-1, hTERT-RPE-1-BLM^−/−^, hTERT-RPE-1-BLM^−/−^-GFP-BLM WT and hTERT-RPE-1-BLM^−/−^-GFP-BLM K695R tagged with mScarlet-I-Tubulin and DLD1 neon-PICH/mCherry-Tubulin cells, were performed in the presence of 20 nM SiR-DNA (Spirochrome) and 10 µM Verapamil (Spirochrome) to stain the nuclear DNA (for detection in the far-red channel).

### Drug treatment

To inhibit RPA function in cells, either TDRL-505 (cat# 5305350001; Sigma-Aldrich) or HAMNO (cat# NSC 111847; Axon Medchem) was used at a concentration of 50 µM. These drugs were added only as cells neared the end of prometaphase. To generate a population of cells in prometaphase, cells were released from a G2/M arrest (induced by the CDK1 inhibitor RO-3306 used at 5 µM) for 30 min. Before adding the drugs to the medium, cell cycle status was confirmed by visualizing the characteristic pattern of chromosome condensation using a light microscope. For inhibiting the ATR kinase, Berzosertib (VE-822; Sellekchem, Cat.# S7102) was added at a concentration of 2 µM at the end of prometaphase as described above for the RPA inhibitors. To ensure immediate mixing of the drugs in the medium, a 4x solution of drugs was prepared in warm media before addition to the culture medium. To induce BLM degradation in the ‘degron’ cells, 100 ng/ml of doxycycline and 500 µM Auxin (IAA; Santa Cruz Biotech. Cat# CAS 6505-45-9) were added for 24 h and 1 h, respectively, prior to release from arrest at the G2/M boundary. Following release from the arrest, cells were washed with warm medium containing doxycycline and auxin and were then allowed to progress in presence of these drugs for further analysis.

### RNA interference

Wild-type U2OS cells or U2OS cells stably expressing both eGFP-histone H2B and mCherry-tubulin were seeded one day prior to transfection in culture medium without antibiotics. The medium was then exchanged for OptiMEM (GIBCO; Cat# 31985062), and the transfection was performed with 25 nM siRNAs for 48 h, using Lipofectamine RNAiMAX (Thermo Fisher Scientific; Cat# 13778150) following manufacturer’s protocol. The siRNAs used in this study were: siBLM, equimolar pool of 5′-CUAAAUCUGUGGAGGGUUA-3′; 5′-GCAACUAGAACGUCACUCA-3′; 5′-GAGAAACUCACUUCAAUAA-3′; 5′-GGAUGACUCAGAAUGGUUA-3′; siPICH, 5′-AGUAGGUGGUGUCGGUUUA-3′; siNUP153, 5′- AAGGCAGACUCUACCAAAUGUTT-3’ and non-targeting control siRNA (D-001810-01-05, Dharmacon Inc.), 5′-UGGUUUACAUGUCGACUAA-3′. All siRNAs were custom synthesized by Sigma Aldrich with a 3′dTdT addition. The efficiency of protein depletion was monitored by western blotting.

### Cell synchronization

To analyze the frequency of binucleation and micronucleus formation, cells were synchronized at the G2/M boundary using 5 µM RO-3306 (CDK1 inhibitor, MERCK, Cat# SML0569) for 6–8 h. G2/M synchronized cells were released by washing (three times) with warm medium and were then allowed to progress for 30 min into prometaphase. The prometaphase cells were shaken off and re-seeded onto poly-lysine-coated slides and were then allowed to progress for 3 h to enrich for cells in G1 before fixing and analysis. For UFB analysis, G2/M synchronized cells were washed and allowed to progress for 45–55 min before fixing. For midbody isolation or western blot analysis of cytokinetic cells, asynchronously growing cells were synchronized at the G1/S boundary using 2 mM thymidine (Sigma Aldrich; Cat# T1895) for 16 h. G1/S arrested cells were then washed with warm PBS (two times) followed by warm media (three times) to release them into S phase, incubated for 10 h and then exposed to Nocodazole (Sigma Aldrich; Cat# M1404) (50 ng/ml) for a further 6 h. The prometaphase arrested cells were shaken off and washed three times with warm media (37 °C) and then incubated for 1.5 h in suspension, for midbody isolation. For analysis of pCHK1 activation, the prometaphase cells purified by mitotic shake-off were reseeded for 2 h and 4 h on dishes with warm media to allow them to progress to late mitosis/early G1 (confirmed by visualization under a light microscope). It should be noted that the prometaphase cells re-seeded after shake-off show a delay in mitotic progression such that the cells approach abscission at around 2 h after re-seeding (Appendix Fig. S3A,B) (Mackay and Ullman, 2015). Because the BLM^−/−^ cells are more difficult to synchronize than the WT cells, we pre-synchronized them at the G1/S boundary using Thymidine and then at the G2-M boundary with RO-3306). Cells were released from G2 synchronization and then exposed to nocodazole to induce a prometaphase arrest prior to treatment with the RPAi. Where appropriate, asynchronous cells were subjected to siRNA-mediated depletion of proteins for 48 h before scoring the percentage of cytokinetic cells.

### Western blotting

Cells were harvested by trypsinization and then lysed in cell extraction buffer (10 mM Tris-HCl, pH 7.4, 100 mM NaCl, 1 mM EDTA, 1 mM EGTA, 1 mM NaF, 20 mM Na_4_P_2_O_7_, 2 mM Na_3_VO_4_, 1% Triton X-100, 10% glycerol, 0.1% SDS, 0.5% deoxycholate) supplemented with Protease Inhibitor Cocktail (Roche; Cat# 11836153001), PhosSTOP (Phosphatase Inhibitor Cocktail, Roche; Cat# 04906837001), PMSF (Phenyl-Methanesulfonyl Fluoride Solution; Sigma-Aldrich; Cat# 93482) and benzonase (Sigma Aldrich; Cat# E1014) for 30 min on ice with frequent vortexing. The cell lysates were then subjected to sonication with a Bioruptor Pico sonicator (Diagenode) for 10 cycles of 30 s on/30 s off, followed by centrifugation 12,000 × *g* for 10 min. Supernatants were stored at −80 °C or subjected to protein quantification using a BCA assay kit (Thermo Fischer Scientific; Cat# 23227). Finally, 15–30 µg of protein were boiled for 5 min in 4X NuPAGE LDS sample buffer (3:1 ratio) (Thermo Fischer Scientific; Cat# NP0007) and run on a NuPAGE Bis-Tris gels (4–12%) (Thermo Fischer Scientific; Cat# NP0322PK2) and transferred to a Hybond-PVDF membrane (Amersham Pharmacia; Cat# GE10600021). The membrane was blocked in 5% non-fat dry milk or 3% BSA in PBST for 1 h at room temperature and incubated overnight with the appropriate primary antibody re-suspended in 5% milk or 3% BSA in PBST at 4 °C. Following 3 × 10 min washes using PBST (1% Tween 20 in PBS), the membrane was incubated for 1 h at room temperature with the appropriate secondary antibody re-suspended in 5% non-fat dry milk or 3% BSA in PBST at room temperature, followed by 4 × 15 min washes using PBST. Luminata Forte HRP substrate (Millipore; Cat# ELLUF0100) or ECL (Thermo Fischer Scientific; Cat# 34580) was used for signal detection and images were captured on an Amersham Imager 600. All western blots were performed independently and at least twice. Primary and secondary antibodies are indicated in Appendix Table S2.

### Immunofluorescence

Cells on glass coverslips or poly-lysine glass slides were either pre-extracted (0.5% Triton X-100 in PBS) for 2 min on ice before fixation with 4% formaldehyde in PBS for 10 min or fixed and permeabilized using cold PTEMF buffer (20 mM PIPES, pH 6.8, 10 mM EGTA, 0.2% Triton X-100, 1 mM MgCl_2_ and 4% formaldehyde) for 10 min at room temperature. Fixed cells were washed with PBS three times and were blocked by incubation in 3% BSA, 0.1% Triton X-100 in PBS (PBSAT) for 20 min at room temperature followed by incubation with primary antibodies diluted in blocking buffer for overnight at 4 °C. Unbound primary antibody was removed by washing four times with PBSAT for 15 min each. Samples were then incubated with secondary antibodies for 60 min at room temperature, followed by 4 washes with PBSAT for 15 min each. Slides or coverslips were then briefly air-dried followed by mounting with VectaShield mounting medium containing DAPI (Vector Laboratories; Cat# H-1200-10). Images were captured using an Olympus BX63 microscope. Representative images were processed with Image J analysis software. Representative images of control and treatment samples were processed in an identical way. Primary and secondary antibodies are detailed in Appendix Table S2.

### Midbody purification

A Midbody enriched fraction was isolated as described previously (Bhowmick et al, 2019). Briefly, U2OS BLM^WT^ and BLM^−/−^ cells were synchronized at the G1/S boundary using thymidine (2 mM for 16 h). Cells were then washed three times with warm PBS to release them into S phase for 10 h in presence of Aph (0.3 µM) before the addition of Nocodazole (50 ng/ml) to the medium for 6 h. The prometaphase cells were shaken off and washed three times with warm medium (37 °C) and then incubated in suspension in warm medium for 1–1.5 h to allow them to progress to the cytokinetic phase (confirmed under a light microscope). Cytokinetic cells were harvested by centrifugation (200 × *g* for 3 min) and the pellet was gently resuspended using 25 volumes of the pellet in a hypotonic buffer (1 M hexylene glycol, 20 mM MgCl_2_, 2 mM PIPES, pH 7.2) for 2 min at room temperature. Swollen cells were then centrifuged at 200 × *g* for 3 min, and the pellet was vigorously resuspended (by vortexing for 1 min at the highest speed) in 50 volumes of warm (37 °C) lysis solution (1 M hexylene glycol, 1 mM EGTA, 1% Nonidet P-40, 2 mM PIPES, pH 7.2). Immediately after lysis, the tube was chilled in ice and 0.3 volumes of ice-cold pH lowering solution was added (1 M hexylene glycol, 50 mM MES, pH 6.3) followed by centrifugation (250 × *g* for 10 min) to remove large cell debris. The supernatant containing the midbody samples was then layered over a cushion of 40% glycerol (w/v) in 50 mM MES, pH 6.3 and then centrifuged at 2800 × *g* for 20 min to pellet the midbodies. The pellet was then washed (at 2800 × *g* for 20 min) three times with 50 mM MES, pH 6.3 before finally being resuspended in cell extraction buffer.

### Flow cytometry

Wild type DLD1 (DLD1-TET-OsTIR1) and BLM degron cells treated as described in Fig. 5A, or RPE p53^−/−^, RPE p53^−/−^ MYC and RPE p53^−/−^ BLM^−/−^ cultured for 5 generations were subjected to flow cytometry, as described previously (Minocherhomji et al, 2015). Briefly, cells were harvested by trypsinization and washed once with cold PBS followed by fixation with 70% ethanol at −20 °C overnight. Fixed cells were washed 2 times with 1% BSA in PBS. Then cells were treated with RNase A (final concentration 0.5 µg/ml) and propidium iodide solution (final concentration 50 µg/ml) in PBS for 20 min at 37 °C. Cells were then washed with PBS once, passed through a cell strainer (40 µm) to remove clumps and finally analyzed on a BD celesta flow cytometer. Singlets were selected based on forward scatter/side scatter and ploidy was determined depending on the total PI intensity.

### Soft agar colony formation assay

RPE-1 wild type, RPE p53^−/−^, RPE BLM^−/−^, RPE p53^−/−^ BLM^−/−^, RPE MYC p53^−/−^ and RPE MYC p53^−/−^ BLM^−/−^ cells were grown for 5 generations. Alternatively, diploid and aneuploid/polyploid cells were sorted separately from cultures of either RPE p53^−/−^, RPE MYC p53^−/−^ or RPE MYC p53^−/−^ BLM^−/−^ cells. All cells were subjected to a soft agar colony formation assay, as described previously (Borowicz et al, 2014). Briefly, 2x DMEM (with 10% FBS, 1% penicillin-streptomycin-glutamine) medium was mixed with the same volume of sterile 1% agarose (at 42 °C) and layered into each well of a 6-well dish (1.5 ml per well). After this agar layer had solidified 10,000 cells, suspended in 750 µl of 2x DMEM (with 10% FBS, 1% penicillin-streptomycin-glutamine) were mixed with the same volume 0.6% agarose (at 42 °C) and layered on top of the solidified agar (1.5 ml per well). After this upper agar layer had solidified, 500 µl of 1X DMEM was added to each well, which was exchanged for fresh medium every 3 days. After 4 weeks of incubation at 37 °C, colonies were stained using nitroblue tetrazolium chloride solution (Sigma-Aldrich; Cat# T4375) (1 mg/ml in 1x PBS) overnight at 37 °C. The number of colonies was counted, and the fold change was calculated relative to the average number of colonies in the wells containing RPE-1 wild-type cells. During imaging, samples were randomized, and the investigators were blinded to their identity.

### Quantification and statistical analysis

All experiments, if not indicated otherwise in the figure legend, were performed three times and representative experiments are depicted. No statistical methods or criteria were used to pre-determine sample size or to include/exclude samples. Other than the soft agar colony formation assays, all experiments were non-randomized, and the investigators were not blinded to allocation during experiments and outcome assessment. Statistics were performed either with Prism or Microsoft Excel using either a t-test or a Mann–Whitney test. Depending on the variance (calculated by F test), a type 1, 2 or 3 t-test was performed. Statistical details for each experiment including sample size, significance values (*P = 0.05; **P = 0.01; ***P = 0.001; ****P = 0.0001) and tests performed are indicated in the figure legends and figures. Error bars represent standard deviation.

All materials and reagents generated during this study will be provided by the corresponding authors upon request.

## Supplementary information

Appendix

Peer Review File

Movie EV1

Movie EV2

Movie EV3

Source data Fig. 1

Source data Fig. 2

Source data Fig. 3

Source data Fig. 4

Source data Fig. 5

Source data Fig. 6

EV Figure Source Data

Appendix Figure Source data

Expanded View Figures

## Data Availability

This study does not report any original code. No data has been deposited. Any additional information required to re-analyze the data reported in this paper is available from the corresponding authors upon request. The source data of this paper are collected in the following database record: biostudies:S-SCDT-10_1038-S44318-025-00453-w.
