# Supplementary material for: Mechanistic insight into anaphase bridge signaling to the abscission checkpoint
Source: EMBO J. 2025 May 12;44(13):3824–52. doi: 10.1038/s44318-025-00453-w (PMC12217976; doi:10.1038/s44318-025-00453-w)
Supplement: Supplementary file 1 — Appendix [file 44318_2025_453_MOESM1_ESM.pdf]

Appendix for

MECHANISTIC INSIGHT INTO ANAPHASE BRIDGE SIGNALING TO THE ABSCISSION CHECKPOINT

Manika I. Singh[1,2], Girish Rajendraprasad[3], Vasileios Katopodis [3], Rui Cui[1], Marin Barisic[3,4], Rahul Bhowmick[5]* and Ian D. Hickson[1]*

Table of Contents:

Appendix Figure S1	Pages 2-3
Appendix Figure S2	Pages 4-5
Appendix Figure S3	Pages 6-7
Appendix Table S1	Pages 8-9
Appendix Table S2	Page 10
References	Page 11

Appendix Figure S1

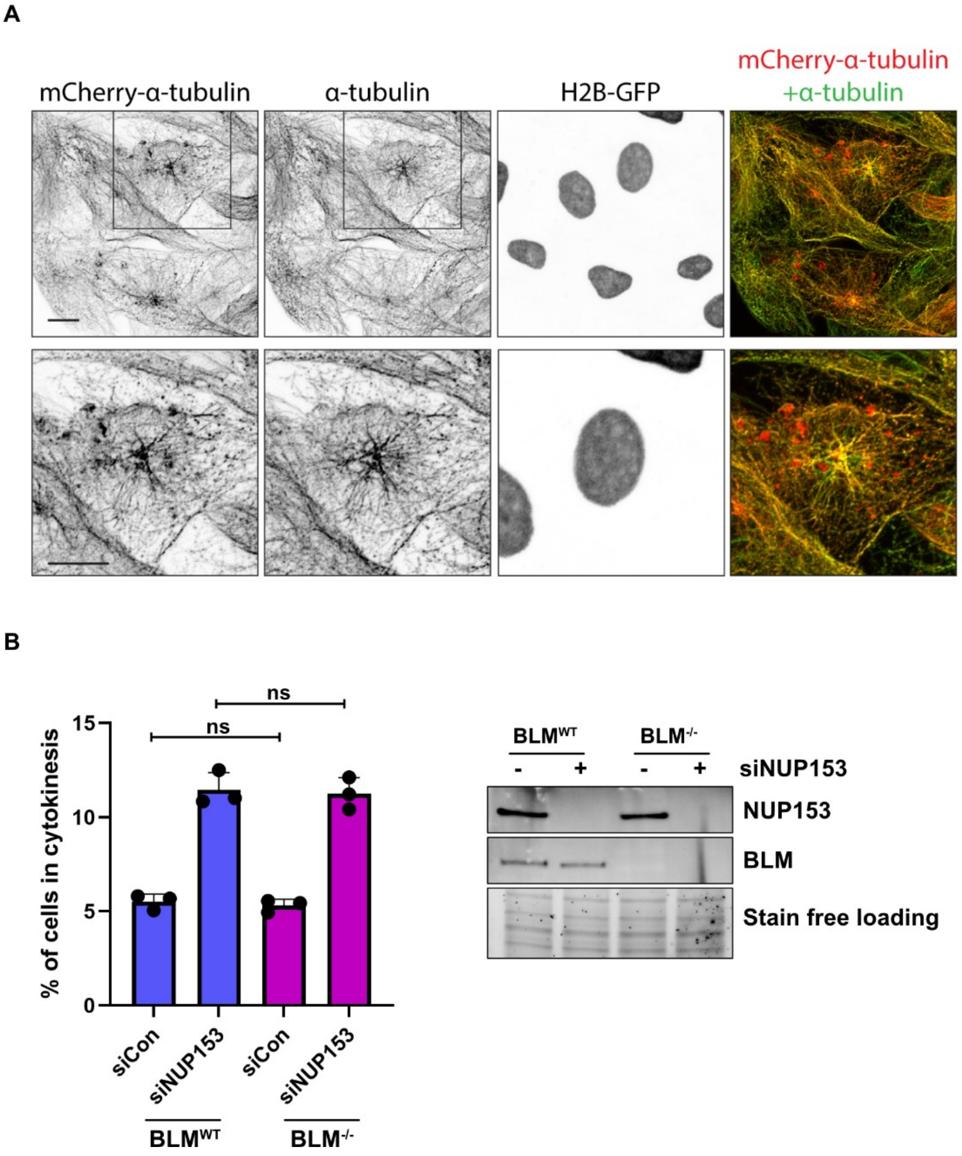

Appendix Figure S1. BLM influences UFB induced abscission delay independent of nucleoporin-triggered cytokinesis regulation.

(A) Representative immunofluorescence image showing that the atypical aggregates visible in live cell imaging of U2OS H2B-GFP mCherry-tubulin cells are mCherry aggregates, not tubulin. The aggregates visible with mCherry-tubulin are not visible in the anti-tubulin channel. The square box in the top panel is zoomed in the lower panel. Scale bar, 10 µm.

(B) Quantification (left) of cytokinetic cells in asynchronous population with indicated cell lines, after 48 hours of siRNA depletion. The data are an average of three independent biological

replicates with error bars representing the standard deviation. A Mann-Whitney test was performed to derive significance. Exact *p* values are 0.400 (ns) between siCon of BLM^{WT} and $BLM^{-/-}$ and >0.99 (n.s.) between siNUP153 of BLM^{WT} and $BLM^{-/-}$. Representative western blot (right) of the whole cell lysate from BLM^{WT} and $BLM^{-/-}$ cells, showing the depletion of NUP153.

Appendix Figure S2

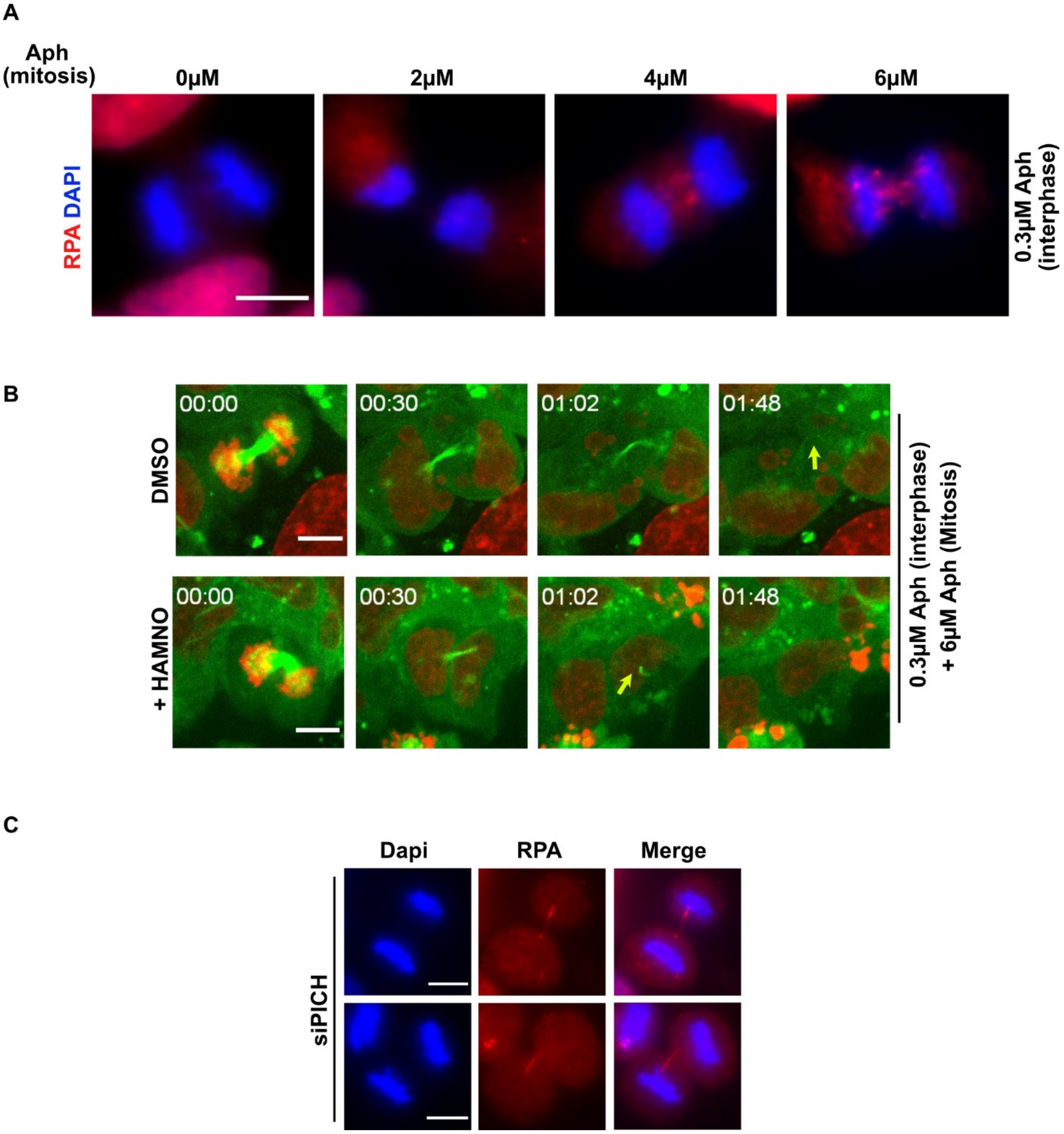

Appendix Figure S2. Generation of RPA-coated UFBs is sufficient to induce abscission delay.

(A) Representative immunofluorescence images of BLM^{WT} cells in anaphase exposed to RS (0.3 μM Aph) in the preceding interphase and then treated with high dose Aph (2μM, 4μM, 6μM) exclusively during mitosis. Scale bar, 10 μm.
(B) Representative still pictures of live-cell imaging of U2OS cells (with no visible chromatin bridges) stably expressing fluorescently tagged histone H2B (green) and α-tubulin (red) transfected with siBLM and exposed to RS (0.3 μM Aph) during the preceding interphase, and then treated with high dose Aph (6 μM) in the presence or absence of an RPA inhibitor (50 μM, HAMNO) exclusively during mitosis. The yellow arrows indicate the site of abscission. Scale bar, 10 μm.
(C) Representative immunofluorescence images of U2OS cells treated with siPICH and stained with anti-RPA antibody and DAPI after fixing cells at anaphase. Scale bar, 10 μm.

Appendix Figure S3

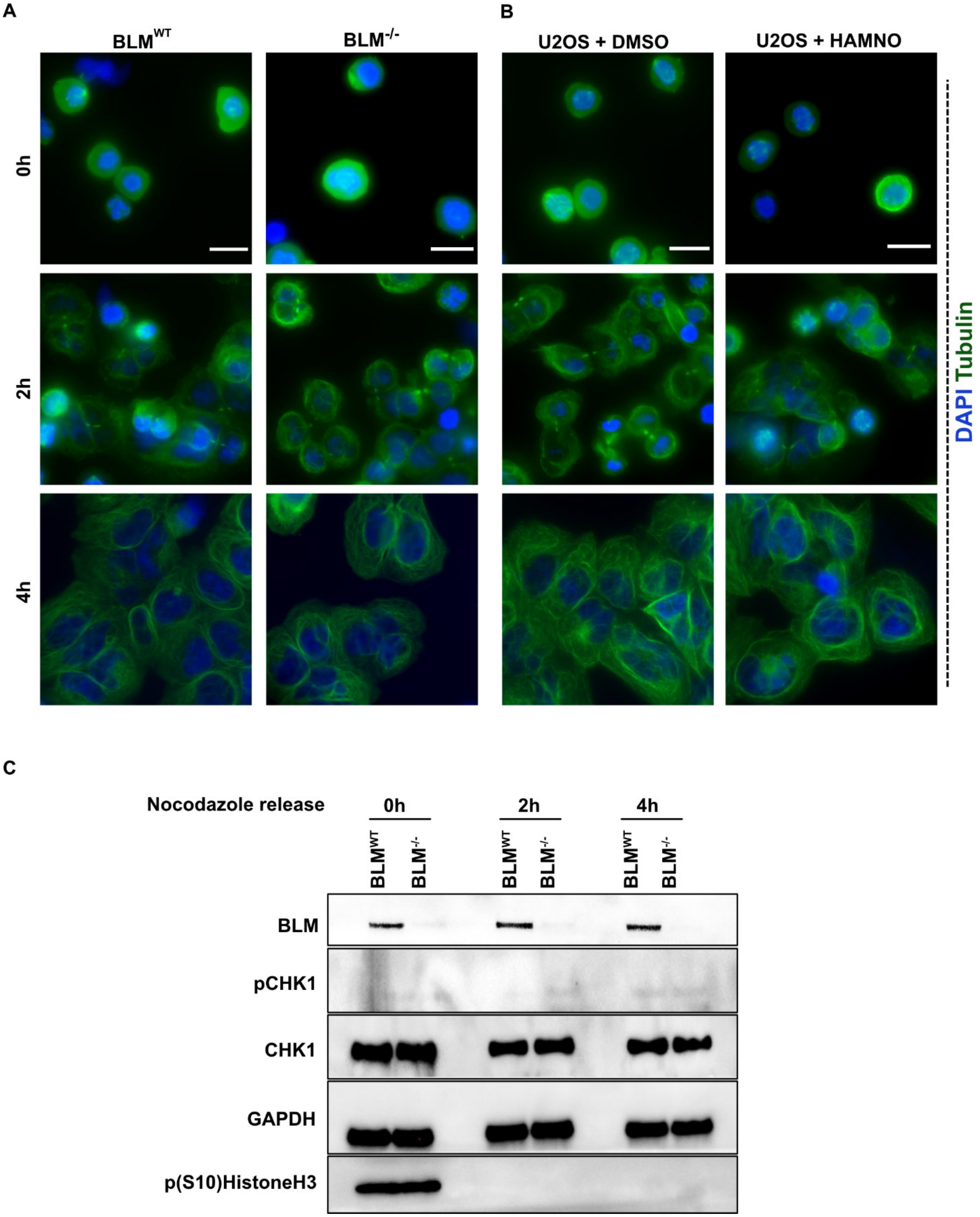

Appendix Figure S3. RPA signals to Aurora B through ChK1 in presence of RS but not in unperturbed cells.

(A-B) Representative immunofluorescence images of BLM^{WT}, $BLM^{-/-}$ and U2OS cells with or without an RPA inhibitor (50 µM, HAMNO) after the treatments indicated in Figures 4A and 4C, respectively. Scale bar, 20 µm.

(C) Representative western blots with the indicated antibodies of whole cell lysates derived from BLM^{WT} and $BLM^{-/-}$ U2OS cells, arrested with nocodazole and released for the indicated time, in absence of RS.

Appendix Table S1. List of oligos used in the study.

Oligo name	Sequence (5` - 3`)
BLM-gDNA2_For	CACCGGATGCTGACAAACAAGAAAG
BLM-gDNA2_Rev	AAACCTTTCTTGTTTGTCAGCATCC
BLM-3'Arm_For	GTTATTAGGTCCCTCGAAGAGGTTCACTAGTCTTGTTTGT<br>CAGCATCTGACCATC
BLM-3'Arm_Rev	AGTCACGACGTTGTAAAACGACGGCCAGTGGTCCACCCA<br>AACAATCTGCCAATCTTTC
BLM-5'Arm_For	ACTCTAGAGGATCCCCGGGTACCGAGCTCGTTGGTAATC<br>GGCTCTGGCGGCTTCT
BLM-5'Arm_Rev	TGAGAATGCATATGAAGGCTTAAGAAACGGTCTATTTAT<br>AG
BLM-EXT_For	CTGCGGGTAATGCTGCCCACAG
BLM-EXT_Rev2	GTGACCTACAGCTGAGAAACGCATGTC
BLM-Linker-mAID-3UTR	CCGTTTCTTAAGCCTTCATATGCATTCTCAGGATCCGGTT<br>CCGACTACAAGGACGATGATGACAAGGGATCCGGTGCAG<br>GCGCCAAGGAGAAGAGTGCTTGTCCTAAAGATCCAGCCA<br>AACCTCCGGCCAAGGCACAAGTTGTGGGATGGCCACCGG<br>TGAGATCATACCGGAAGAACGTGATGGTTTCCTGCCAAA<br>AATCAAGCGGTGGCCCGGAGGCGGCGGCGTTCGTGAAGG<br>TATCAATGGACGGAGCACCGTACTTGAGGAAAATCGATT<br>TGAGGATGTATAAATAACAATCGAATCTCAATGTACATA<br>GACCTTCTTTCTTGTTTGTCAGCATCTGACCATCTGTGACT<br>ATAAAGCTGTTATTCTTGTTATACCATTTGAAGTTTTTACT<br>CGTCTCTATTAATATTTAAATAAATGCTGGGGGGTGATAG<br>TTCTTCTTTTTAAAATAAACATTTTCTTTTGAATAAGCATG<br>TTTTGCTGCCGCTGCAAGTGTTGTGGCCGTTGTTTCTCAG<br>AACGTCTGAGGCAGCAGCTGAATCATCTCAGTGCAAGAG<br>CTTCTGAGCATAACACGAAACCCAGAAGCCAAAGGAAGA<br>GCCACGCGTGGGCCCTTGTGAAACTAAAGCTTTTCGTGTA<br>AGACAACACAAACAAAATTTAAAGACAAATGACGGGGA<br>AAAGAGGAGAAAATATATTACAAAGGATTAGTATCCATC<br>ATACCAAATACCCGTGAACCAGTCAGAAACATCCCAGGG<br>GGCAGGTGGACCAAGGATGTGAACAGGCTAGTCTCAGAA<br>GAAGAAATACACATGCTCATGGCCCGGCACTGTGGCTCA<br>CGCCTGGGATCCCAGCACTTTGGGAGGCCGAGGCAGGTG<br>GATCACGAGGTCAGGAGTTTGAGACCAGCCTGCCCAACA<br>TGGTGAAACCCCGTCTCTACTAAAAATACAAAAATTAGC<br>CAGGCGTGGTGTACAGGCACGCCTGTAGTCCCAGCTACT<br>CAGGAGGCTGAGGCAAGAGAATCGCTTGAACCCAGGAG<br>GCGGAGGTTGCAGTGAGCCGAGATCGTGCCACTGCACTC<br>CAGCCTGGGTGACAGAGCAAGACTCCGTCTCAAAAAAAA<br>AAAAAAAATAACAAATATACATGCTCTGCAAATATGTGA<br>AAAAGGTCAATCTCCATGAATAAAAATATGATAAAACCA<br>GTTGTTGTTAACTTGTTTATTGCAGCTTAT

BLMKO_For (oligo for BLM guide RNA)	CACCGGGGACTGTTTACTGACTAC
BLMKO_Rev (oligo for BLM guide RNA)	AAACGTAGTCAGTAAACAGTCCCC
P53KO_For (oligo for p53 guide RNA)	CACCCAGAATGCAAGAAGCCCAGA
P53KO_Rev (oligo for p53 guide RNA)	AAACTCTGGGCTTCTTGCATTCTG
Kpn1-TUB-F	GGTACCATGCGTGAGTGCATCTCCATCCAC
BamH1-TUB-R	GGATCCTTAGTATTCCTCTCCTTCTTCCTC

Appendix Table S2. List of antibodies used in the study.

Antibody	Company; Catalogue number
Rabbit anti Nup153	Abcam; Cat# ab84872
Rabbit anti Tubulin	Abcam; Cat# ab18251
Mouse anti Tubulin	Sigma Aldrich; Cat# T5168
Guinea pig anti PICH	In house; Nielsen et al., 2015
Mouse anti RPA	Abcam; Cat# ab2175
Rabbit anti BLM	Abcam; Cat# ab2179
Rabbit monoclonal anti-CHK1-S345P	Cell signaling; Cat# 2348
Chk1 (2G1D5) Mouse	Cell signaling; Cat# 2360
Rabbit anti-GAPDH	Sigma; Cat# PLA0125
Rabbit anti phospho-Histone H3 (Ser10)	Millipore; Cat# 06-570
Anti-ATR	Abcam; Cat# ab2905
Recombinant Anti-ATRIP antibody	Abcam; Cat# ab175221
Goat anti RACGAP1	Abcam; Cat# Ab2270
Rabbit anti-Aurora B pT232	Rockland Immunochemicals; Cat# 600-401-677
Rabbit Anti-Phospho CHMP4C	Cancertools; Cat# 151827
Anti-Myc/c-Myc	Santa Cruz Biotechnology, Cat# sc-40
Anti-p53	Santa Cruz Biotechnology, Cat# sc-126
Goat anti-Mouse IgG (H+L)–Peroxidase	Sigma Aldrich; Cat# A4416
Goat anti-Rabbit IgG (H+L)–Peroxidase	Sigma Aldrich; Cat# A6667
Goat anti-Goat IgG (H+L)–Peroxidase	Sigma Aldrich; Cat# SA5-10314
Alexa Fluor 488 goat anti-rabbit IgG	Thermo Fisher Scientific; Cat# A-11008
Alexa Fluor 568 goat anti-rabbit IgG	Thermo Fisher Scientific; Cat# A-11011
Alexa Fluor 488 goat anti-mouse IgG	Thermo Fisher Scientific; Cat# A-11001
Alexa Fluor 568 donkey anti-mouse IgG	Thermo Fisher Scientific; Cat# A-10037
Alexa Fluor 568 goat anti- guinea pig IgG	Thermo Fisher Scientific; Cat# A-11075
Alexa Fluor 488 goat anti- guinea pig IgG	Thermo Fisher Scientific; Cat# A-11073

References:

Nielsen CF, Huttner D, Bizard AH, Hirano S, Li T-N, Palmai-Pallag T, Bjerregaard VA, Liu Y, Nigg EA, Wang LH-C *et al* (2015) PICH promotes sister chromatid disjunction and co-operates with topoisomerase II in mitosis. *Nature Communications* 6: 8962
