# Supplementary material for: Mechanistic insight into anaphase bridge signaling to the abscission checkpoint
Source: EMBO J. 2025 May 12;44(13):3824–52. doi: 10.1038/s44318-025-00453-w (PMC12217976; doi:10.1038/s44318-025-00453-w)
Supplement: Supplementary file 3 — Movie EV1 [file 44318_2025_453_MOESM3_ESM.zip › Movie EV1 Legend.docx]

Movie EV1: BLM-depleted cells fail to undergo any abscission delay. Related to Figure 1A and 1B.

Representative movie of fluorescently tagged histone H2B (green) and α-tubulin (red) U2OS cells going through cytokinesis after treatment with siCon (left) or siBLM (right) for 48 hours.
