# Supplementary material for: Mechanistic insight into anaphase bridge signaling to the abscission checkpoint
Source: EMBO J. 2025 May 12;44(13):3824–52. doi: 10.1038/s44318-025-00453-w (PMC12217976; doi:10.1038/s44318-025-00453-w)
Supplement: Supplementary file 4 — Movie EV2 [file 44318_2025_453_MOESM4_ESM.zip › Movie EV2 Legend.docx]

Movie EV2: BLM depleted cells, unlike control cells, fail to undergo any abscission delay following replication stress. Related to Figure 1C and 1D.

Representative movie of fluorescently tagged histone H2B (green) and α-tubulin (red) U2OS cells going through cytokinesis after treatment with siCon (left) or siBLM (right) for 48 hours and in the presence of replication stress during interphase.
