# Supplementary material for: Mechanistic insight into anaphase bridge signaling to the abscission checkpoint
Source: EMBO J. 2025 May 12;44(13):3824–52. doi: 10.1038/s44318-025-00453-w (PMC12217976; doi:10.1038/s44318-025-00453-w)
Supplement: Supplementary file 5 — Movie EV3 [file 44318_2025_453_MOESM5_ESM.zip › Movie EV3 Legend.docx]

Movie EV3: Inhibition of RPA fails to induce any abscission delay upon replication stress. Related to Figure 2E and 2F.

Representative movie of fluorescently tagged histone H2B (green) and α-tubulin (red) U2OS cells going through cytokinesis after treatment with DMSO (left) or RPAi (right) during mitosis and in the presence of replication stress during interphase.
