# Supplementary figures and images for: Mechanistic insight into anaphase bridge signaling to the abscission checkpoint (part 1 of 2)
Source: EMBO J. 2025 May 12;44(13):3824–52. doi: 10.1038/s44318-025-00453-w (PMC12217976; doi:10.1038/s44318-025-00453-w)

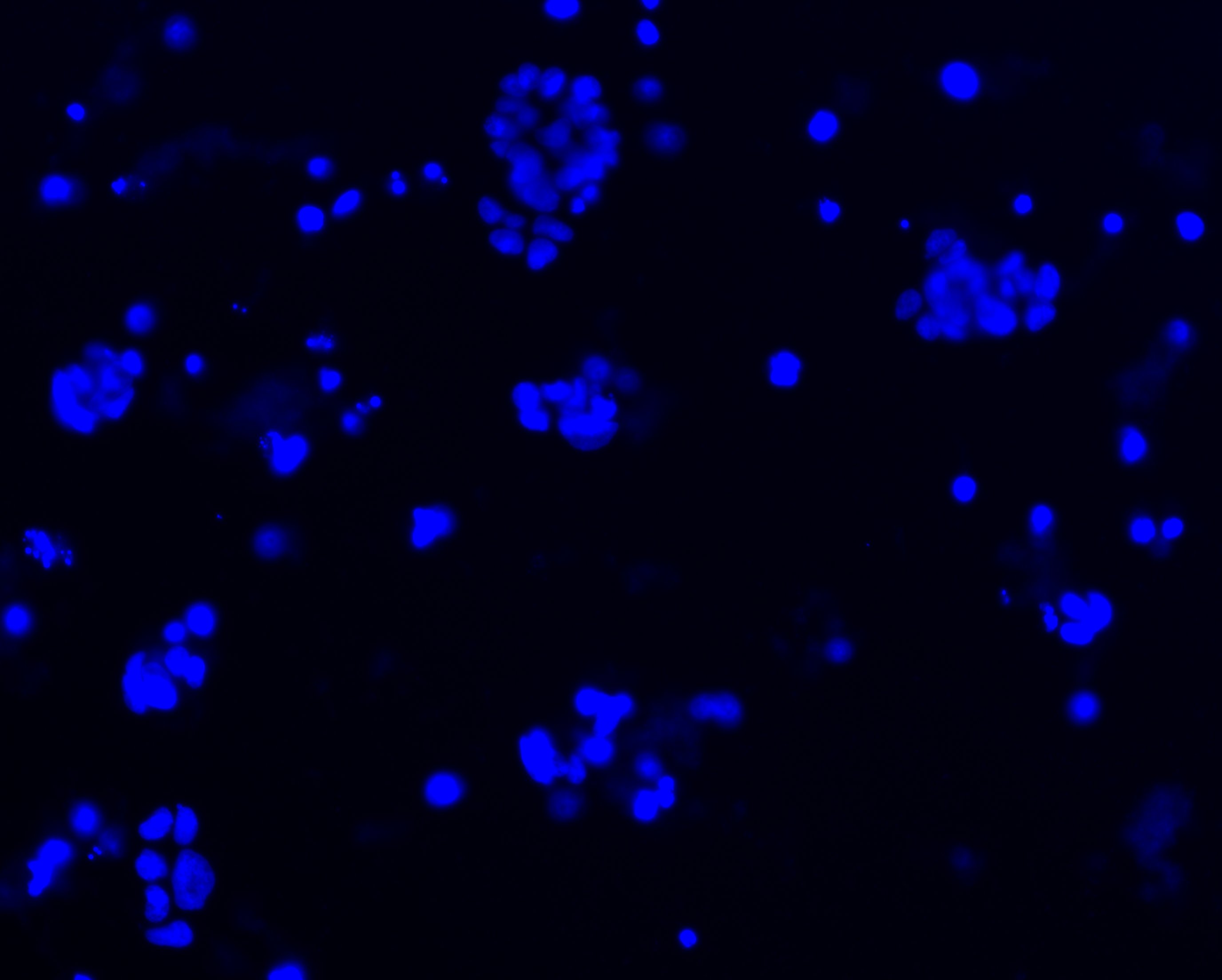

Supplement: Supplementary file 6 — Source data Fig. 1 [file 44318_2025_453_MOESM6_ESM.zip › Figure 1/1H/Raw file +iaa +aph.jpg]

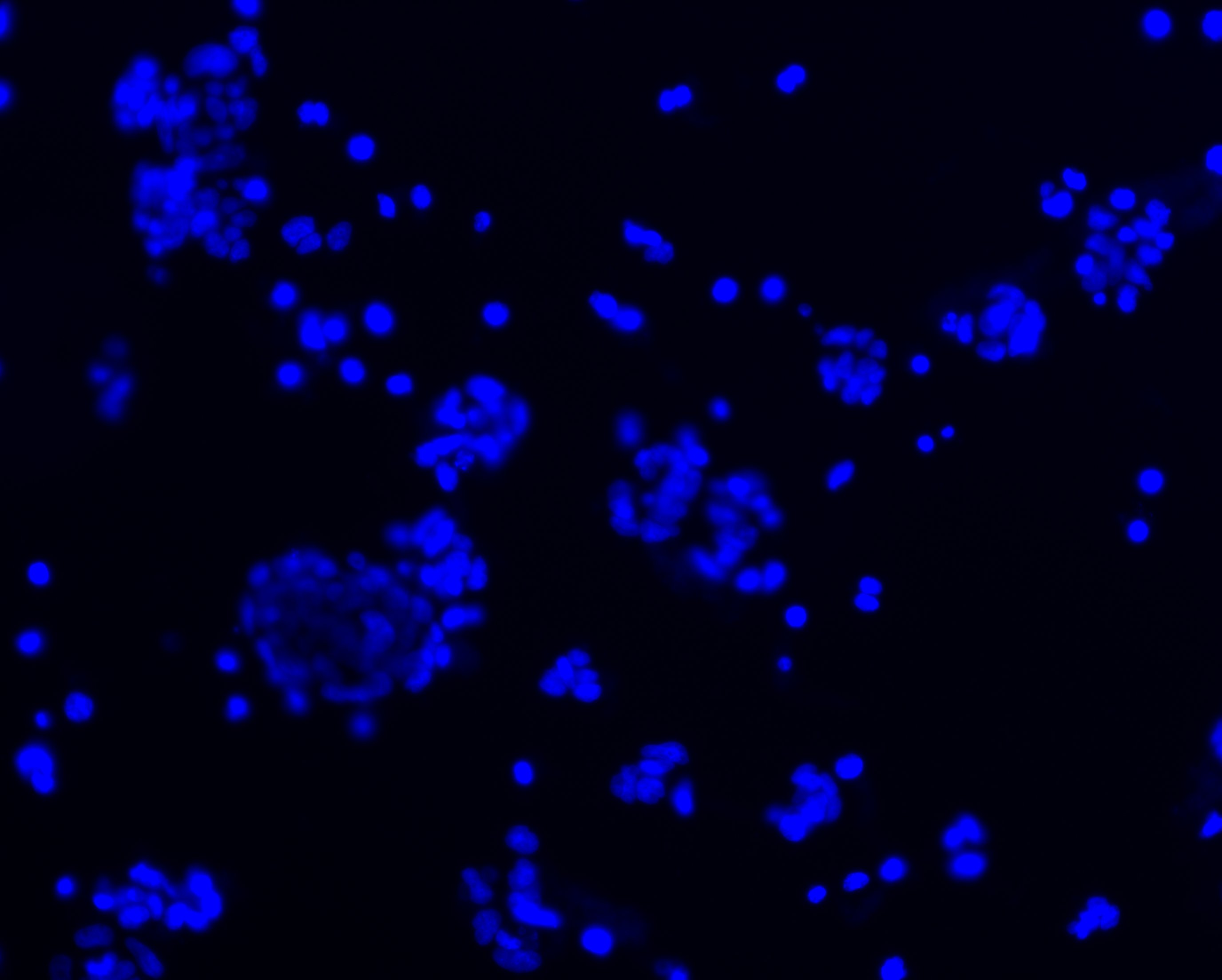

Supplement: Supplementary file 6 — Source data Fig. 1 [file 44318_2025_453_MOESM6_ESM.zip › Figure 1/1H/Raw file -iaa -aph.jpg]

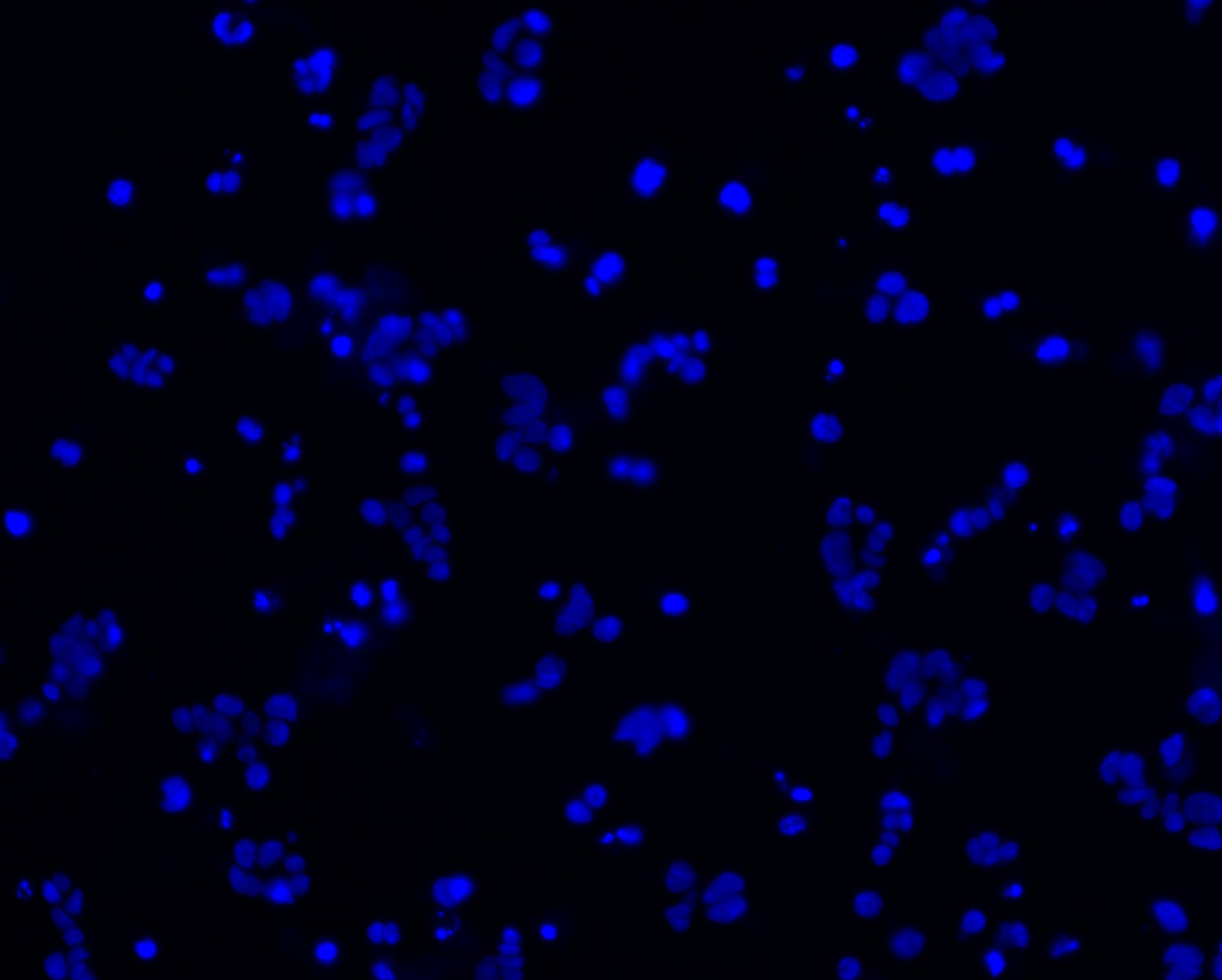

Supplement: Supplementary file 6 — Source data Fig. 1 [file 44318_2025_453_MOESM6_ESM.zip › Figure 1/1H/Raw file -iaa +aph.jpg]

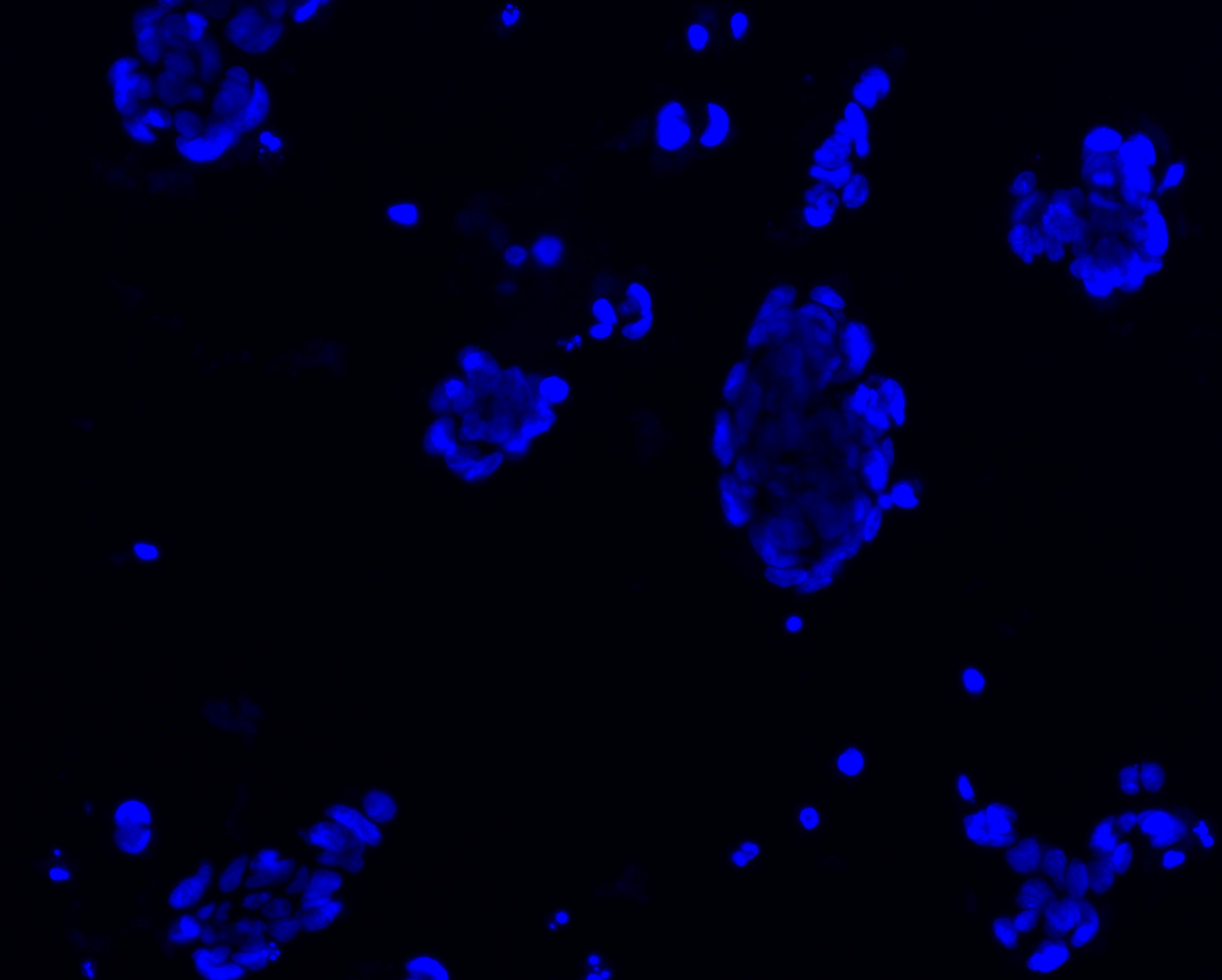

Supplement: Supplementary file 6 — Source data Fig. 1 [file 44318_2025_453_MOESM6_ESM.zip › Figure 1/1H/Raw file +iaa -aph.jpg]

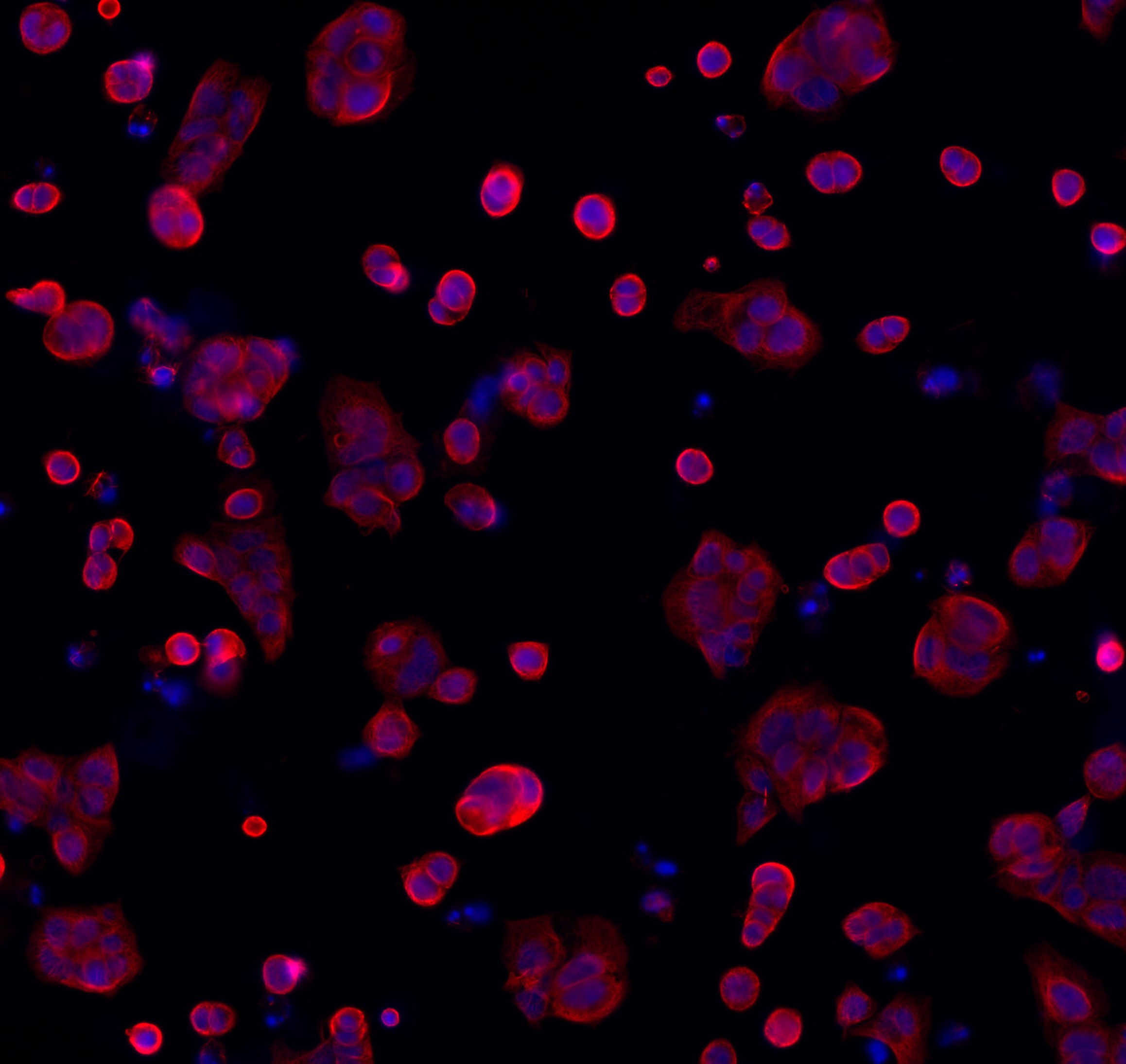

Supplement: Supplementary file 6 — Source data Fig. 1 [file 44318_2025_453_MOESM6_ESM.zip › Figure 1/1G/Raw file -iaa-aph.jpg]

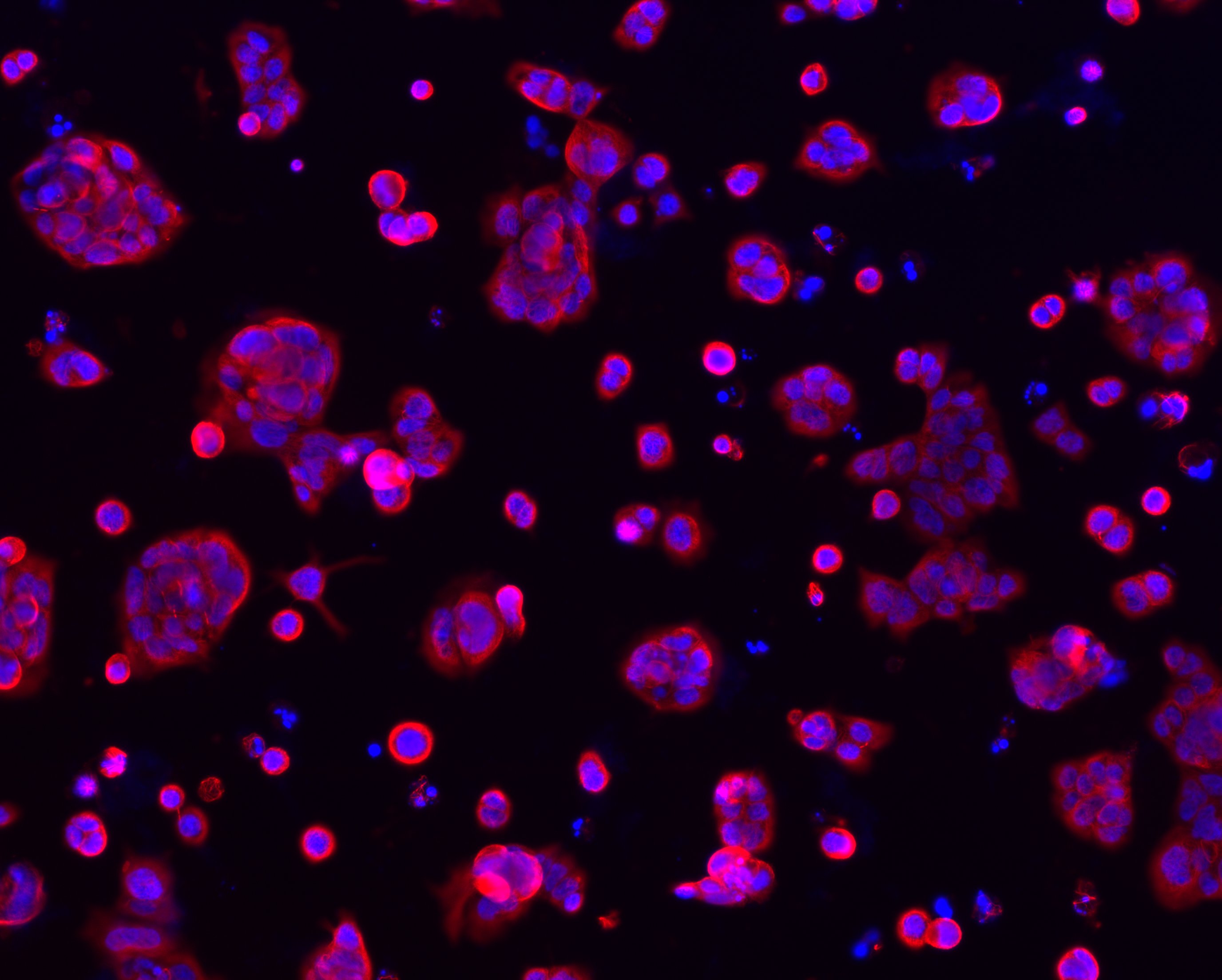

Supplement: Supplementary file 6 — Source data Fig. 1 [file 44318_2025_453_MOESM6_ESM.zip › Figure 1/1G/Raw file +iaa +aph.jpg]

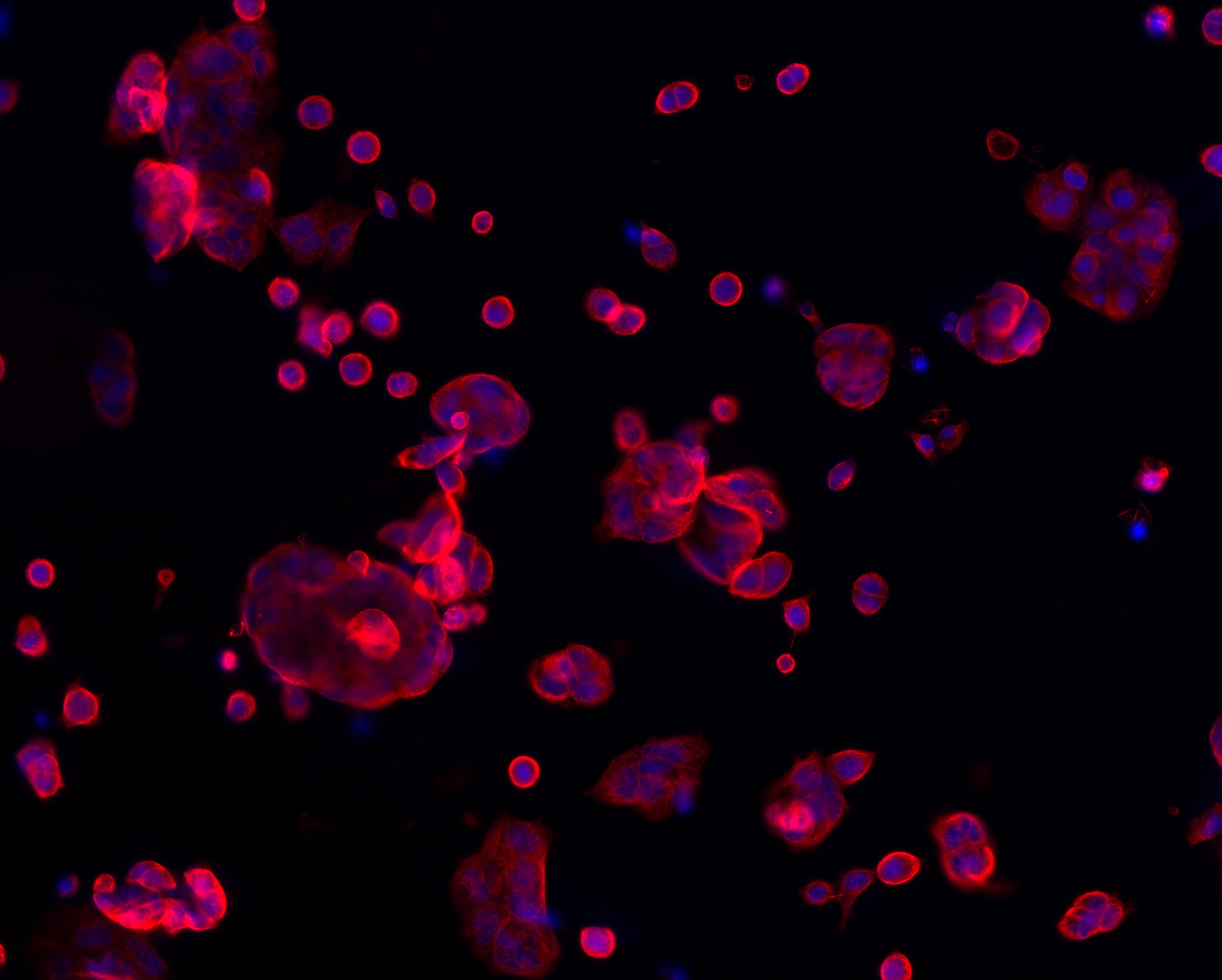

Supplement: Supplementary file 6 — Source data Fig. 1 [file 44318_2025_453_MOESM6_ESM.zip › Figure 1/1G/Raw file -iaa +aph .jpg]

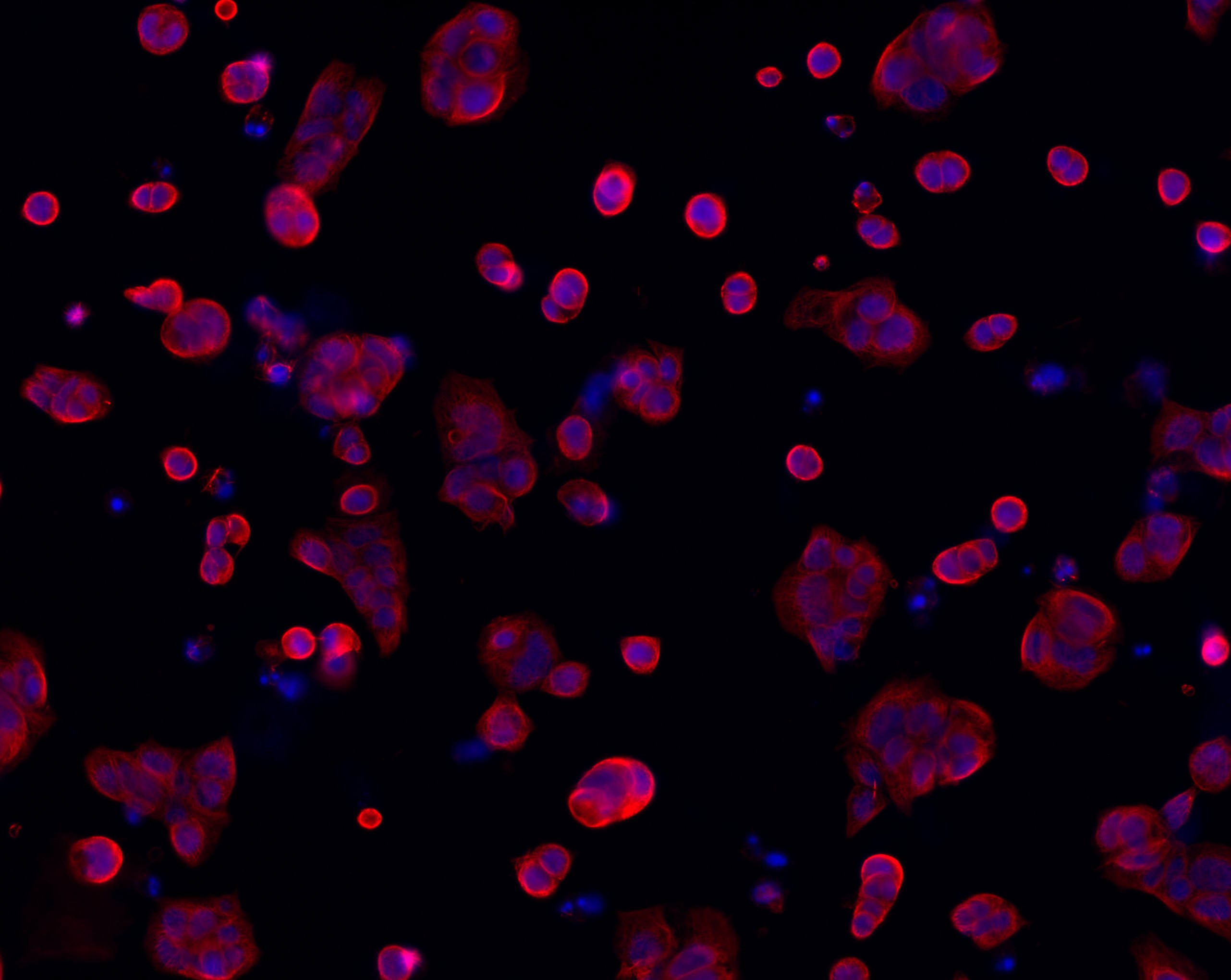

Supplement: Supplementary file 6 — Source data Fig. 1 [file 44318_2025_453_MOESM6_ESM.zip › Figure 1/1G/Raw file +iaa -aph.jpg]

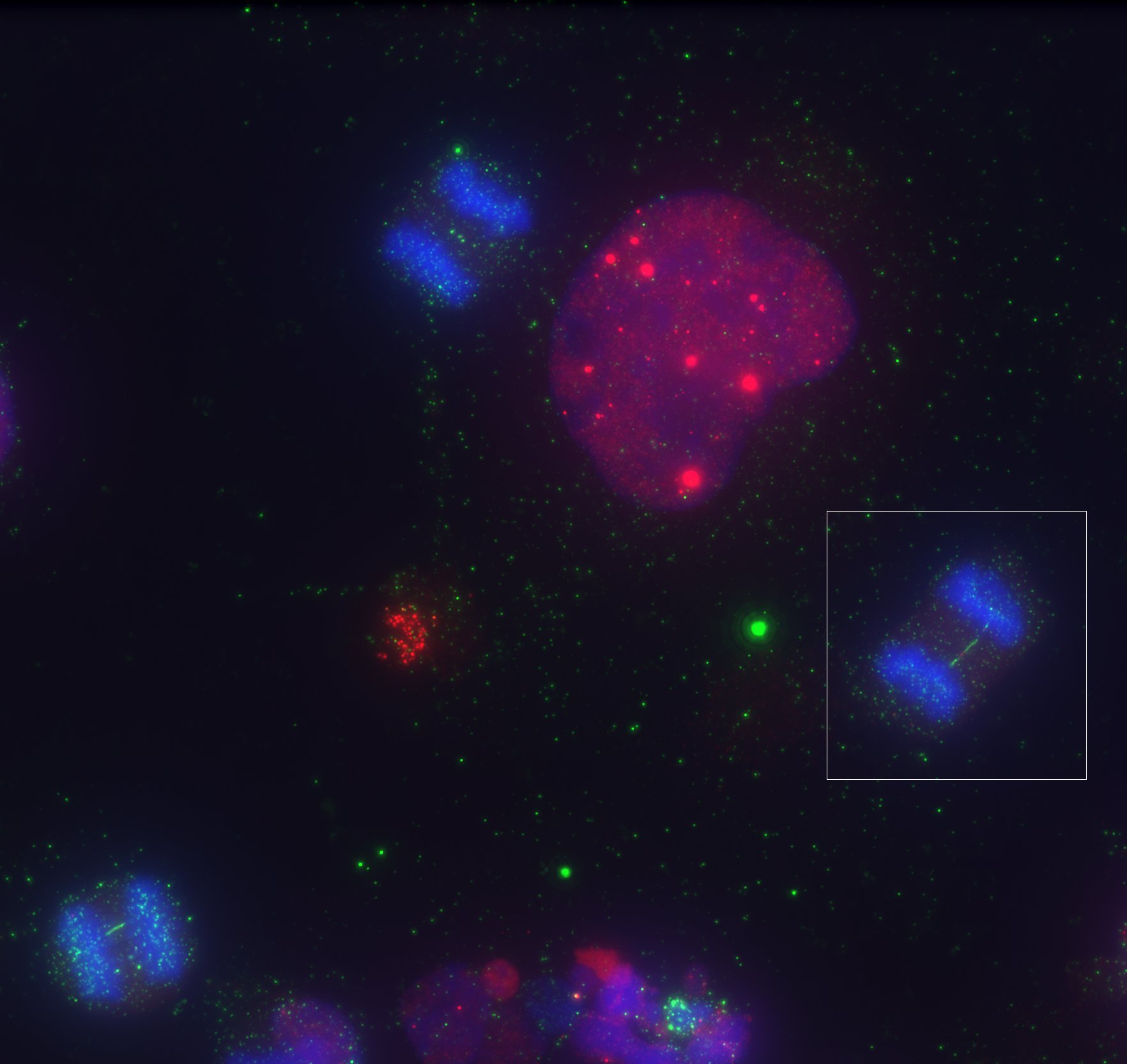

Supplement: Supplementary file 7 — Source data Fig. 2 [file 44318_2025_453_MOESM7_ESM.zip › Figure 2/2A/Raw WT -APH.jpg]

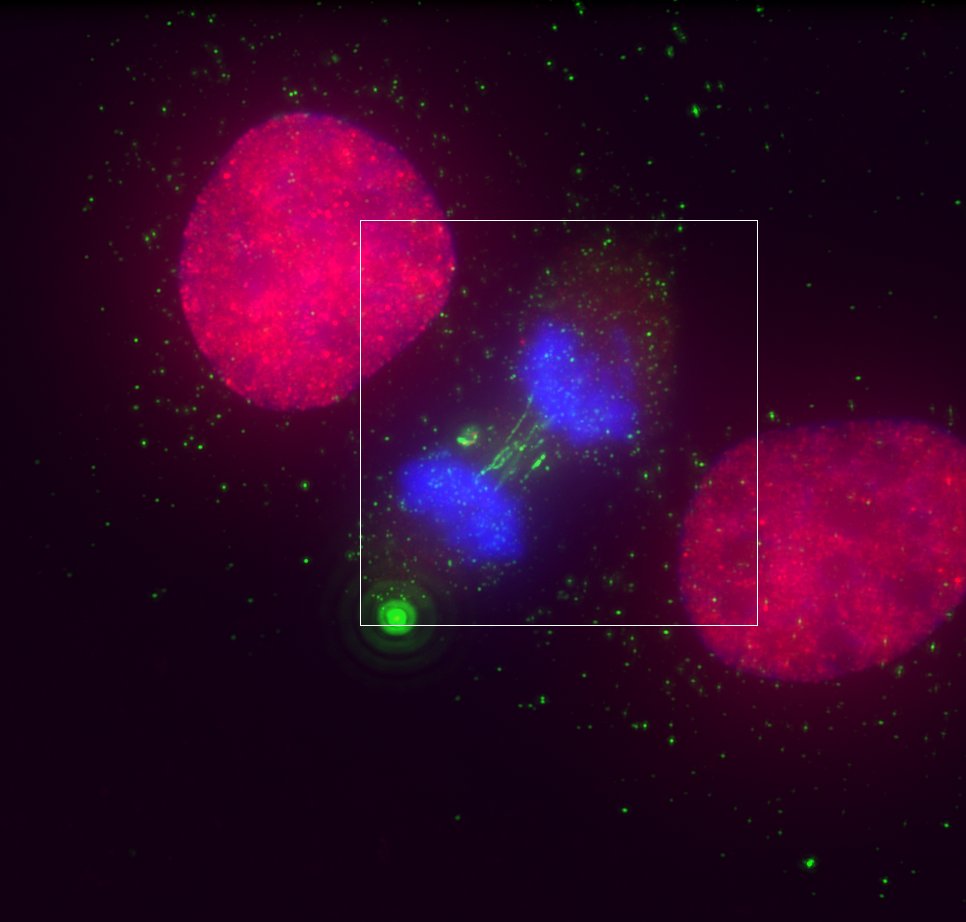

Supplement: Supplementary file 7 — Source data Fig. 2 [file 44318_2025_453_MOESM7_ESM.zip › Figure 2/2A/Raw KO +APH.jpg]

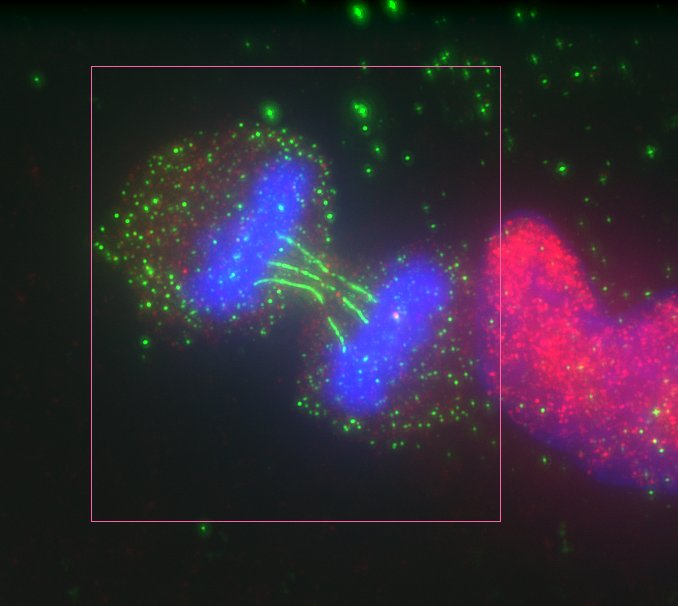

Supplement: Supplementary file 7 — Source data Fig. 2 [file 44318_2025_453_MOESM7_ESM.zip › Figure 2/2A/Raw KO -APH.jpg]

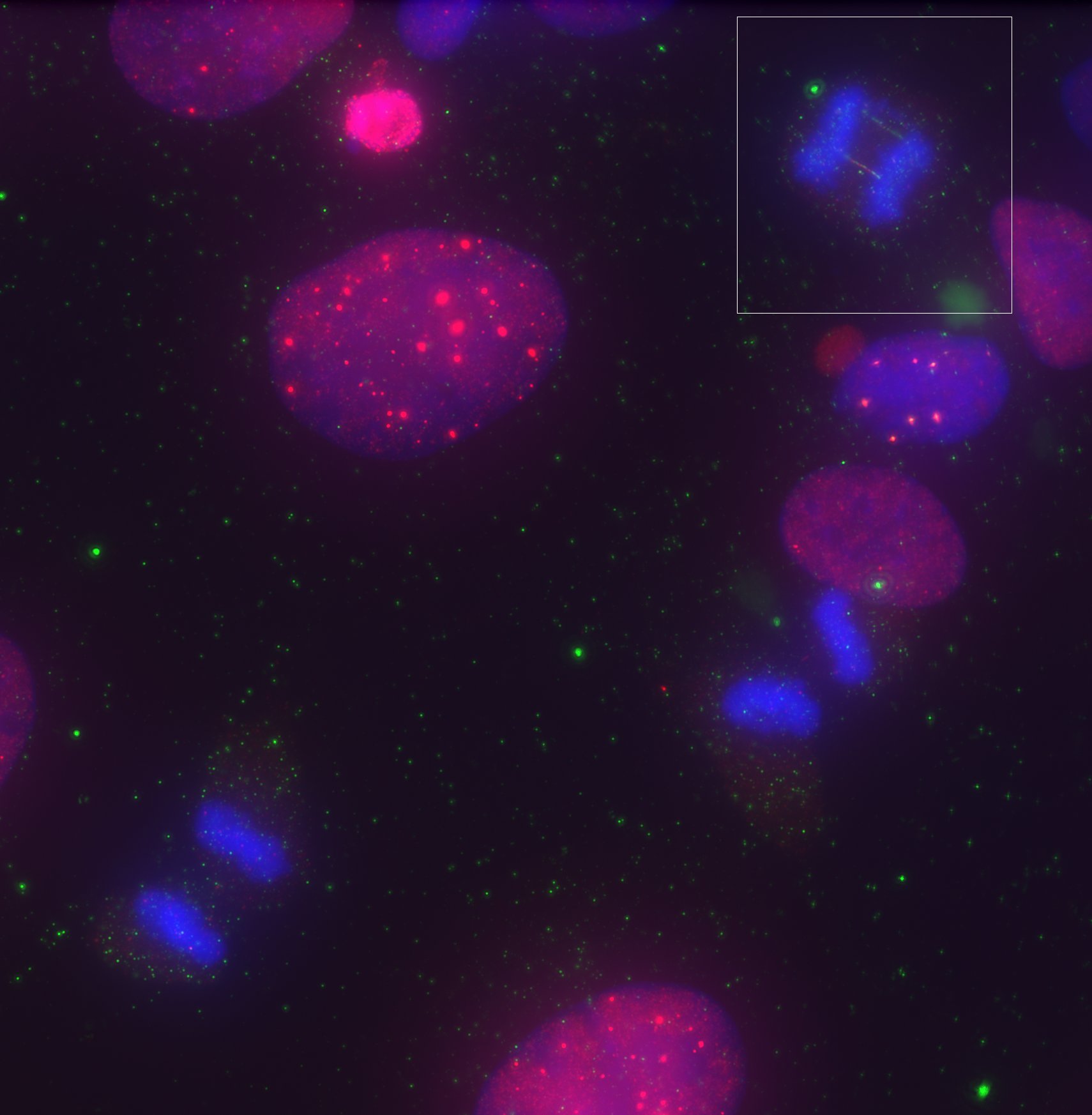

Supplement: Supplementary file 7 — Source data Fig. 2 [file 44318_2025_453_MOESM7_ESM.zip › Figure 2/2A/Raw WT +APH.jpg]

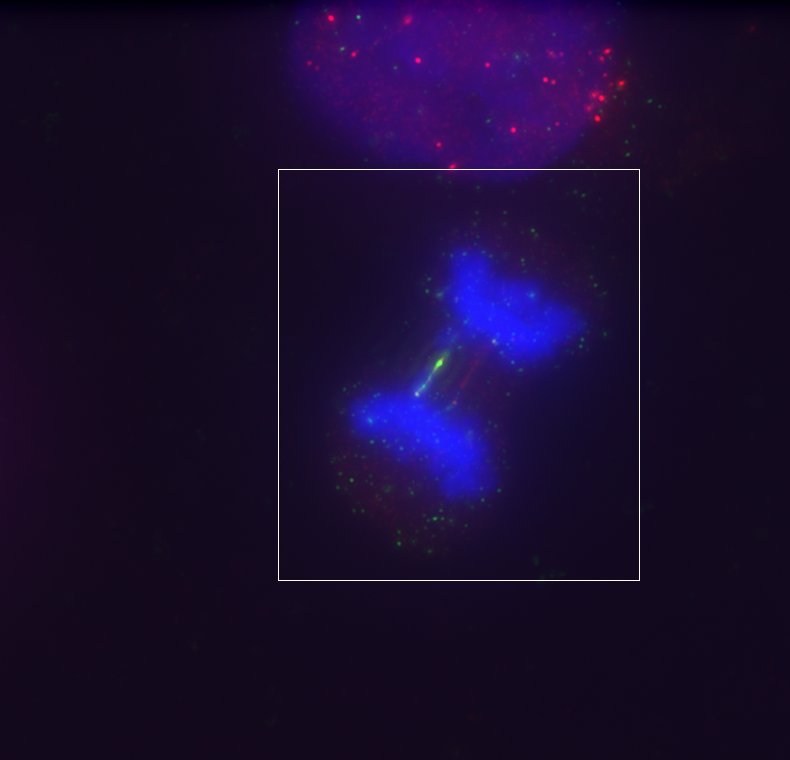

Supplement: Supplementary file 7 — Source data Fig. 2 [file 44318_2025_453_MOESM7_ESM.zip › Figure 2/2C/raw degron -IAA.jpg]

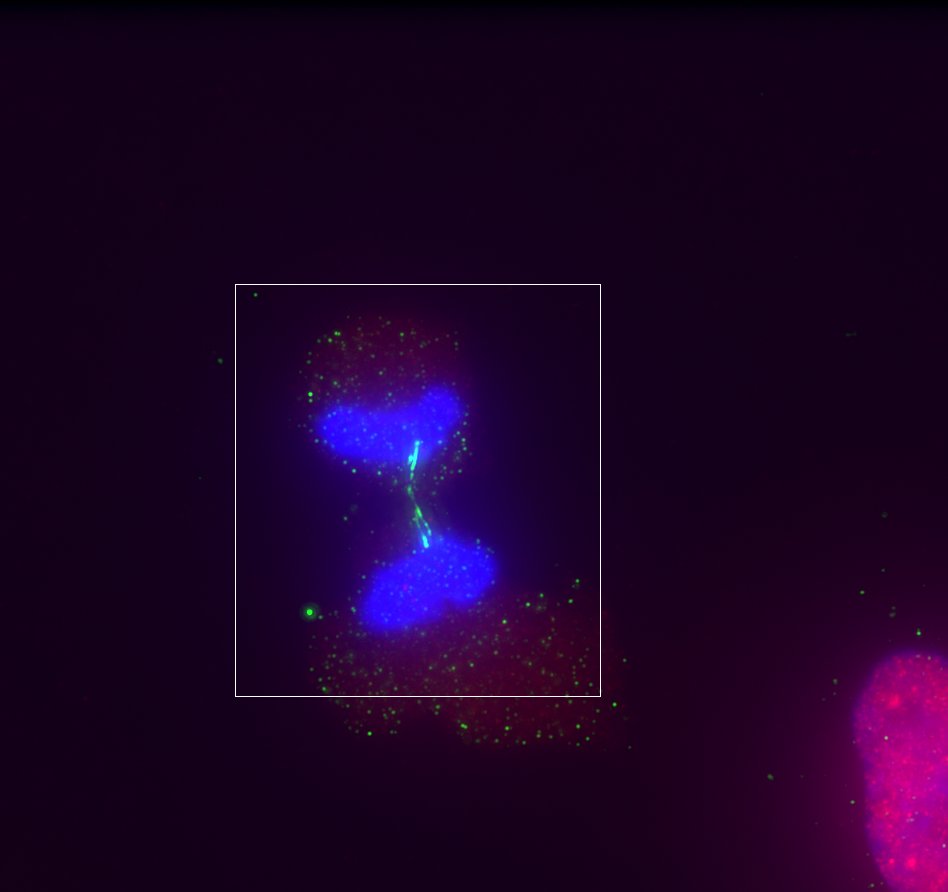

Supplement: Supplementary file 7 — Source data Fig. 2 [file 44318_2025_453_MOESM7_ESM.zip › Figure 2/2C/raw degron +IAA.jpg]

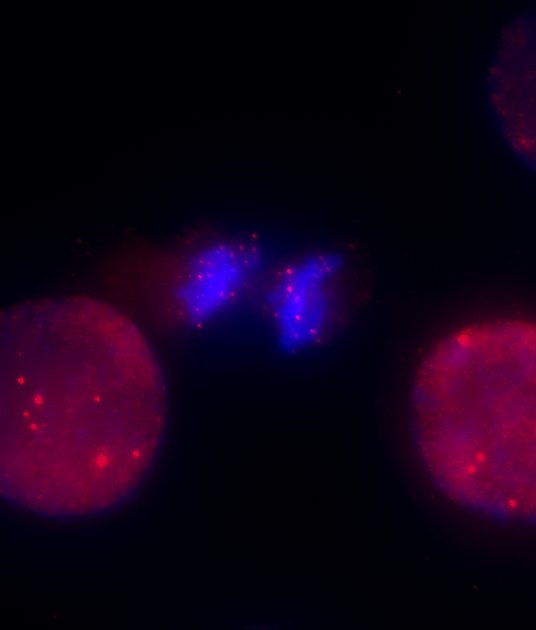

Supplement: Supplementary file 8 — Source data Fig. 3 [file 44318_2025_453_MOESM8_ESM.zip › Figure 3/3B/Raw fig 3B upper panel-8.jpg]

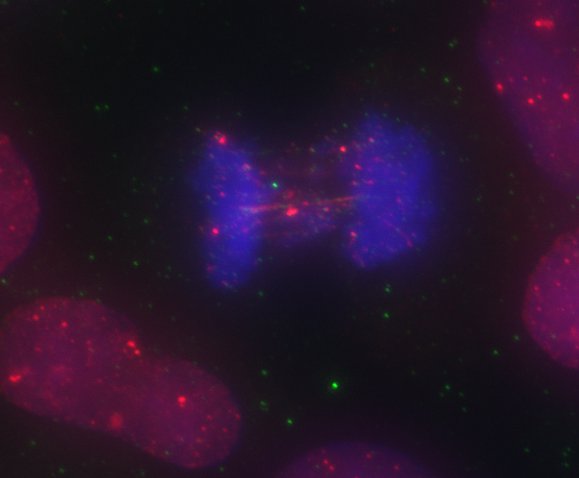

Supplement: Supplementary file 8 — Source data Fig. 3 [file 44318_2025_453_MOESM8_ESM.zip › Figure 3/3B/Raw fig 3B bottom panel.jpg]

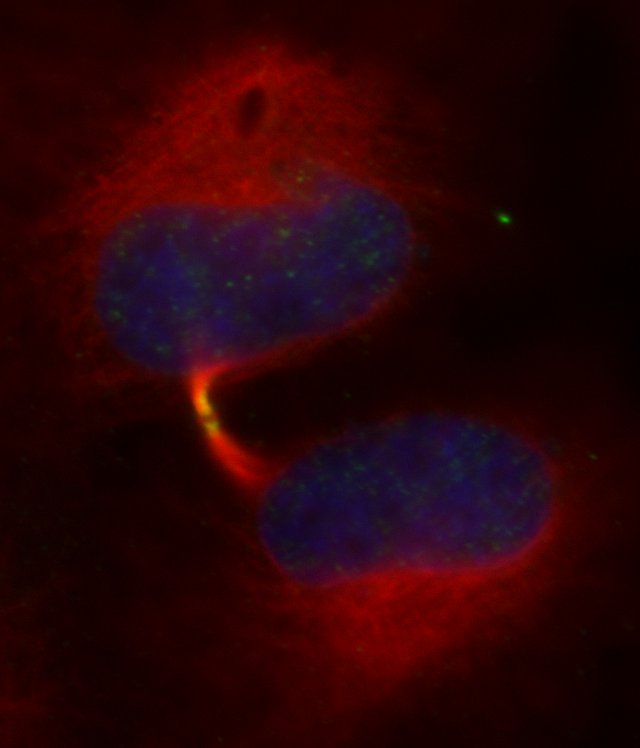

Supplement: Supplementary file 9 — Source data Fig. 4 [file 44318_2025_453_MOESM9_ESM.zip › Figure 4/4E/wt Raw.jpg]

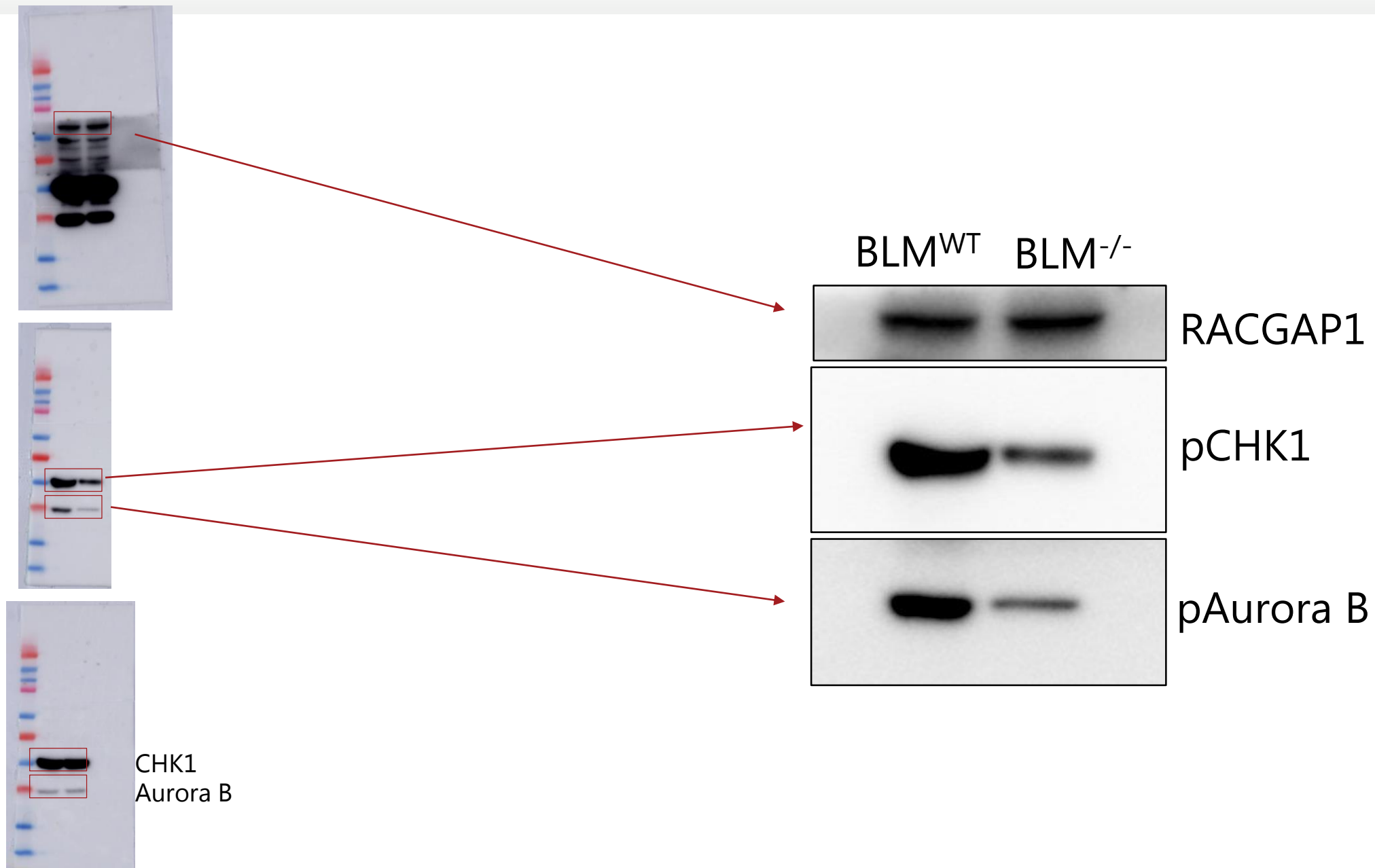

Supplement: Supplementary file 9 — Source data Fig. 4 [file 44318_2025_453_MOESM9_ESM.zip › Figure 4/4G/Figure 4G.pdf]

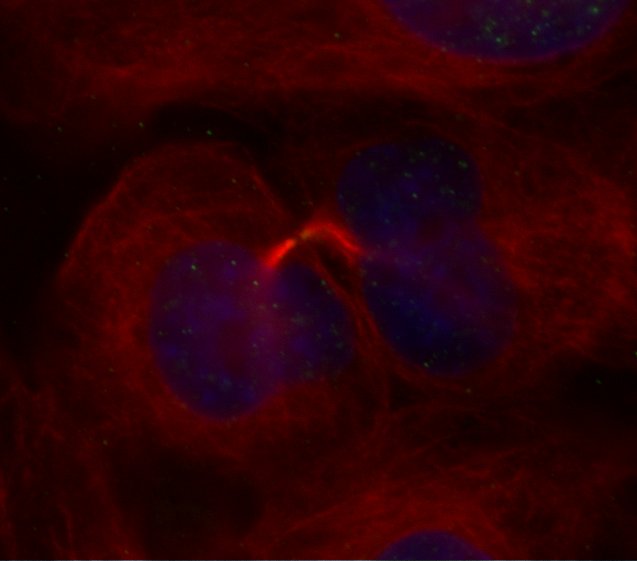

Supplement: Supplementary file 9 — Source data Fig. 4 [file 44318_2025_453_MOESM9_ESM.zip › Figure 4/4E/BLM KO Raw.jpg]

**pCHK1**

**GapDH**

**pHisH3**

**BLM**


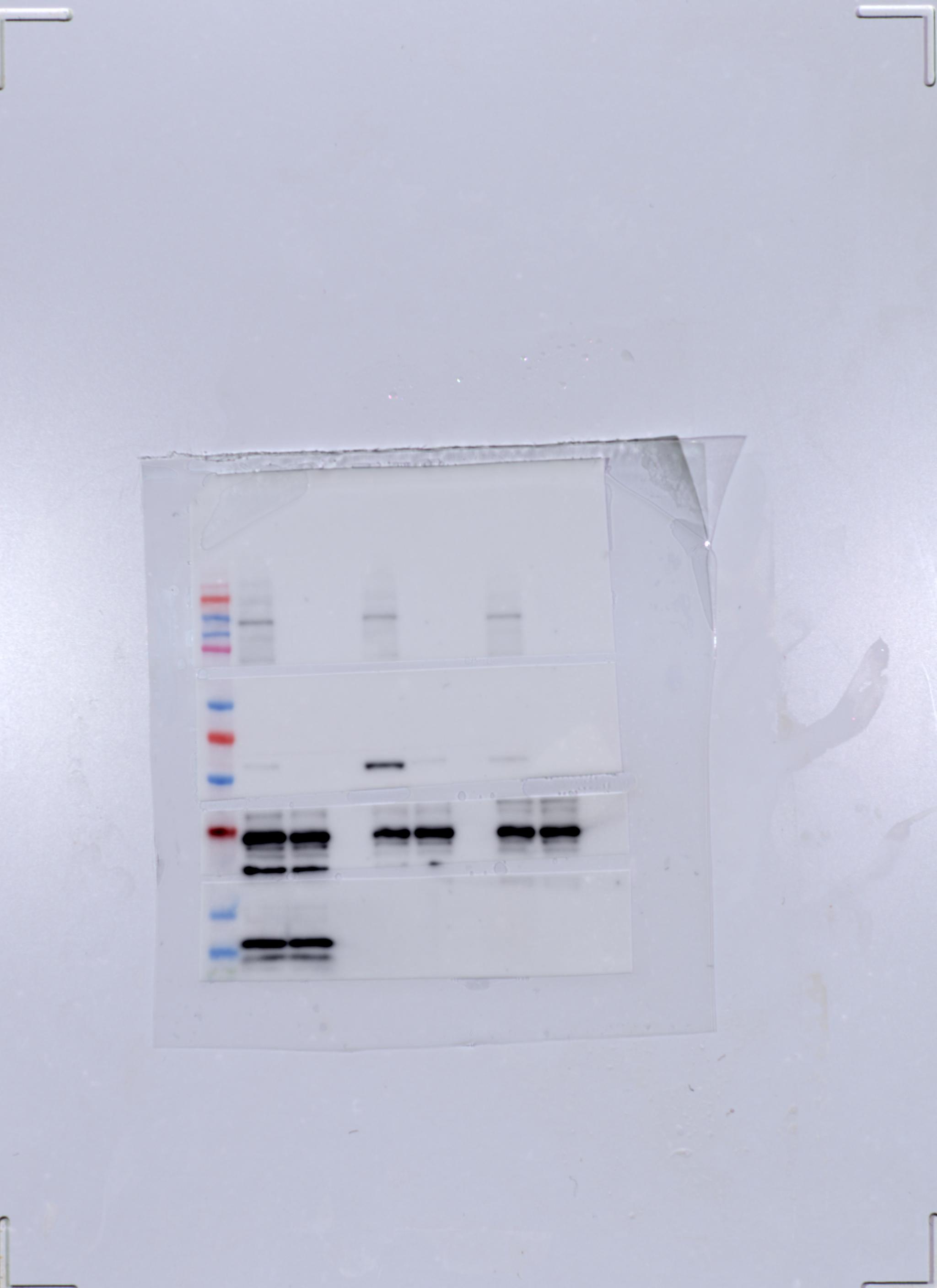

Supplement: Supplementary file 9 — Source data Fig. 4 [file 44318_2025_453_MOESM9_ESM.zip › Figure 4/4B/Readme Fig 4B.docx]

**pHisH3**

**GapDH**

**pCHK1**

**BLM**


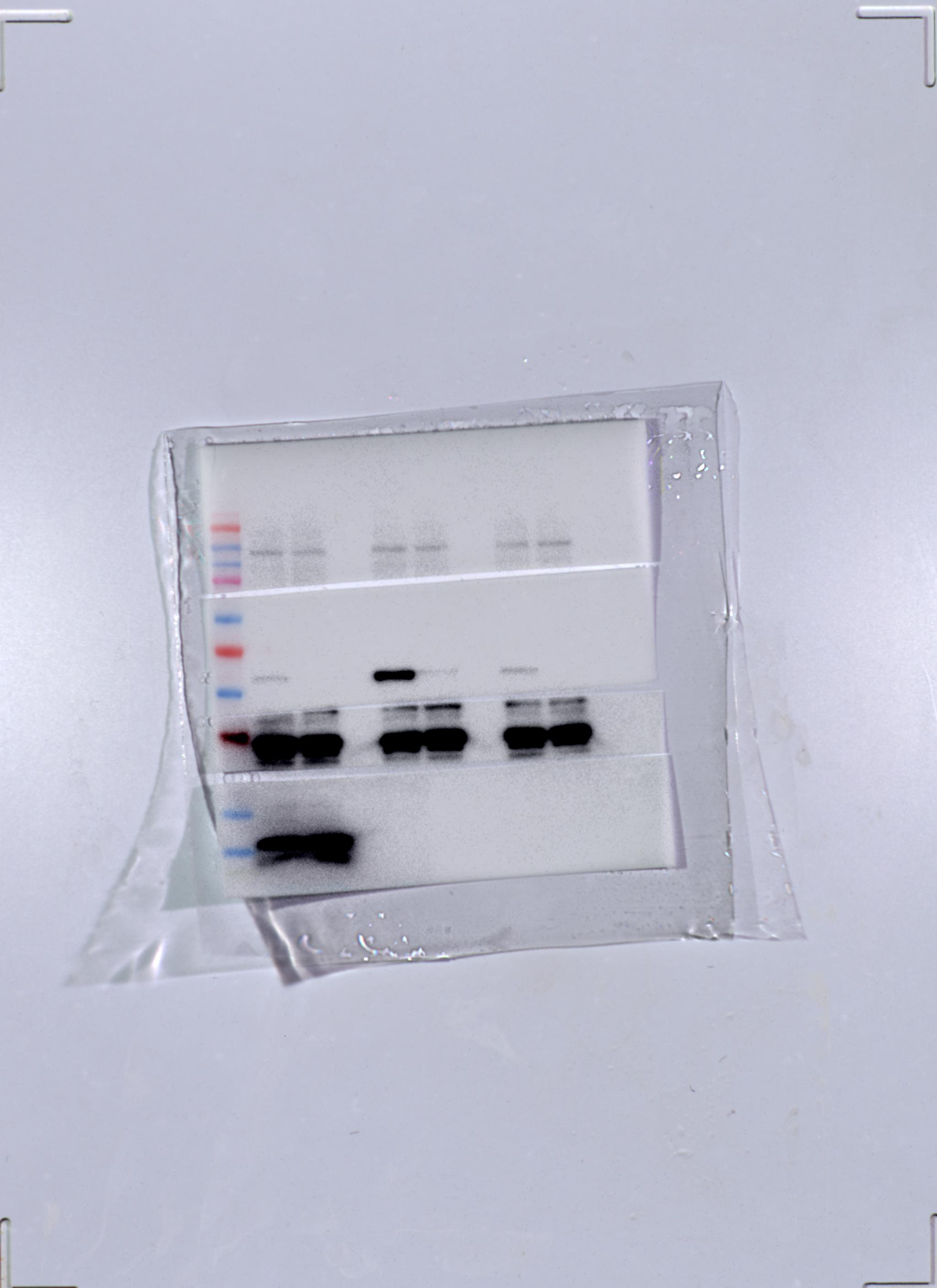

Supplement: Supplementary file 9 — Source data Fig. 4 [file 44318_2025_453_MOESM9_ESM.zip › Figure 4/4D/Readme Fig 4D.docx]

BLM-/-

BLMWT

pAurora B

pCHK1


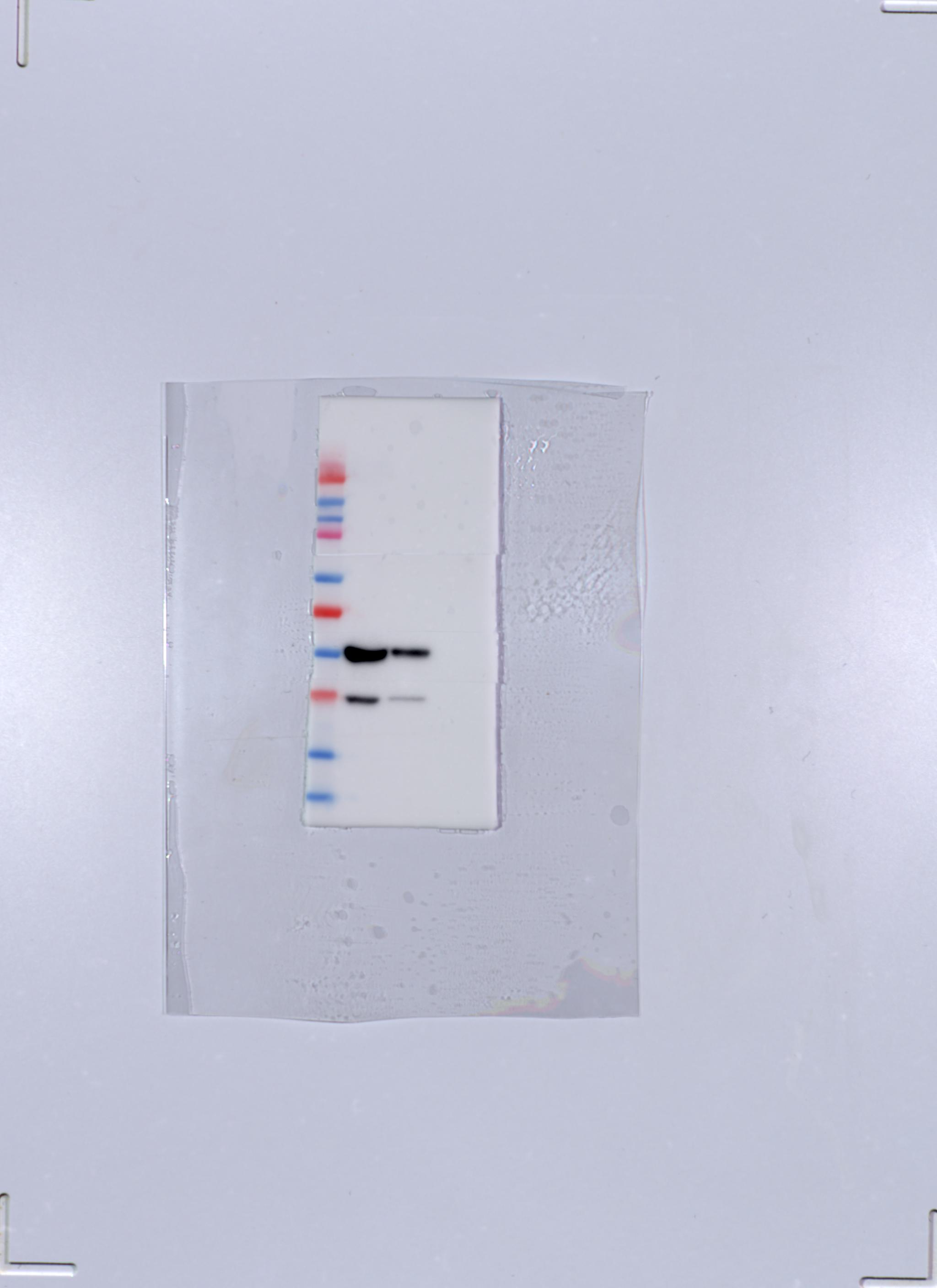


RACGAP1


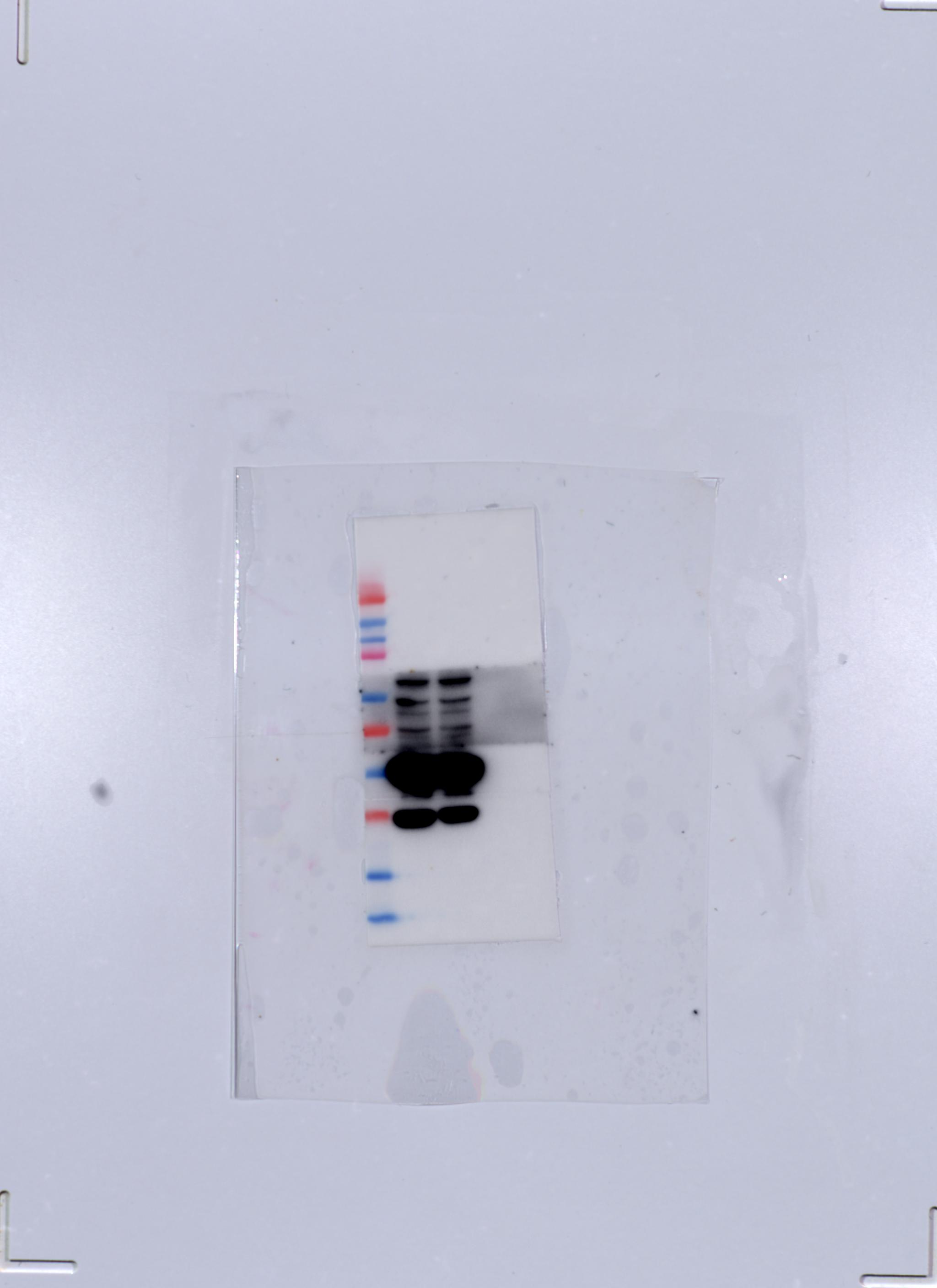


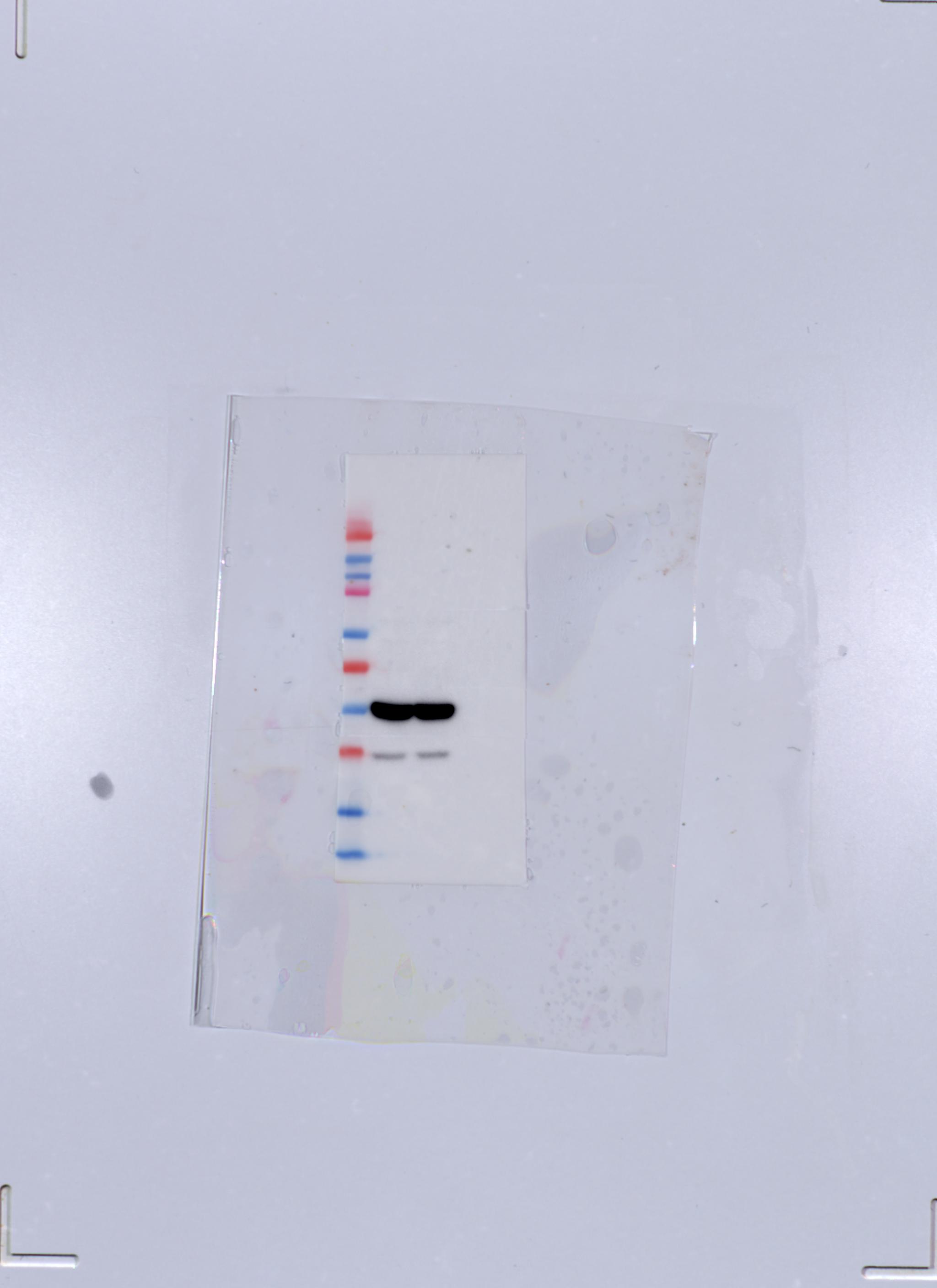


CHK1

Aurora B

Supplement: Supplementary file 9 — Source data Fig. 4 [file 44318_2025_453_MOESM9_ESM.zip › Figure 4/4G/Readme Fig 4G.docx]

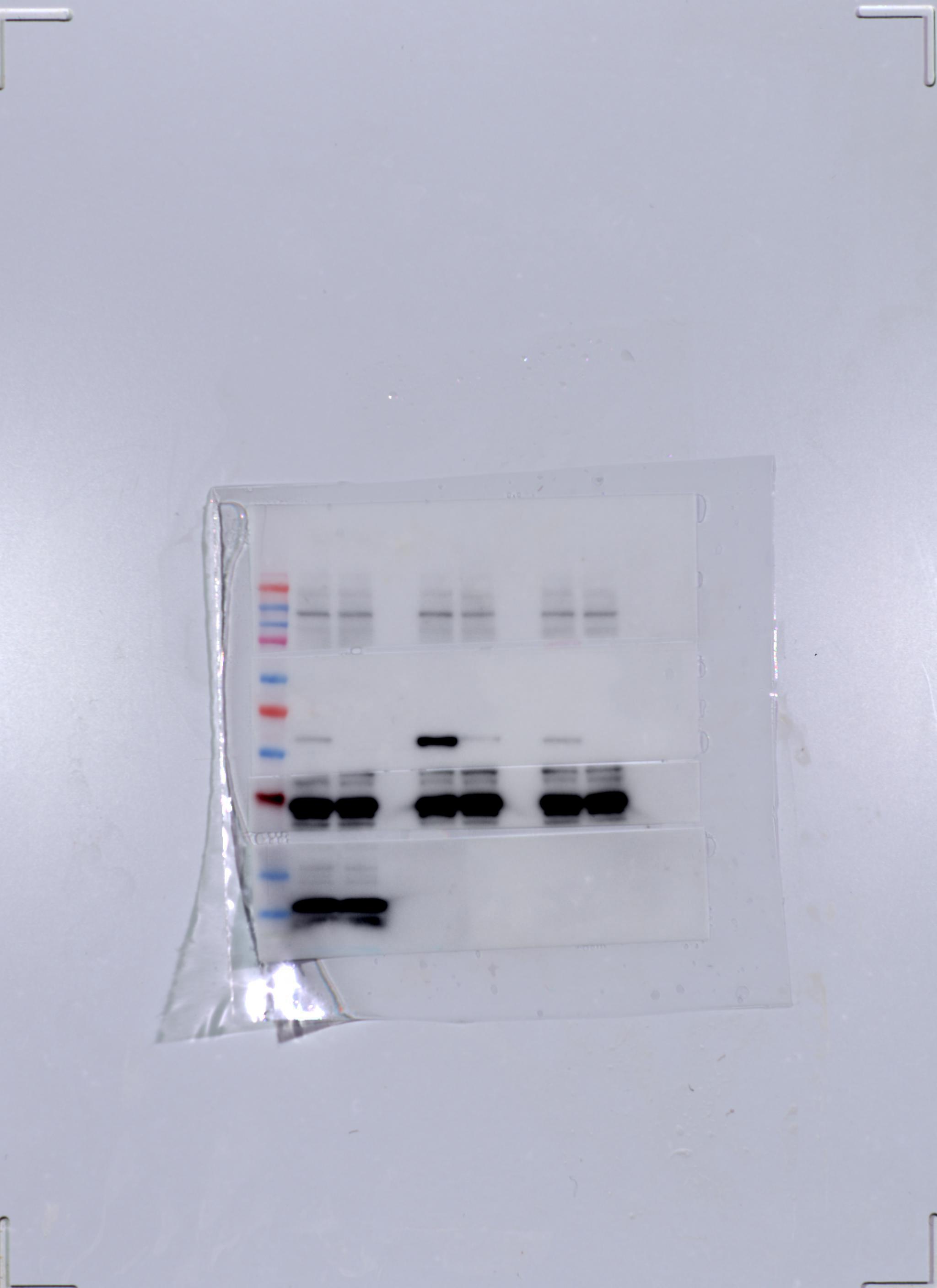

Supplement: Supplementary file 9 — Source data Fig. 4 [file 44318_2025_453_MOESM9_ESM.zip › Figure 4/4D/2021.12.20_17.38.24_Ch+Marker.jpg]

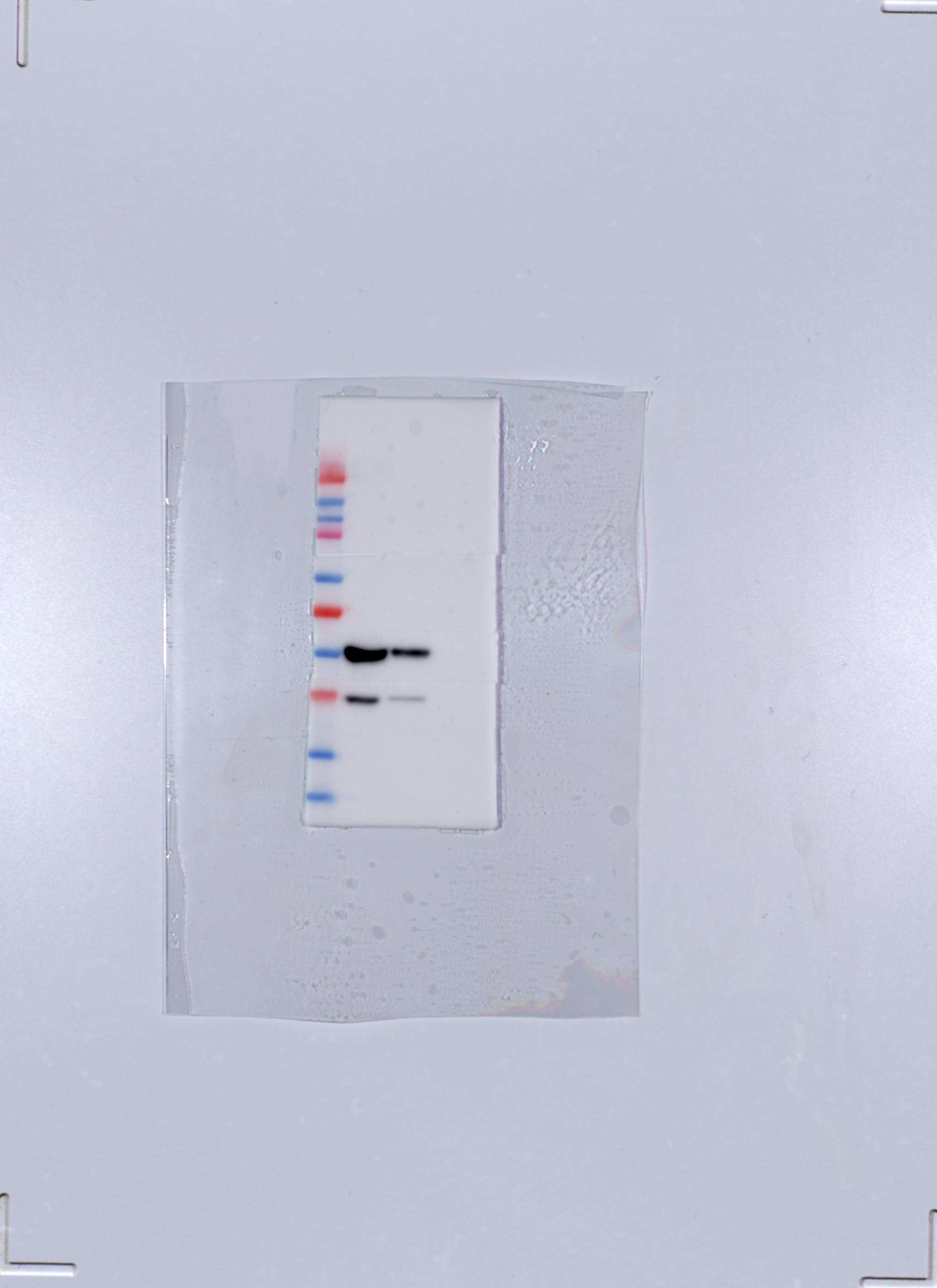

Supplement: Supplementary file 9 — Source data Fig. 4 [file 44318_2025_453_MOESM9_ESM.zip › Figure 4/4G/2022.02.17_16.33.38_Ch+Marker.jpg]

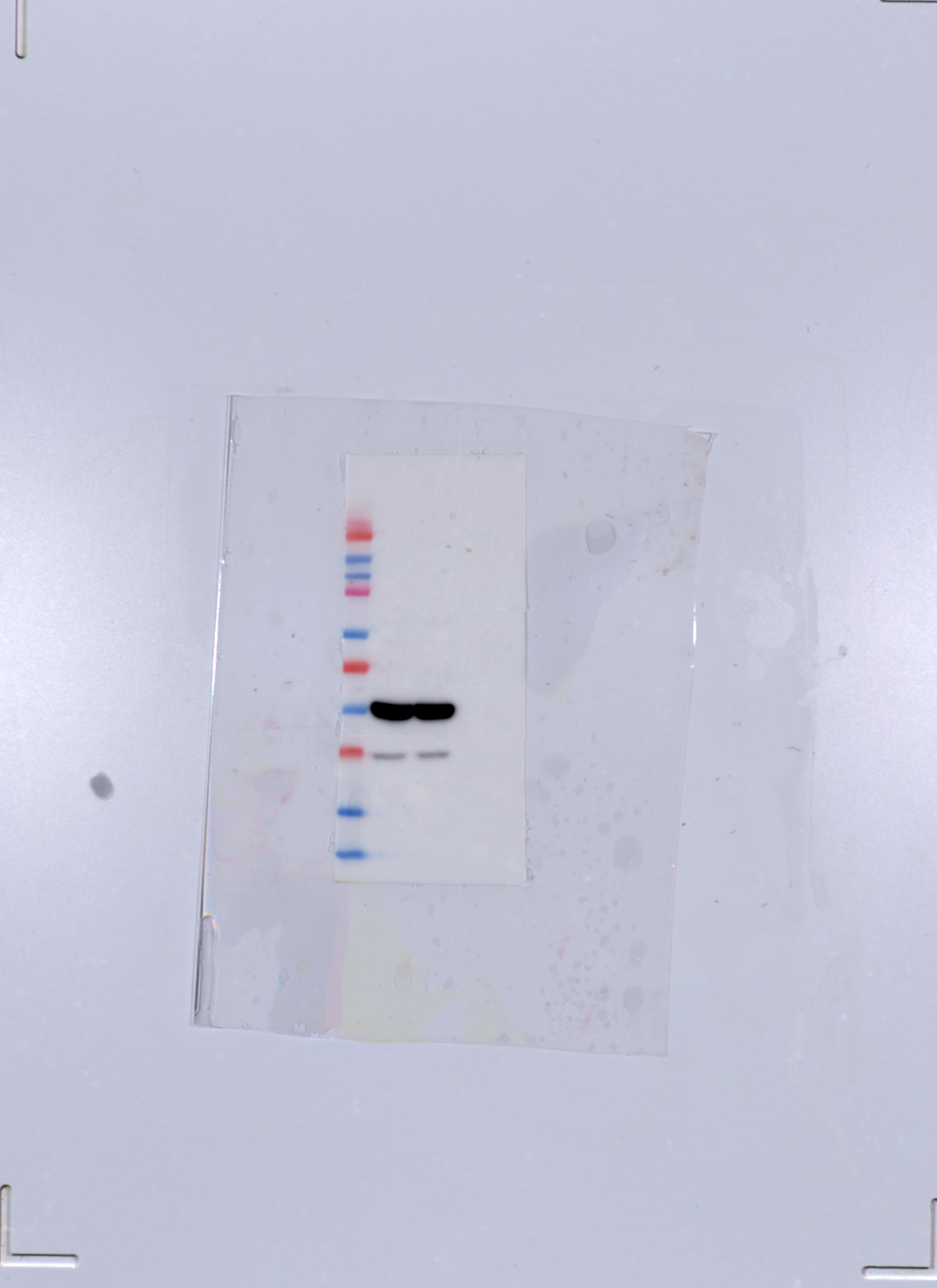

Supplement: Supplementary file 9 — Source data Fig. 4 [file 44318_2025_453_MOESM9_ESM.zip › Figure 4/4G/2022.02.17_19.11.13_Ch+Marker.jpg]

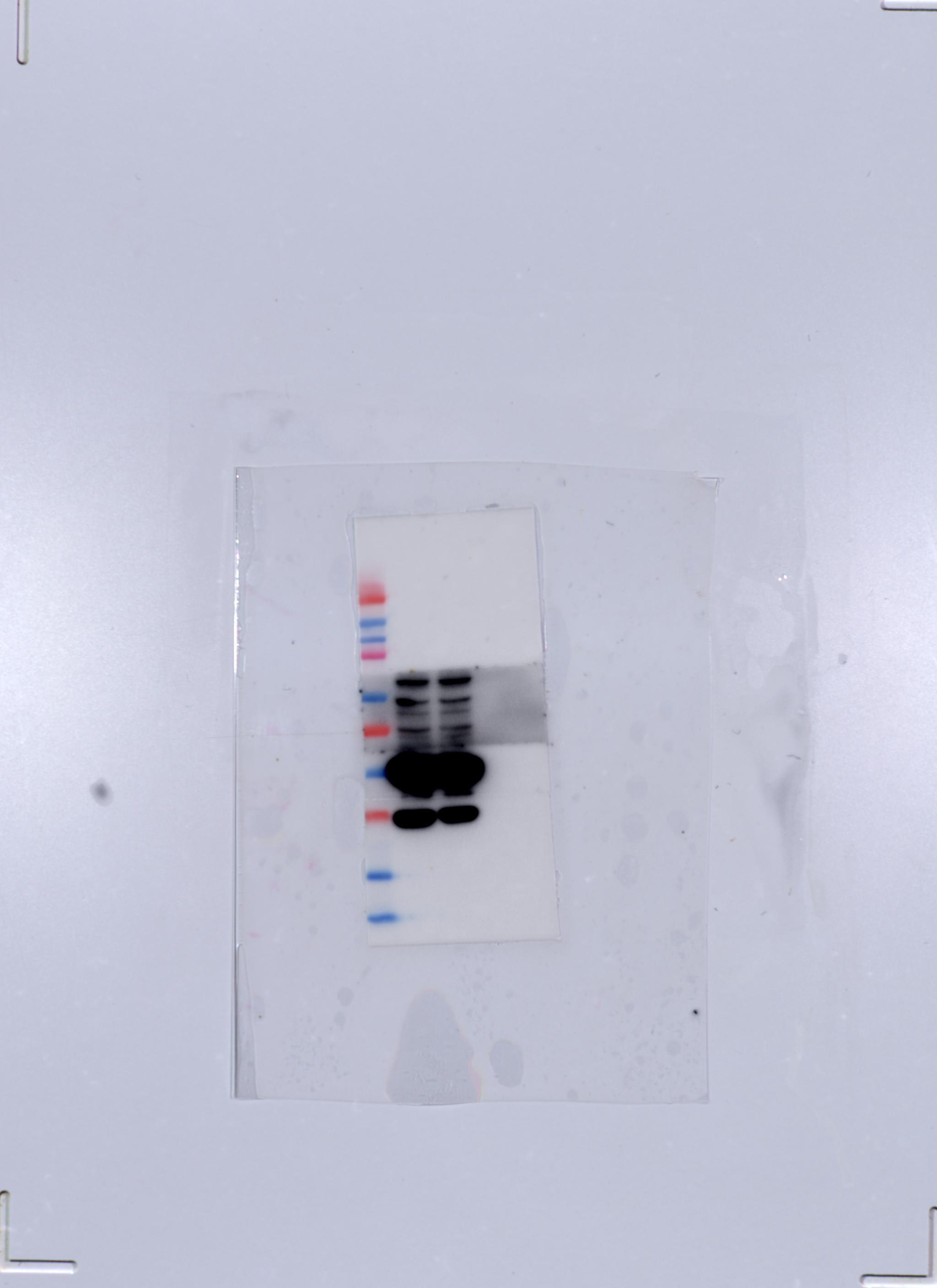

Supplement: Supplementary file 9 — Source data Fig. 4 [file 44318_2025_453_MOESM9_ESM.zip › Figure 4/4G/2022.02.17_19.15.27_Ch+Marker.jpg]

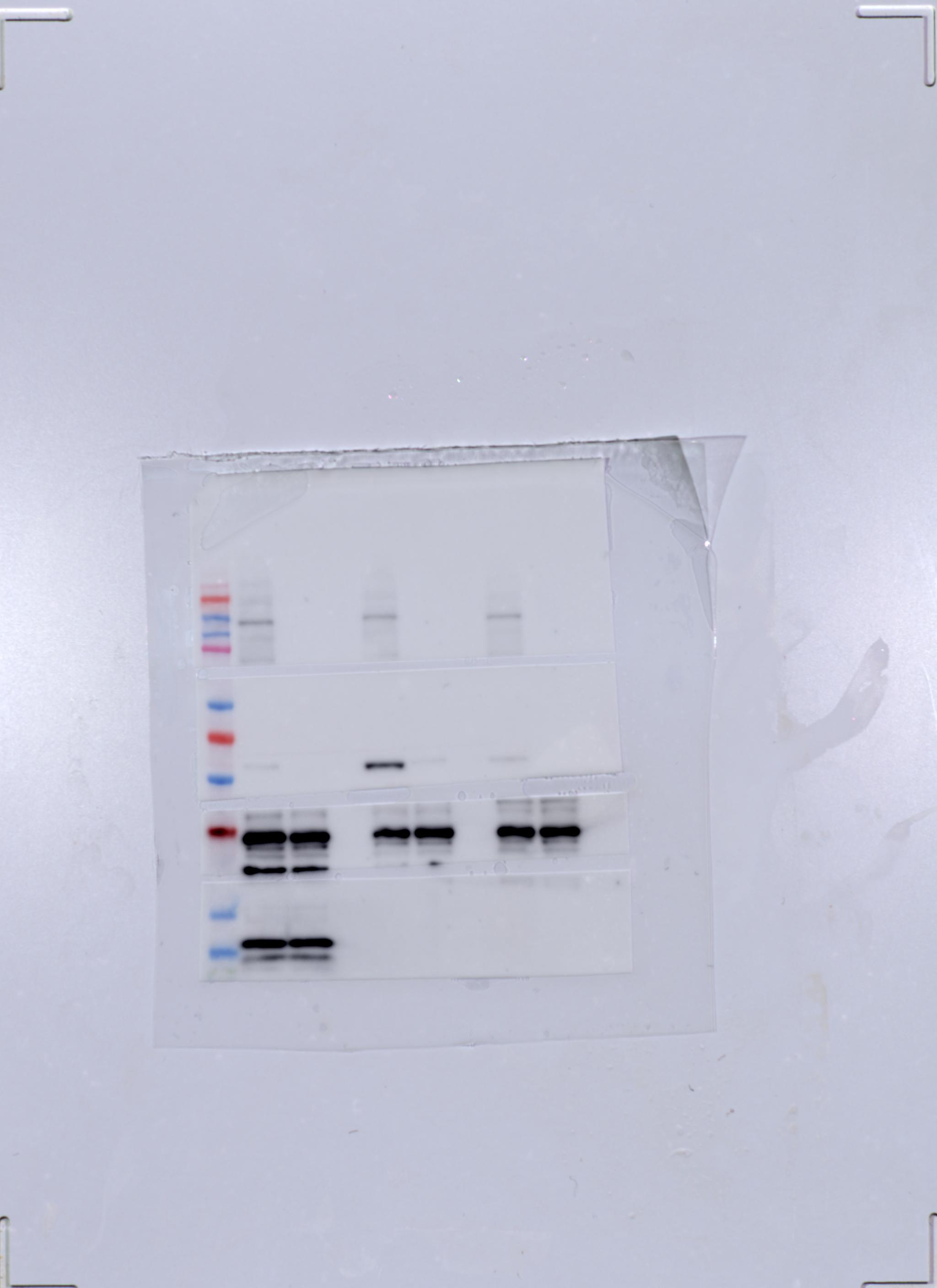

Supplement: Supplementary file 9 — Source data Fig. 4 [file 44318_2025_453_MOESM9_ESM.zip › Figure 4/4B/jjh 2021.12.20_17.42.10_Ch+Marker (1).jpg]

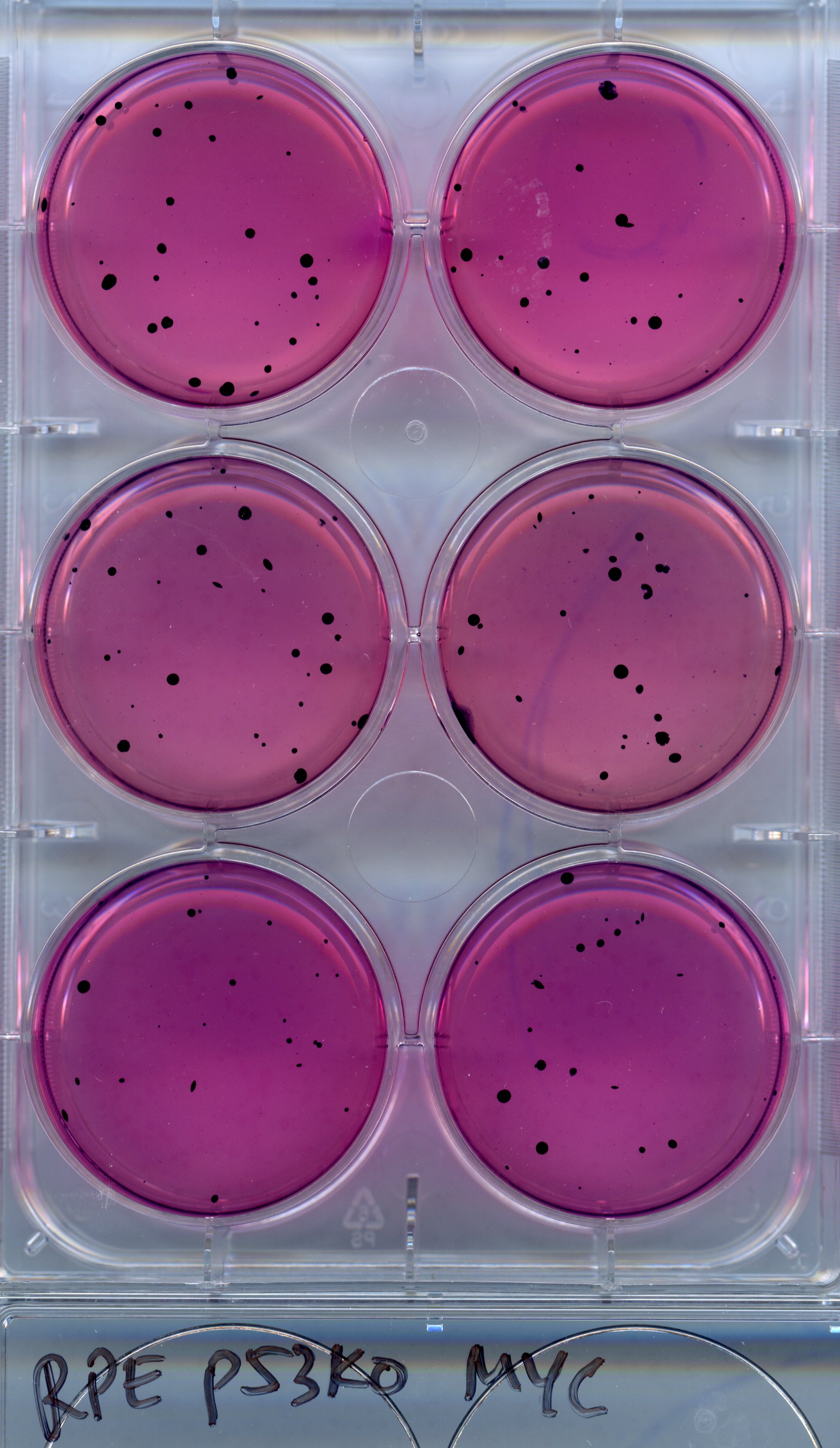

Supplement: Supplementary file 10 — Source data Fig. 5 [file 44318_2025_453_MOESM10_ESM.zip › Figure 5/5F/RPE p53KO MYC (5).jpg]

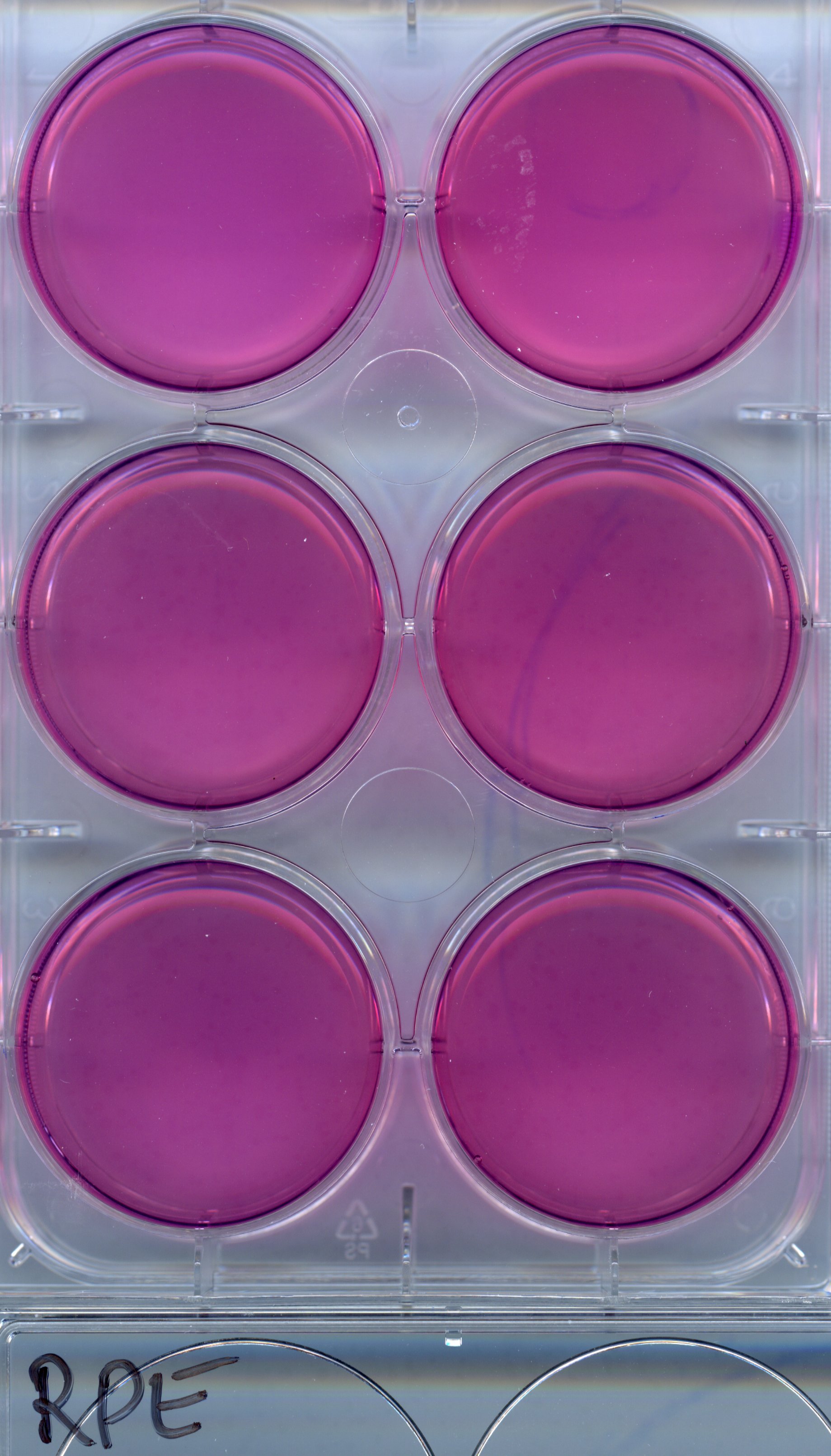

Supplement: Supplementary file 10 — Source data Fig. 5 [file 44318_2025_453_MOESM10_ESM.zip › Figure 5/5F/RPE (5).jpg]

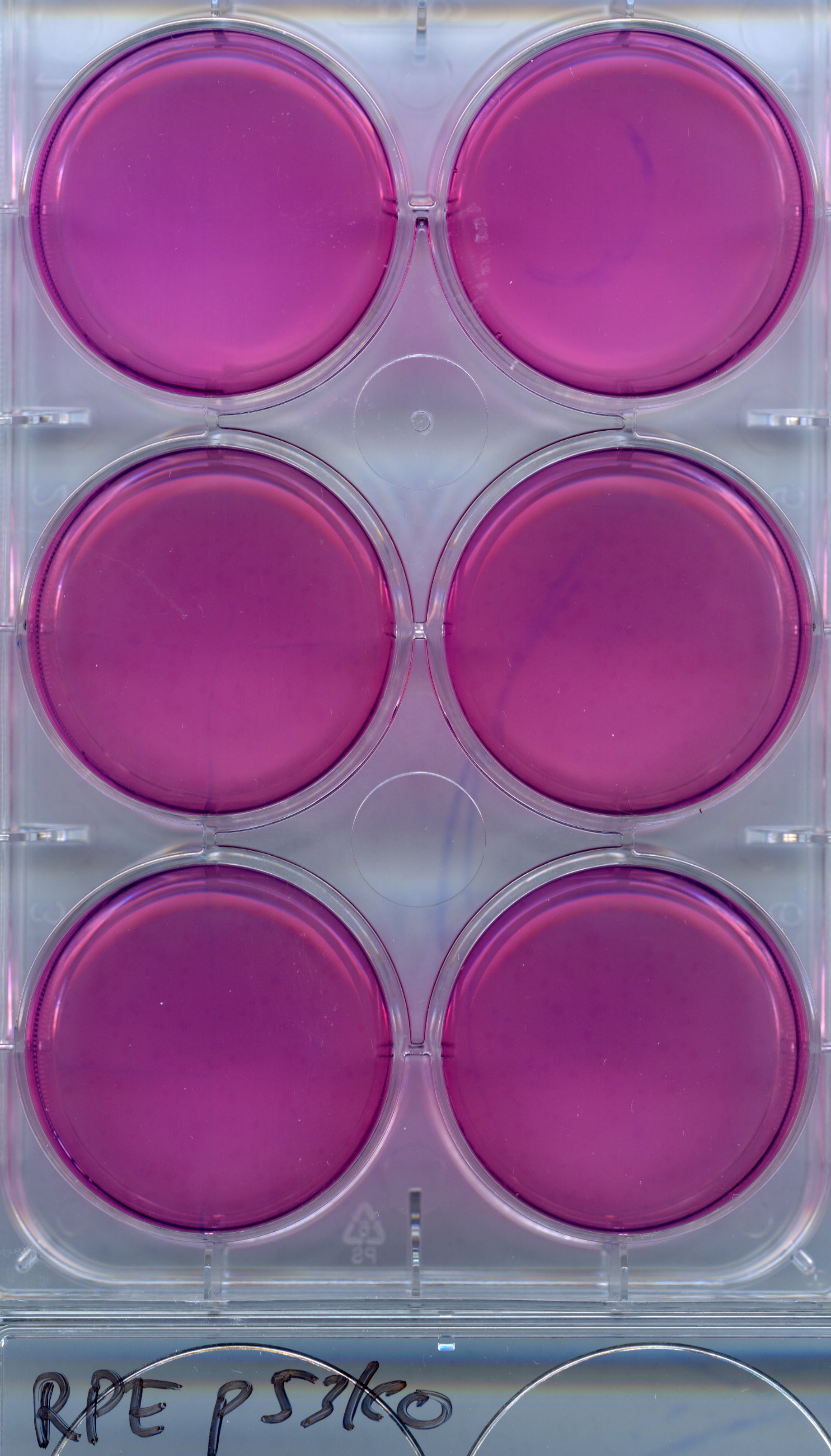

Supplement: Supplementary file 10 — Source data Fig. 5 [file 44318_2025_453_MOESM10_ESM.zip › Figure 5/5F/RPE p53KO (5).jpg]

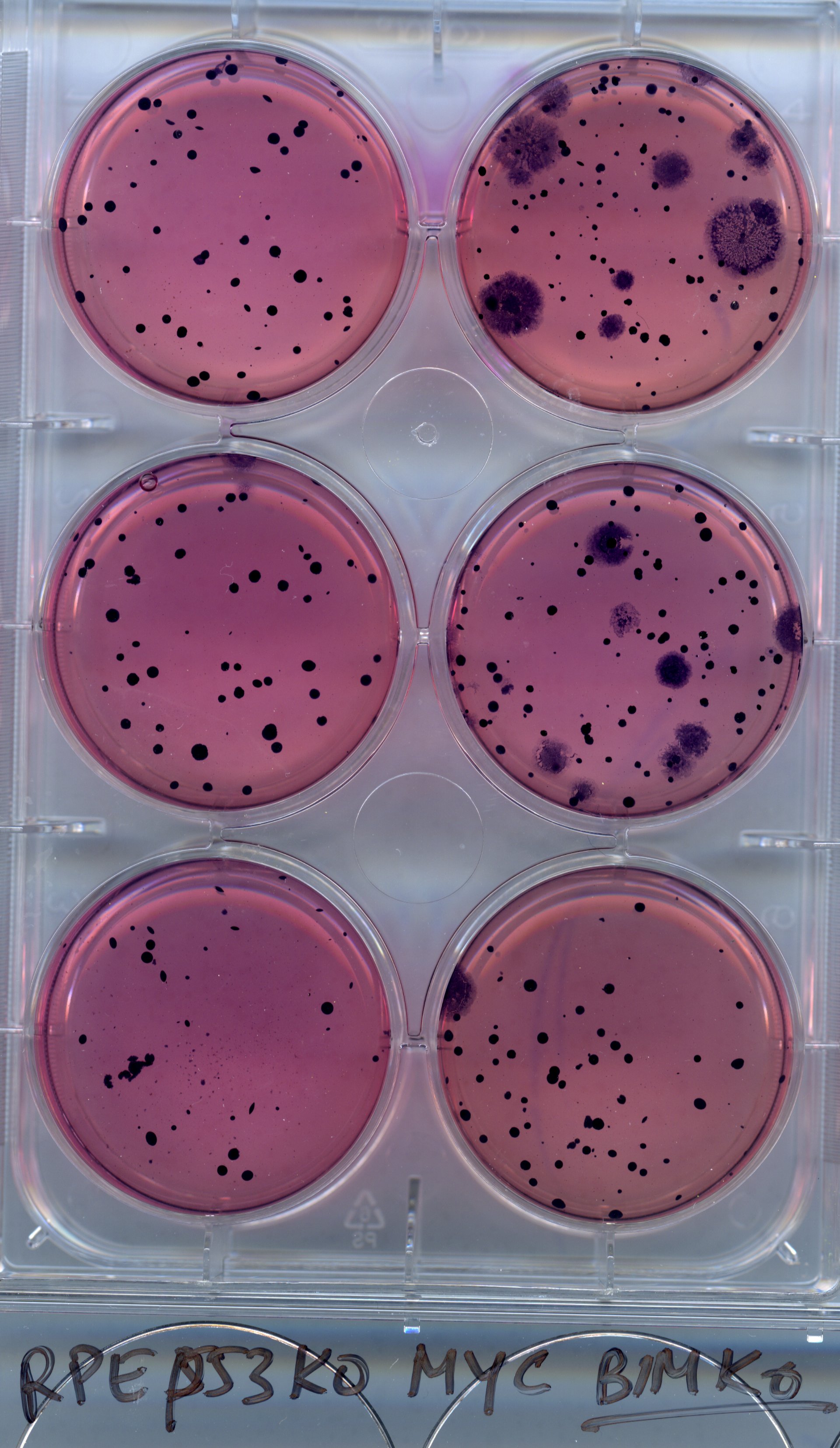

Supplement: Supplementary file 10 — Source data Fig. 5 [file 44318_2025_453_MOESM10_ESM.zip › Figure 5/5F/RPE p53KO BLMKO MYC (5).jpg]

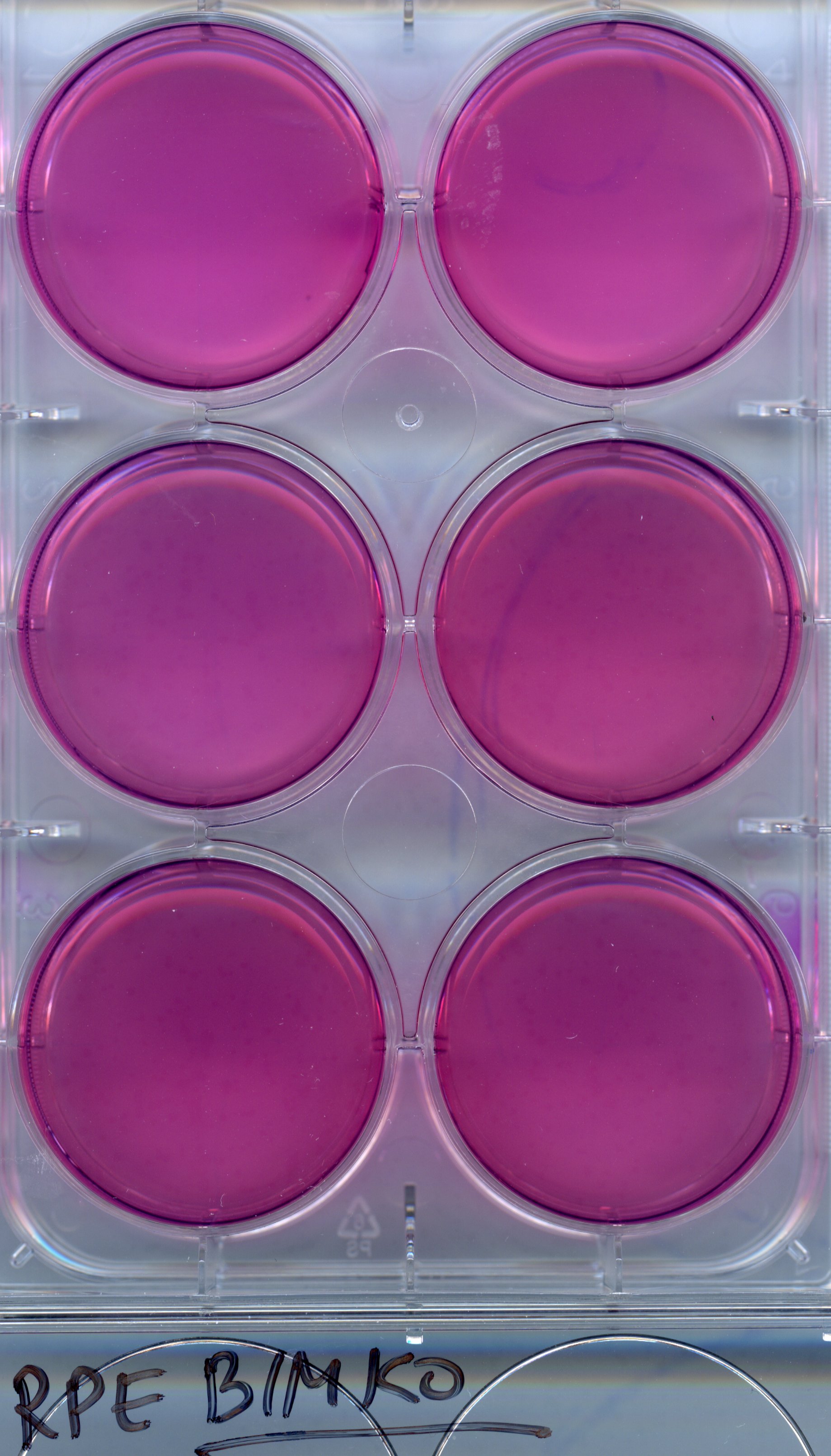

Supplement: Supplementary file 10 — Source data Fig. 5 [file 44318_2025_453_MOESM10_ESM.zip › Figure 5/5F/RPE BLMKO (5).jpg]

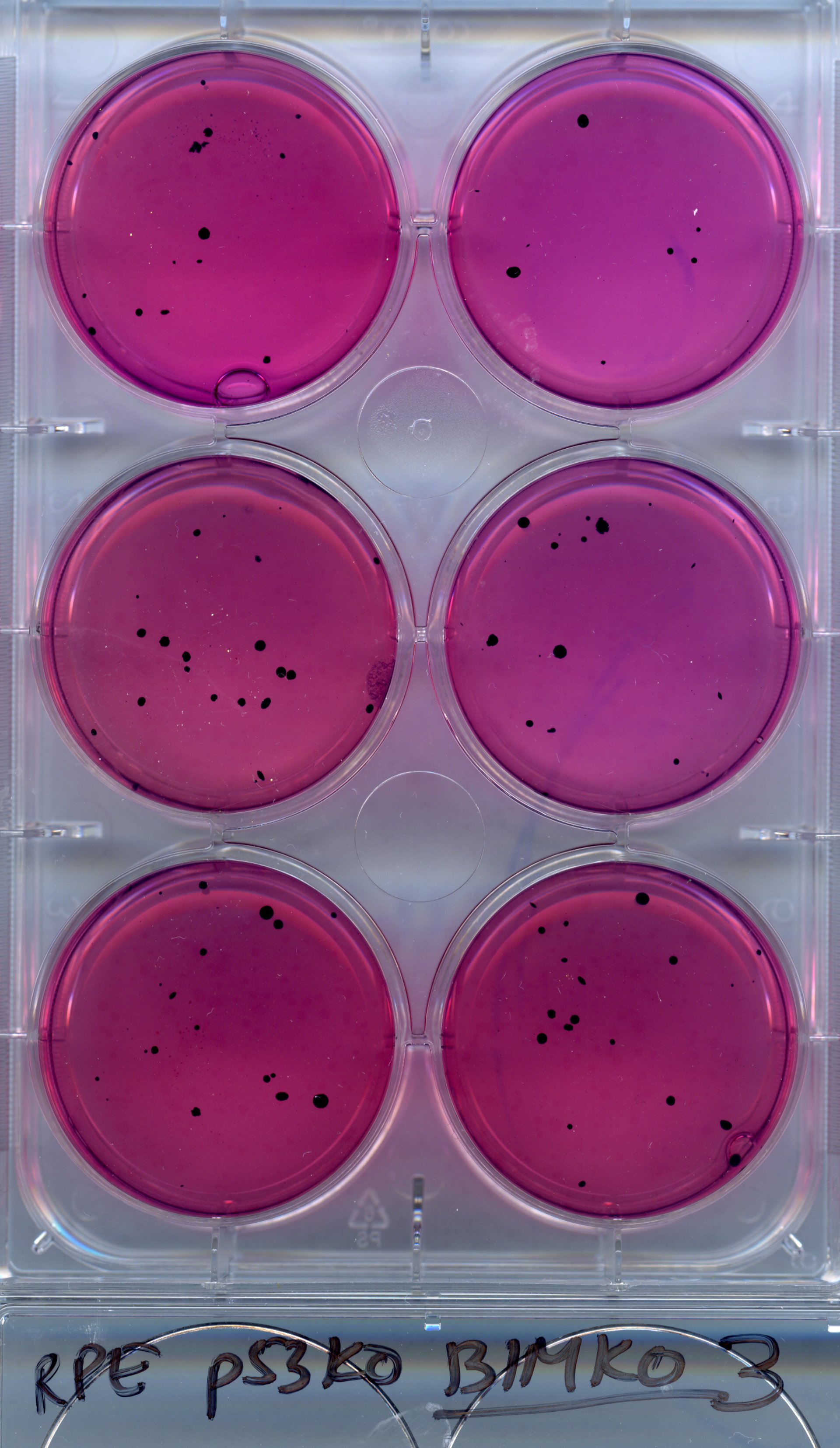

Supplement: Supplementary file 10 — Source data Fig. 5 [file 44318_2025_453_MOESM10_ESM.zip › Figure 5/5F/RPE p53KO BLMKO-3 (5).jpg]

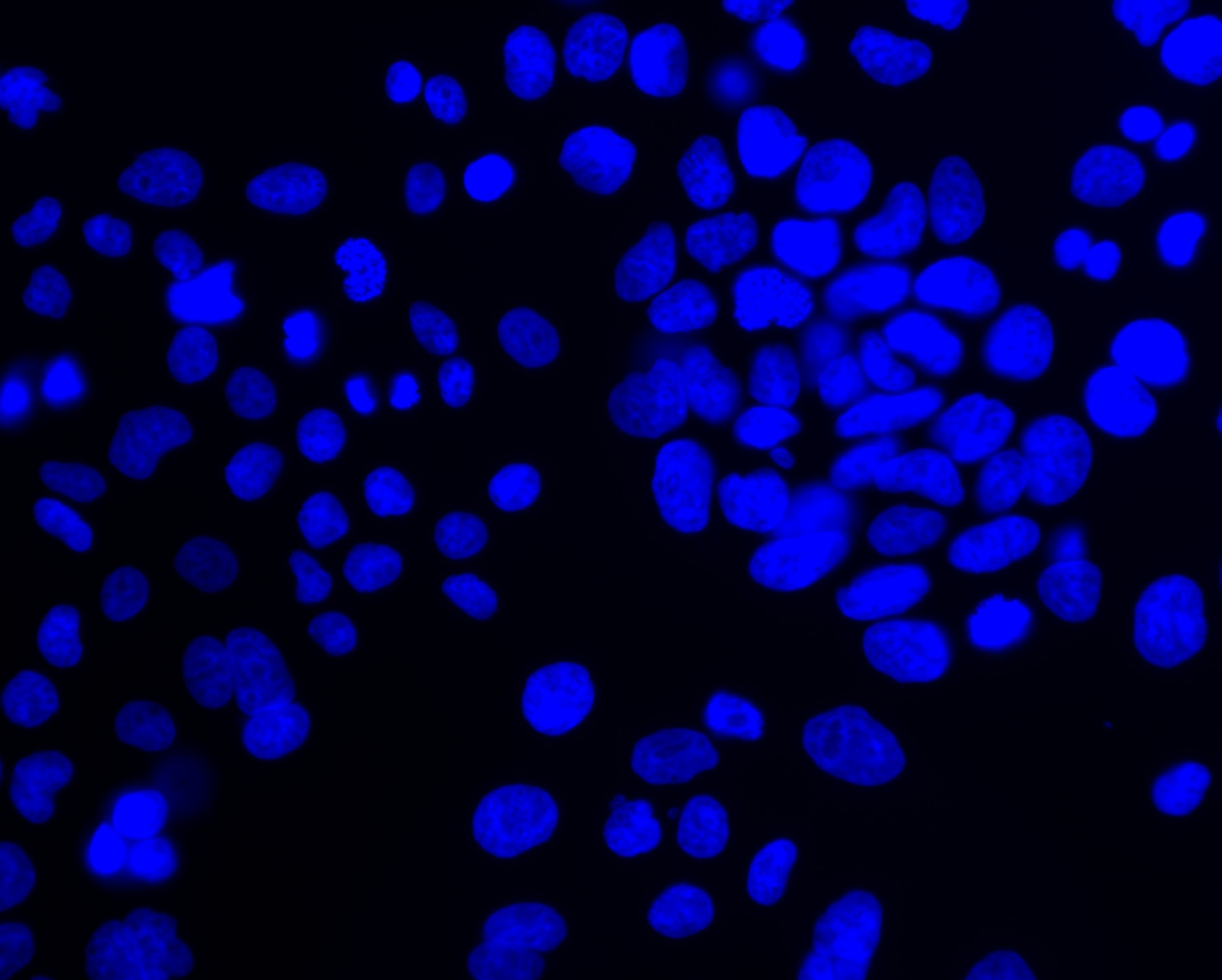

Supplement: Supplementary file 10 — Source data Fig. 5 [file 44318_2025_453_MOESM10_ESM.zip › Figure 5/5C/BLM degron +IAA Raw.jpg]

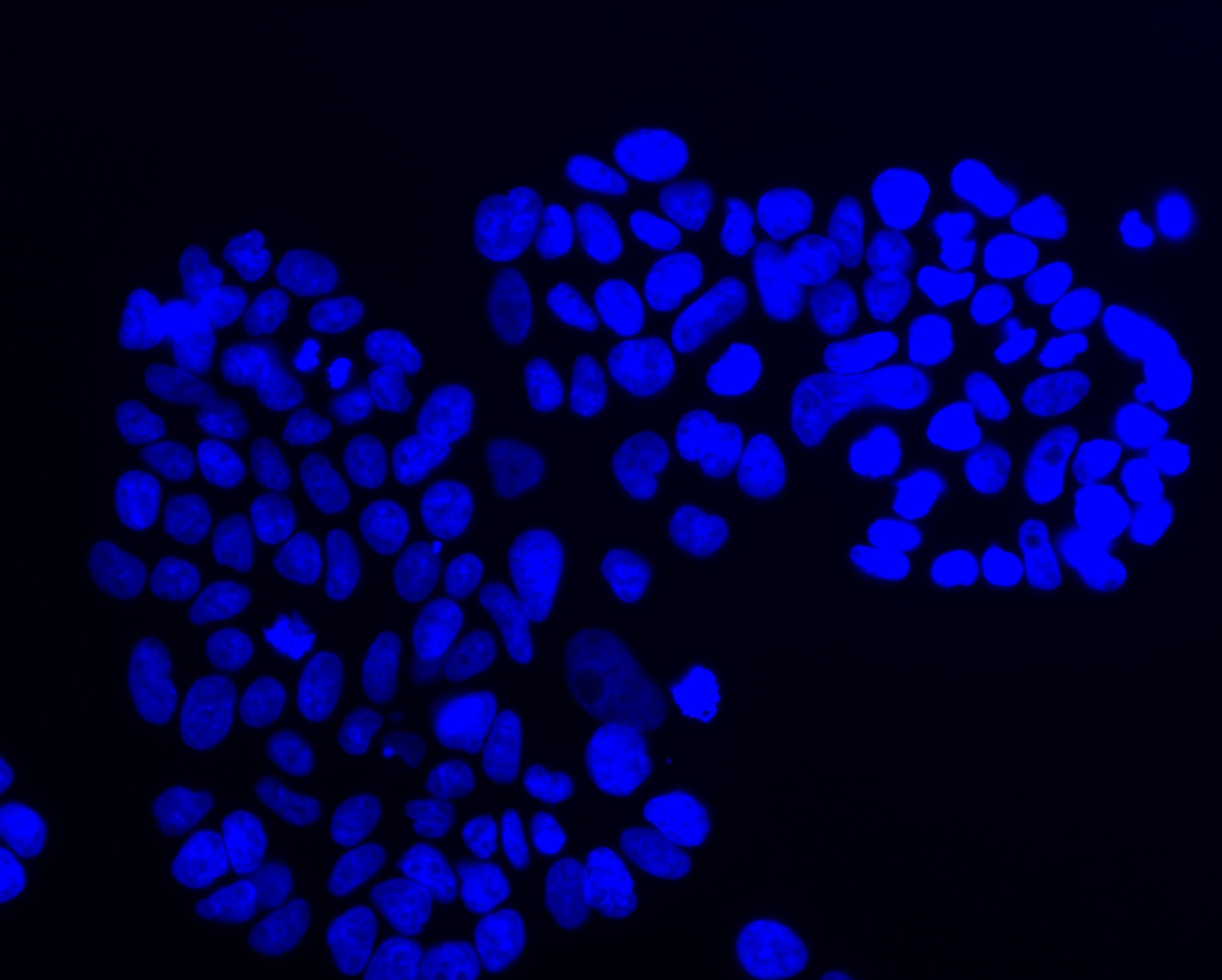

Supplement: Supplementary file 10 — Source data Fig. 5 [file 44318_2025_453_MOESM10_ESM.zip › Figure 5/5C/dldi raw.jpg]

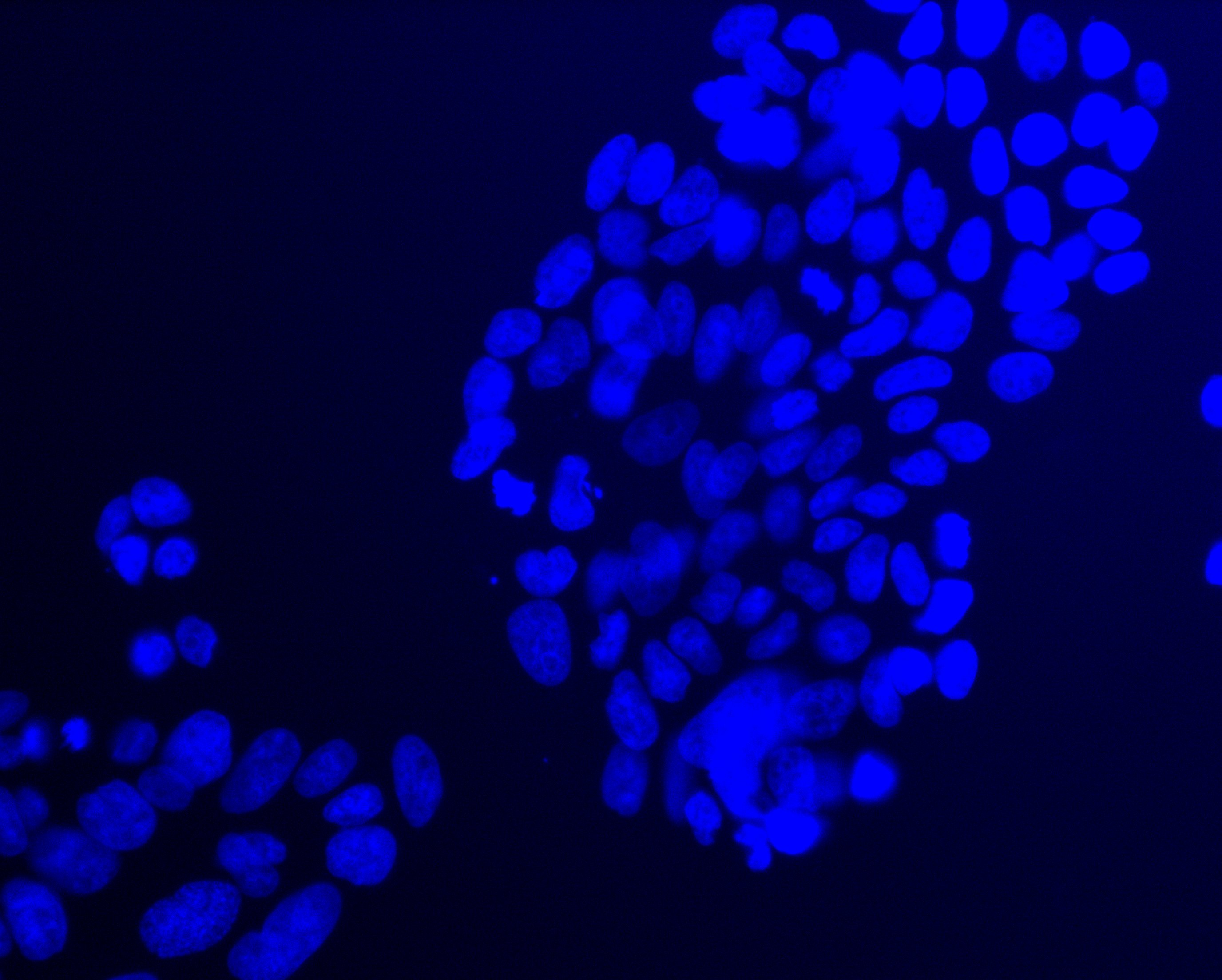

Supplement: Supplementary file 10 — Source data Fig. 5 [file 44318_2025_453_MOESM10_ESM.zip › Figure 5/5C/BLM degron -IAA Raw.jpg]

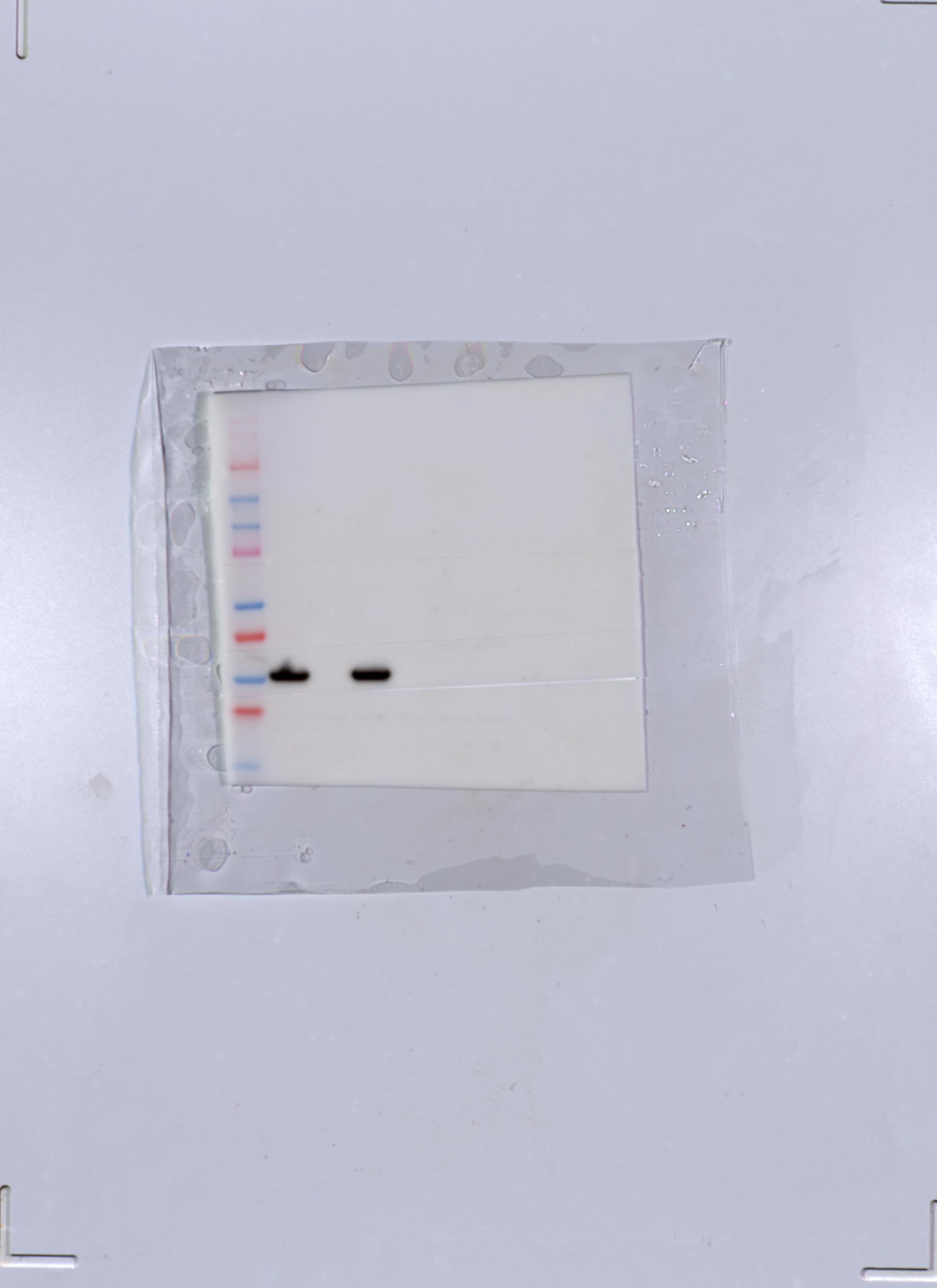

Supplement: Supplementary file 10 — Source data Fig. 5 [file 44318_2025_453_MOESM10_ESM.zip › Figure 5/5E/2022.02.24_21.47.59_Ch+Marker.jpg]

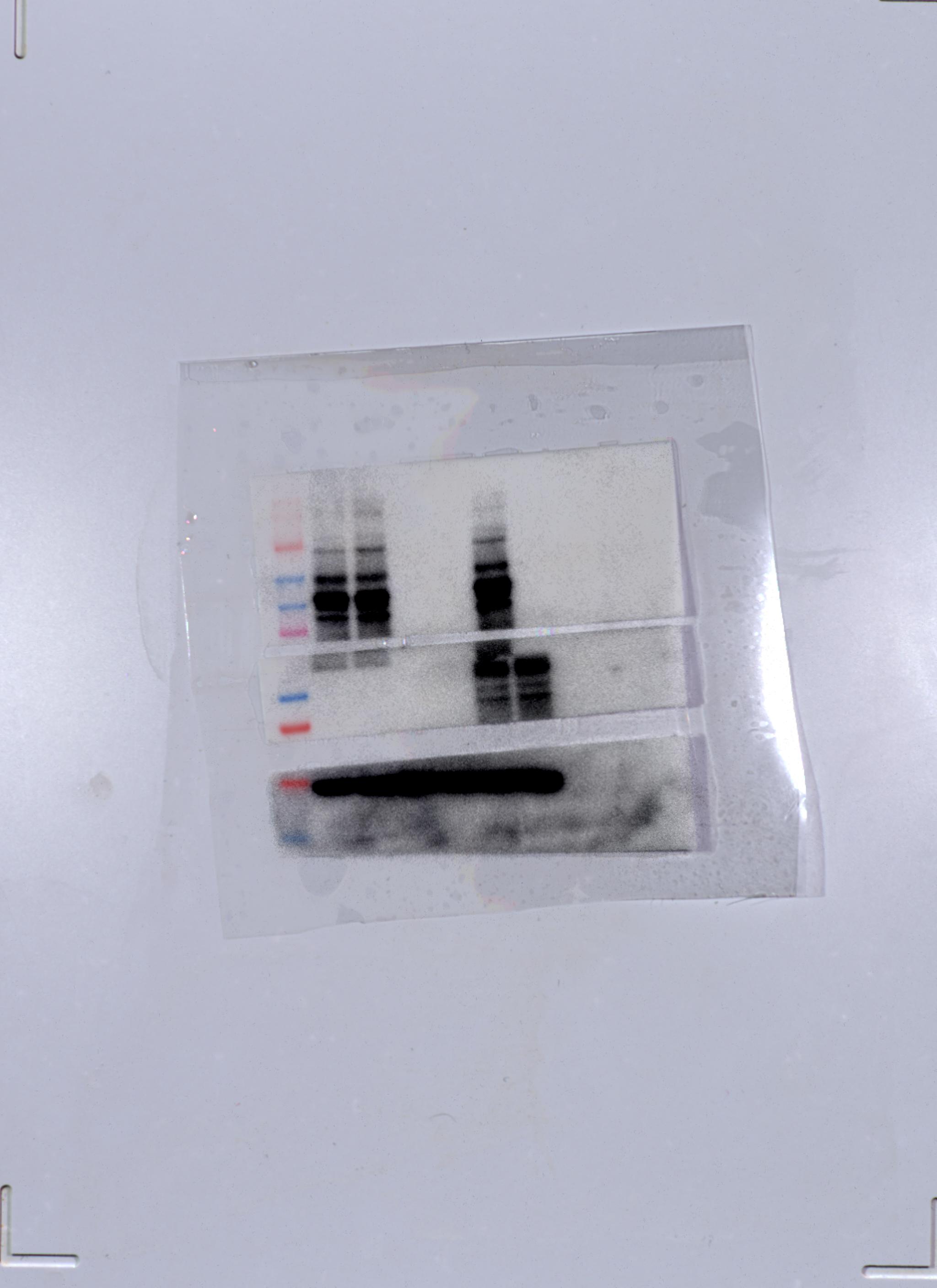

Supplement: Supplementary file 10 — Source data Fig. 5 [file 44318_2025_453_MOESM10_ESM.zip › Figure 5/5E/2022.02.24_22.05.52_Ch+Marker.jpg]

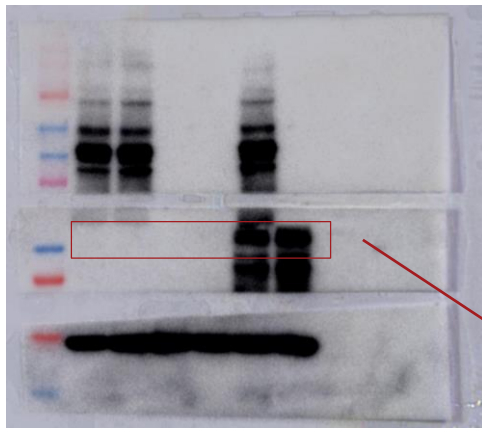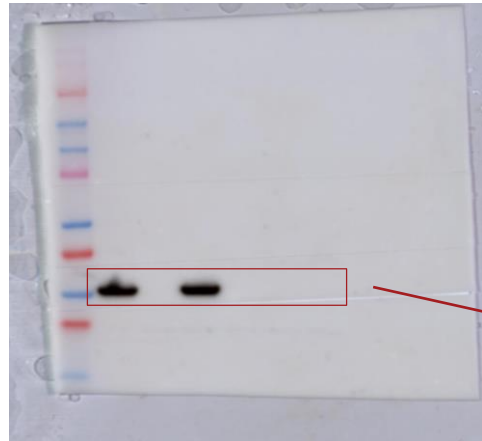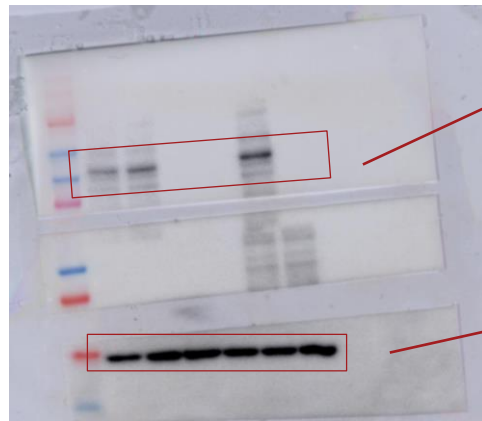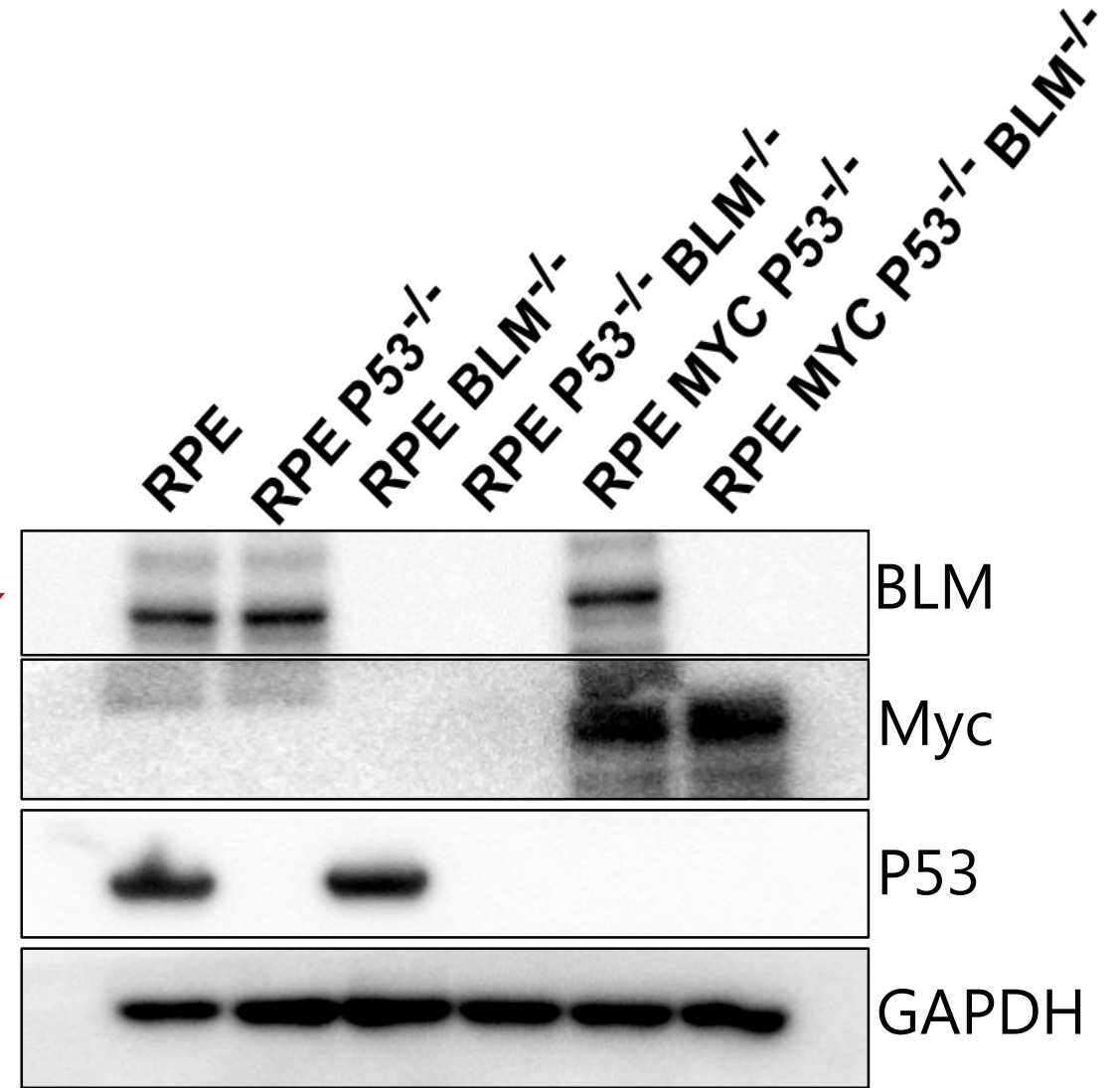

Supplement: Supplementary file 10 — Source data Fig. 5 [file 44318_2025_453_MOESM10_ESM.zip › Figure 5/5E/Figure 5E.pdf]

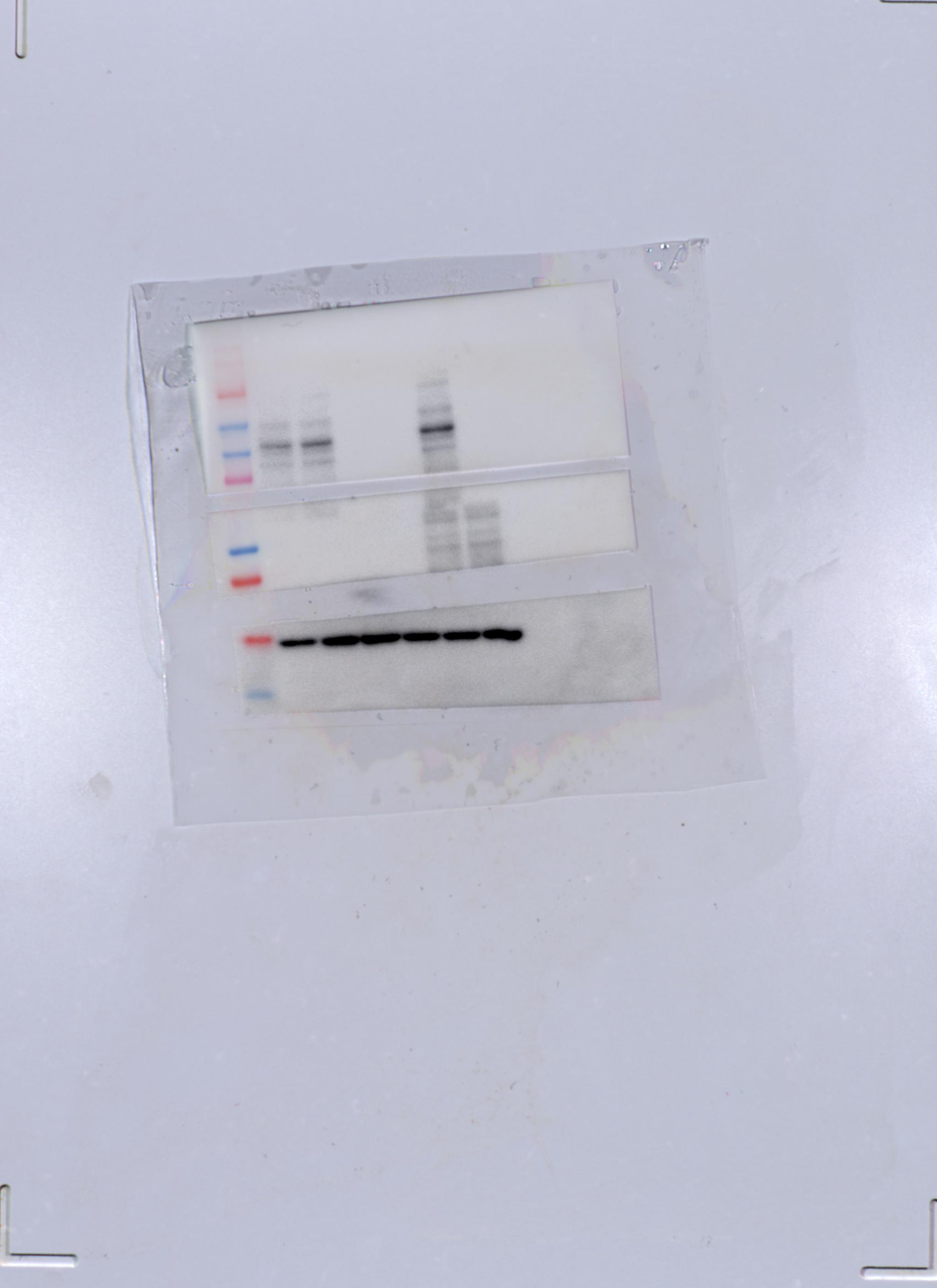

Supplement: Supplementary file 10 — Source data Fig. 5 [file 44318_2025_453_MOESM10_ESM.zip › Figure 5/5E/2022.02.24_21.56.14_Ch+Marker.jpg]

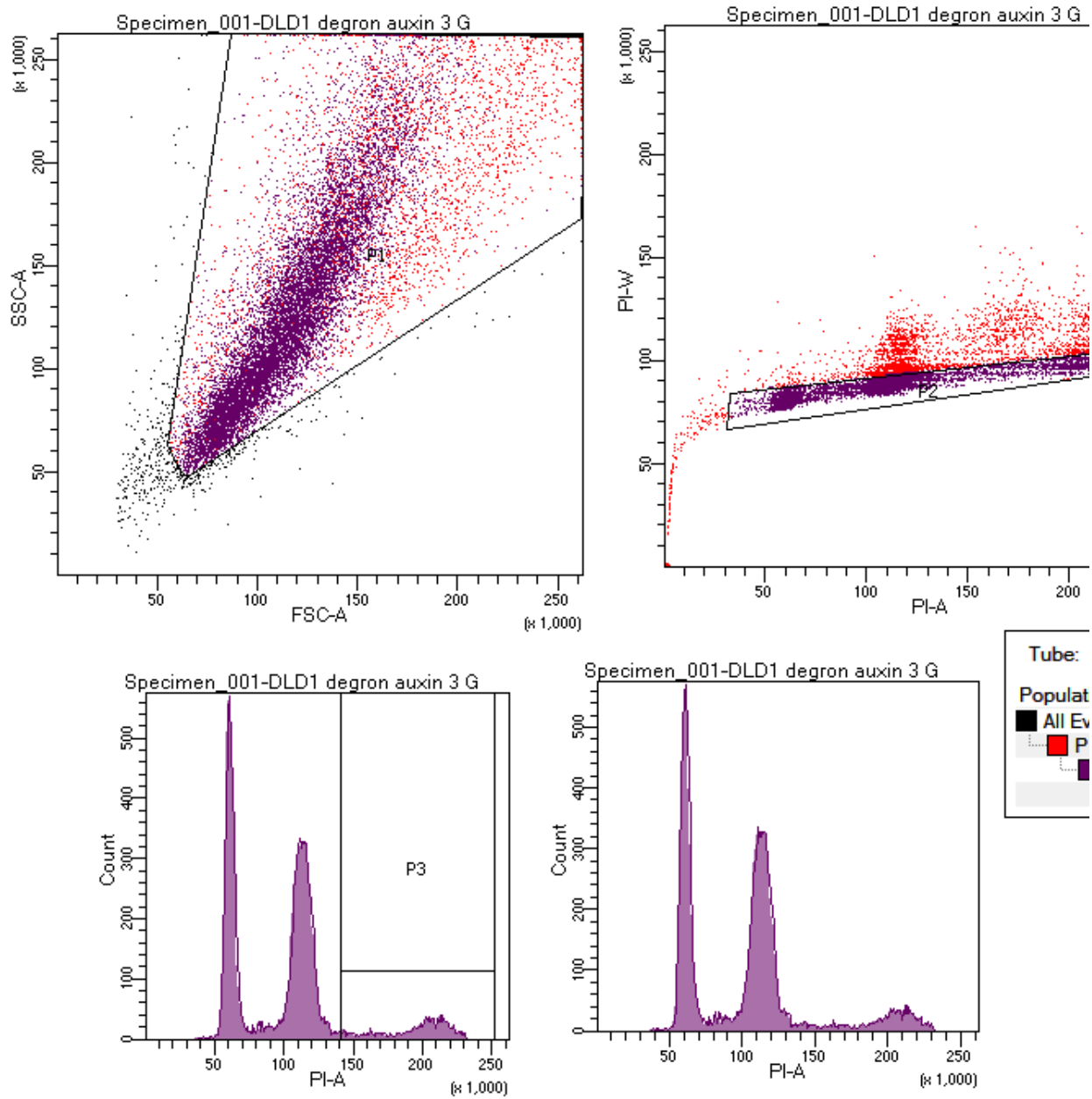

Supplement: Supplementary file 10 — Source data Fig. 5 [file 44318_2025_453_MOESM10_ESM.zip › Figure 5/5B/BLM degrom+IAA.pdf]

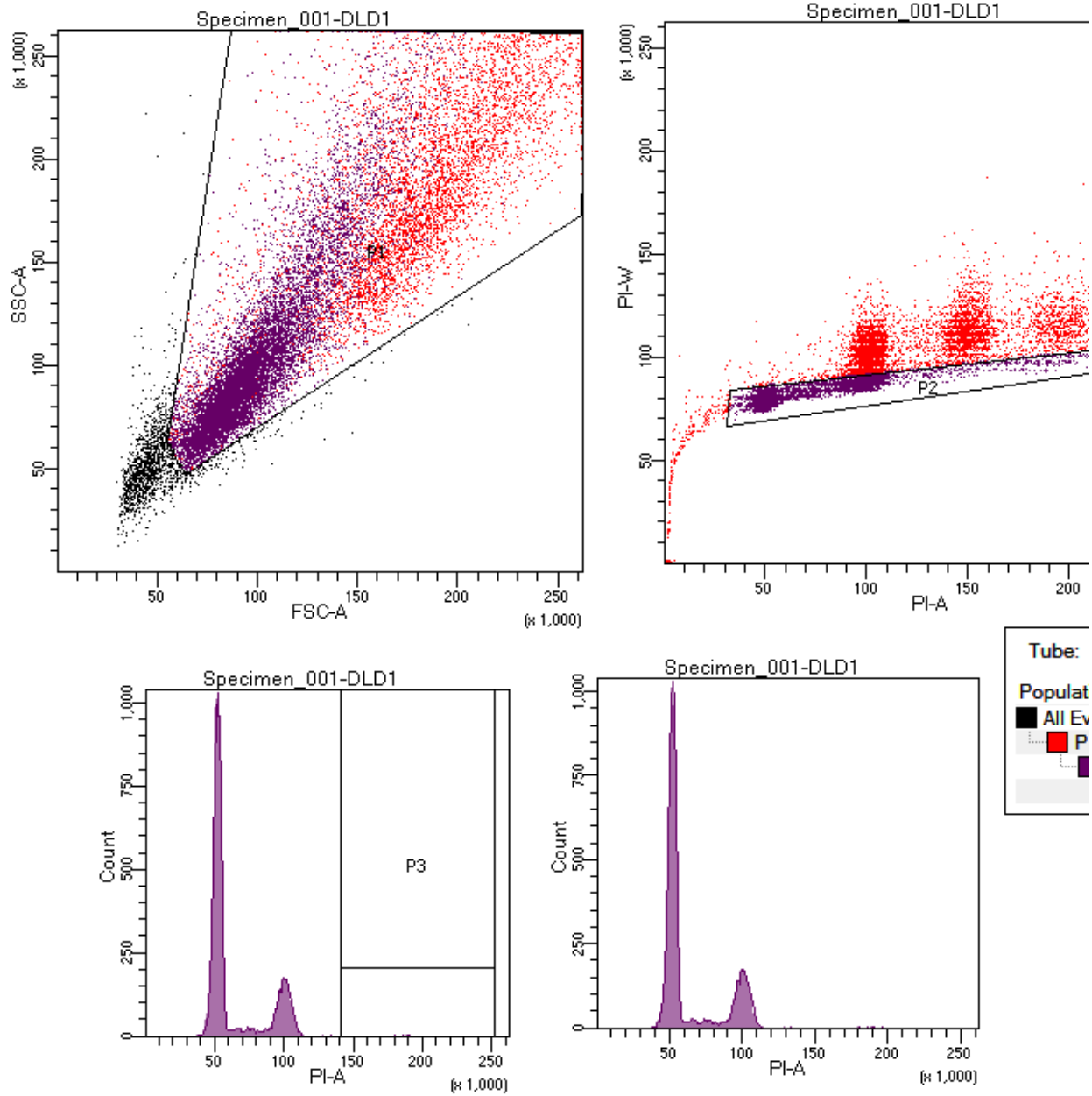

Supplement: Supplementary file 10 — Source data Fig. 5 [file 44318_2025_453_MOESM10_ESM.zip › Figure 5/5B/DLD1 control.pdf]

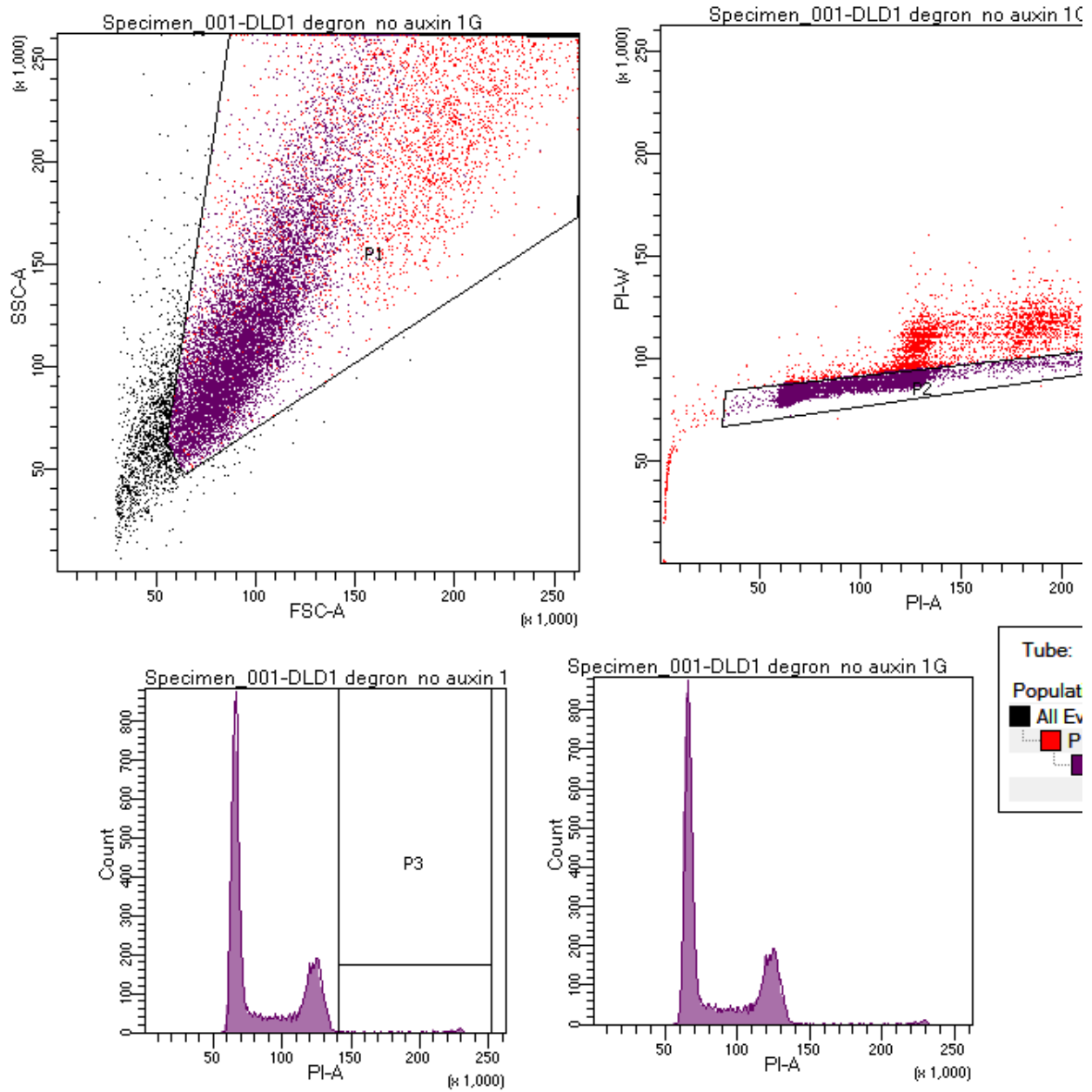

Supplement: Supplementary file 10 — Source data Fig. 5 [file 44318_2025_453_MOESM10_ESM.zip › Figure 5/5B/BLM degrom -IAA.pdf]

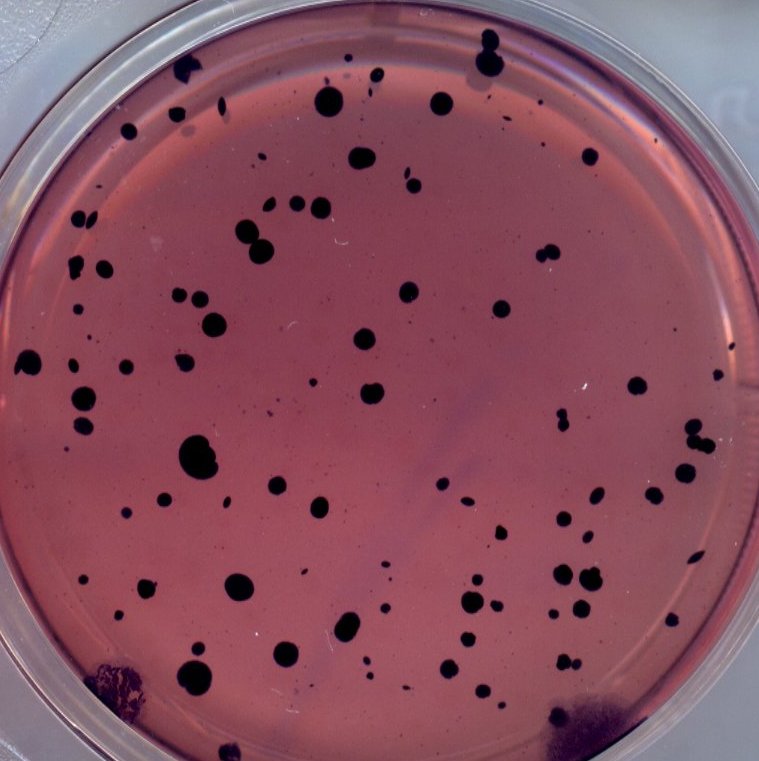

Supplement: Supplementary file 11 — Source data Fig. 6 [file 44318_2025_453_MOESM11_ESM.zip › Figure 6/6B/(BLMmyc53 4N).jpg]

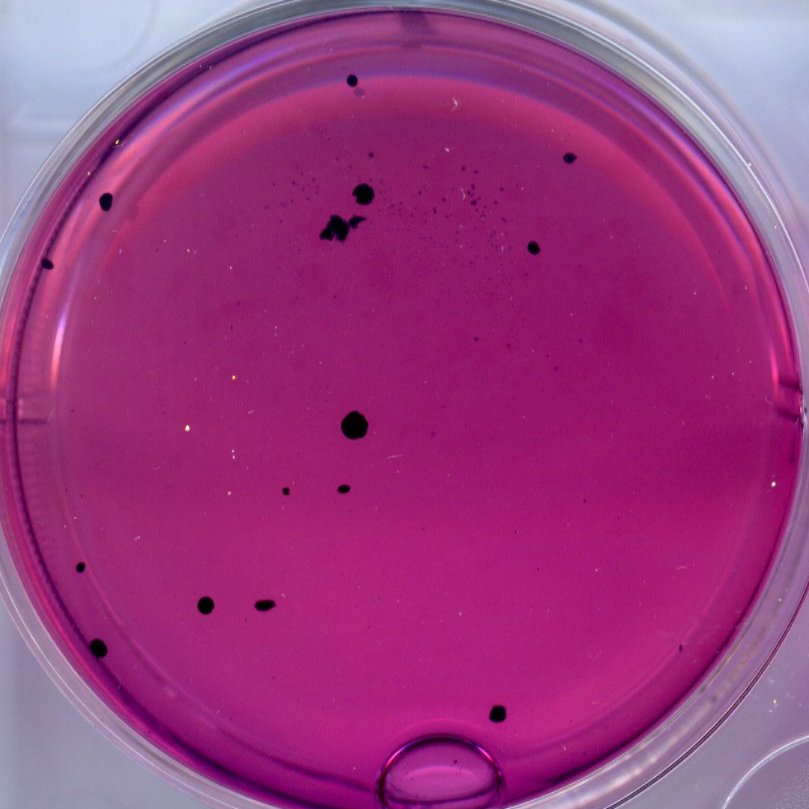

Supplement: Supplementary file 11 — Source data Fig. 6 [file 44318_2025_453_MOESM11_ESM.zip › Figure 6/6B/(RPE53KOMYC 2N).jpg]

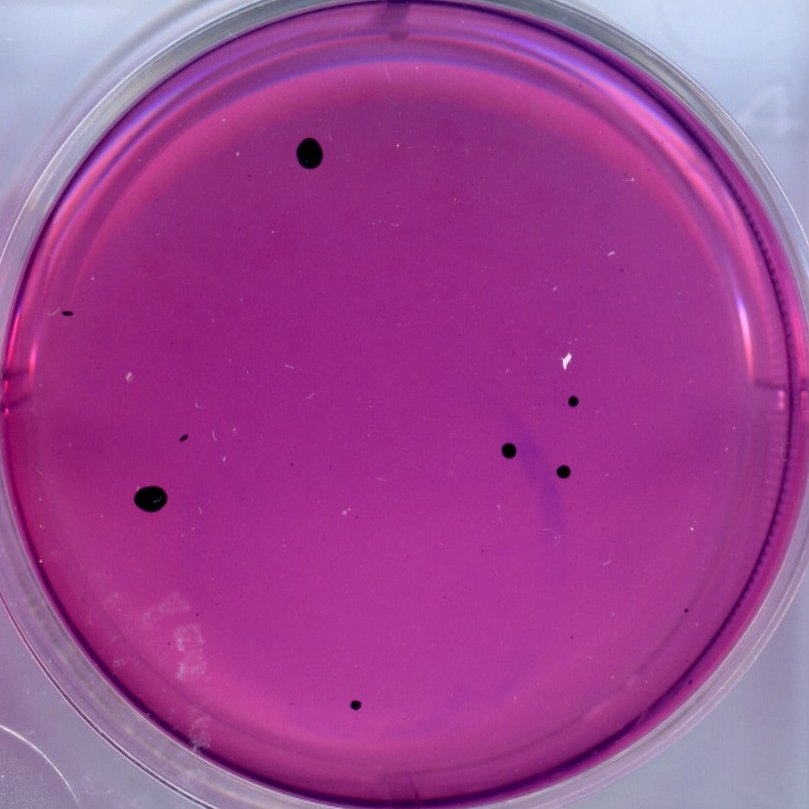

Supplement: Supplementary file 11 — Source data Fig. 6 [file 44318_2025_453_MOESM11_ESM.zip › Figure 6/6B/(RPEBLMKOMYCKO 2N).jpg]

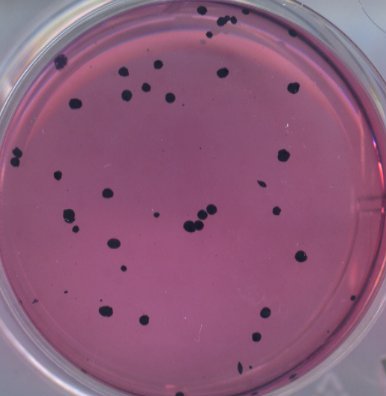

Supplement: Supplementary file 11 — Source data Fig. 6 [file 44318_2025_453_MOESM11_ESM.zip › Figure 6/6B/(Myc 53ko 4N).jpg]

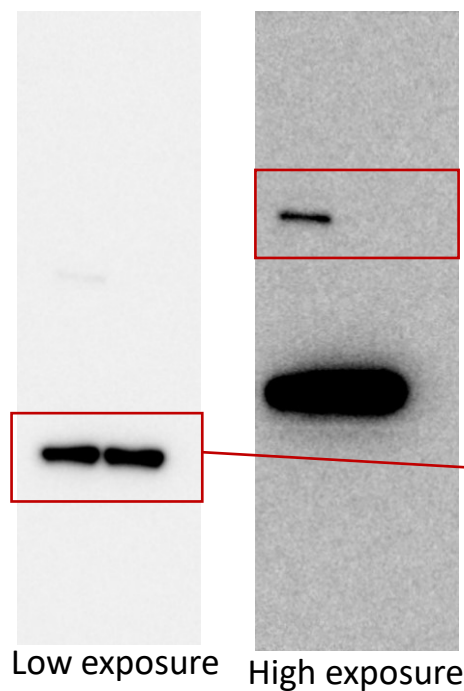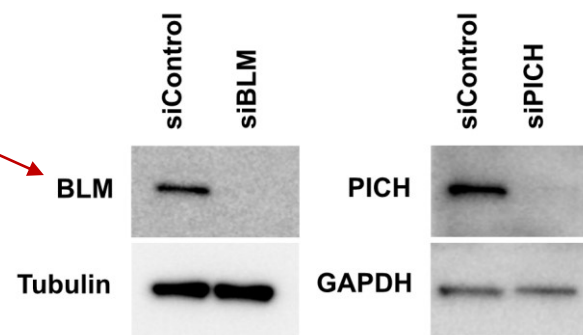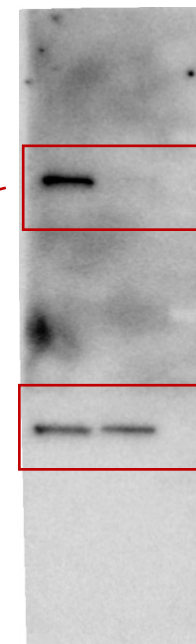

Supplement: Supplementary file 12 — EV Figure Source Data [file 44318_2025_453_MOESM12_ESM.zip › Source data EV-2/Figure EV1/EV1A/EV1A Readme.pdf]

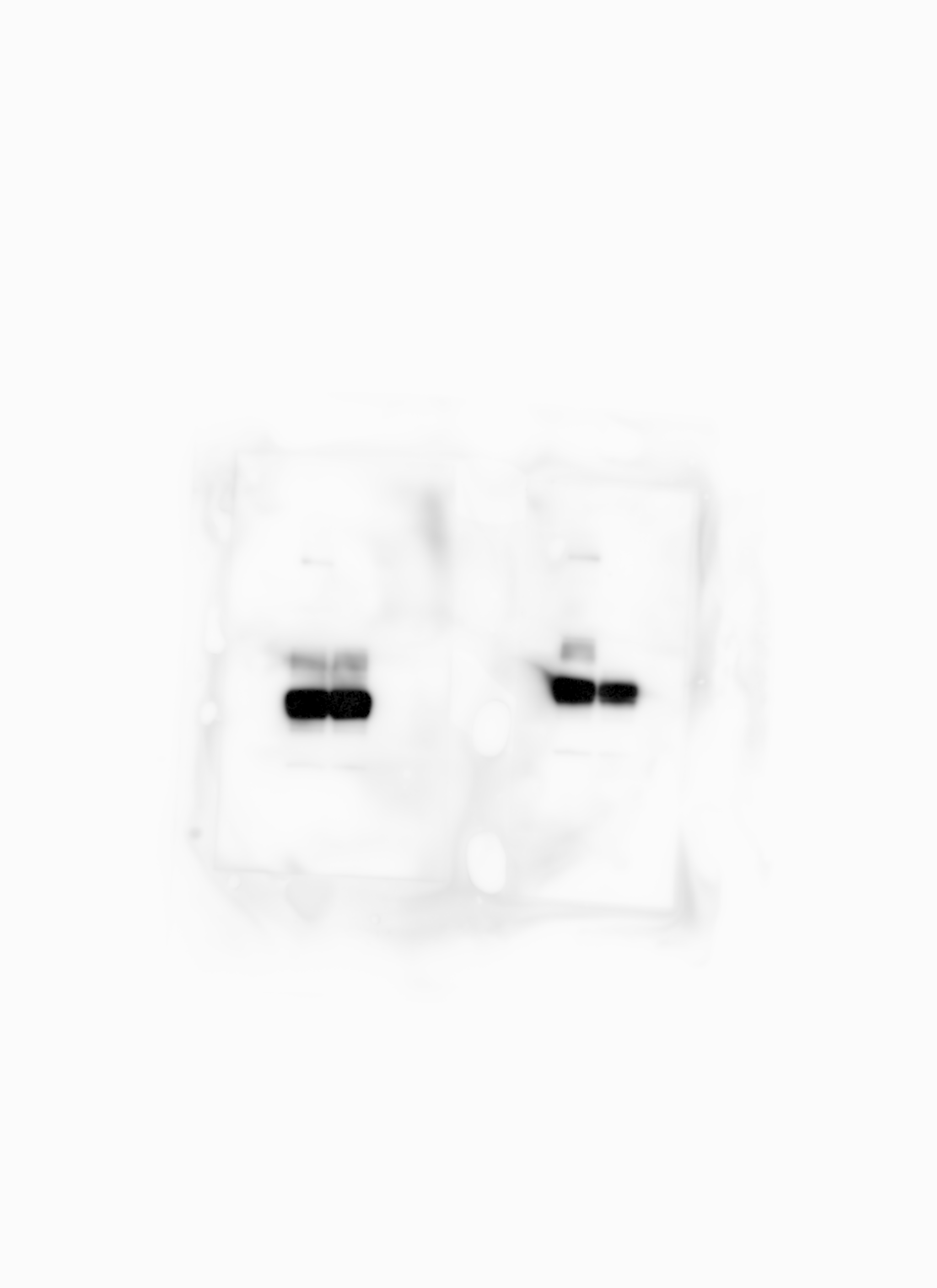

Supplement: Supplementary file 12 — EV Figure Source Data [file 44318_2025_453_MOESM12_ESM.zip › Source data EV-2/Figure EV1/EV1A/REP1/2022.01.14_14.47.10_Ch/2022.01.14_14.47.10_Ch.tif]

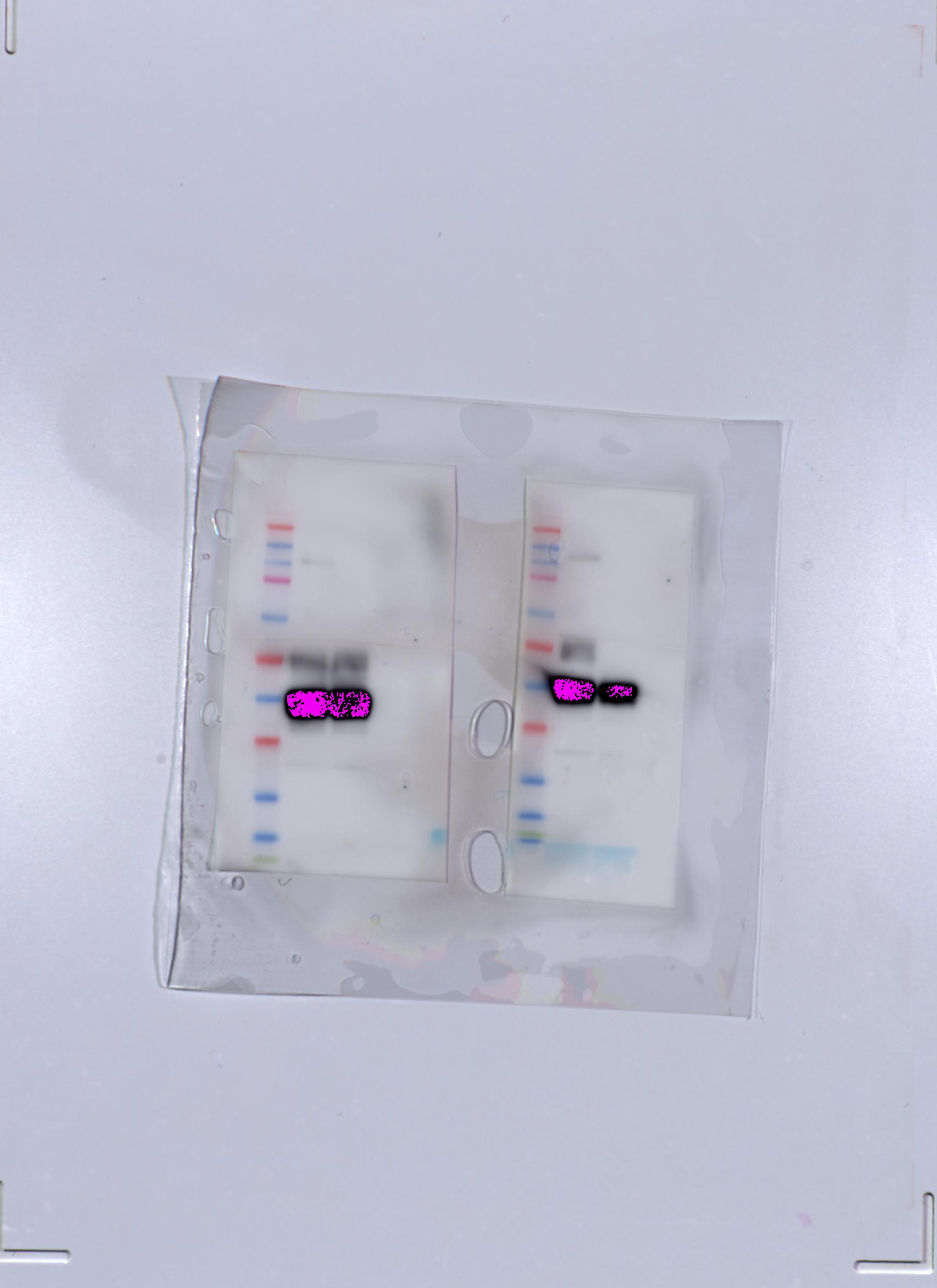

Supplement: Supplementary file 12 — EV Figure Source Data [file 44318_2025_453_MOESM12_ESM.zip › Source data EV-2/Figure EV1/EV1A/REP1/2022.01.14_14.47.10_Ch/2022.01.14_14.47.10_Ch+Marker.jpg]

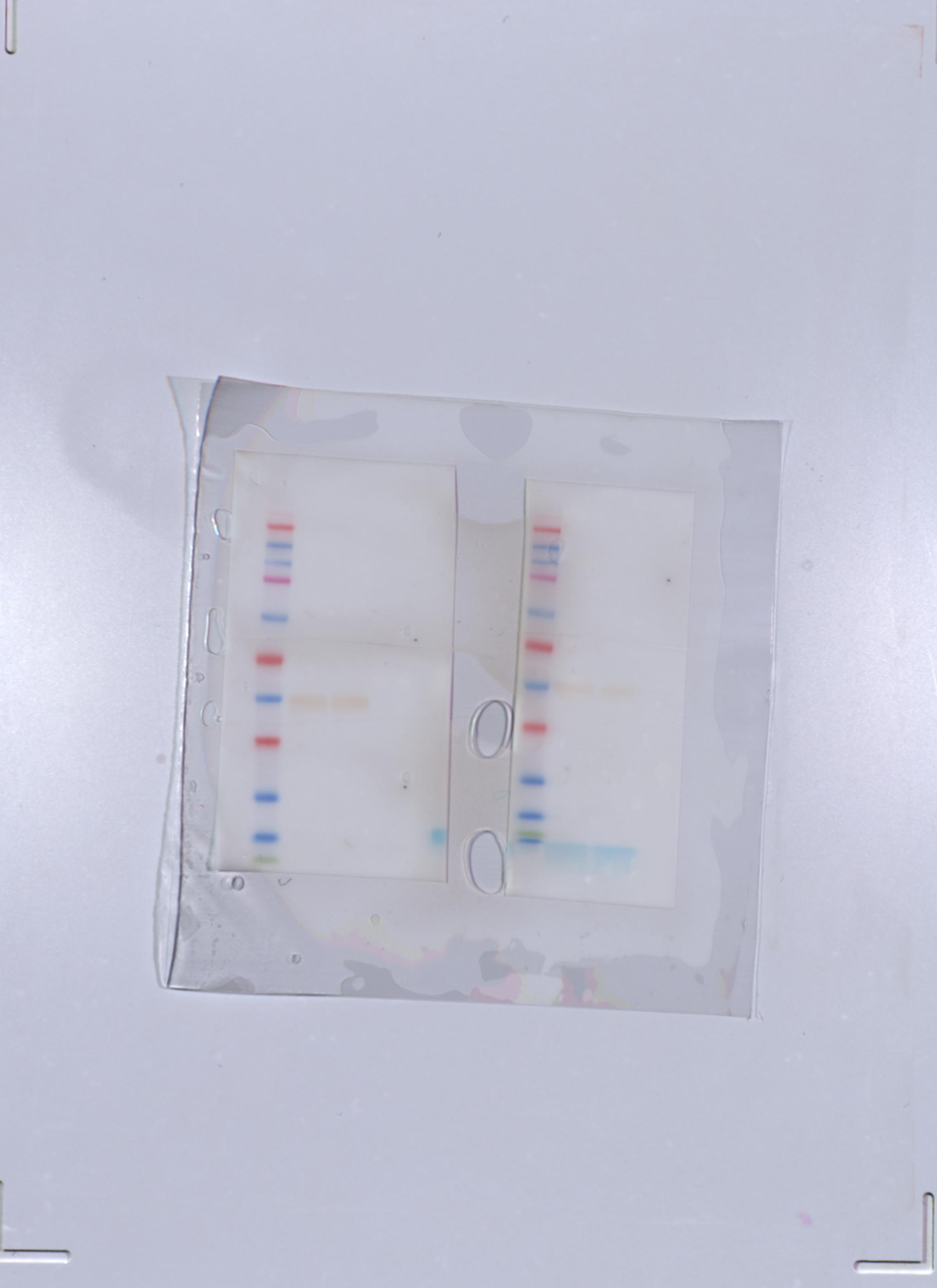

Supplement: Supplementary file 12 — EV Figure Source Data [file 44318_2025_453_MOESM12_ESM.zip › Source data EV-2/Figure EV1/EV1A/REP1/2022.01.14_14.47.10_Ch/2022.01.14_14.47.10_Ch-Marker.jpg]

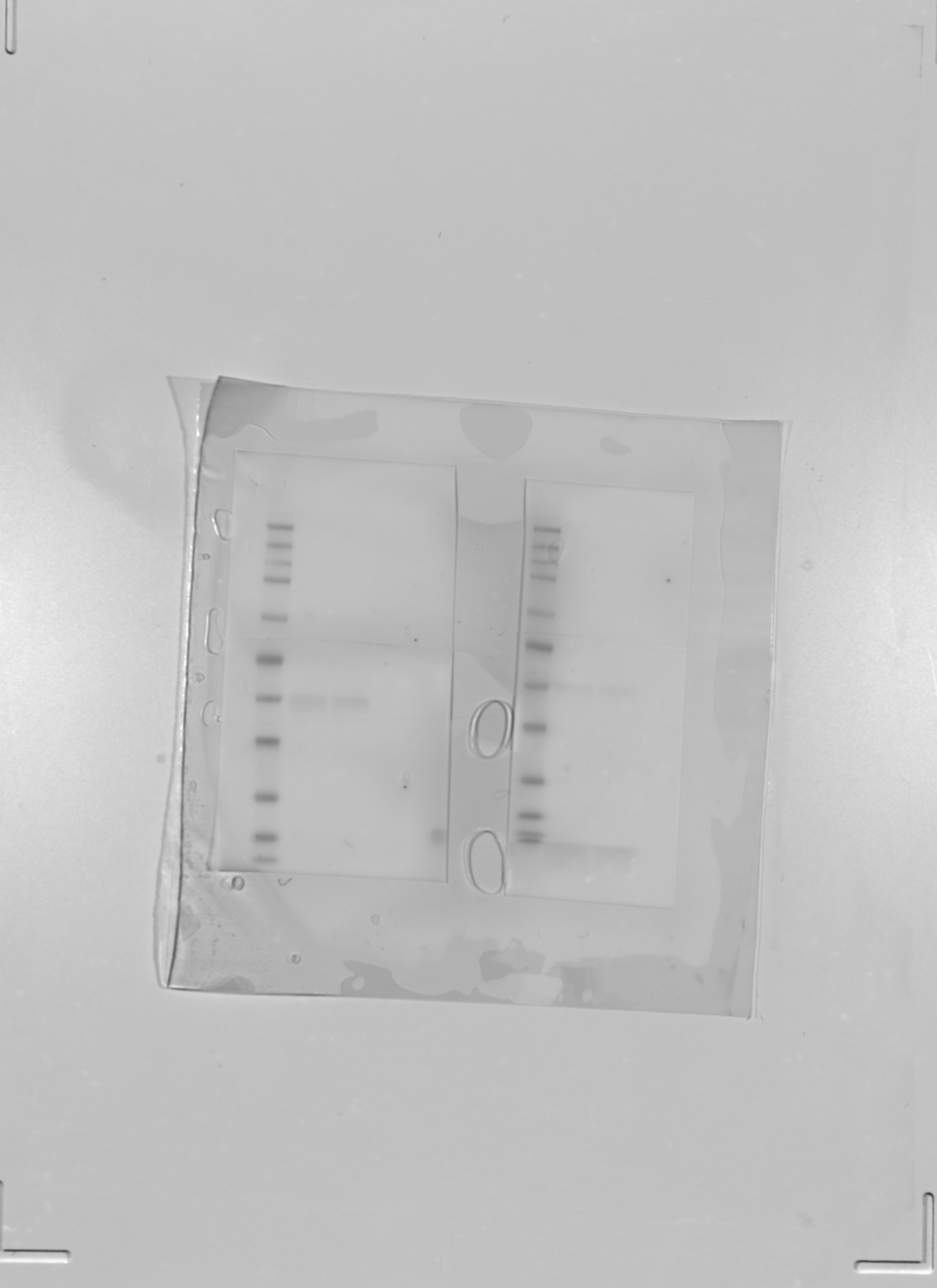

Supplement: Supplementary file 12 — EV Figure Source Data [file 44318_2025_453_MOESM12_ESM.zip › Source data EV-2/Figure EV1/EV1A/REP1/2022.01.14_14.47.10_Ch/2022.01.14_14.47.10_Ch-Marker.tif]

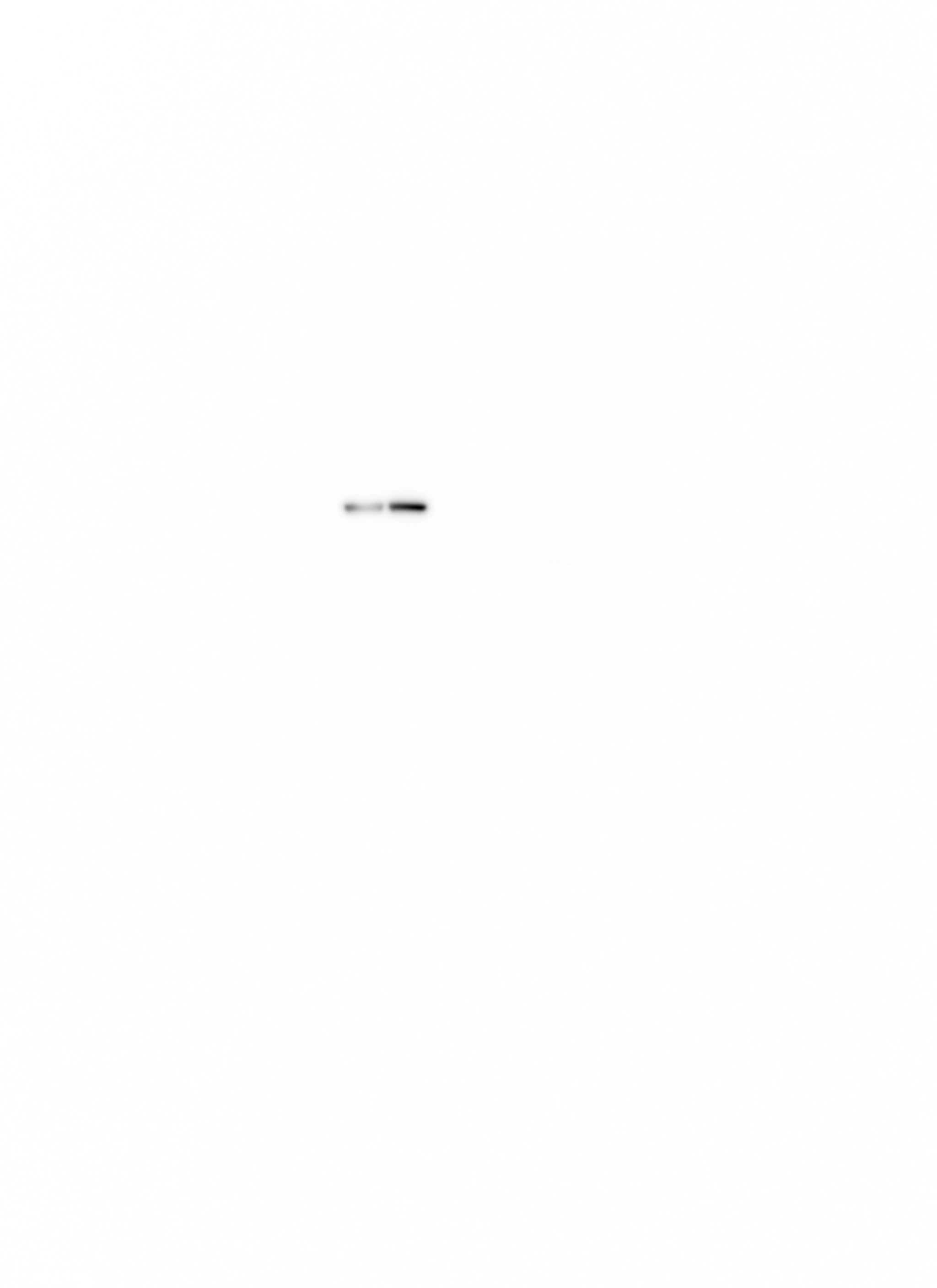

Supplement: Supplementary file 12 — EV Figure Source Data [file 44318_2025_453_MOESM12_ESM.zip › Source data EV-2/Figure EV1/EV1A/REP2/2022.02.20_19.55.27_Ch/2022.02.20_19.55.27_Ch.tif]

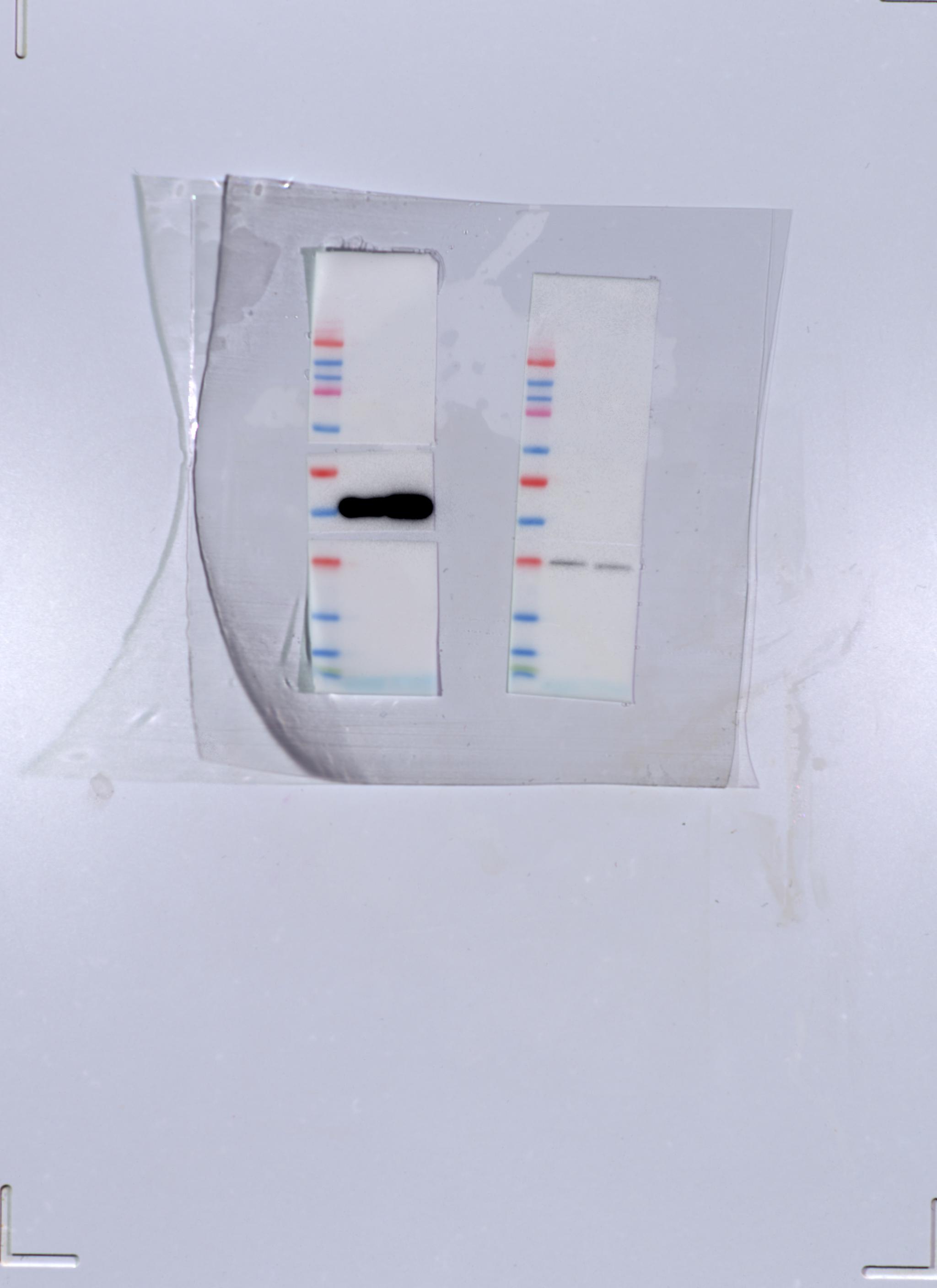

Supplement: Supplementary file 12 — EV Figure Source Data [file 44318_2025_453_MOESM12_ESM.zip › Source data EV-2/Figure EV1/EV1A/REP2/2022.02.20_19.55.27_Ch/2022.02.20_19.55.27_Ch+Marker.jpg]

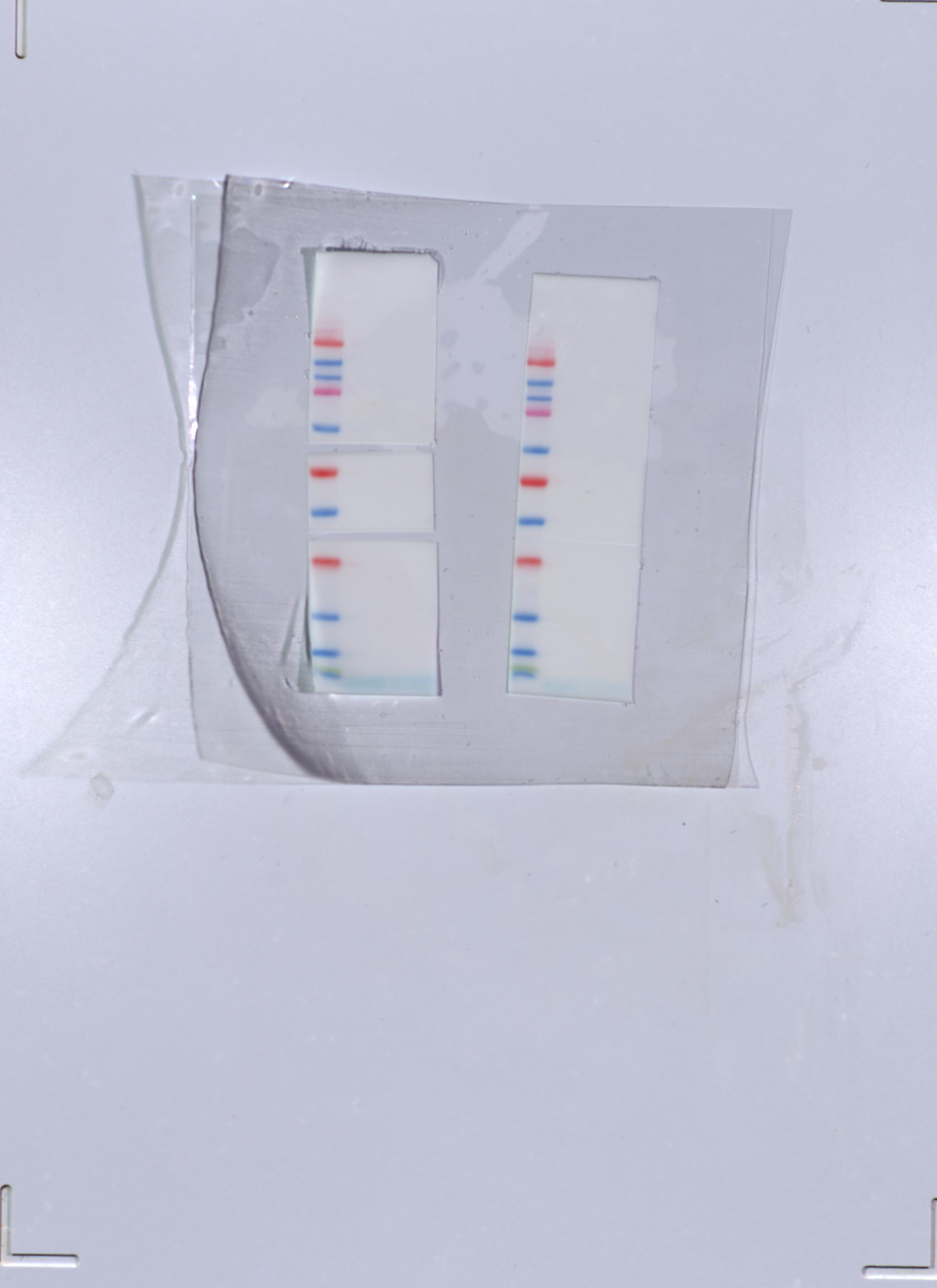

Supplement: Supplementary file 12 — EV Figure Source Data [file 44318_2025_453_MOESM12_ESM.zip › Source data EV-2/Figure EV1/EV1A/REP2/2022.02.20_19.55.27_Ch/2022.02.20_19.55.27_Ch-Marker.jpg]

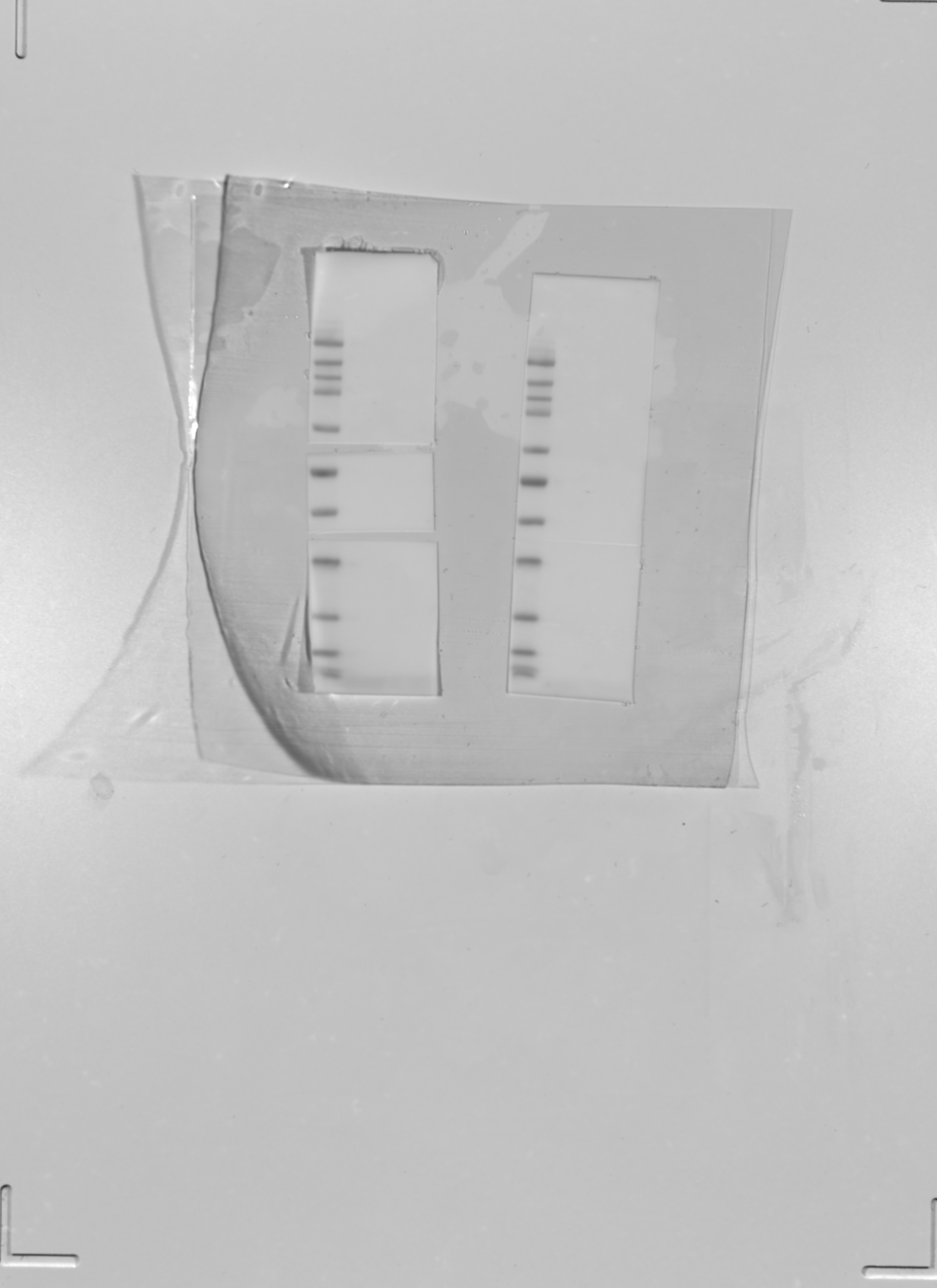

Supplement: Supplementary file 12 — EV Figure Source Data [file 44318_2025_453_MOESM12_ESM.zip › Source data EV-2/Figure EV1/EV1A/REP2/2022.02.20_19.55.27_Ch/2022.02.20_19.55.27_Ch-Marker.tif]

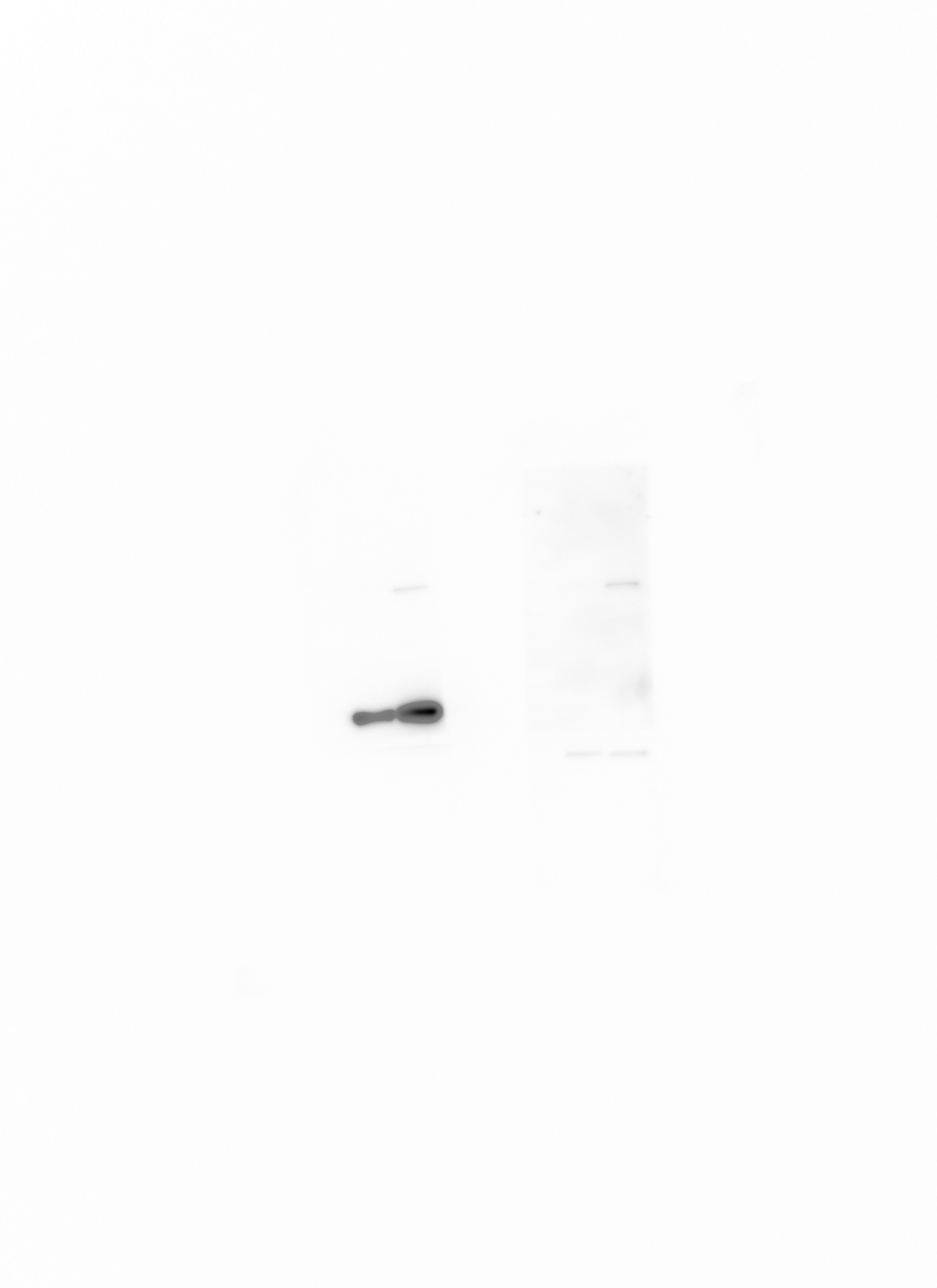

Supplement: Supplementary file 12 — EV Figure Source Data [file 44318_2025_453_MOESM12_ESM.zip › Source data EV-2/Figure EV1/EV1A/REP2/2022.02.20_19.59.18_Ch/2022.02.20_19.59.18_Ch.tif]

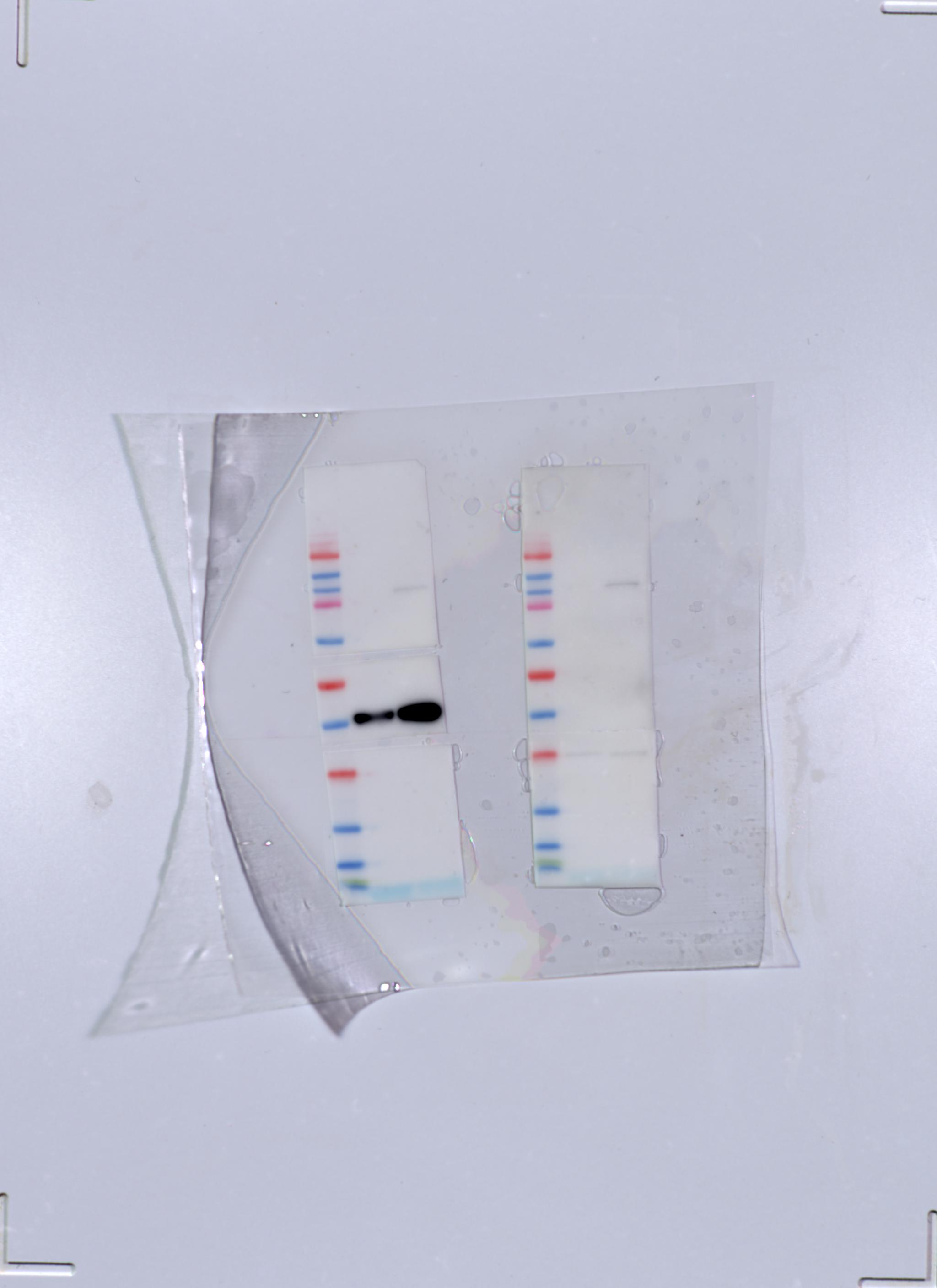

Supplement: Supplementary file 12 — EV Figure Source Data [file 44318_2025_453_MOESM12_ESM.zip › Source data EV-2/Figure EV1/EV1A/REP2/2022.02.20_19.59.18_Ch/2022.02.20_19.59.18_Ch+Marker.jpg]

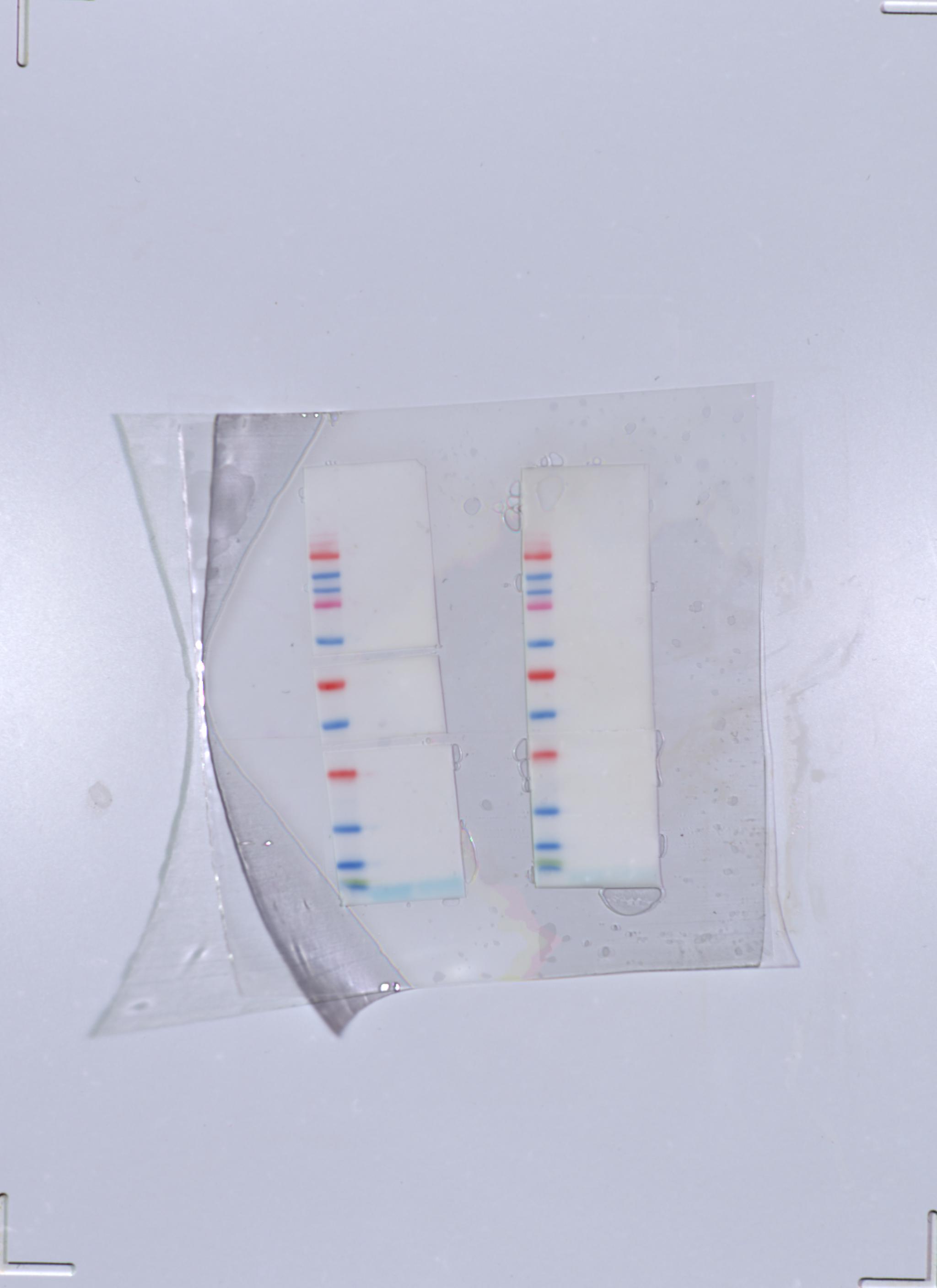

Supplement: Supplementary file 12 — EV Figure Source Data [file 44318_2025_453_MOESM12_ESM.zip › Source data EV-2/Figure EV1/EV1A/REP2/2022.02.20_19.59.18_Ch/2022.02.20_19.59.18_Ch-Marker.jpg]

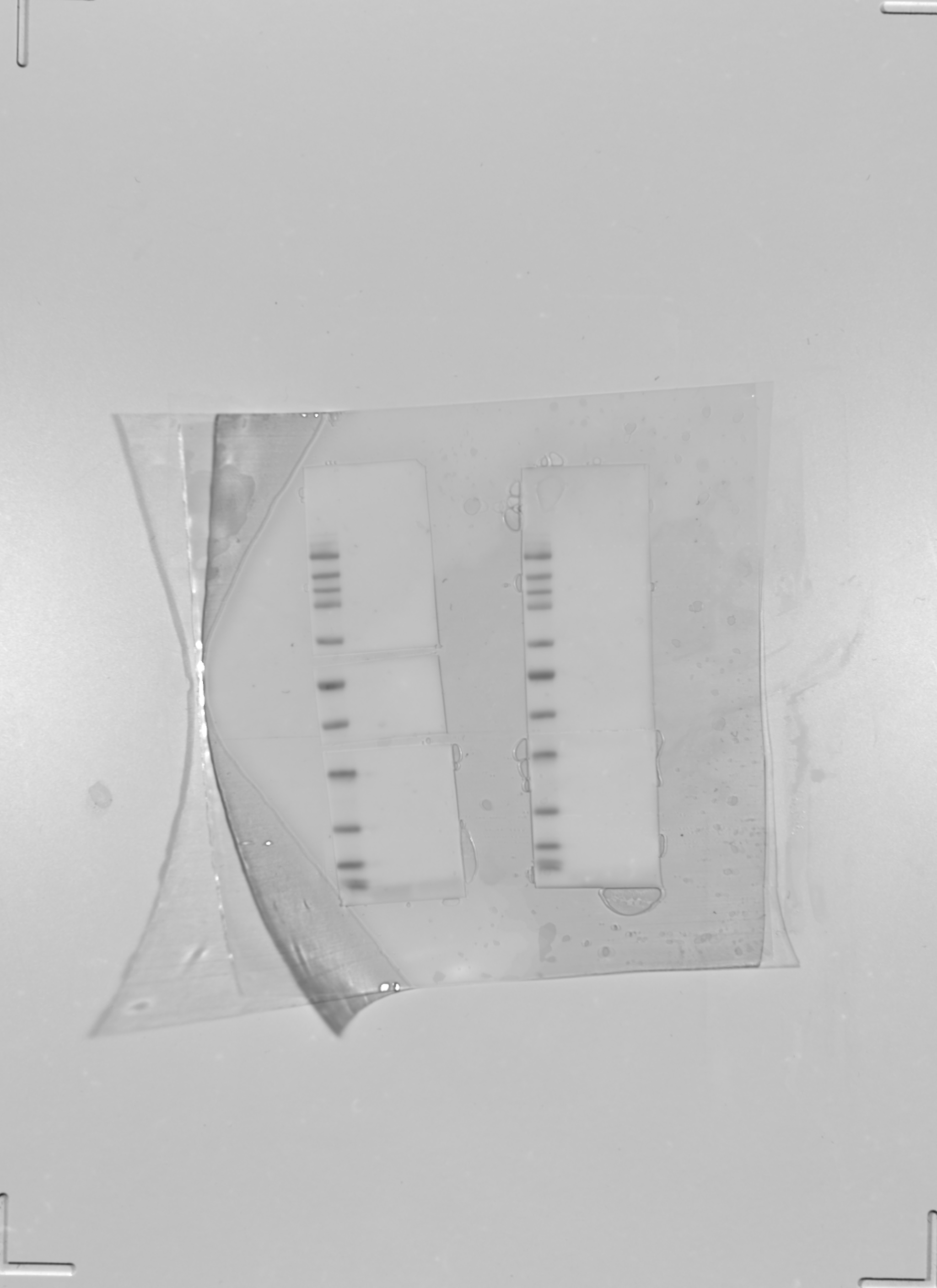

Supplement: Supplementary file 12 — EV Figure Source Data [file 44318_2025_453_MOESM12_ESM.zip › Source data EV-2/Figure EV1/EV1A/REP2/2022.02.20_19.59.18_Ch/2022.02.20_19.59.18_Ch-Marker.tif]

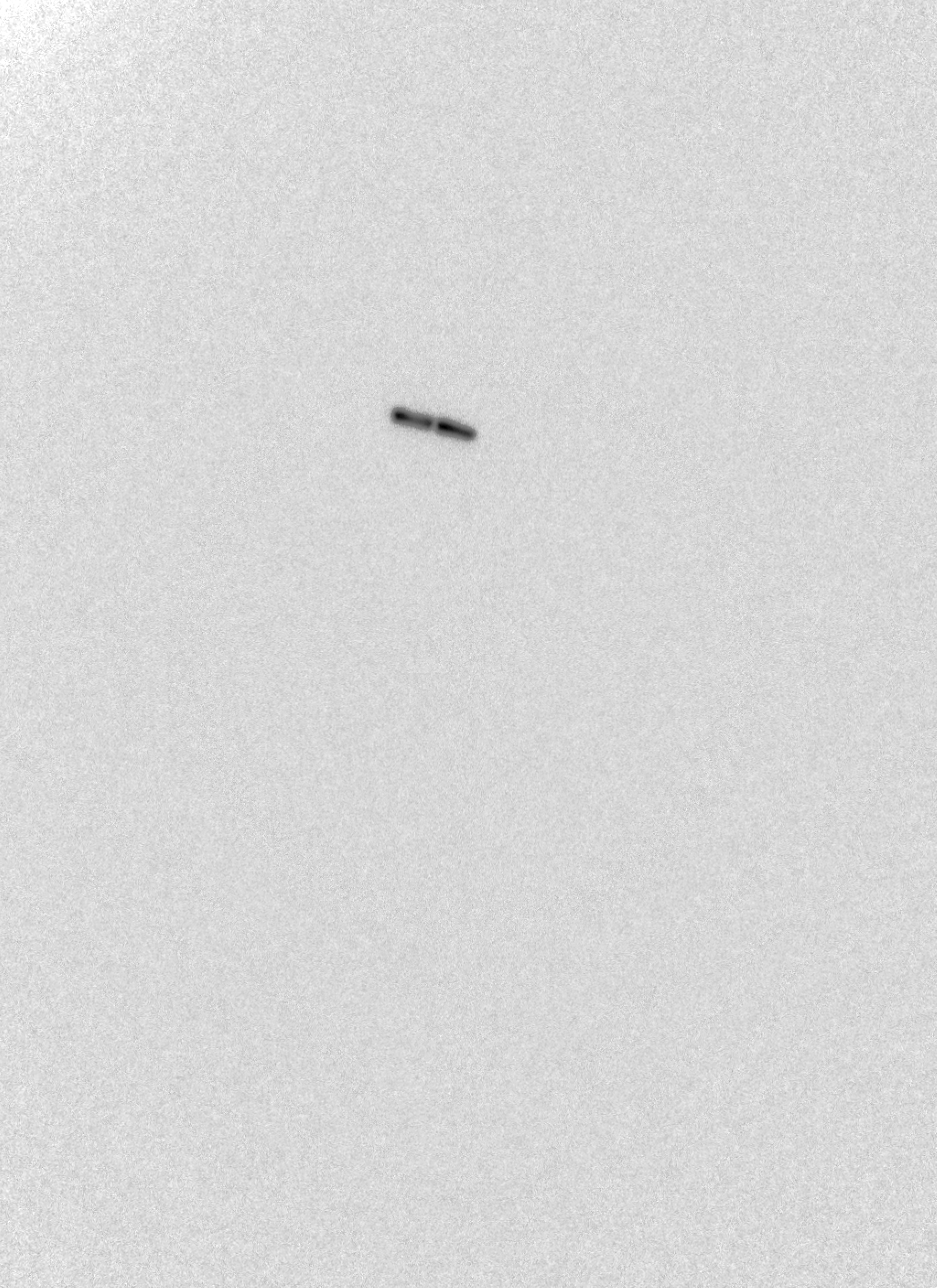

Supplement: Supplementary file 12 — EV Figure Source Data [file 44318_2025_453_MOESM12_ESM.zip › Source data EV-2/Figure EV1/EV1A/REP2/2022.02.21_14.12.59_Ch/2022.02.21_14.12.59_Ch.tif]

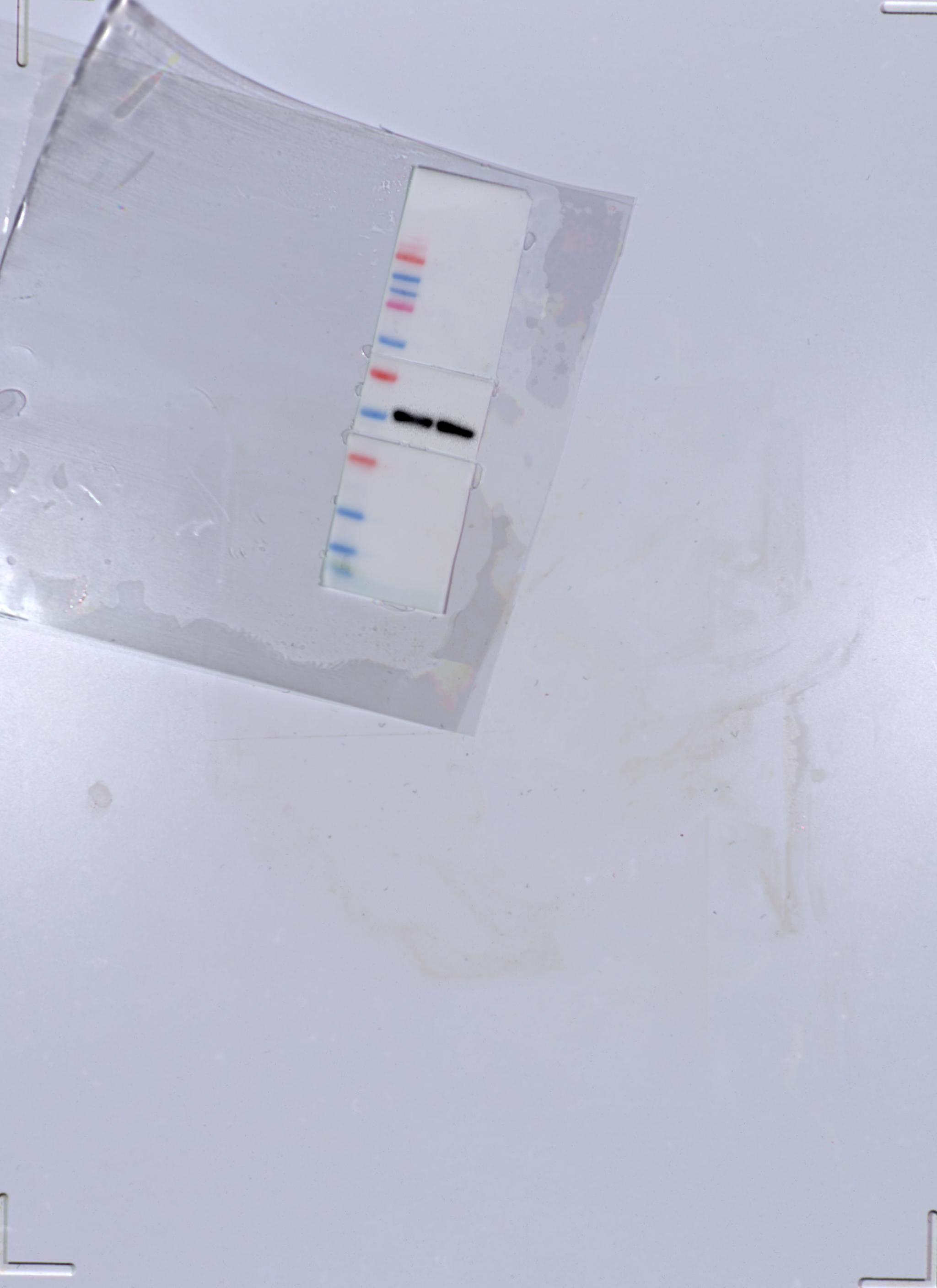

Supplement: Supplementary file 12 — EV Figure Source Data [file 44318_2025_453_MOESM12_ESM.zip › Source data EV-2/Figure EV1/EV1A/REP2/2022.02.21_14.12.59_Ch/2022.02.21_14.12.59_Ch+Marker.jpg]

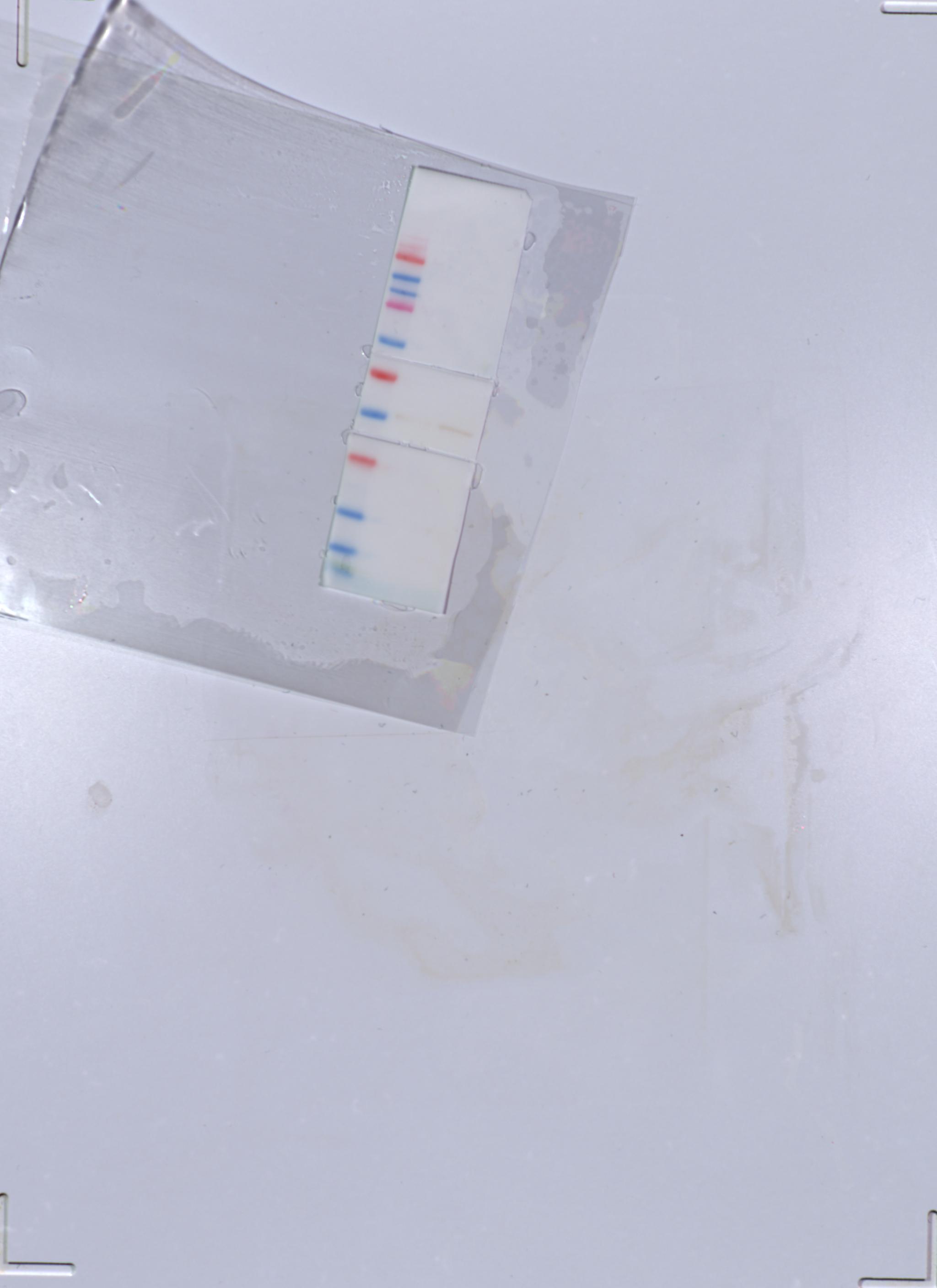

Supplement: Supplementary file 12 — EV Figure Source Data [file 44318_2025_453_MOESM12_ESM.zip › Source data EV-2/Figure EV1/EV1A/REP2/2022.02.21_14.12.59_Ch/2022.02.21_14.12.59_Ch-Marker.jpg]

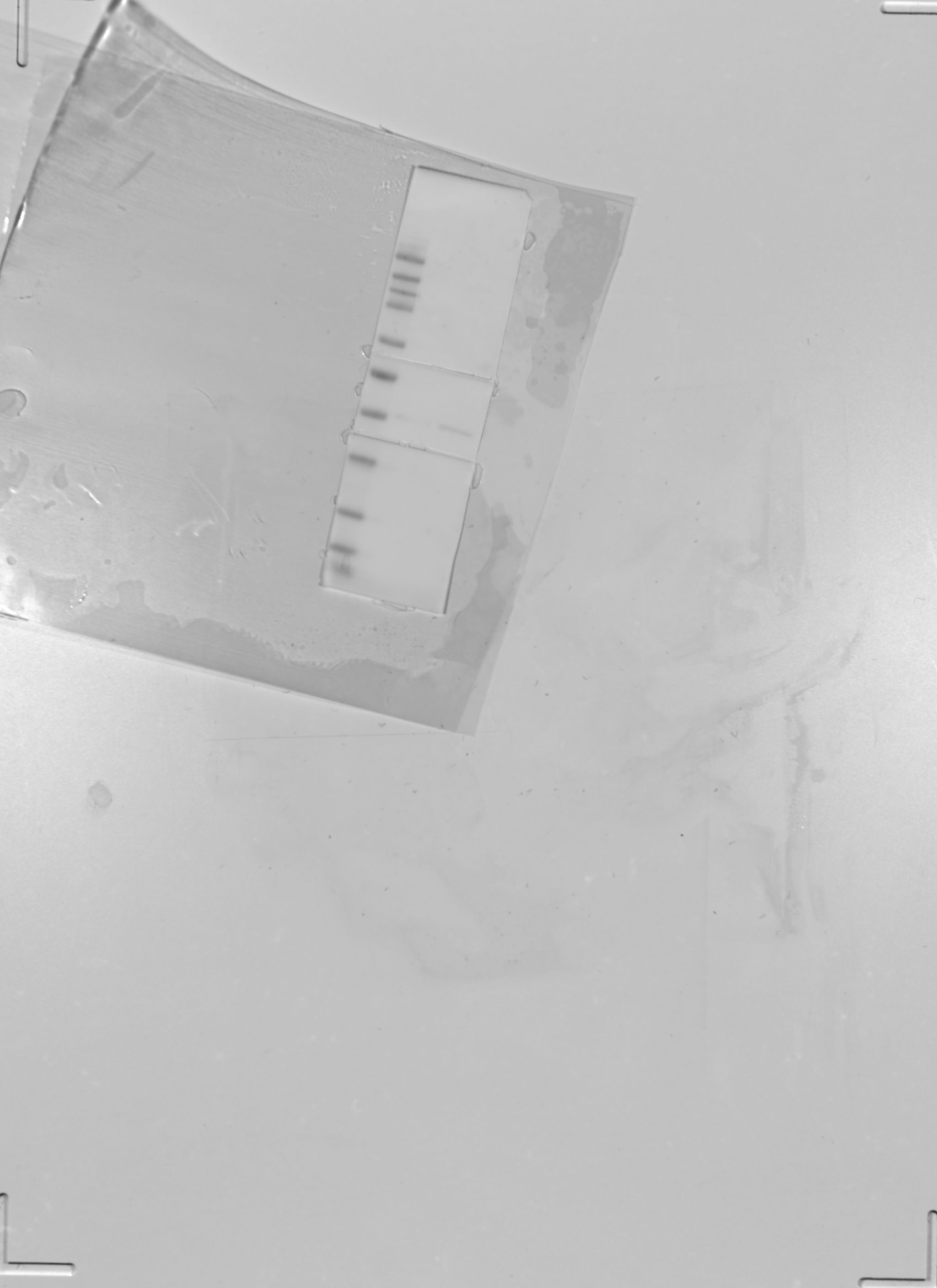

Supplement: Supplementary file 12 — EV Figure Source Data [file 44318_2025_453_MOESM12_ESM.zip › Source data EV-2/Figure EV1/EV1A/REP2/2022.02.21_14.12.59_Ch/2022.02.21_14.12.59_Ch-Marker.tif]

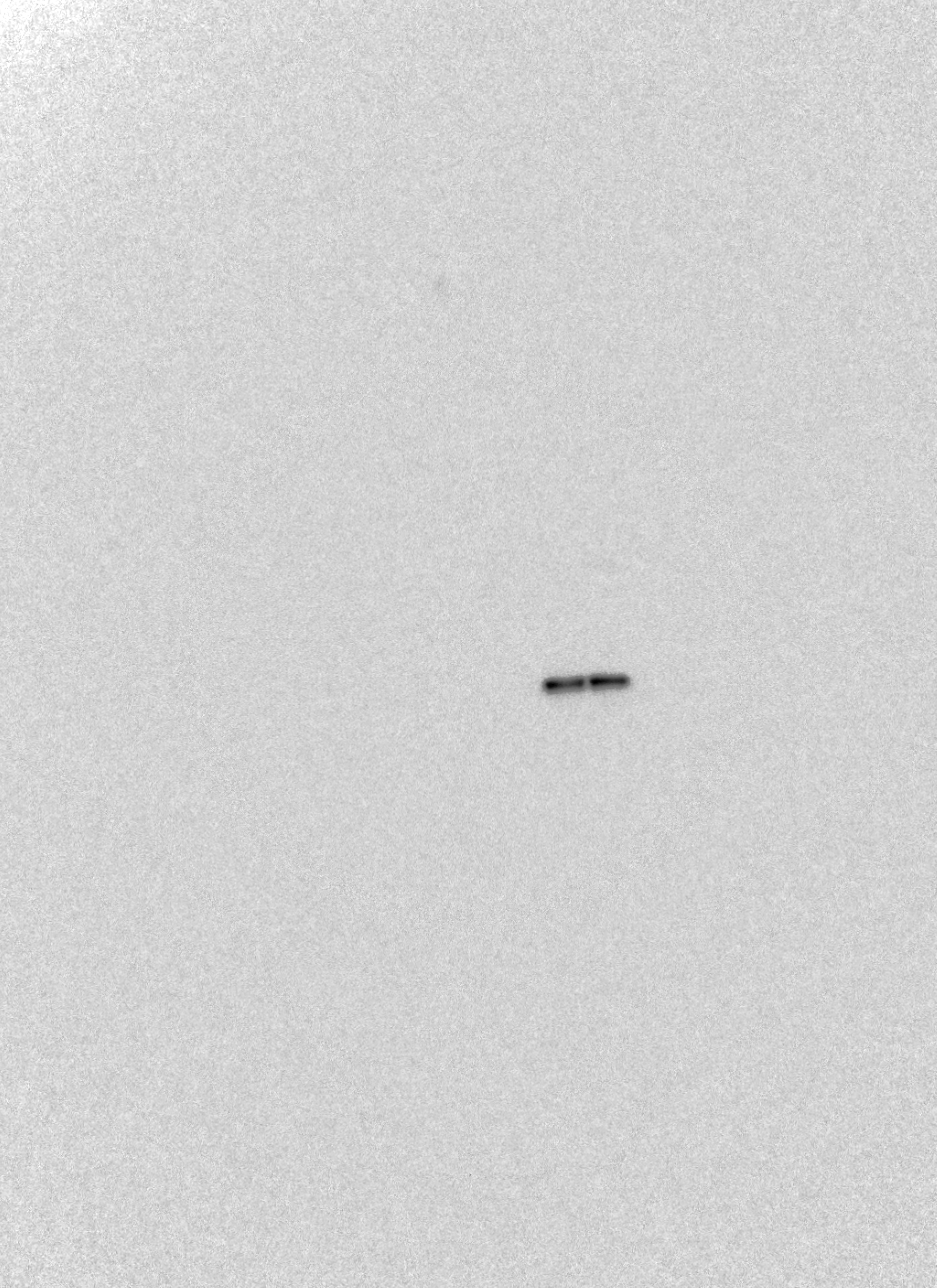

Supplement: Supplementary file 12 — EV Figure Source Data [file 44318_2025_453_MOESM12_ESM.zip › Source data EV-2/Figure EV1/EV1A/REP2/2022.02.21_14.15.26_Ch/2022.02.21_14.15.26_Ch.tif]

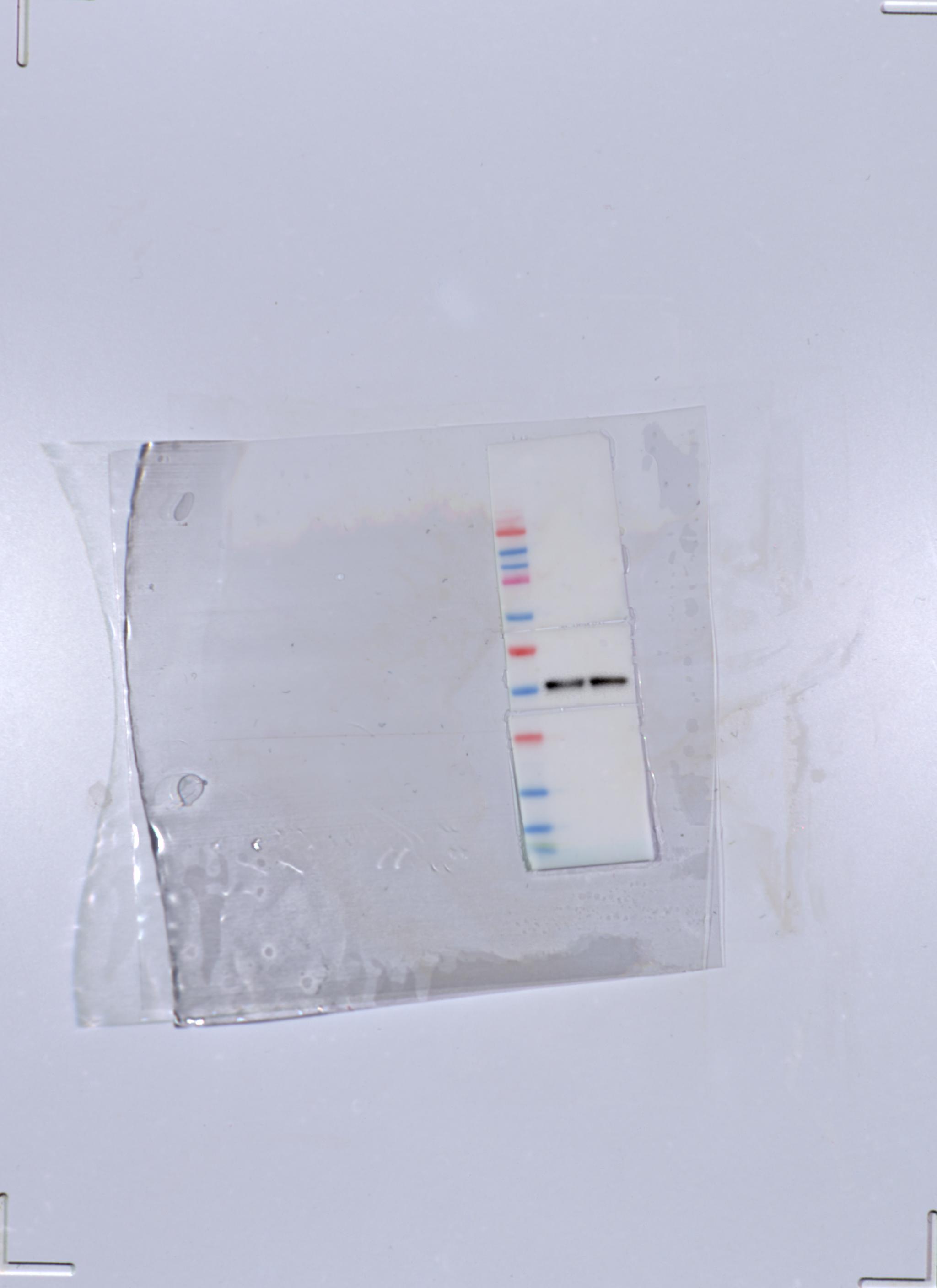

Supplement: Supplementary file 12 — EV Figure Source Data [file 44318_2025_453_MOESM12_ESM.zip › Source data EV-2/Figure EV1/EV1A/REP2/2022.02.21_14.15.26_Ch/2022.02.21_14.15.26_Ch+Marker.jpg]

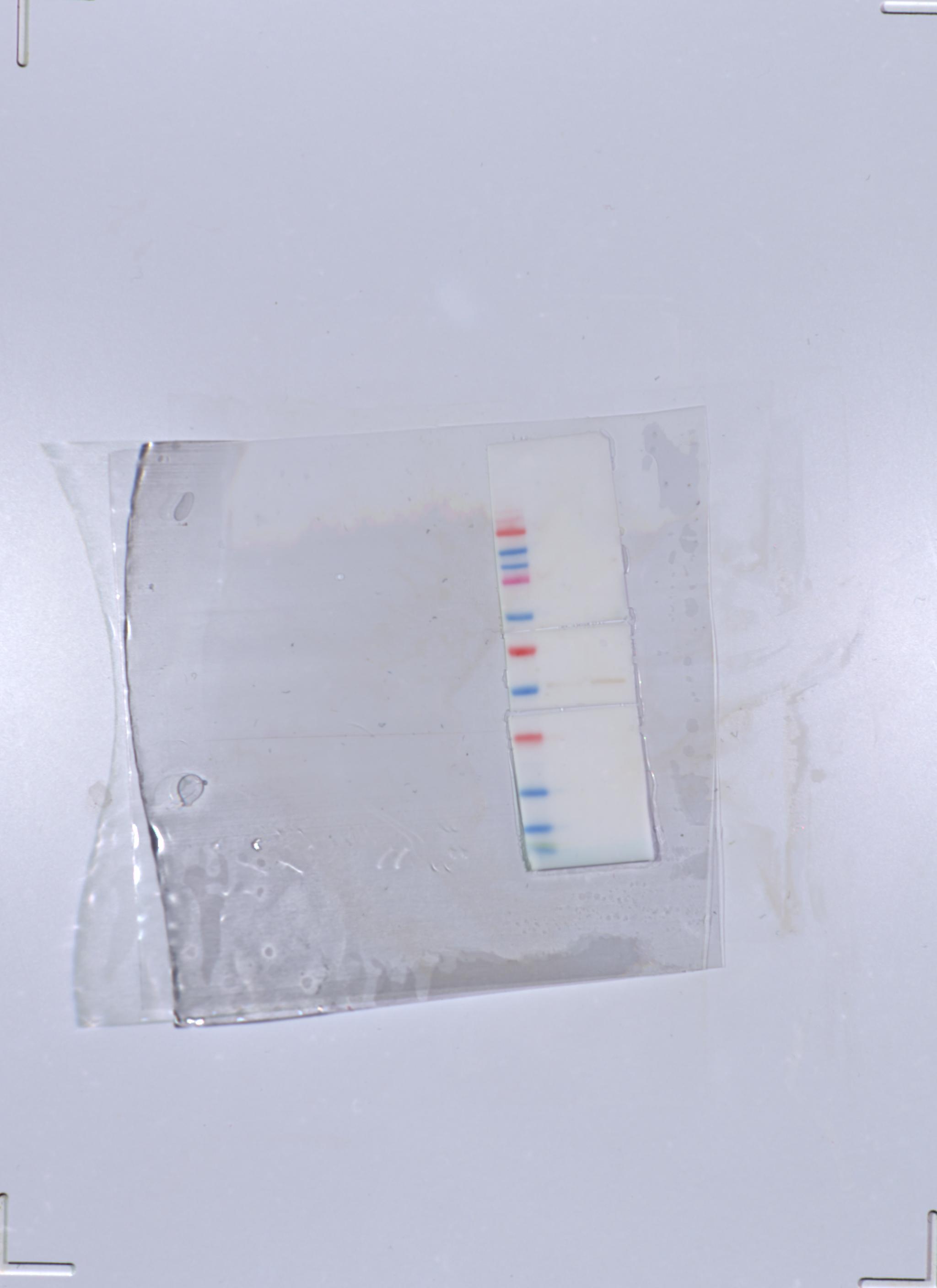

Supplement: Supplementary file 12 — EV Figure Source Data [file 44318_2025_453_MOESM12_ESM.zip › Source data EV-2/Figure EV1/EV1A/REP2/2022.02.21_14.15.26_Ch/2022.02.21_14.15.26_Ch-Marker.jpg]

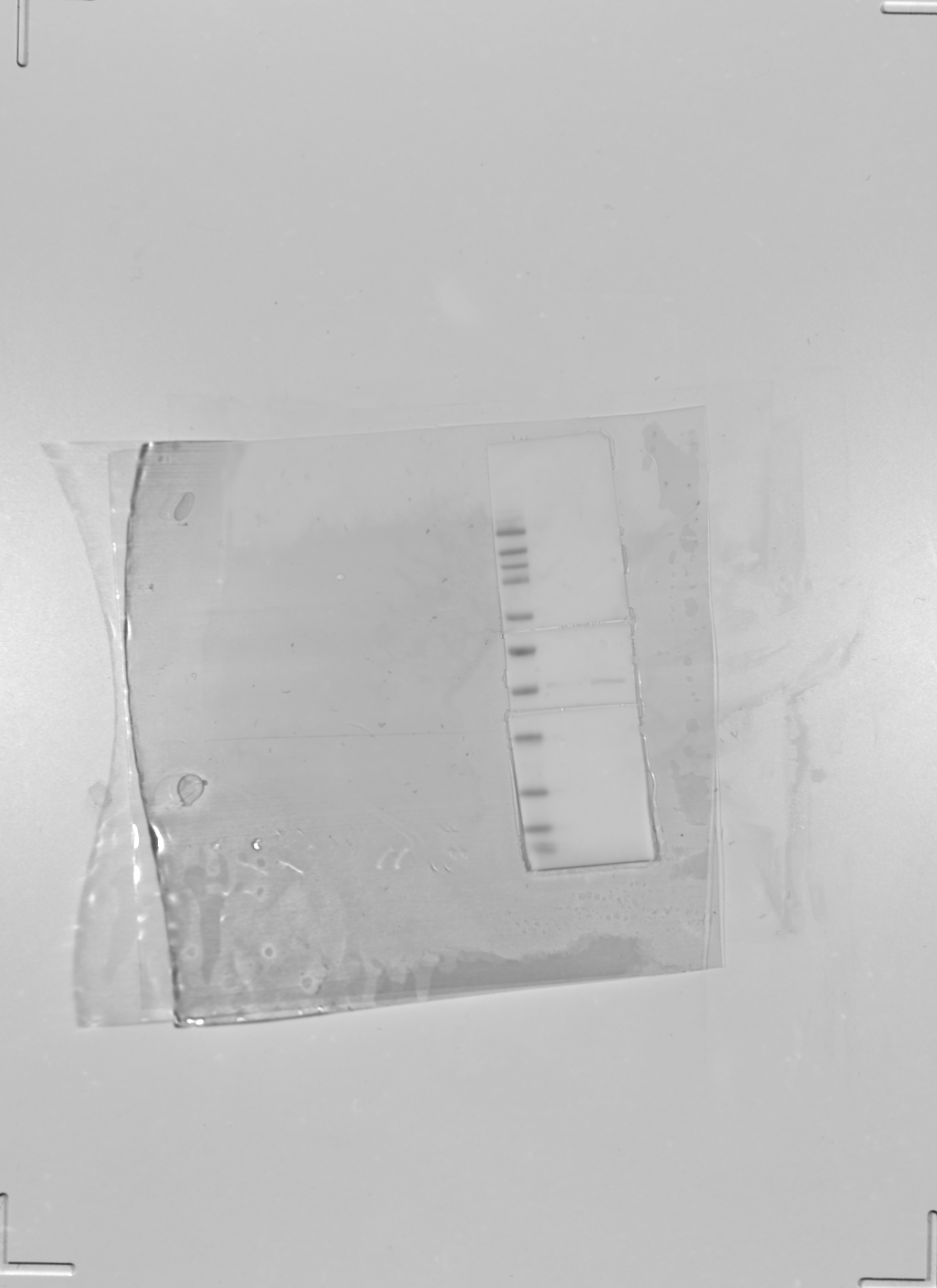

Supplement: Supplementary file 12 — EV Figure Source Data [file 44318_2025_453_MOESM12_ESM.zip › Source data EV-2/Figure EV1/EV1A/REP2/2022.02.21_14.15.26_Ch/2022.02.21_14.15.26_Ch-Marker.tif]

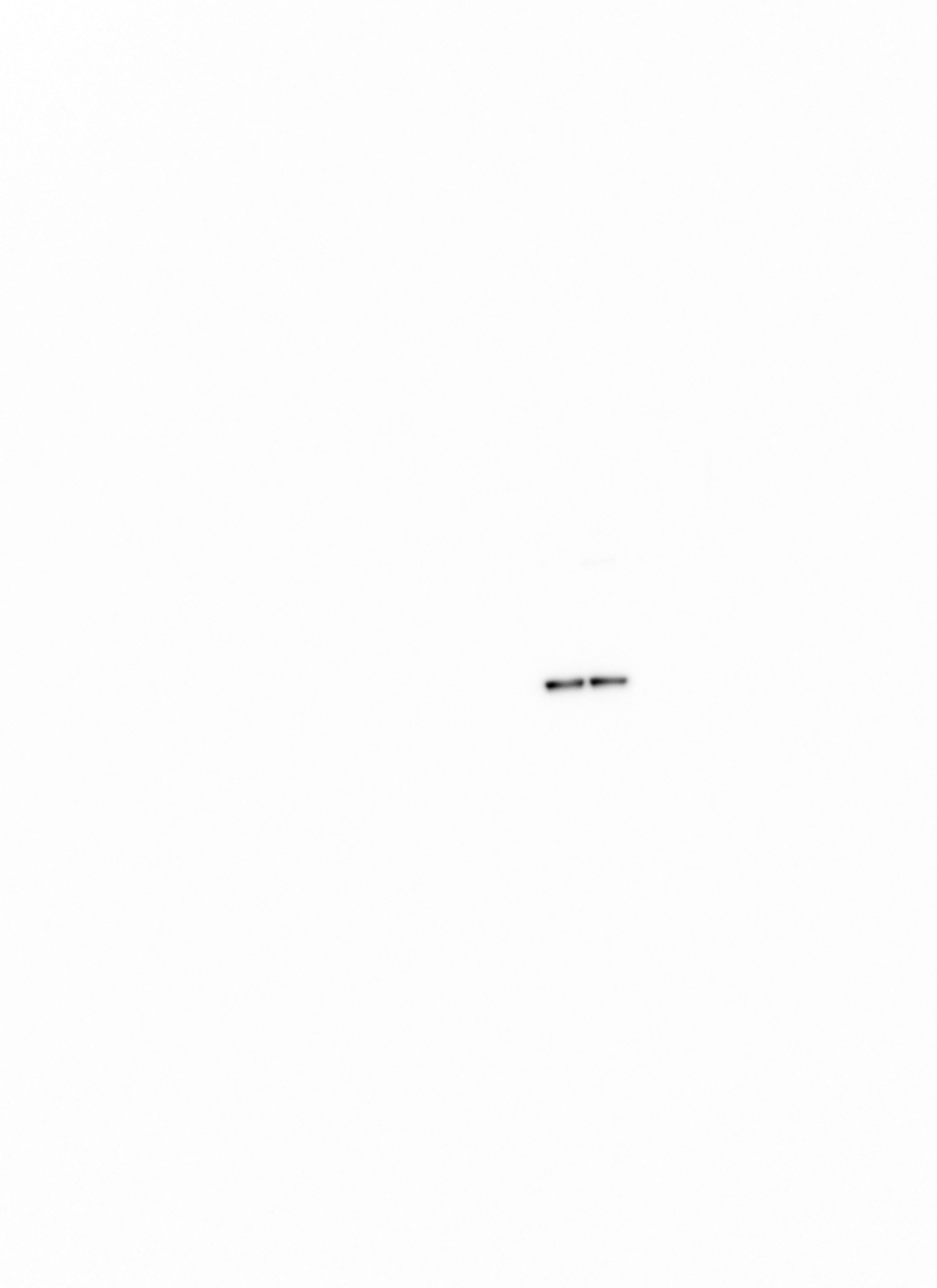

Supplement: Supplementary file 12 — EV Figure Source Data [file 44318_2025_453_MOESM12_ESM.zip › Source data EV-2/Figure EV1/EV1A/REP2/2022.02.21_14.17.12_Ch/2022.02.21_14.17.12_Ch.tif]

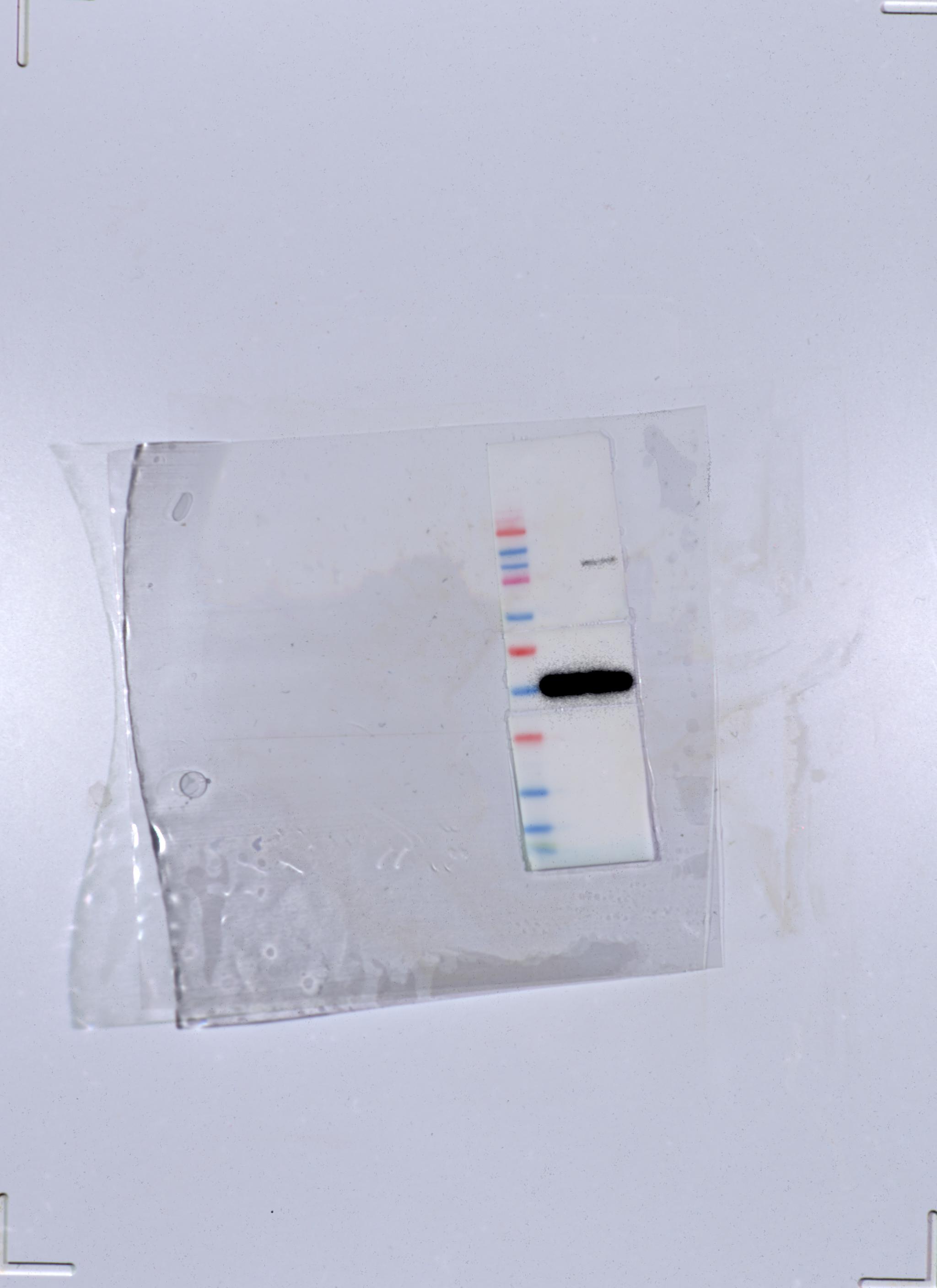

Supplement: Supplementary file 12 — EV Figure Source Data [file 44318_2025_453_MOESM12_ESM.zip › Source data EV-2/Figure EV1/EV1A/REP2/2022.02.21_14.17.12_Ch/2022.02.21_14.17.12_Ch+Marker.jpg]

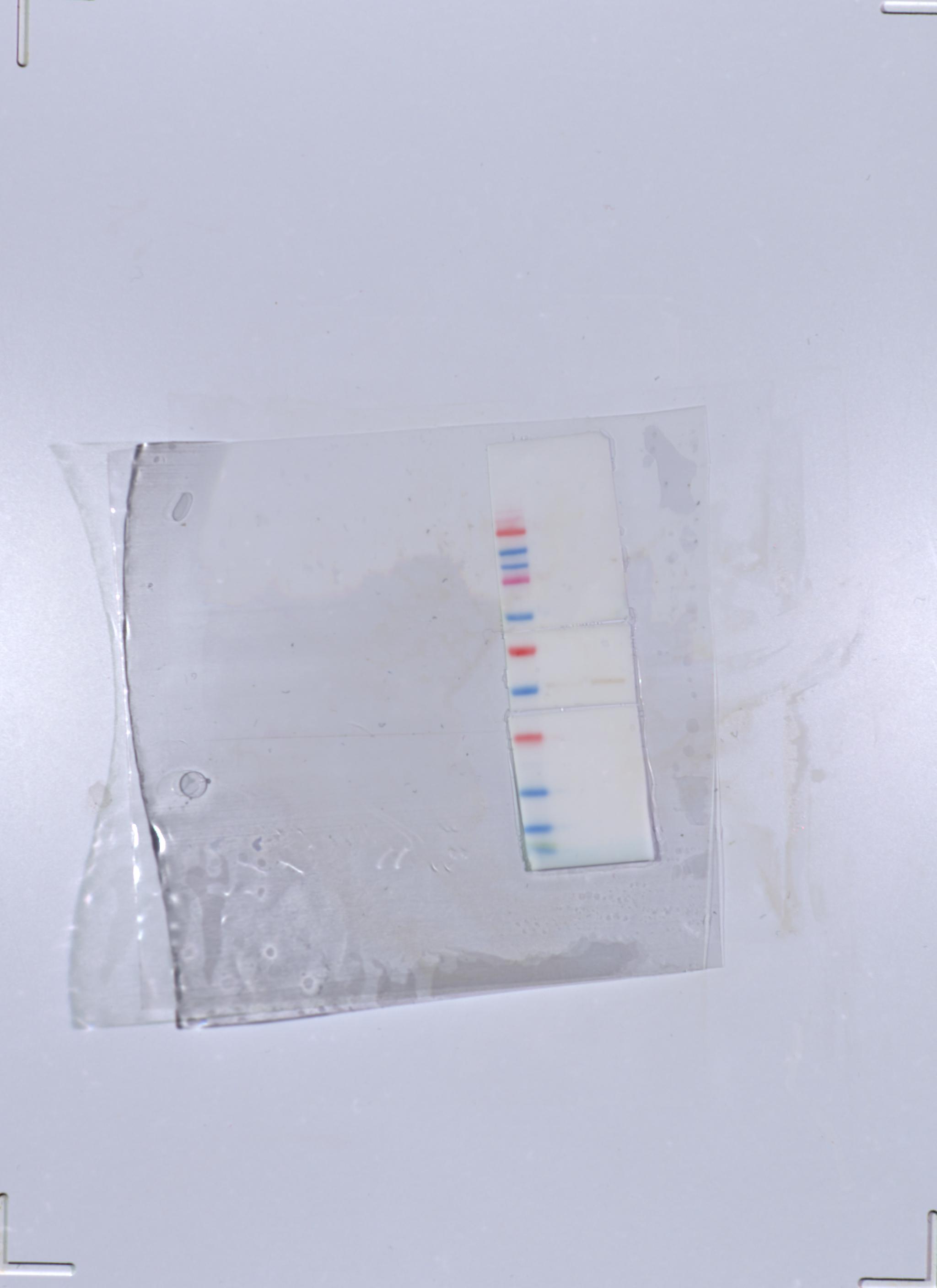

Supplement: Supplementary file 12 — EV Figure Source Data [file 44318_2025_453_MOESM12_ESM.zip › Source data EV-2/Figure EV1/EV1A/REP2/2022.02.21_14.17.12_Ch/2022.02.21_14.17.12_Ch-Marker.jpg]

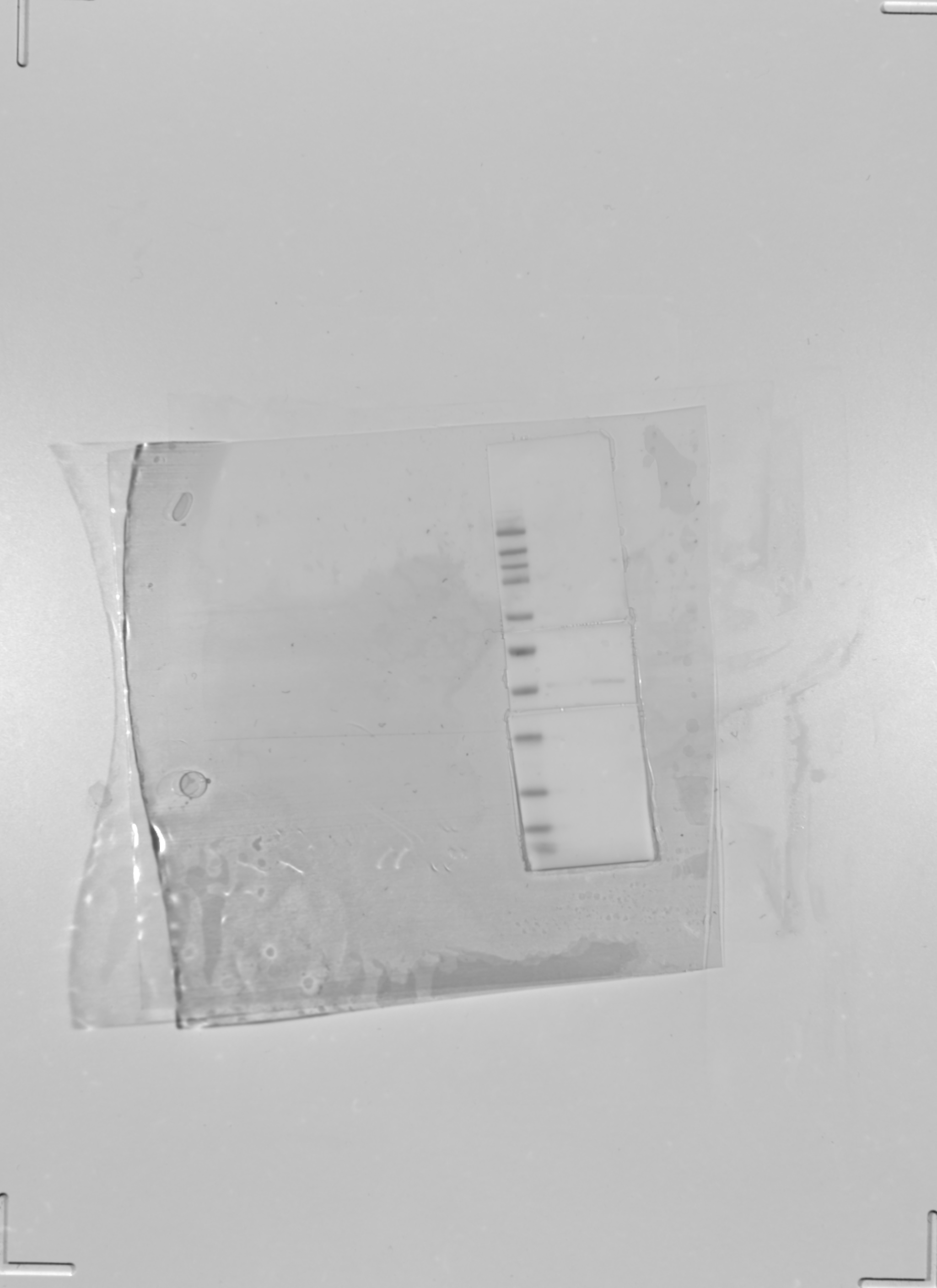

Supplement: Supplementary file 12 — EV Figure Source Data [file 44318_2025_453_MOESM12_ESM.zip › Source data EV-2/Figure EV1/EV1A/REP2/2022.02.21_14.17.12_Ch/2022.02.21_14.17.12_Ch-Marker.tif]

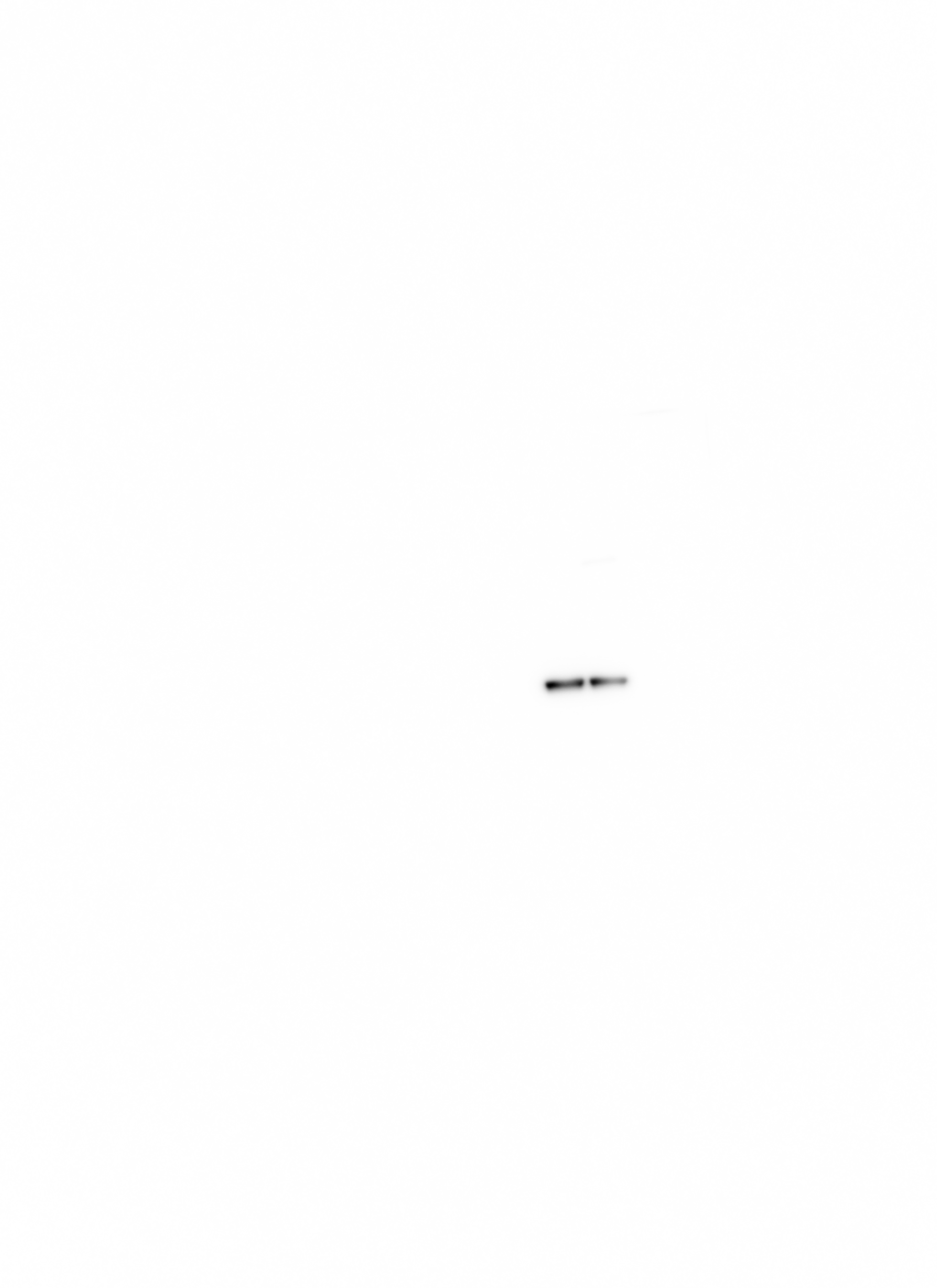

Supplement: Supplementary file 12 — EV Figure Source Data [file 44318_2025_453_MOESM12_ESM.zip › Source data EV-2/Figure EV1/EV1A/REP2/2022.02.21_14.21.17_Ch/2022.02.21_14.21.17_Ch.tif]

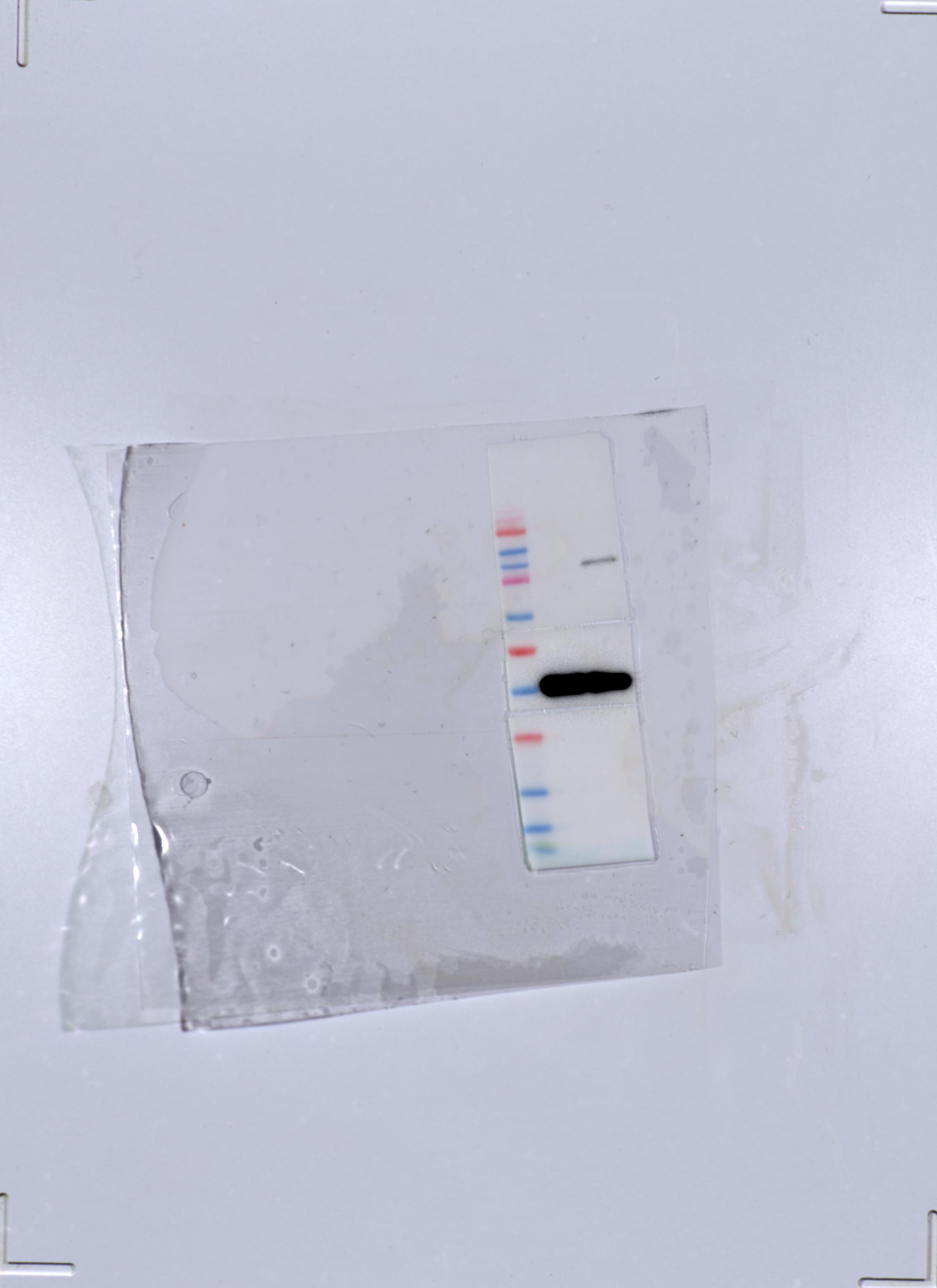

Supplement: Supplementary file 12 — EV Figure Source Data [file 44318_2025_453_MOESM12_ESM.zip › Source data EV-2/Figure EV1/EV1A/REP2/2022.02.21_14.21.17_Ch/2022.02.21_14.21.17_Ch+Marker.jpg]

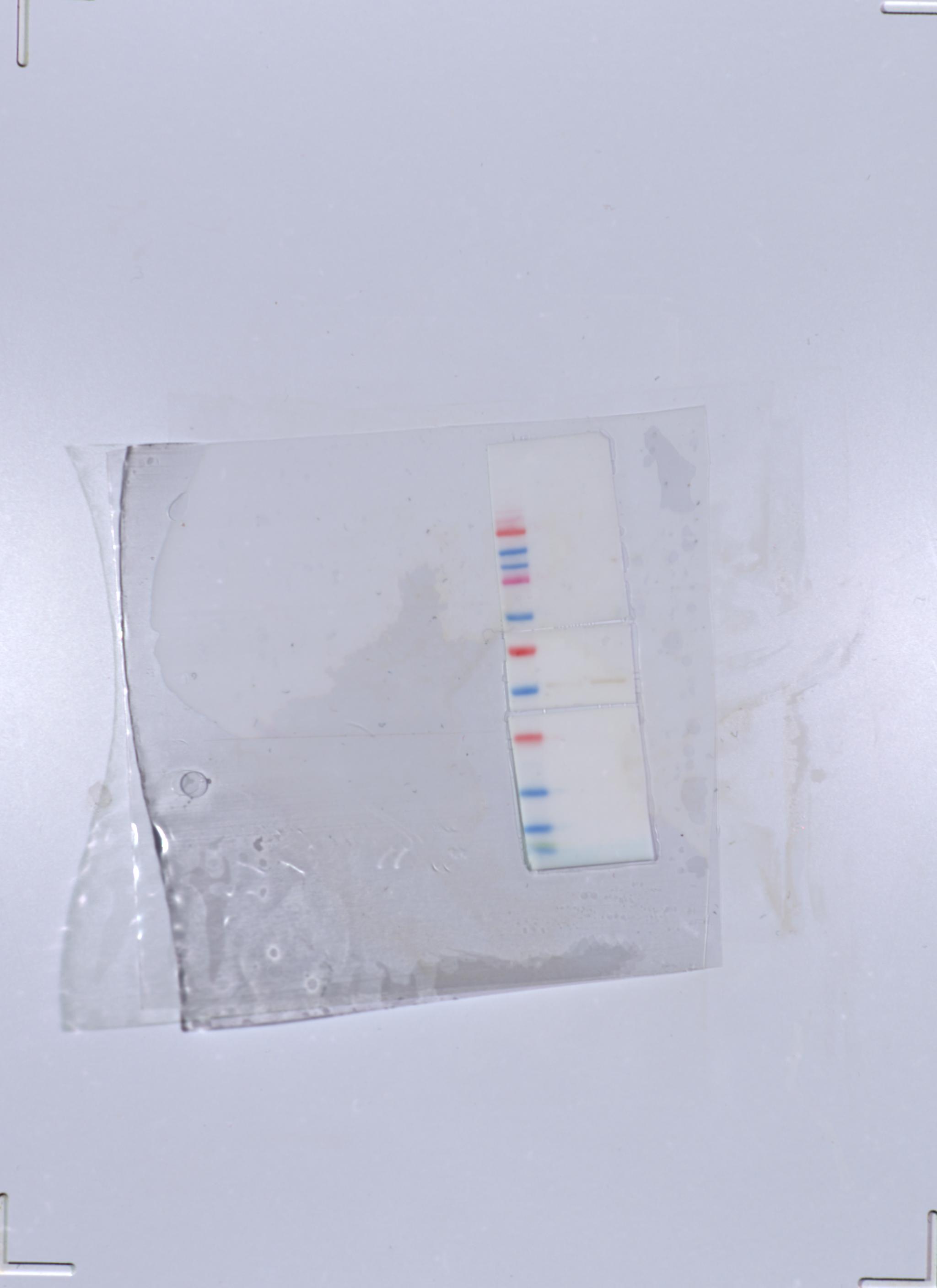

Supplement: Supplementary file 12 — EV Figure Source Data [file 44318_2025_453_MOESM12_ESM.zip › Source data EV-2/Figure EV1/EV1A/REP2/2022.02.21_14.21.17_Ch/2022.02.21_14.21.17_Ch-Marker.jpg]

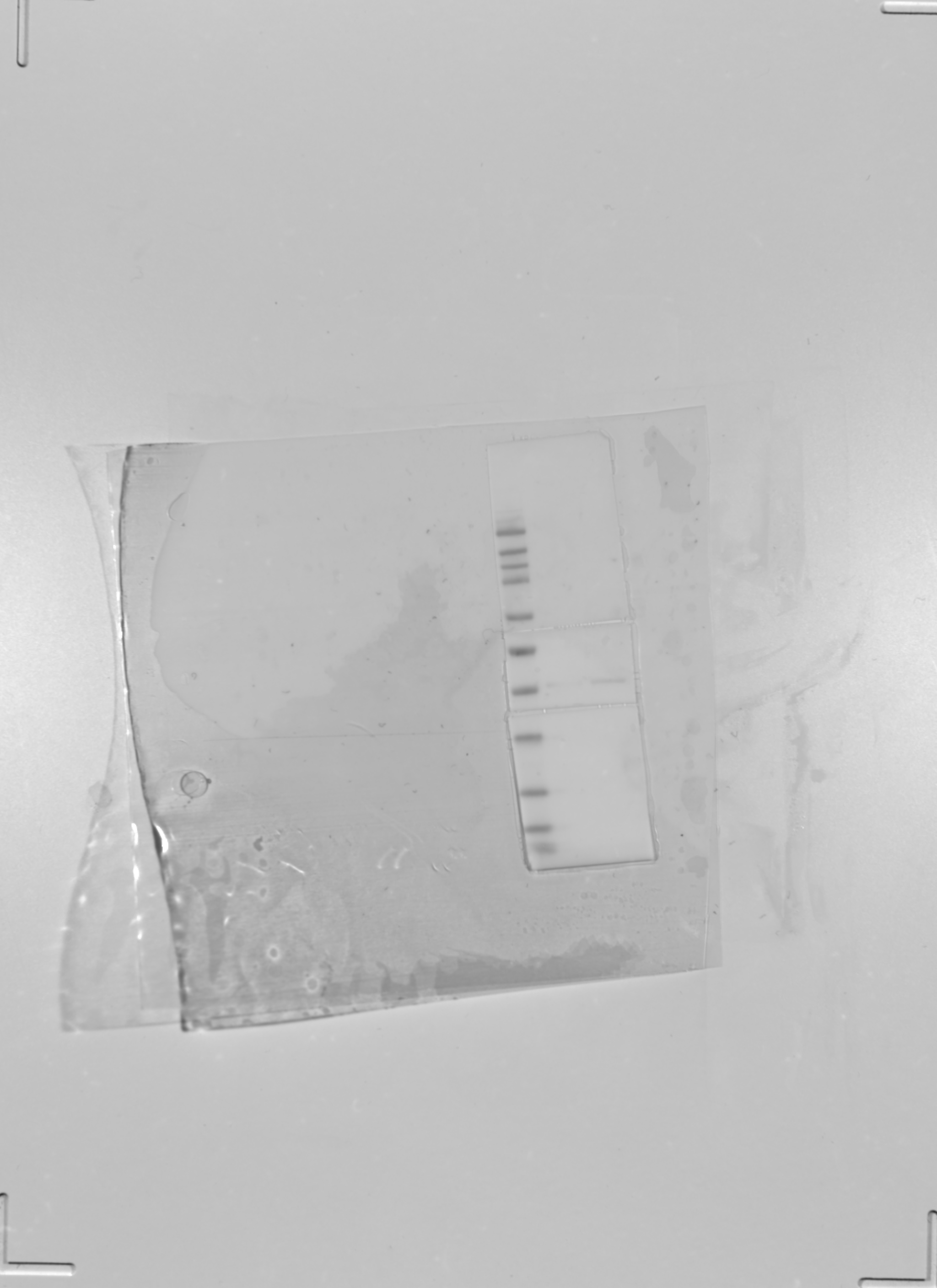

Supplement: Supplementary file 12 — EV Figure Source Data [file 44318_2025_453_MOESM12_ESM.zip › Source data EV-2/Figure EV1/EV1A/REP2/2022.02.21_14.21.17_Ch/2022.02.21_14.21.17_Ch-Marker.tif]

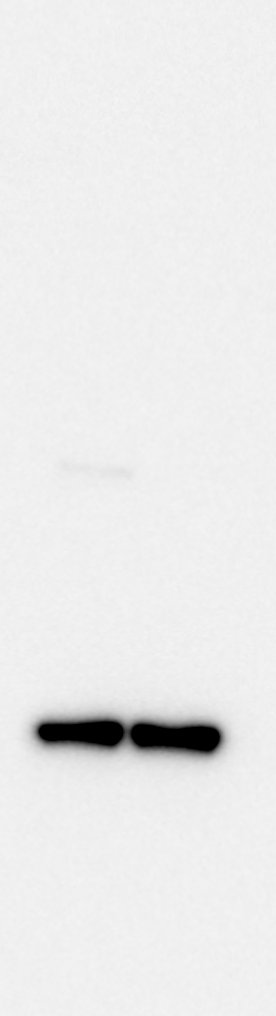

Supplement: Supplementary file 12 — EV Figure Source Data [file 44318_2025_453_MOESM12_ESM.zip › Source data EV-2/Figure EV1/EV1A/REP2/BLM tubuliln low expo 2022.02.21_14.17.12_Ch-1.jpg]

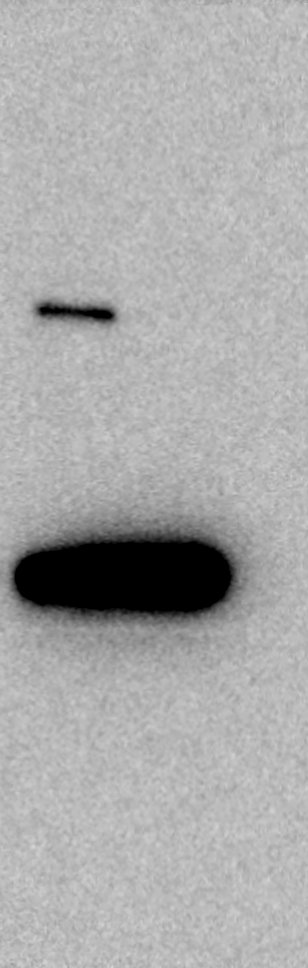

Supplement: Supplementary file 12 — EV Figure Source Data [file 44318_2025_453_MOESM12_ESM.zip › Source data EV-2/Figure EV1/EV1A/REP2/BLM tubulin 2022.02.21_14.21.17_Ch-1.jpg]

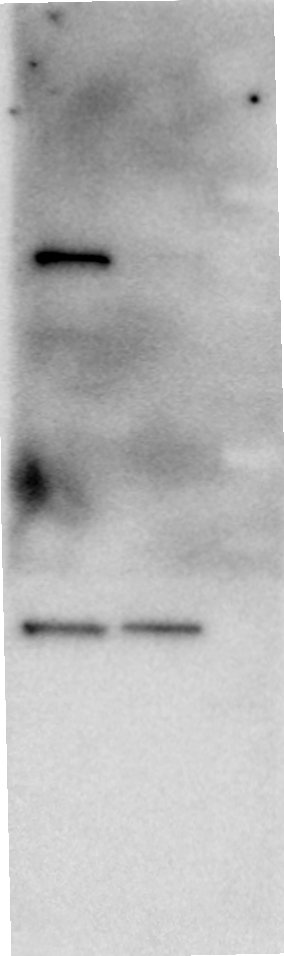

Supplement: Supplementary file 12 — EV Figure Source Data [file 44318_2025_453_MOESM12_ESM.zip › Source data EV-2/Figure EV1/EV1A/REP2/PICH and GAPDH 2022.02.20_19.59.18_Ch-1.jpg]

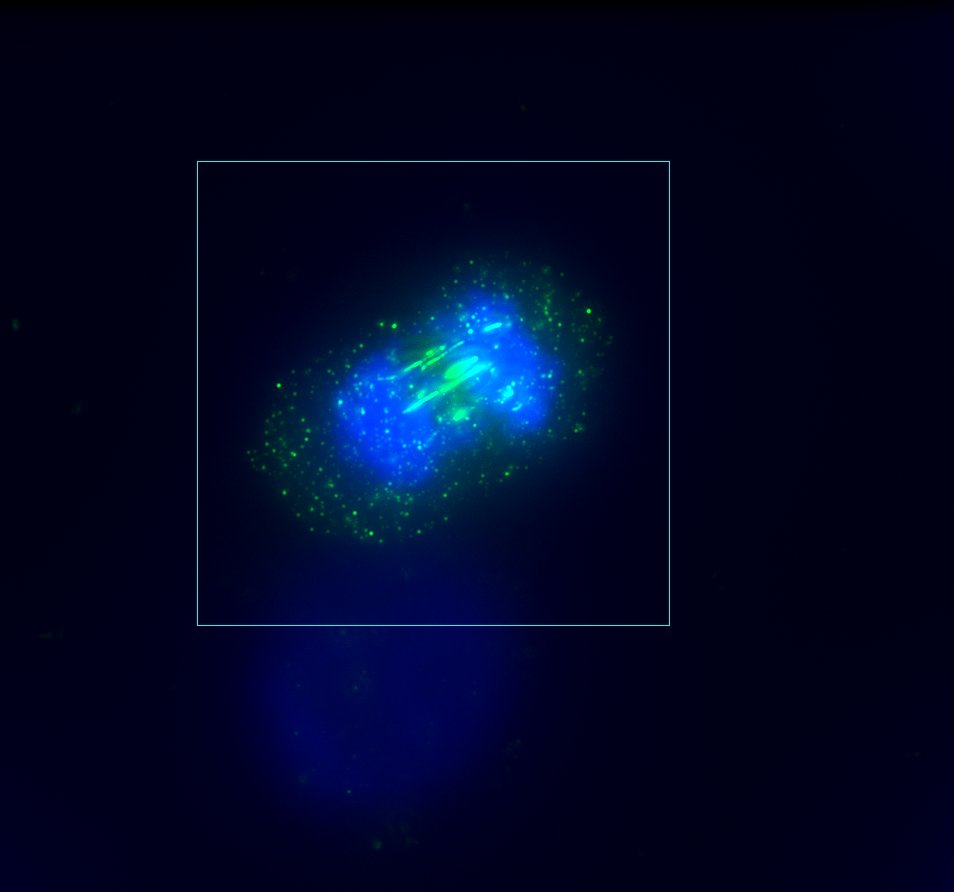

Supplement: Supplementary file 12 — EV Figure Source Data [file 44318_2025_453_MOESM12_ESM.zip › Source data EV-2/Figure EV1/EV1E/Raw siBLM +APH.jpg]

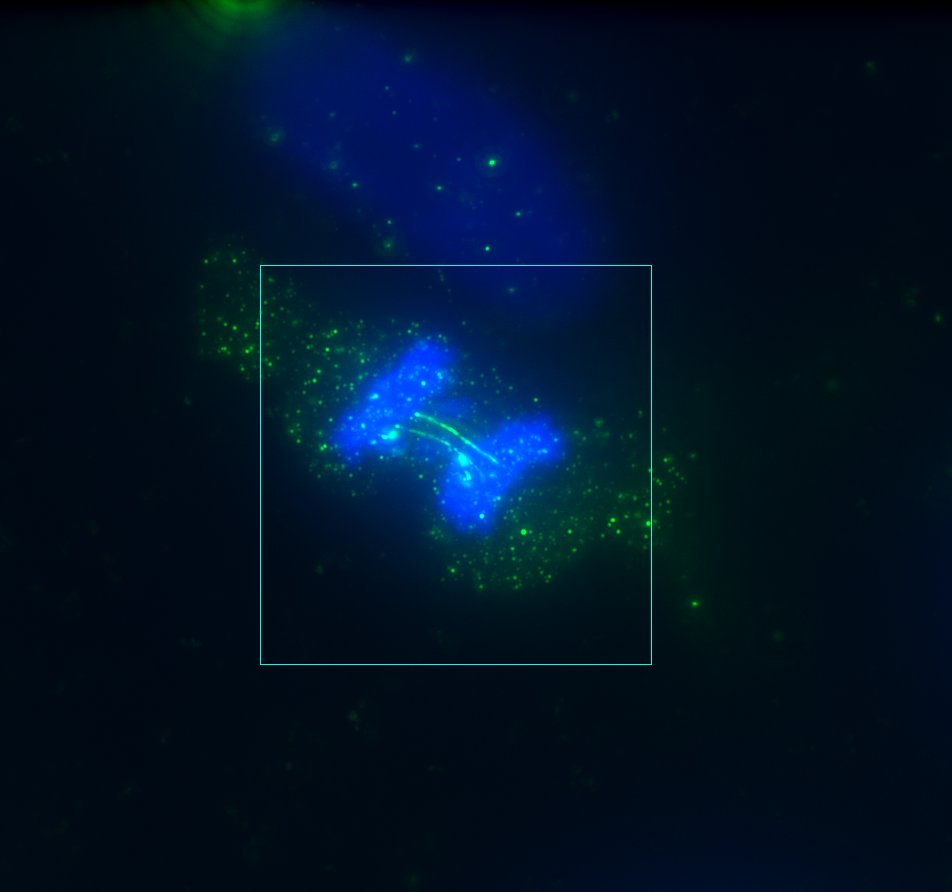

Supplement: Supplementary file 12 — EV Figure Source Data [file 44318_2025_453_MOESM12_ESM.zip › Source data EV-2/Figure EV1/EV1E/Raw siBLM -APH.jpg]

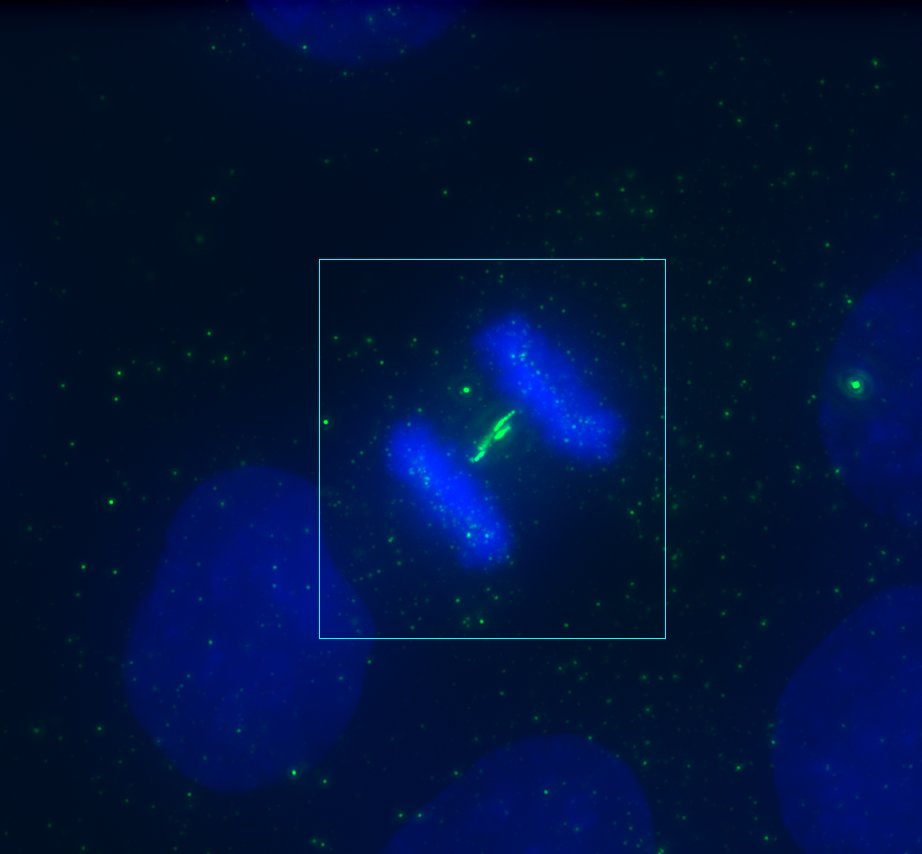

Supplement: Supplementary file 12 — EV Figure Source Data [file 44318_2025_453_MOESM12_ESM.zip › Source data EV-2/Figure EV1/EV1E/Raw siCon +APH.jpg]

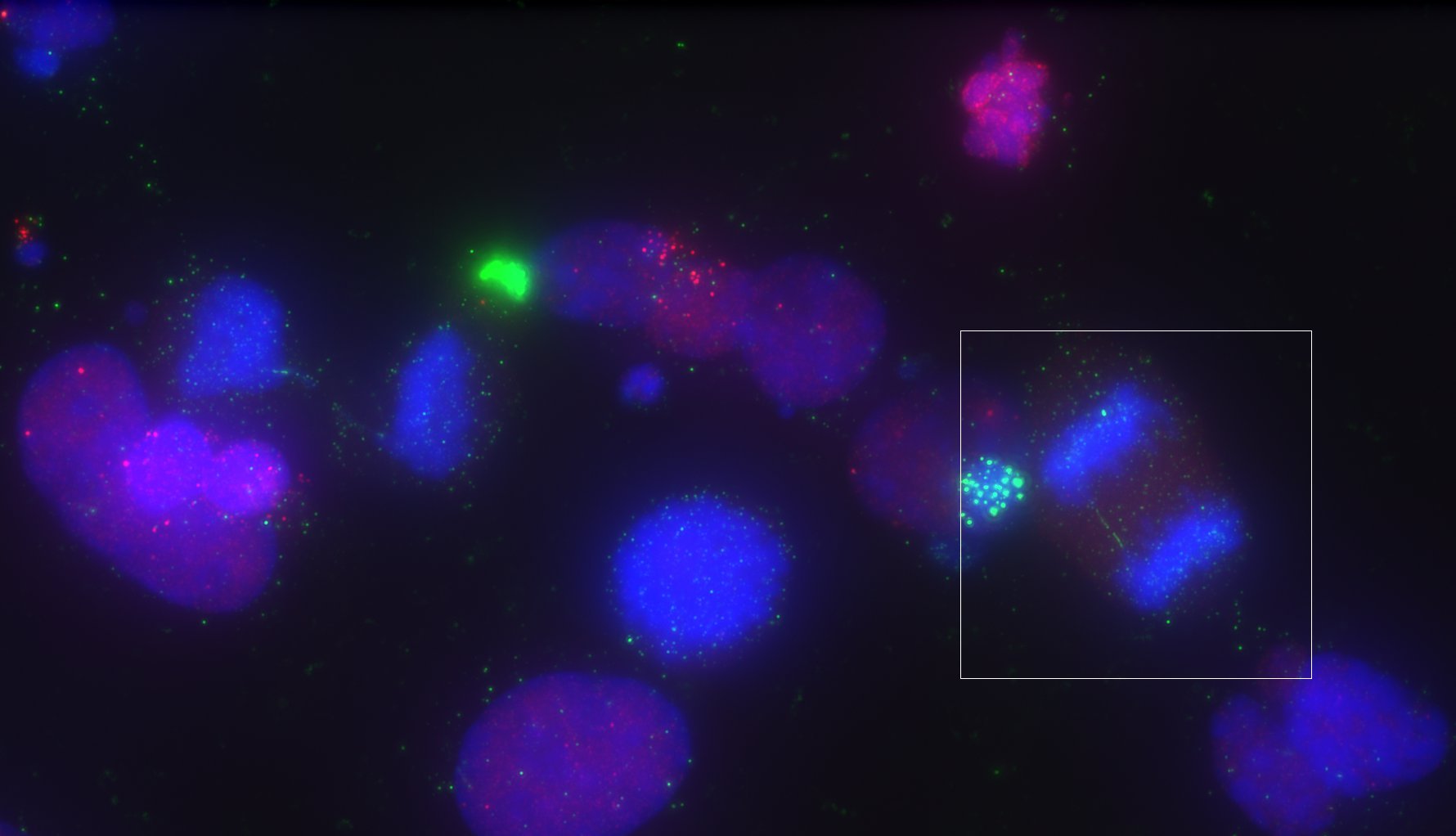

Supplement: Supplementary file 12 — EV Figure Source Data [file 44318_2025_453_MOESM12_ESM.zip › Source data EV-2/Figure EV1/EV1E/Raw siCon -APH.jpg]

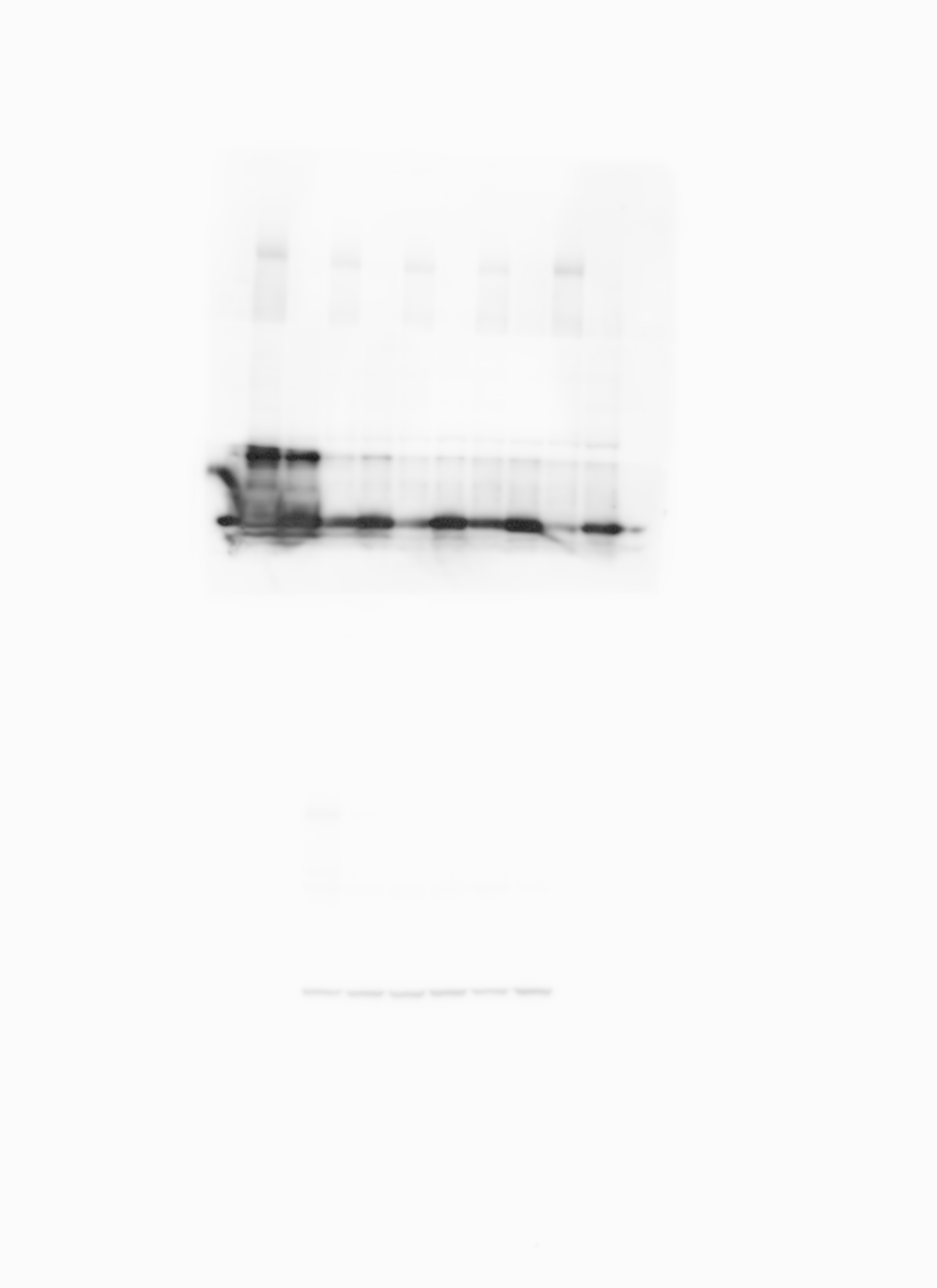

Supplement: Supplementary file 12 — EV Figure Source Data [file 44318_2025_453_MOESM12_ESM.zip › Source data EV-2/Figure EV2/EV2C/2021.01.22_19.27.51_Ch/2021.01.22_19.27.51_Ch.tif]

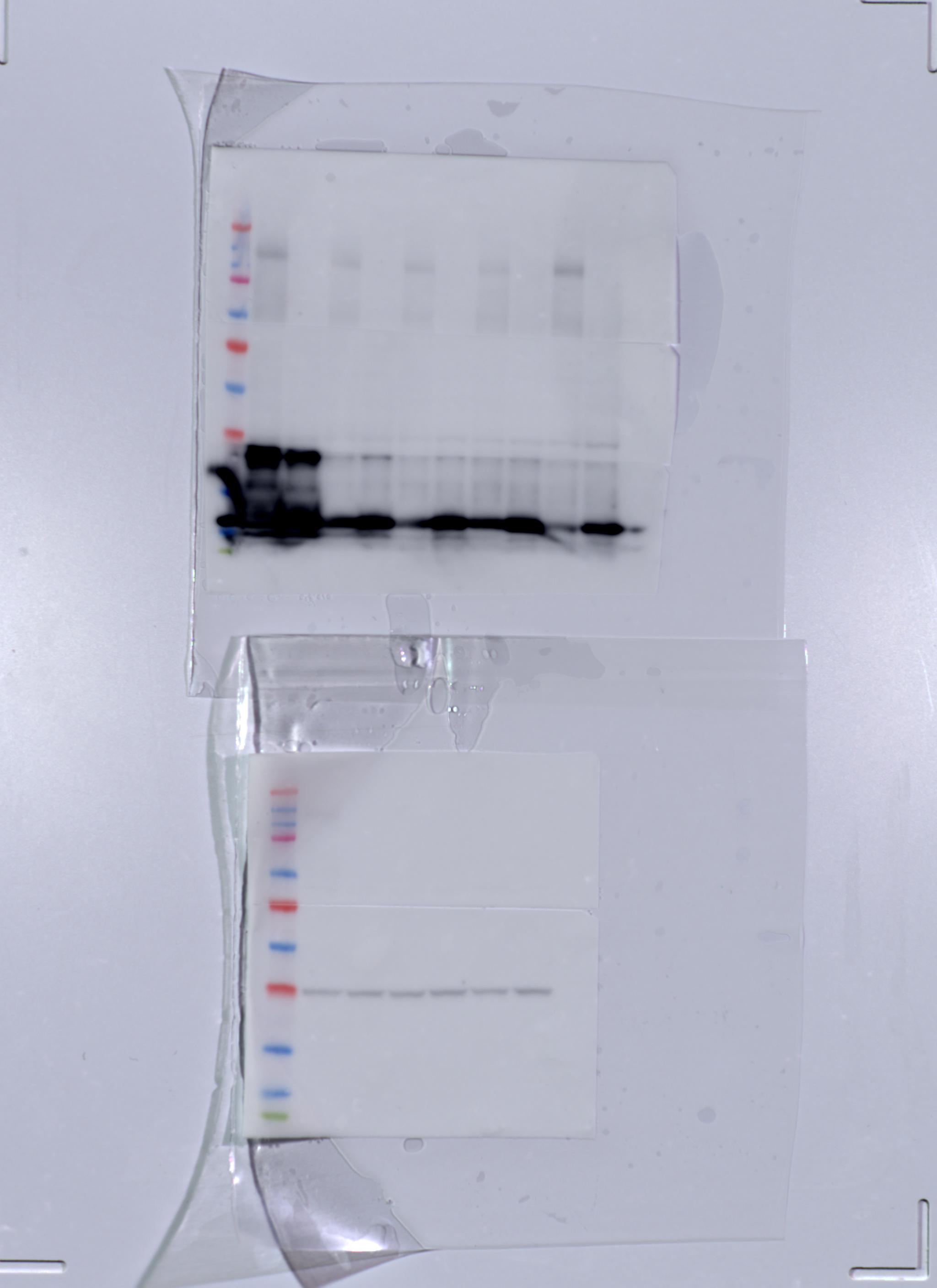

Supplement: Supplementary file 12 — EV Figure Source Data [file 44318_2025_453_MOESM12_ESM.zip › Source data EV-2/Figure EV2/EV2C/2021.01.22_19.27.51_Ch/2021.01.22_19.27.51_Ch+Marker.jpg]

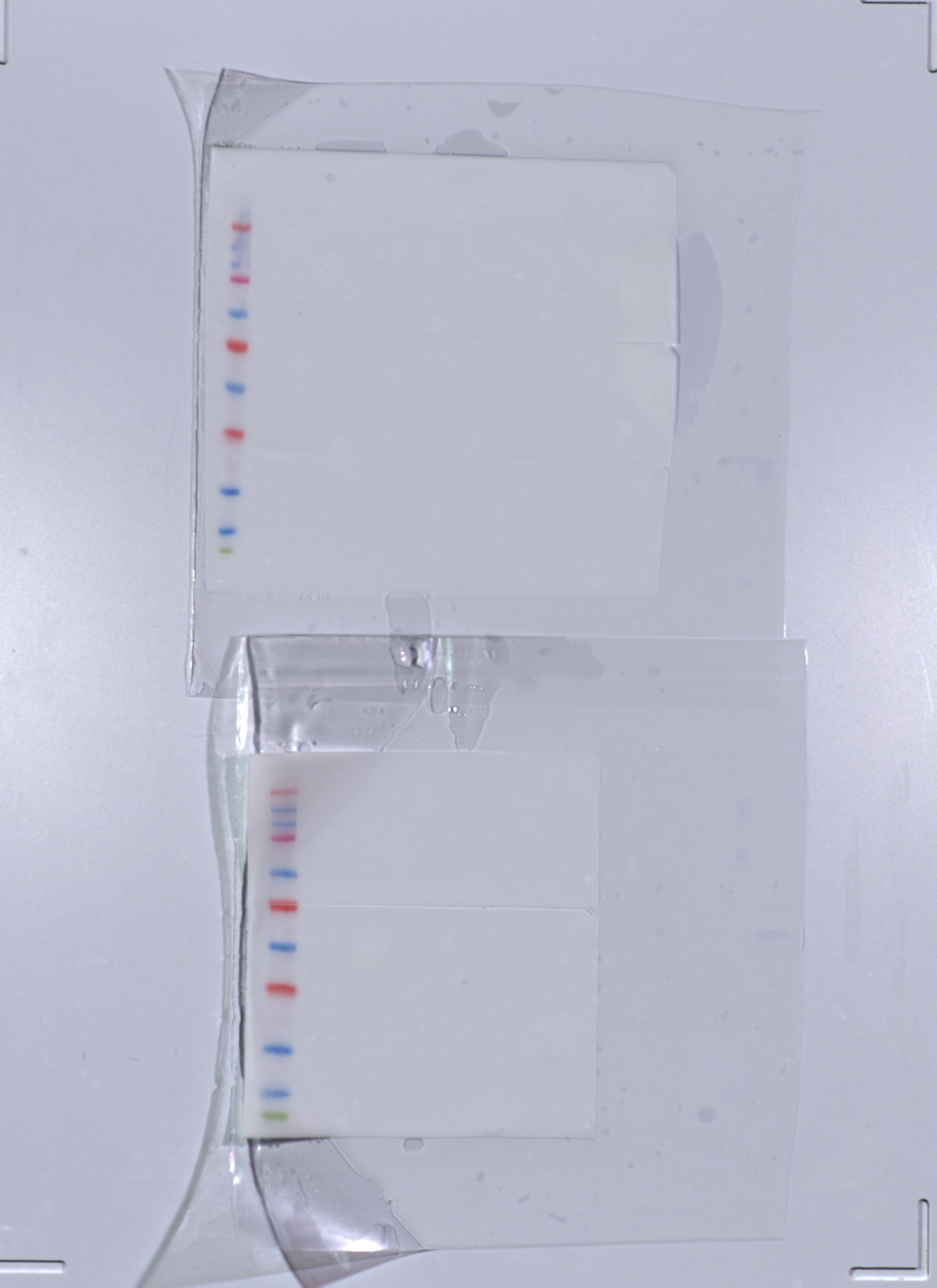

Supplement: Supplementary file 12 — EV Figure Source Data [file 44318_2025_453_MOESM12_ESM.zip › Source data EV-2/Figure EV2/EV2C/2021.01.22_19.27.51_Ch/2021.01.22_19.27.51_Ch-Marker.jpg]

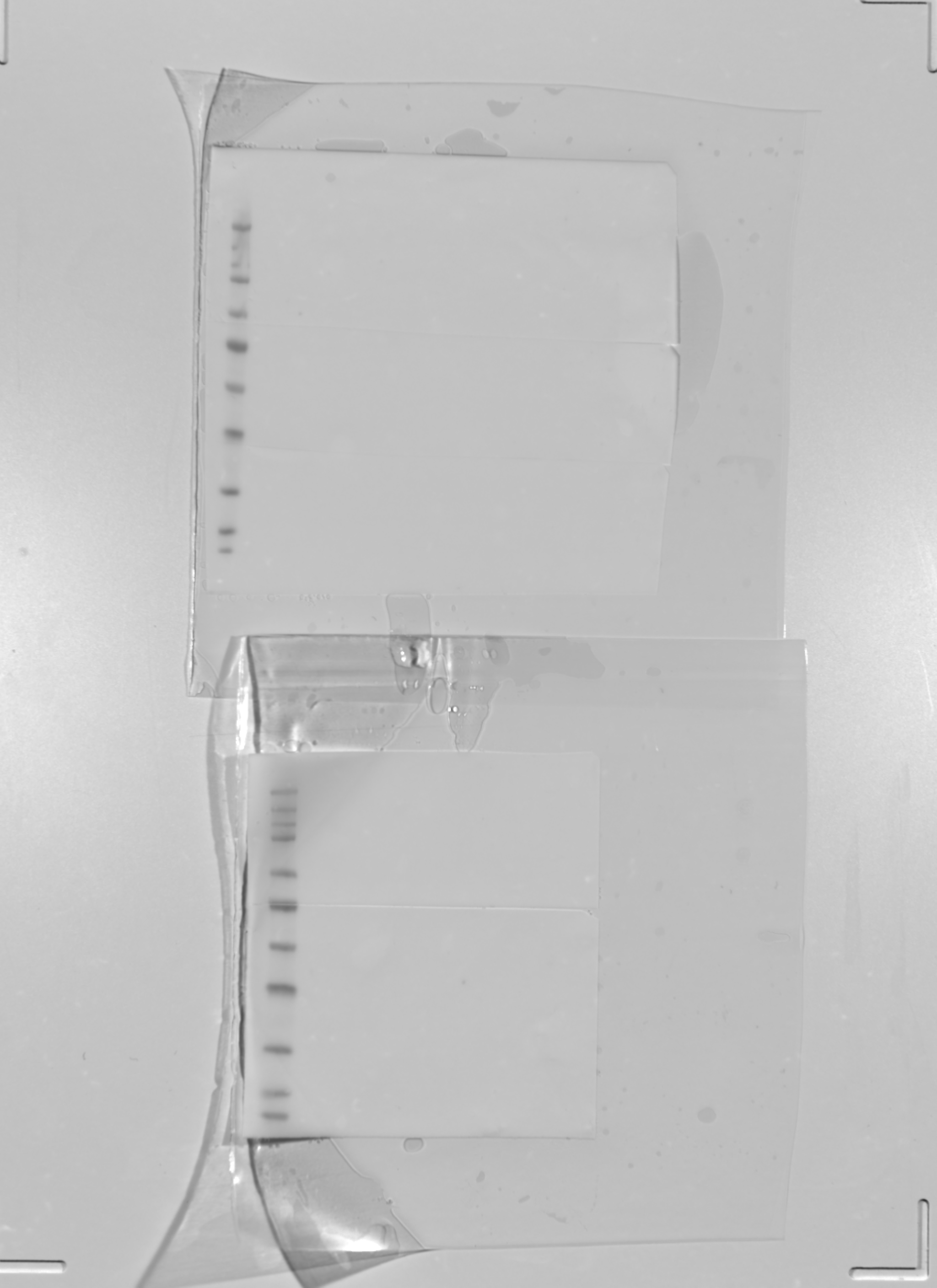

Supplement: Supplementary file 12 — EV Figure Source Data [file 44318_2025_453_MOESM12_ESM.zip › Source data EV-2/Figure EV2/EV2C/2021.01.22_19.27.51_Ch/2021.01.22_19.27.51_Ch-Marker.tif]

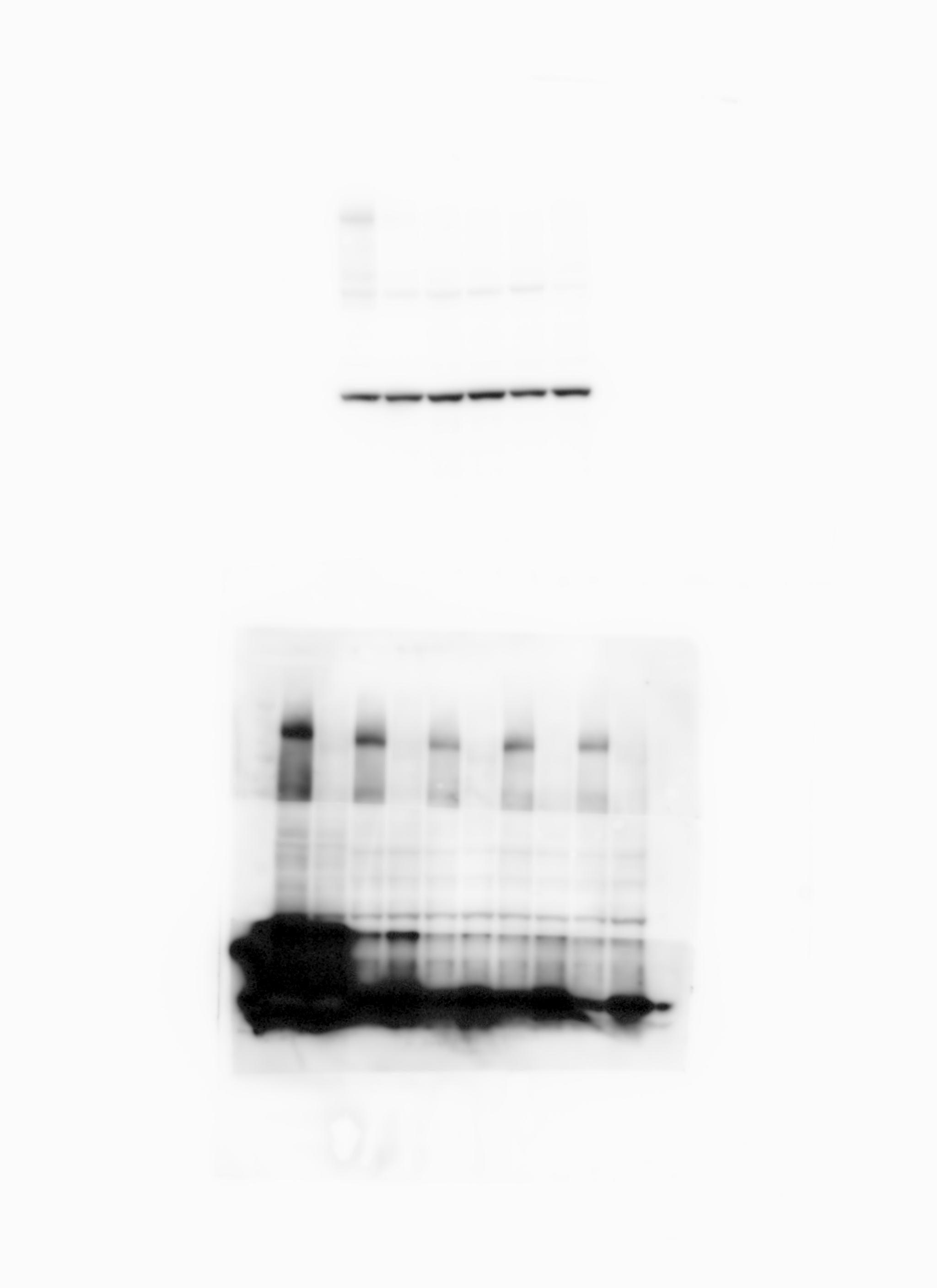

Supplement: Supplementary file 12 — EV Figure Source Data [file 44318_2025_453_MOESM12_ESM.zip › Source data EV-2/Figure EV2/EV2C/2021.01.22_19.51.19_Ch/2021.01.22_19.51.19_Ch.tif]

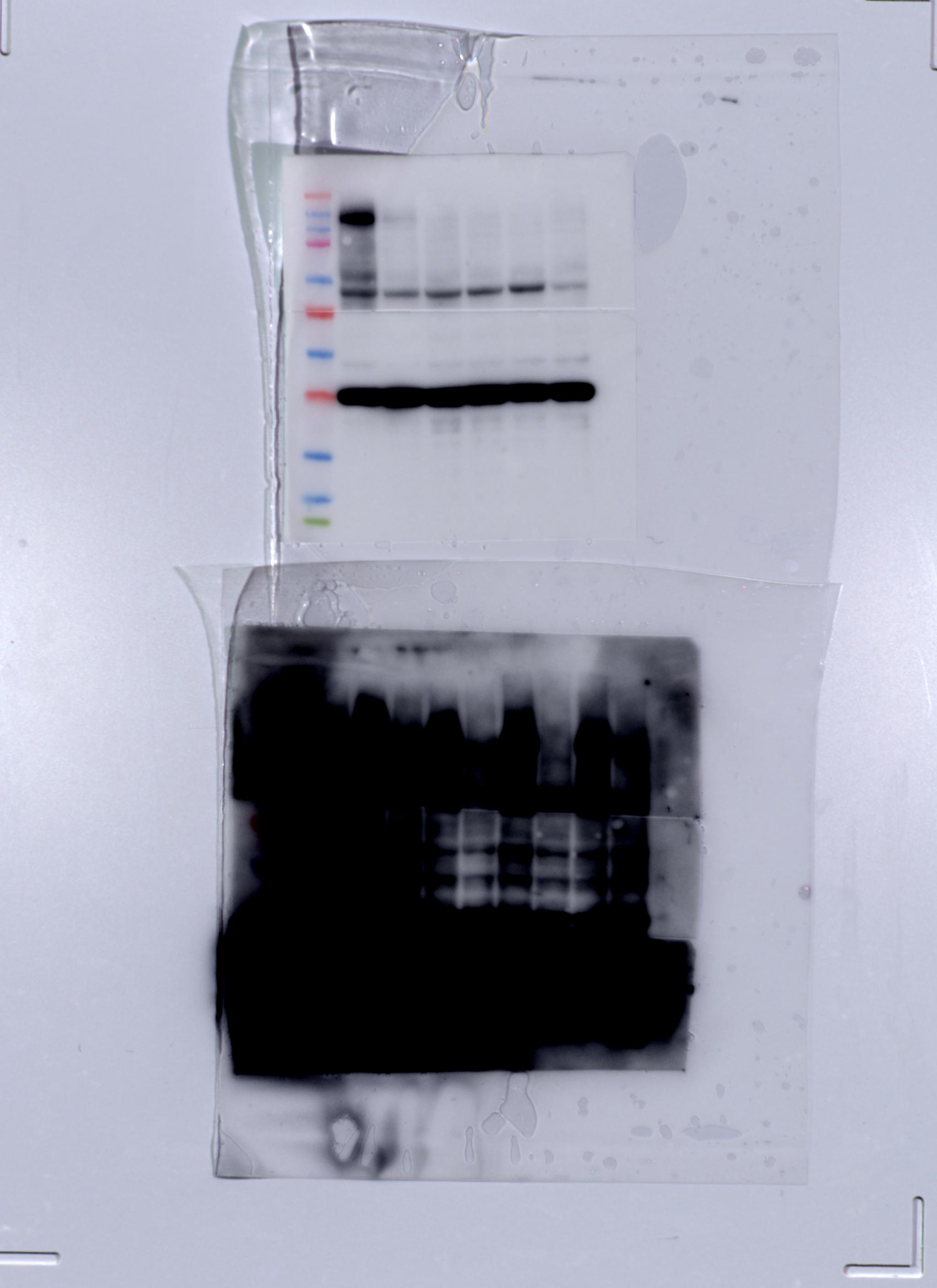

Supplement: Supplementary file 12 — EV Figure Source Data [file 44318_2025_453_MOESM12_ESM.zip › Source data EV-2/Figure EV2/EV2C/2021.01.22_19.51.19_Ch/2021.01.22_19.51.19_Ch+Marker.jpg]

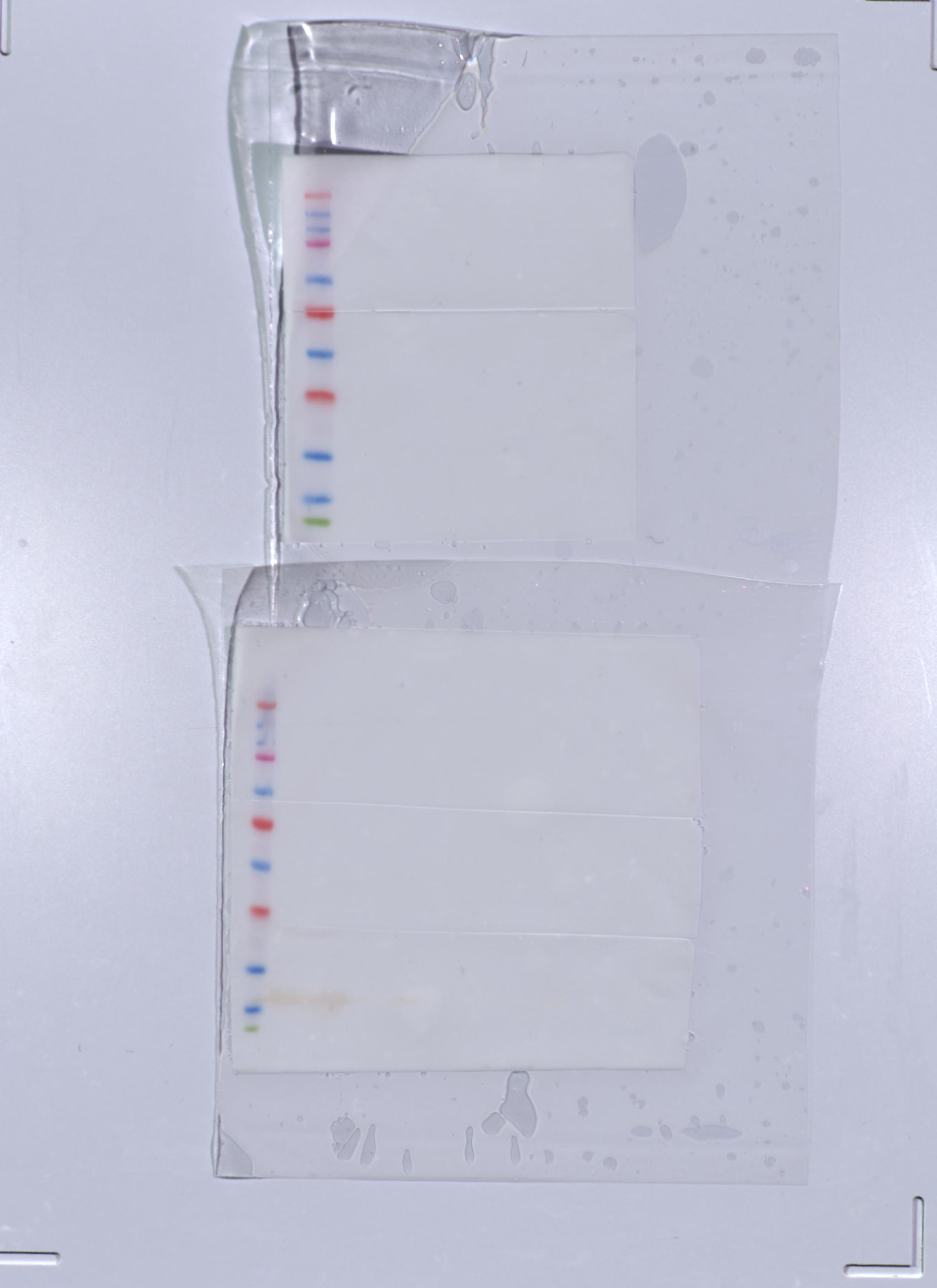

Supplement: Supplementary file 12 — EV Figure Source Data [file 44318_2025_453_MOESM12_ESM.zip › Source data EV-2/Figure EV2/EV2C/2021.01.22_19.51.19_Ch/2021.01.22_19.51.19_Ch-Marker.jpg]

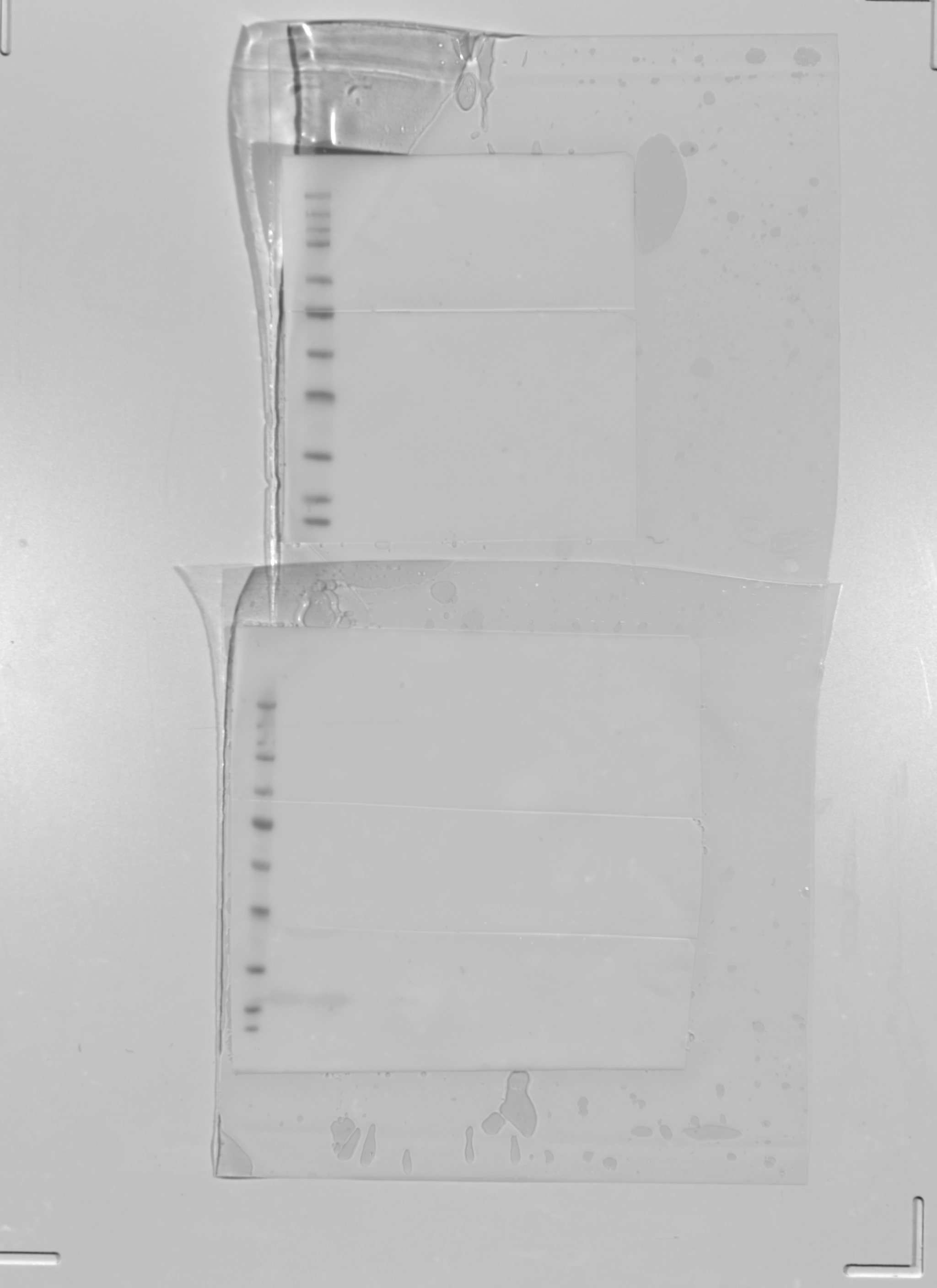

Supplement: Supplementary file 12 — EV Figure Source Data [file 44318_2025_453_MOESM12_ESM.zip › Source data EV-2/Figure EV2/EV2C/2021.01.22_19.51.19_Ch/2021.01.22_19.51.19_Ch-Marker.tif]

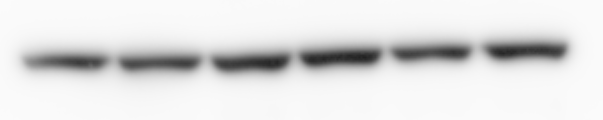

Supplement: Supplementary file 12 — EV Figure Source Data [file 44318_2025_453_MOESM12_ESM.zip › Source data EV-2/Figure EV2/EV2C/blm degradation degron 22012021 gapdh.tif]

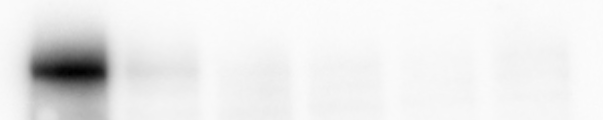

Supplement: Supplementary file 12 — EV Figure Source Data [file 44318_2025_453_MOESM12_ESM.zip › Source data EV-2/Figure EV2/EV2C/blm degradation degron 22012021.tif]

Low exposure

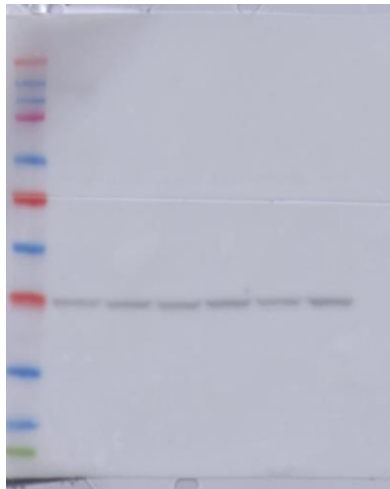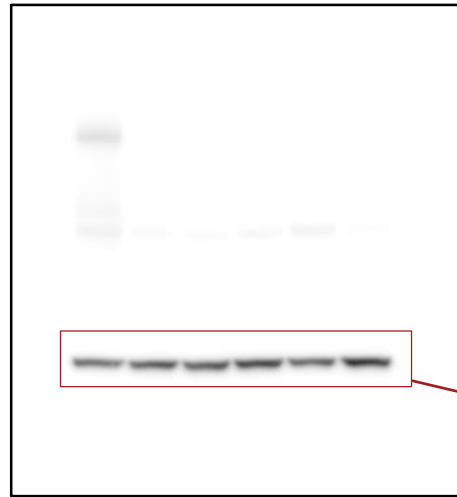

High exposure

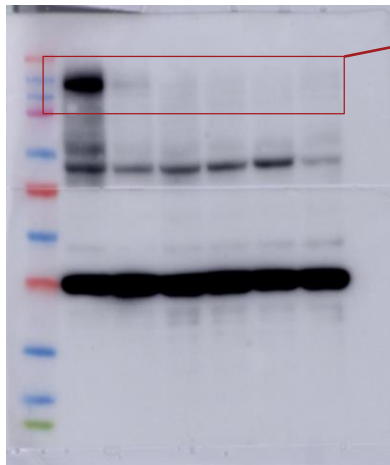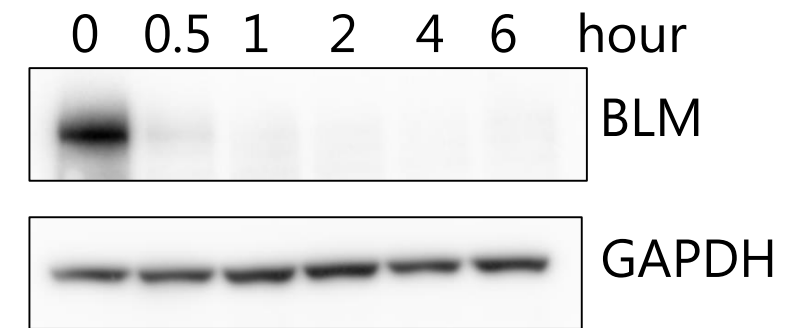

Supplement: Supplementary file 12 — EV Figure Source Data [file 44318_2025_453_MOESM12_ESM.zip › Source data EV-2/Figure EV2/EV2C/Readme figures EV2C.pdf]

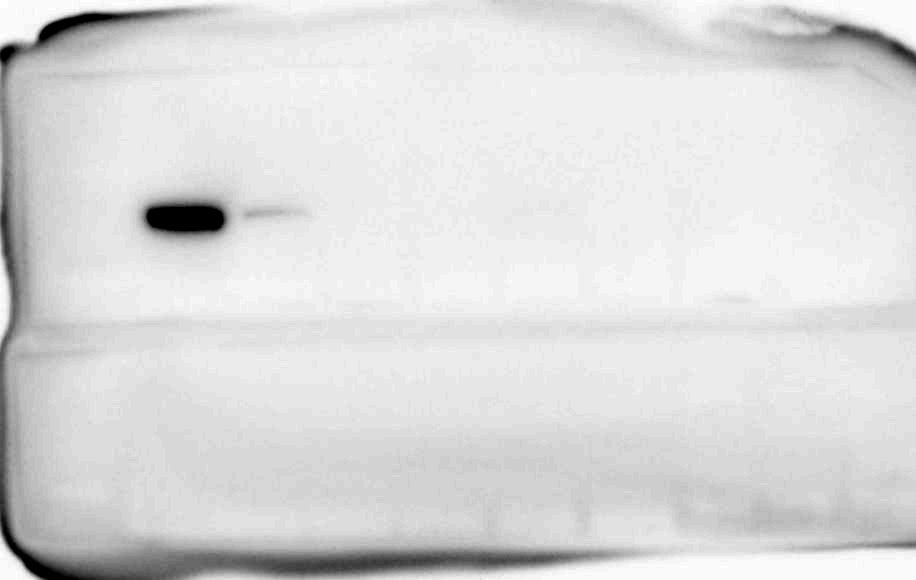

Supplement: Supplementary file 12 — EV Figure Source Data [file 44318_2025_453_MOESM12_ESM.zip › Source data EV-2/Figure EV2/EV2D/blm depletion.tif]

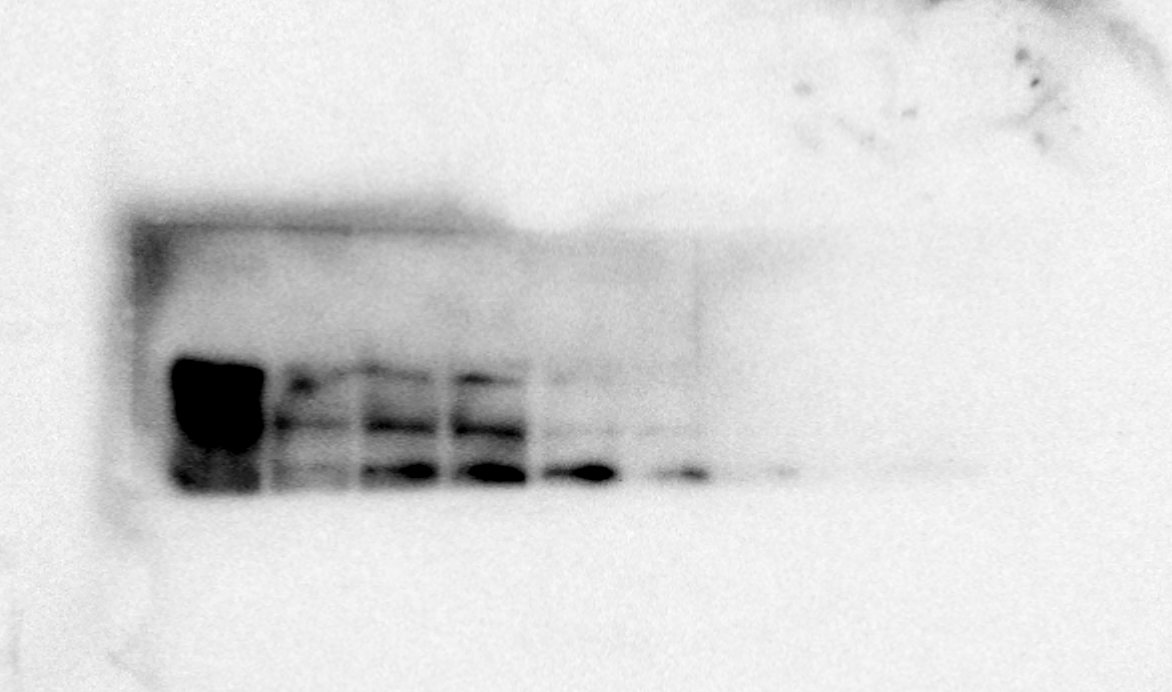

Supplement: Supplementary file 12 — EV Figure Source Data [file 44318_2025_453_MOESM12_ESM.zip › Source data EV-2/Figure EV2/EV2D/blm recovery.jpg]

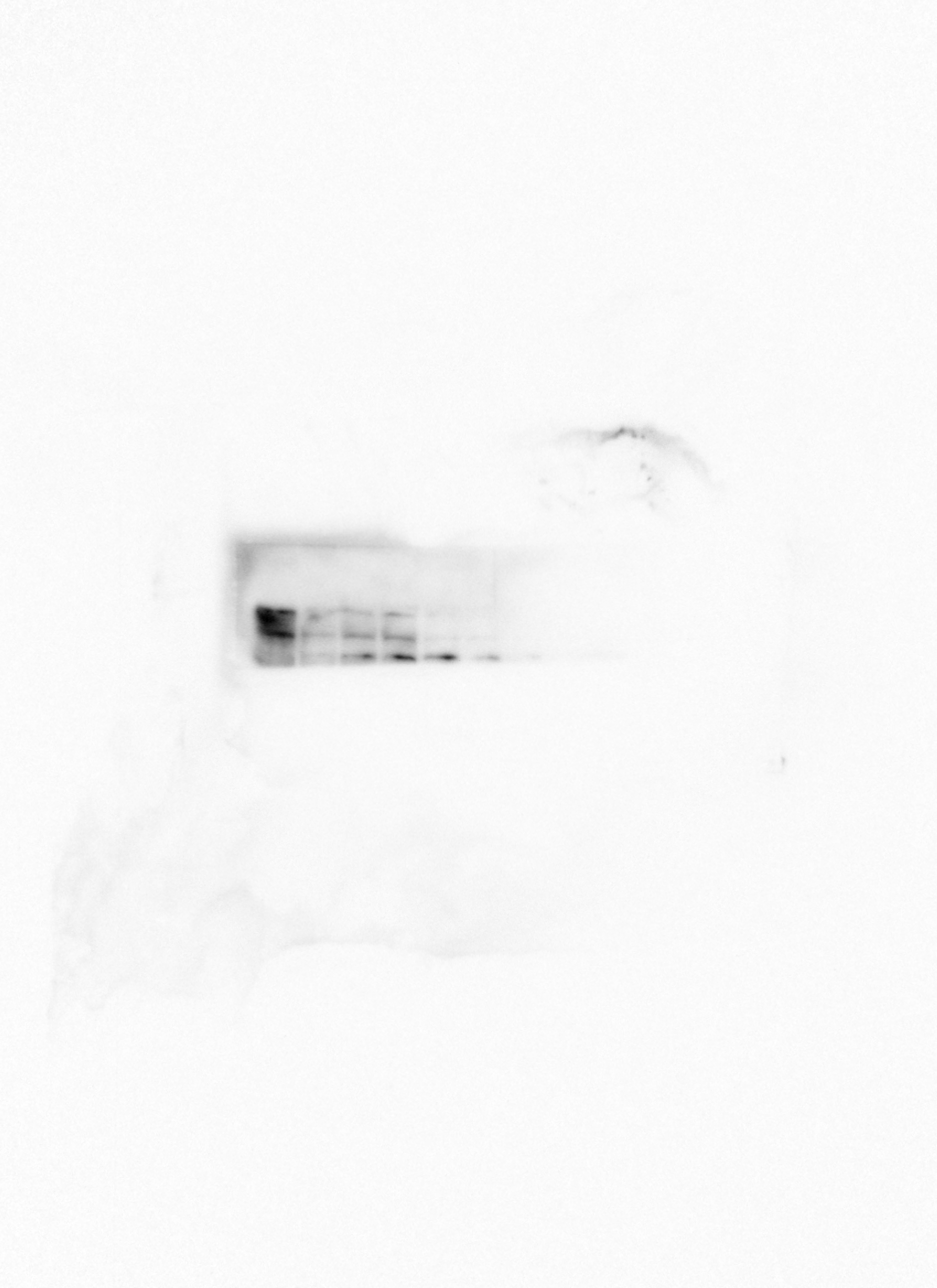

Supplement: Supplementary file 12 — EV Figure Source Data [file 44318_2025_453_MOESM12_ESM.zip › Source data EV-2/Figure EV2/EV2D/BLM_recovery/misBLM6 2024.11.14_15.45.42_Ch.tif]

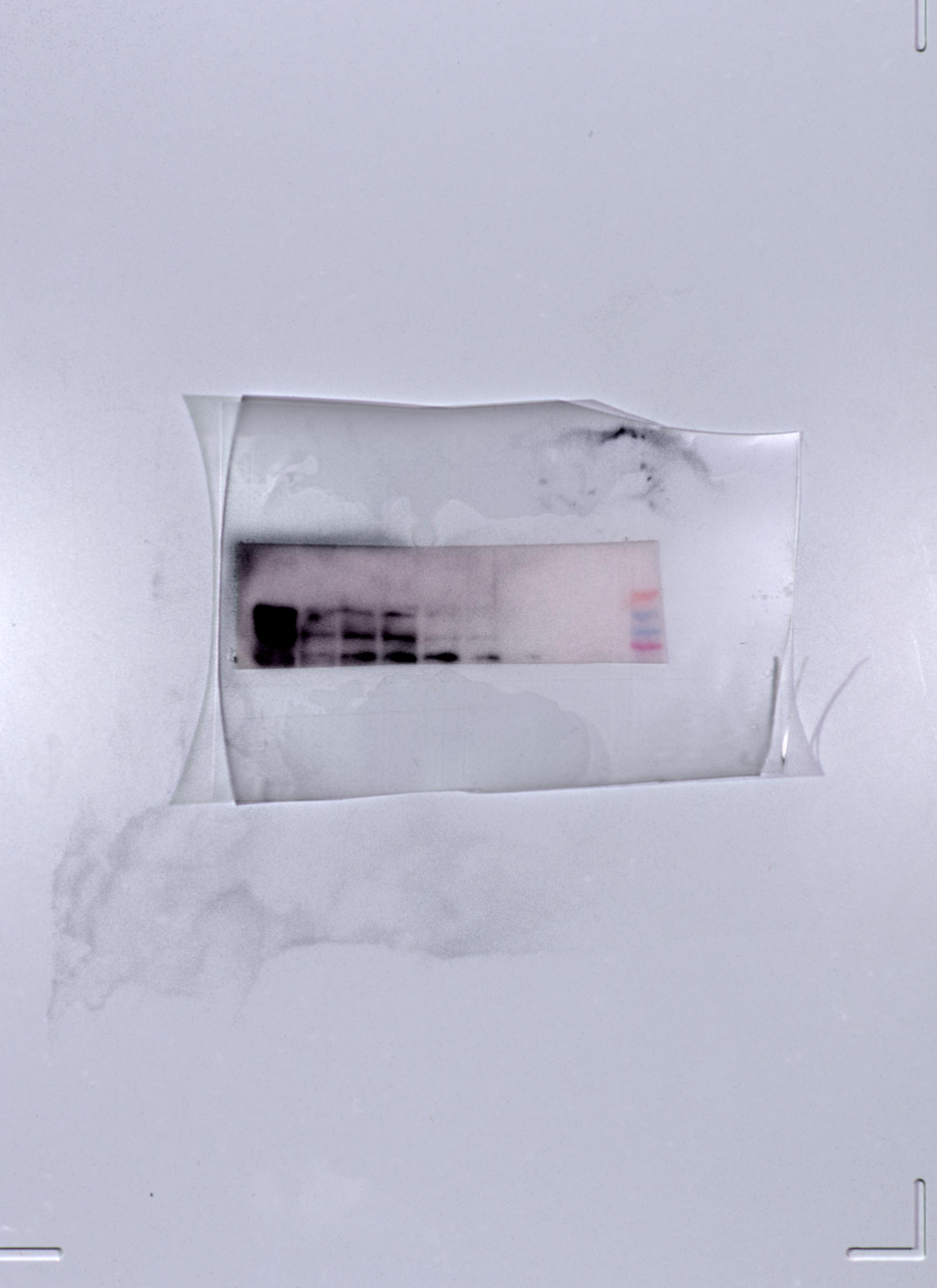

Supplement: Supplementary file 12 — EV Figure Source Data [file 44318_2025_453_MOESM12_ESM.zip › Source data EV-2/Figure EV2/EV2D/BLM_recovery/misBLM6 2024.11.14_15.45.42_Ch+Marker.jpg]

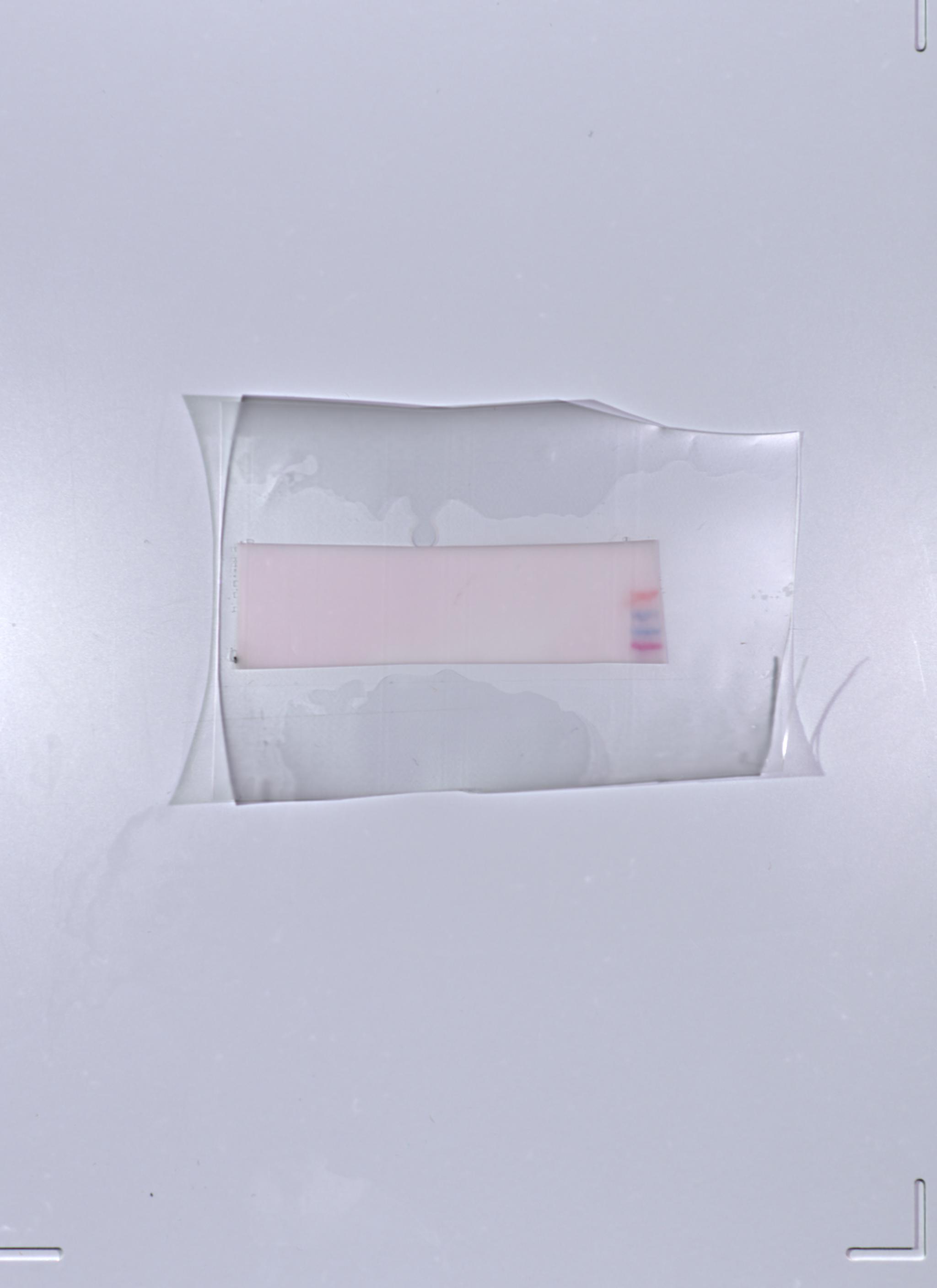

Supplement: Supplementary file 12 — EV Figure Source Data [file 44318_2025_453_MOESM12_ESM.zip › Source data EV-2/Figure EV2/EV2D/BLM_recovery/misBLM6 2024.11.14_15.45.42_Ch-Marker.jpg]

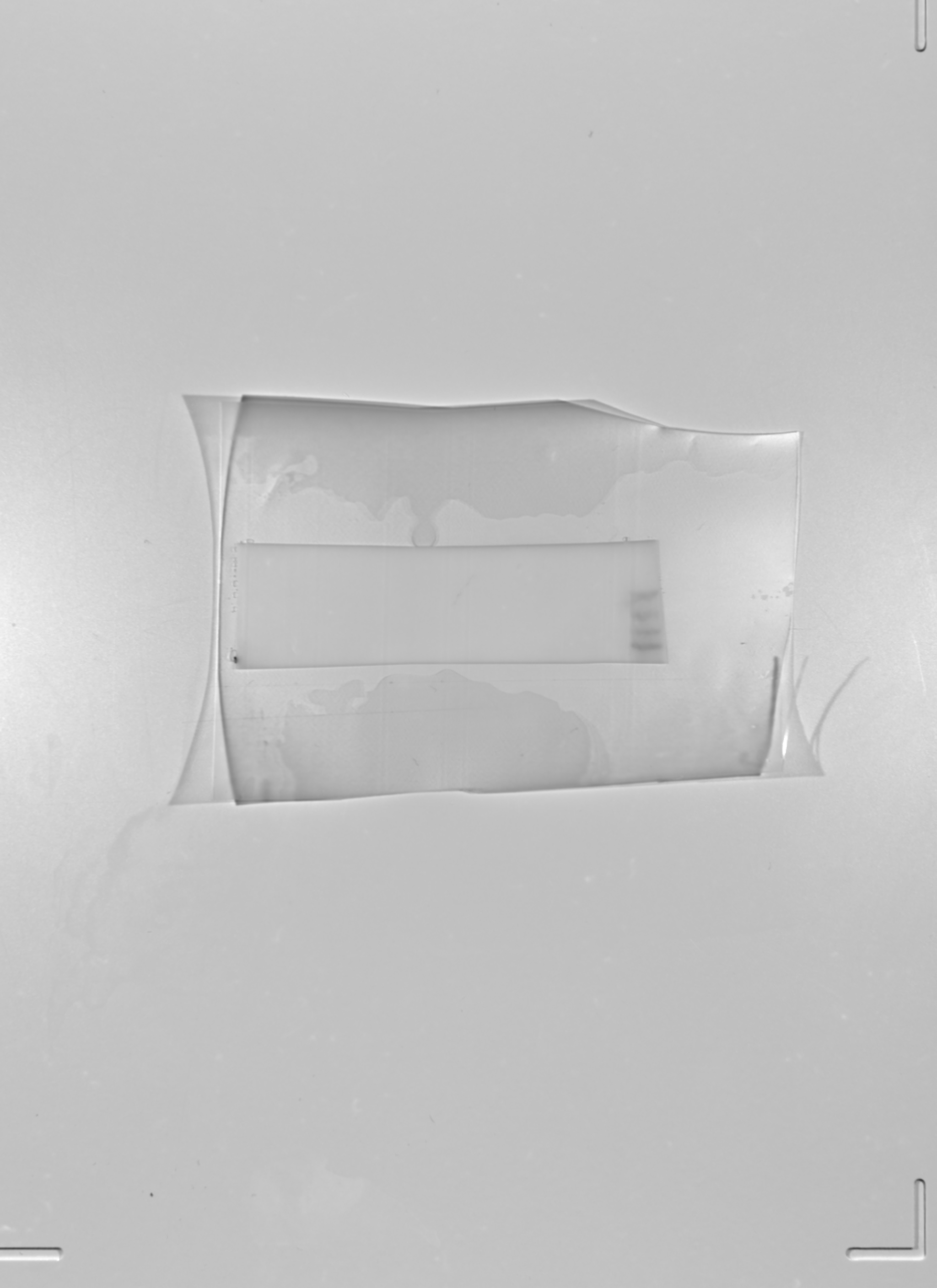

Supplement: Supplementary file 12 — EV Figure Source Data [file 44318_2025_453_MOESM12_ESM.zip › Source data EV-2/Figure EV2/EV2D/BLM_recovery/misBLM6 2024.11.14_15.45.42_Ch-Marker.tif]
