# Supplementary material for: Mechanistic insight into anaphase bridge signaling to the abscission checkpoint
Source: EMBO J. 2025 May 12;44(13):3824–52. doi: 10.1038/s44318-025-00453-w (PMC12217976; doi:10.1038/s44318-025-00453-w)
Supplement: Supplementary file 12 — EV Figure Source Data [file 44318_2025_453_MOESM12_ESM.zip › Source data EV-2/Figure EV4/EV4A/Readme.docx]

**Read Me:**

Dapi: Blue channel

PICH: Green channel

RPA: Red channel

Bright field to depict Midbody stage cells
