# Supplementary figures and images for: Mechanistic insight into anaphase bridge signaling to the abscission checkpoint (part 2 of 2)
Source: EMBO J. 2025 May 12;44(13):3824–52. doi: 10.1038/s44318-025-00453-w (PMC12217976; doi:10.1038/s44318-025-00453-w)

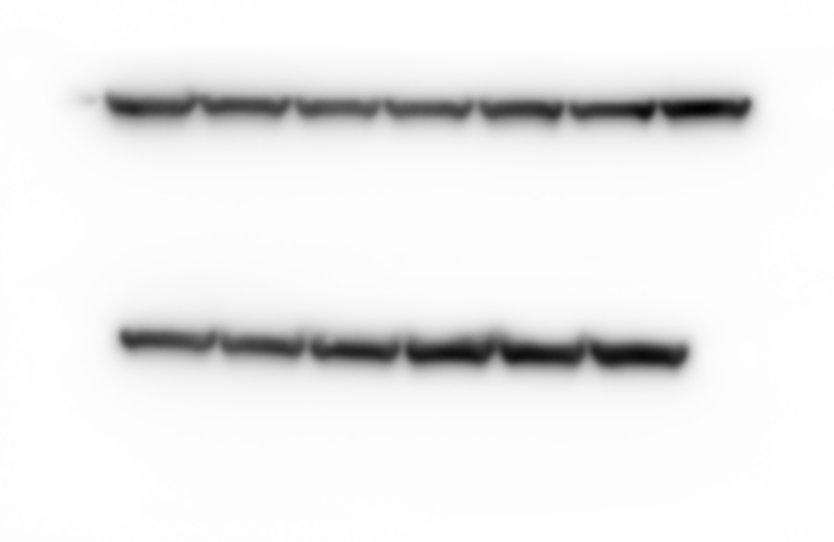

Supplement: Supplementary file 12 — EV Figure Source Data [file 44318_2025_453_MOESM12_ESM.zip › Source data EV-2/Figure EV2/EV2D/gapdh depletion.jpg]

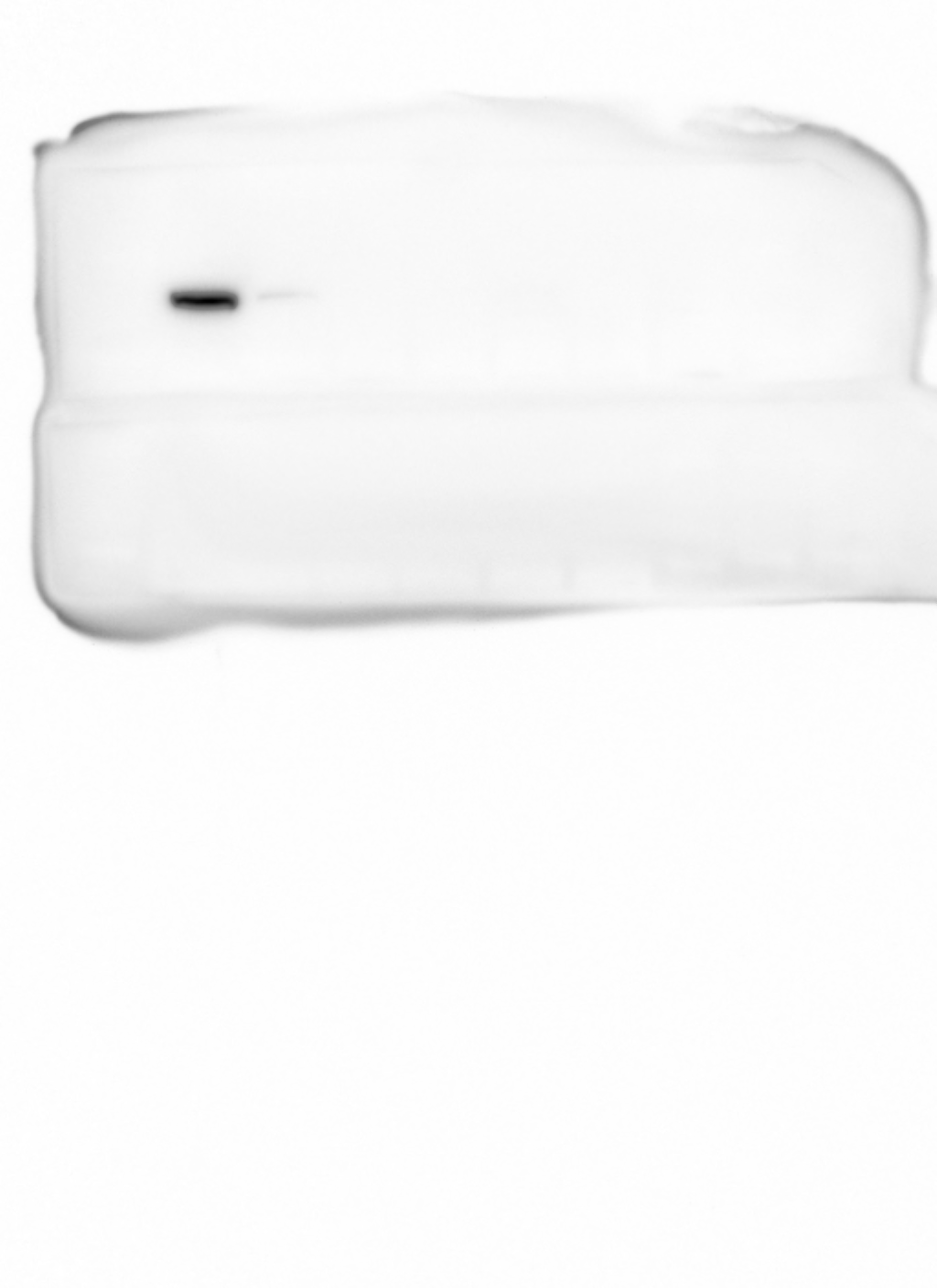

Supplement: Supplementary file 12 — EV Figure Source Data [file 44318_2025_453_MOESM12_ESM.zip › Source data EV-2/Figure EV2/EV2D/GapDH depletion/blm5min 2024.09.02_16.55.43_Ch.tif]

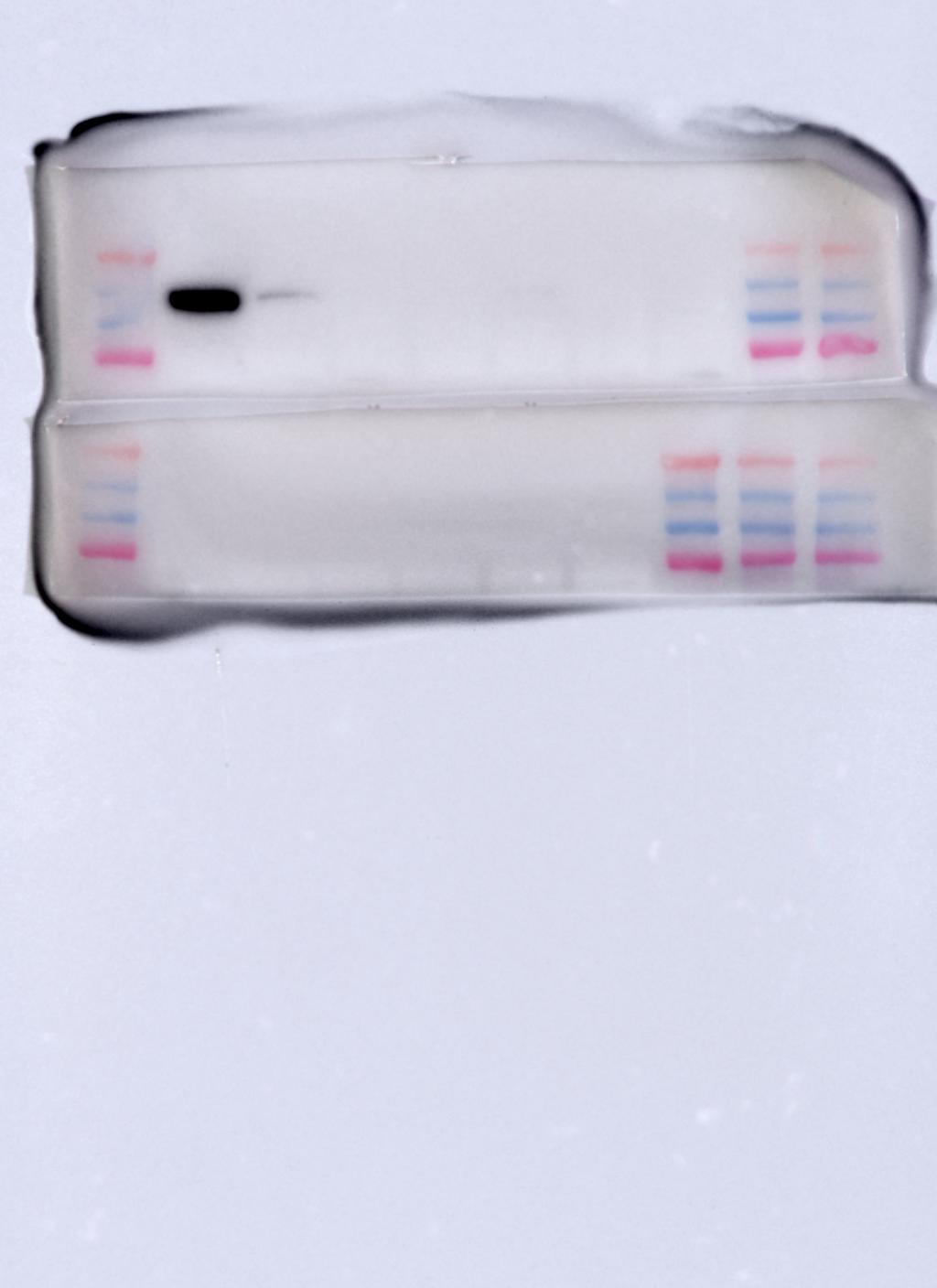

Supplement: Supplementary file 12 — EV Figure Source Data [file 44318_2025_453_MOESM12_ESM.zip › Source data EV-2/Figure EV2/EV2D/GapDH depletion/blm5min 2024.09.02_16.55.43_Ch+Marker.jpg]

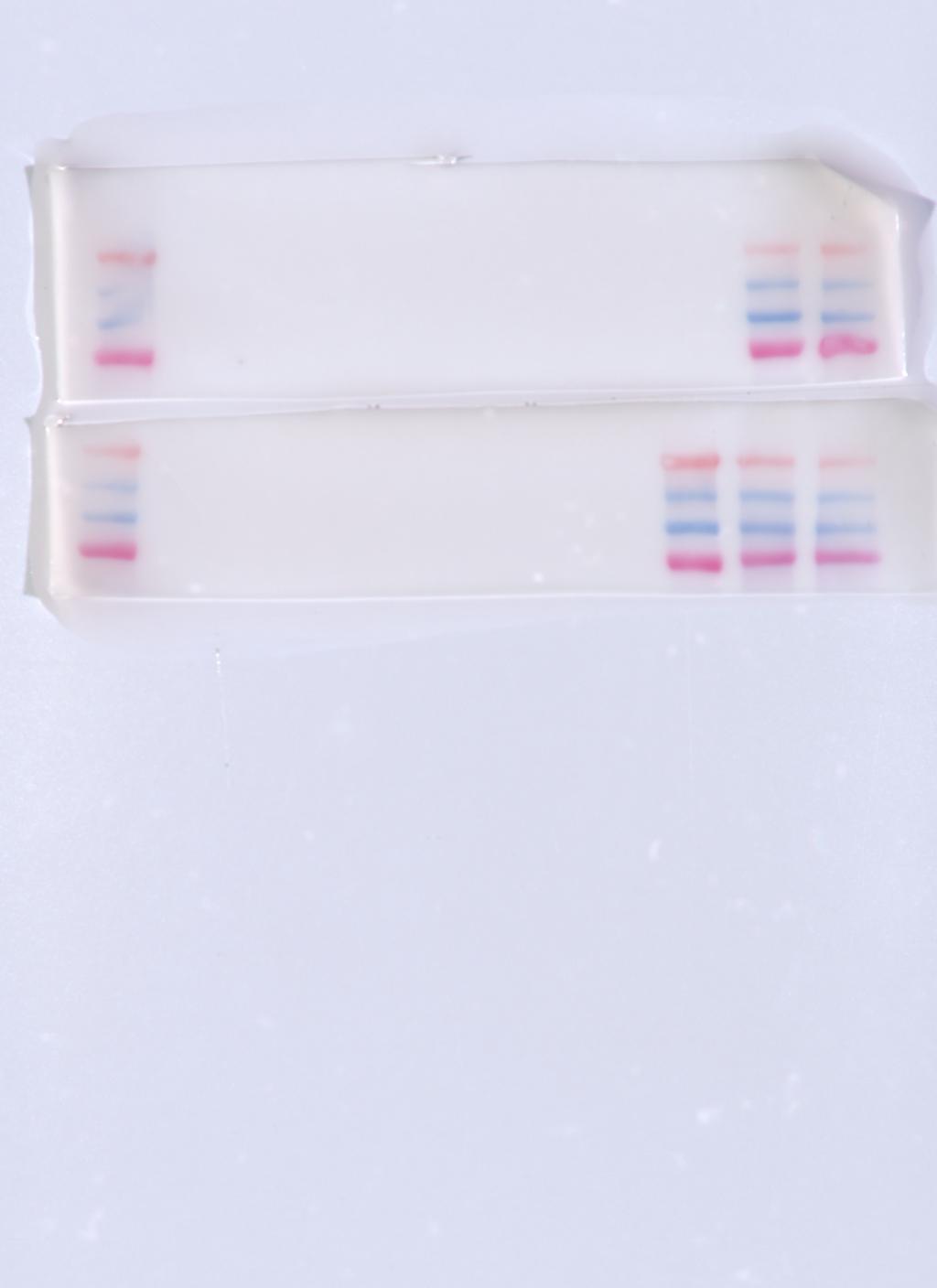

Supplement: Supplementary file 12 — EV Figure Source Data [file 44318_2025_453_MOESM12_ESM.zip › Source data EV-2/Figure EV2/EV2D/GapDH depletion/blm5min 2024.09.02_16.55.43_Ch-Marker.jpg]

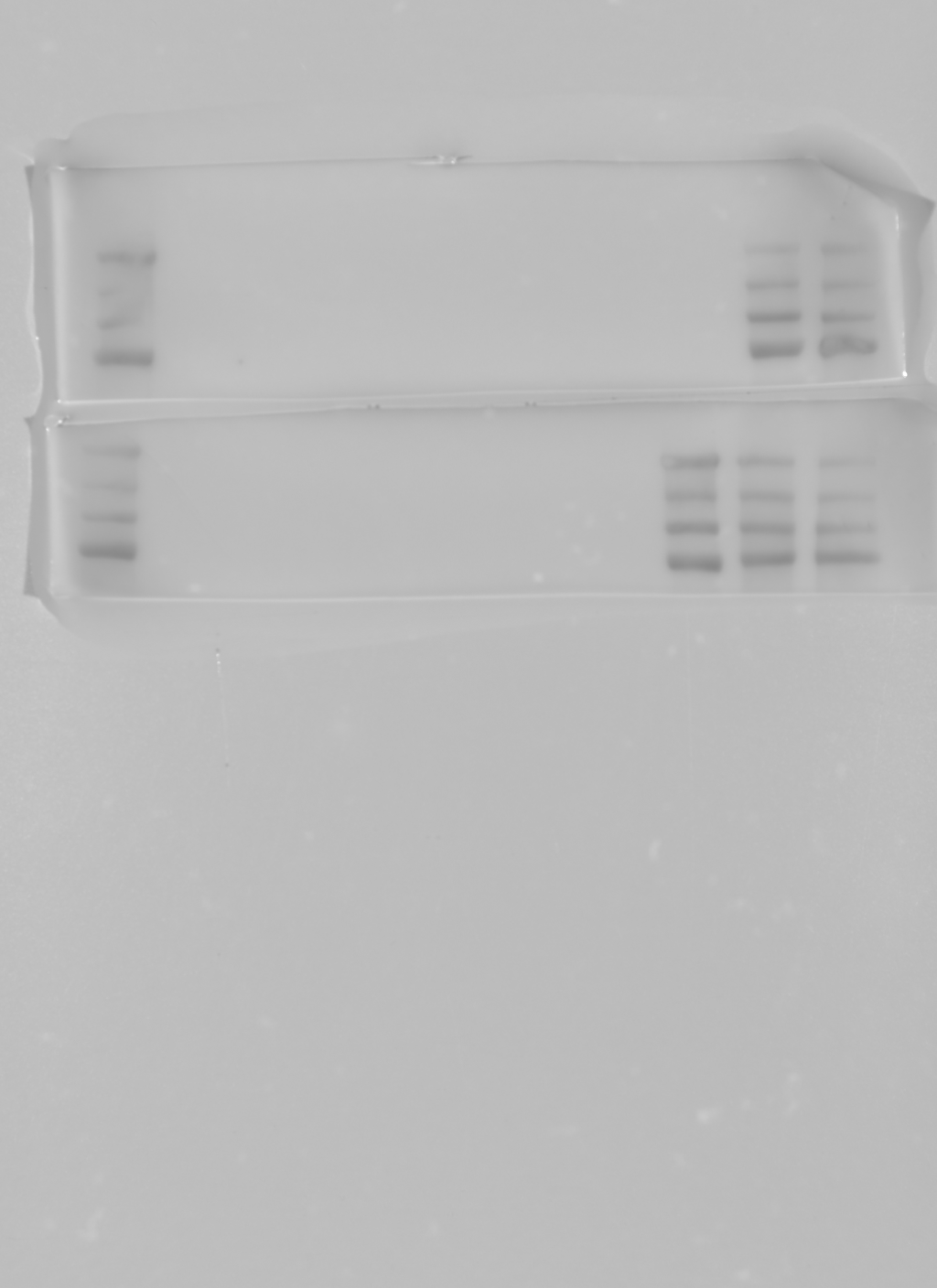

Supplement: Supplementary file 12 — EV Figure Source Data [file 44318_2025_453_MOESM12_ESM.zip › Source data EV-2/Figure EV2/EV2D/GapDH depletion/blm5min 2024.09.02_16.55.43_Ch-Marker.tif]

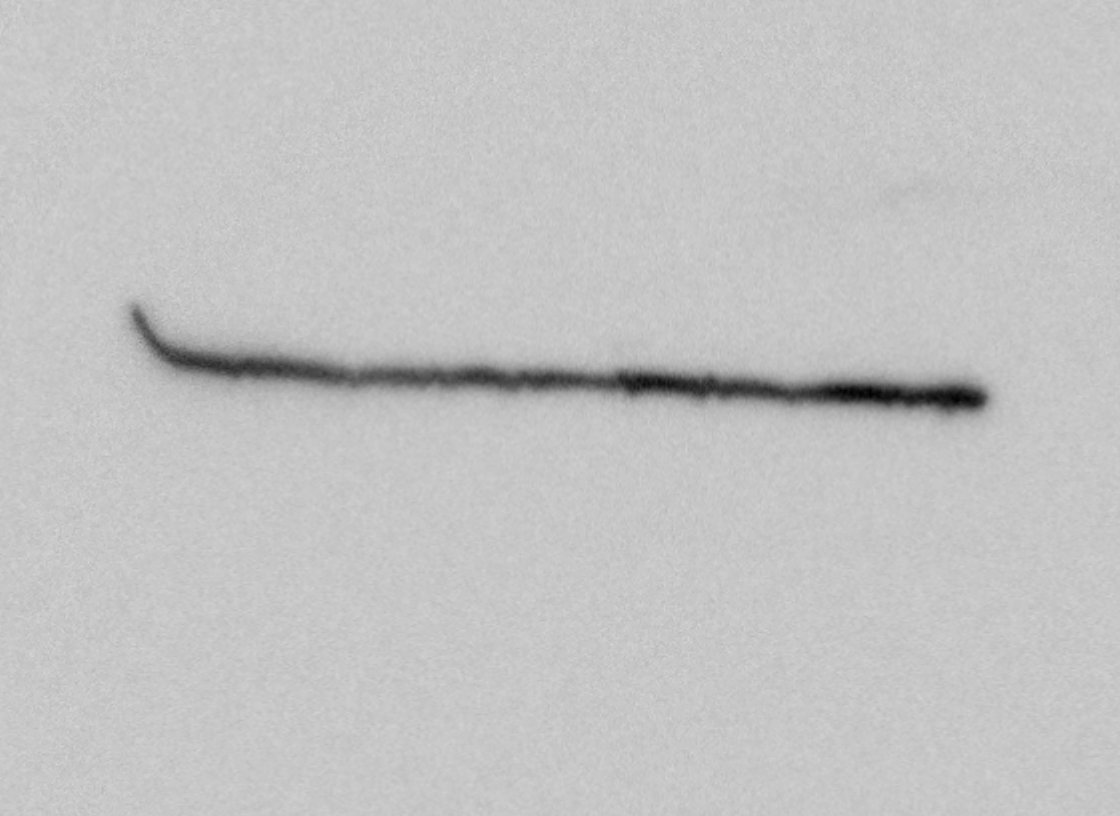

Supplement: Supplementary file 12 — EV Figure Source Data [file 44318_2025_453_MOESM12_ESM.zip › Source data EV-2/Figure EV2/EV2D/gapdh recovery.jpg]

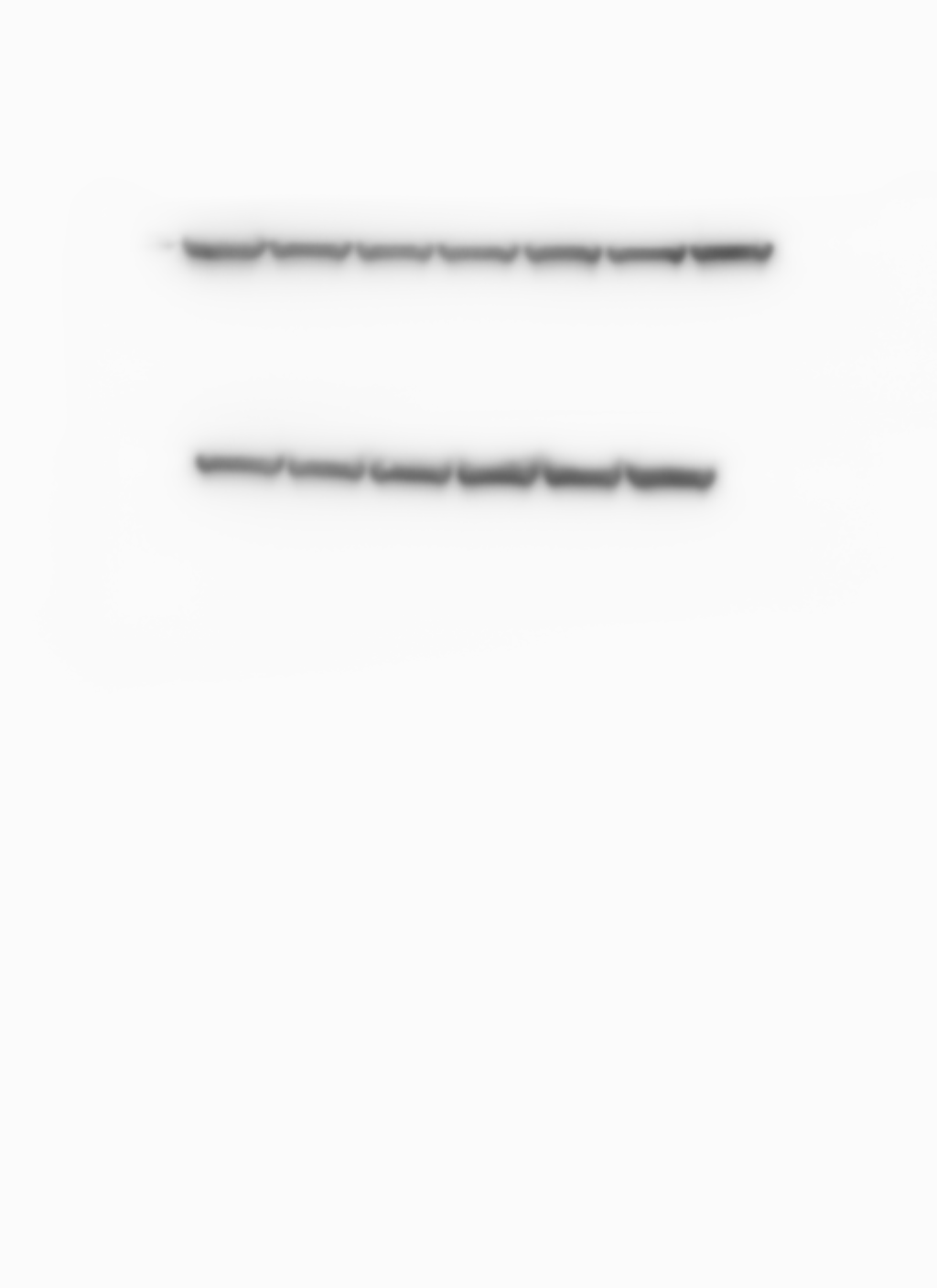

Supplement: Supplementary file 12 — EV Figure Source Data [file 44318_2025_453_MOESM12_ESM.zip › Source data EV-2/Figure EV2/EV2D/GapDH_depletion/gapdh30s 2024.09.02_16.50.56_Ch.tif]

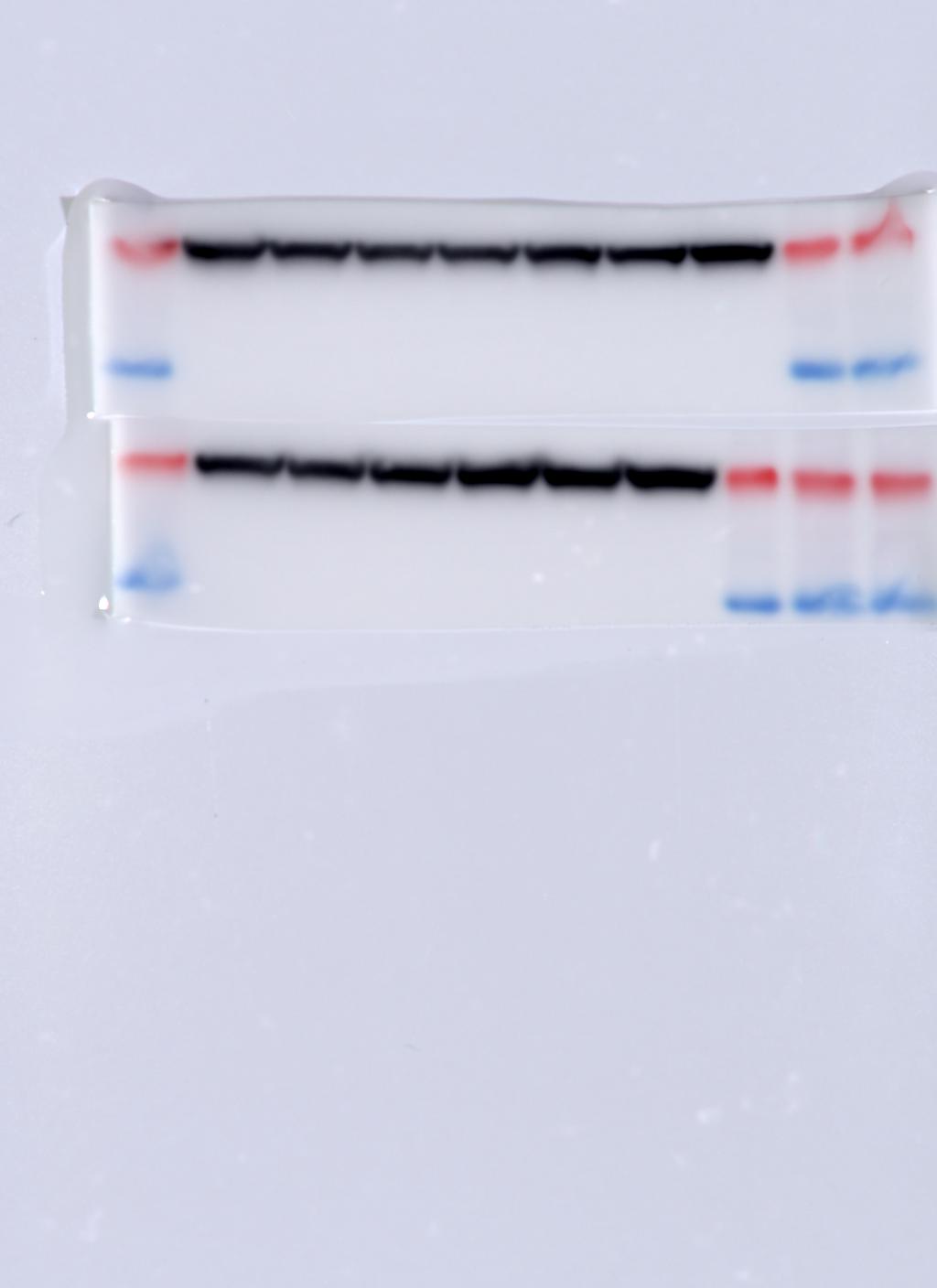

Supplement: Supplementary file 12 — EV Figure Source Data [file 44318_2025_453_MOESM12_ESM.zip › Source data EV-2/Figure EV2/EV2D/GapDH_depletion/gapdh30s 2024.09.02_16.50.56_Ch+Marker.jpg]

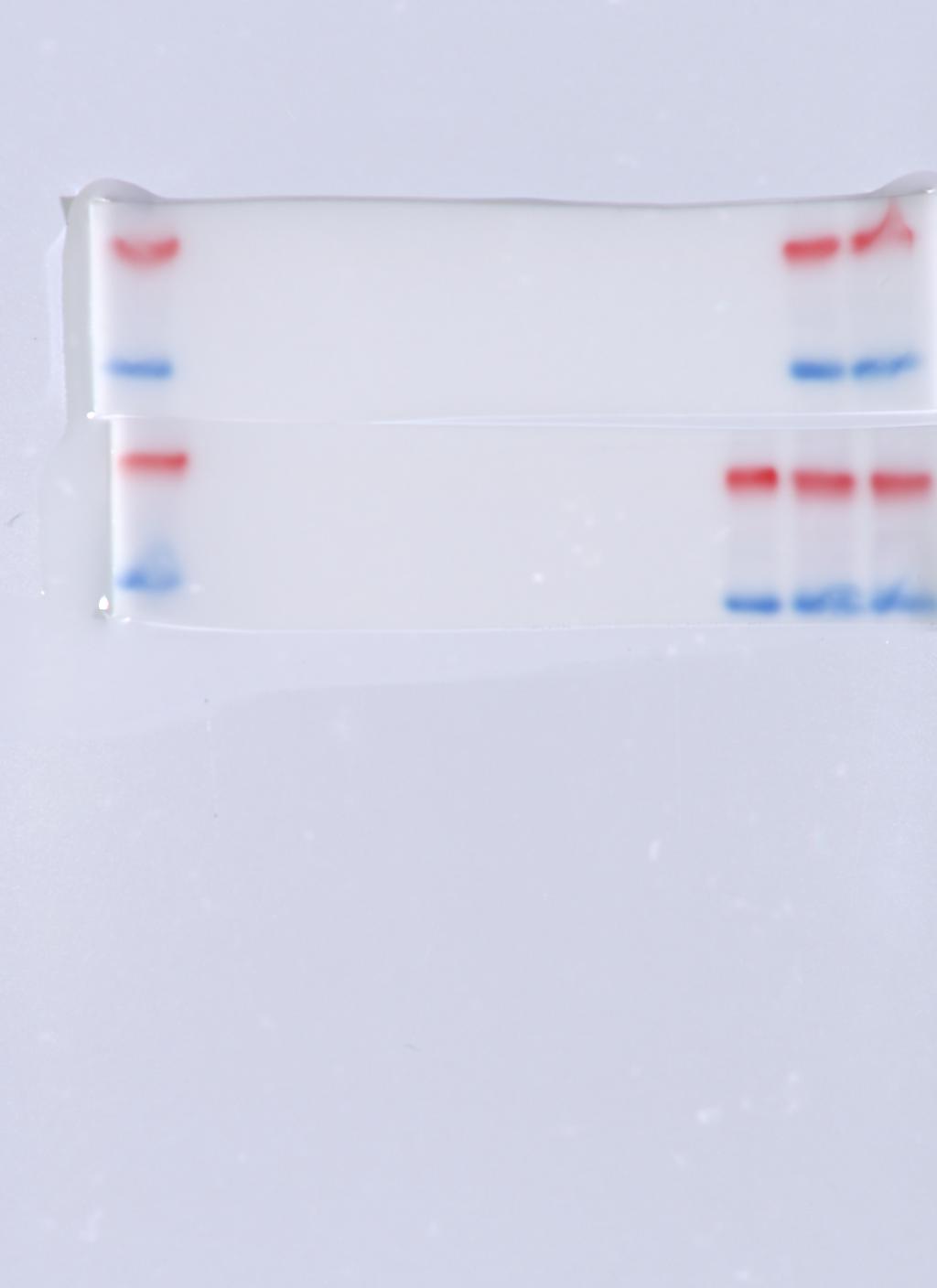

Supplement: Supplementary file 12 — EV Figure Source Data [file 44318_2025_453_MOESM12_ESM.zip › Source data EV-2/Figure EV2/EV2D/GapDH_depletion/gapdh30s 2024.09.02_16.50.56_Ch-Marker.jpg]

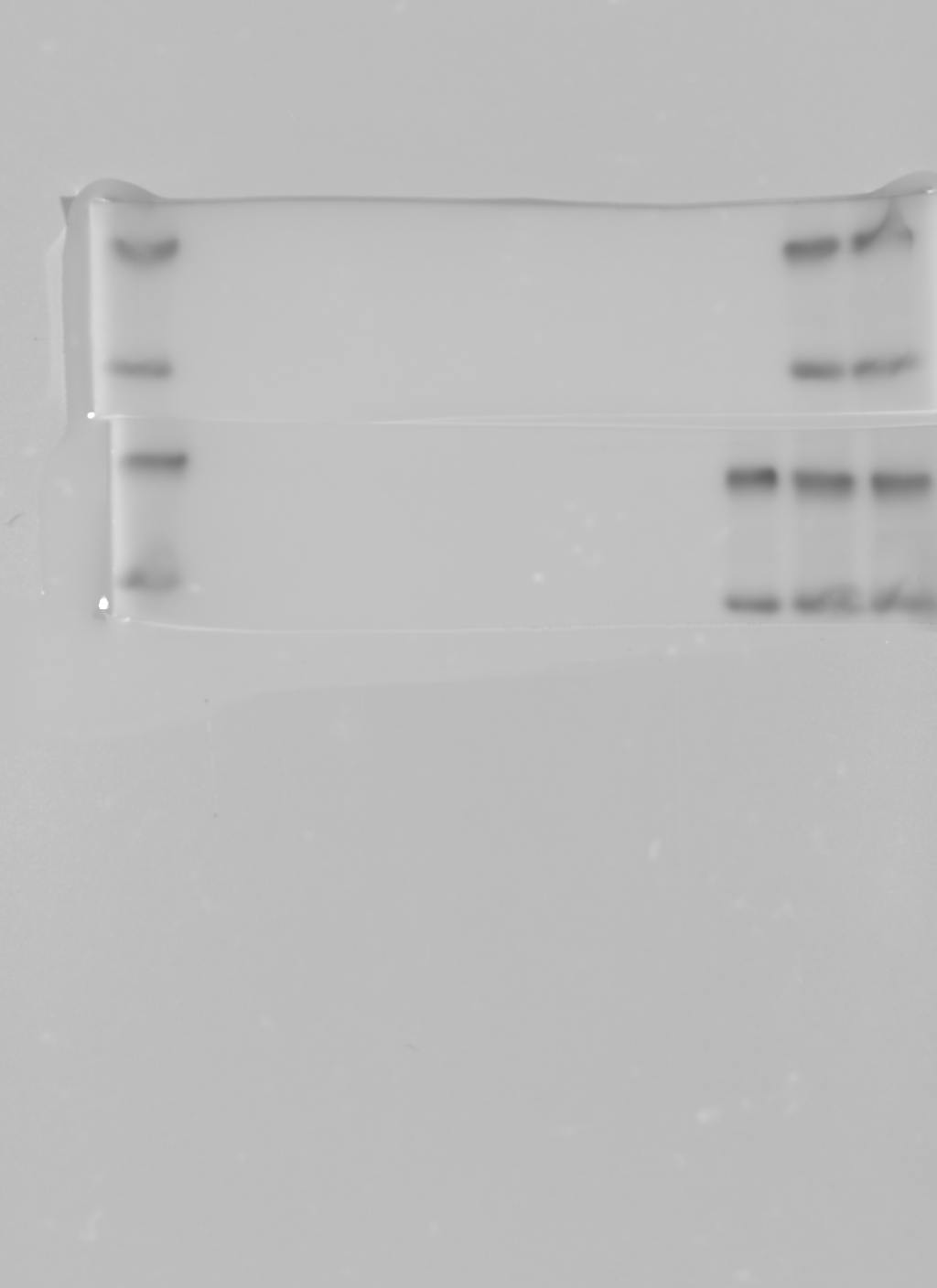

Supplement: Supplementary file 12 — EV Figure Source Data [file 44318_2025_453_MOESM12_ESM.zip › Source data EV-2/Figure EV2/EV2D/GapDH_depletion/gapdh30s 2024.09.02_16.50.56_Ch-Marker.tif]

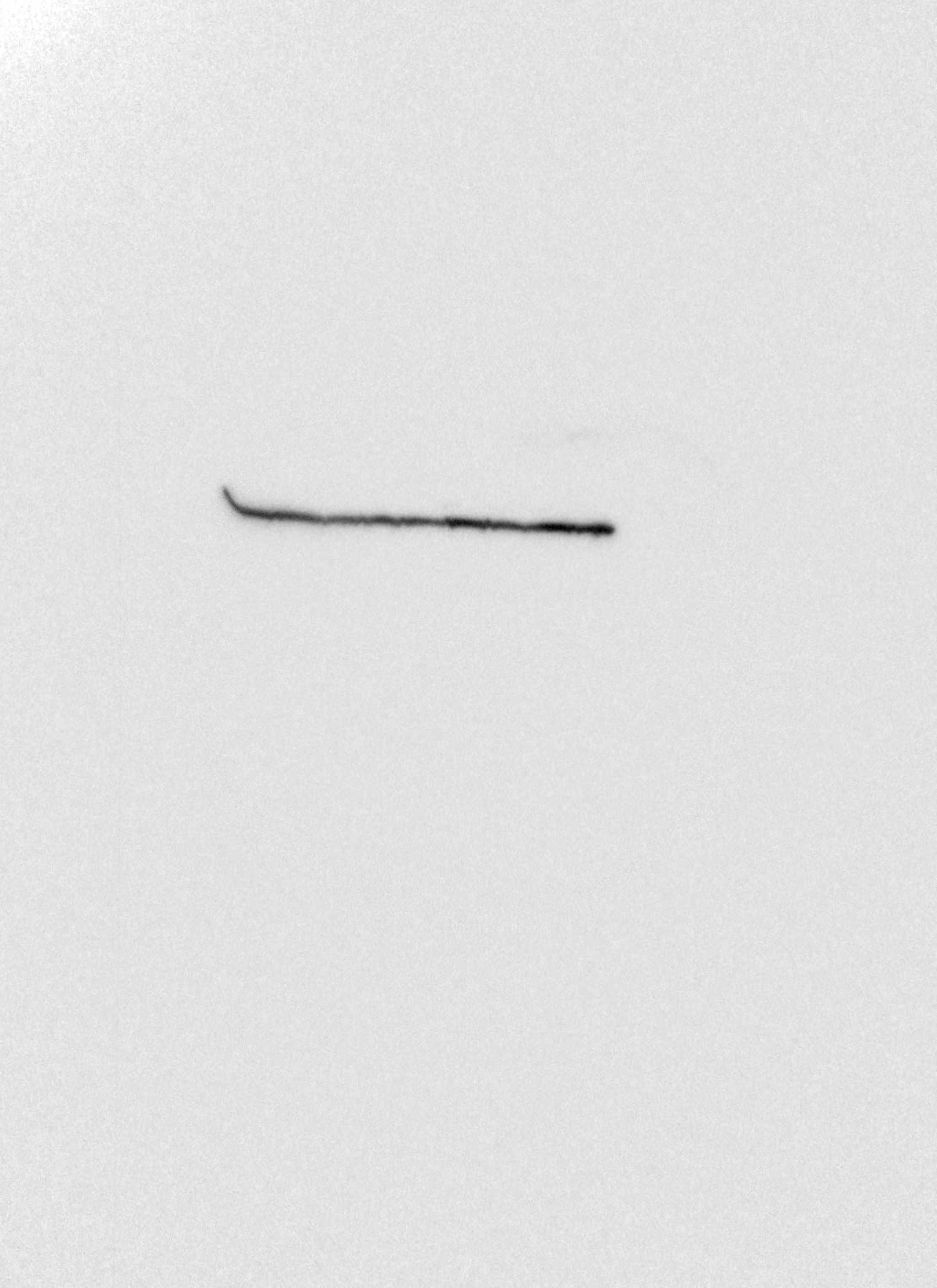

Supplement: Supplementary file 12 — EV Figure Source Data [file 44318_2025_453_MOESM12_ESM.zip › Source data EV-2/Figure EV2/EV2D/GapDH_recovery/misBLM3 2024.11.14_15.29.07_Ch.tif]

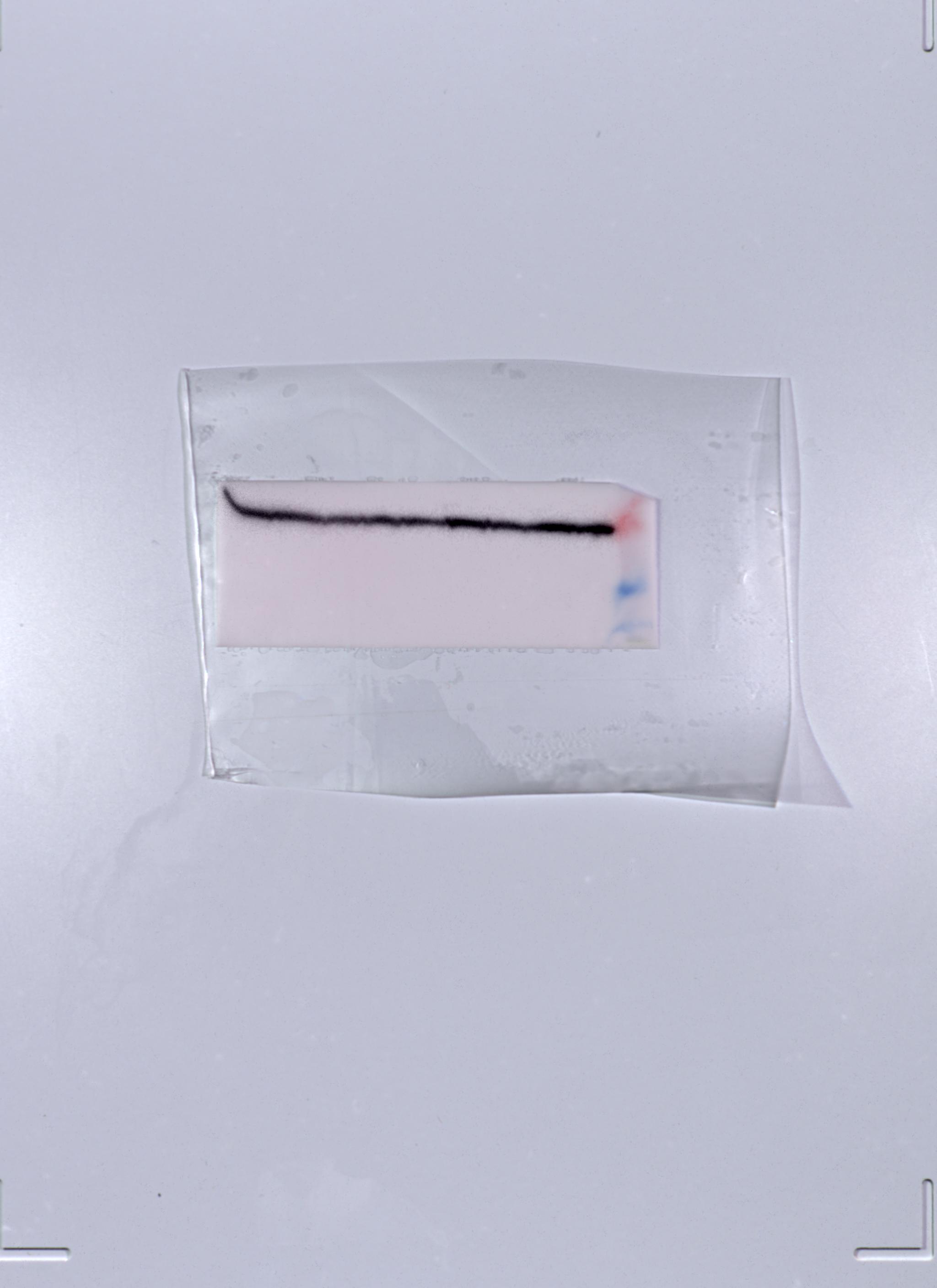

Supplement: Supplementary file 12 — EV Figure Source Data [file 44318_2025_453_MOESM12_ESM.zip › Source data EV-2/Figure EV2/EV2D/GapDH_recovery/misBLM3 2024.11.14_15.29.07_Ch+Marker.jpg]

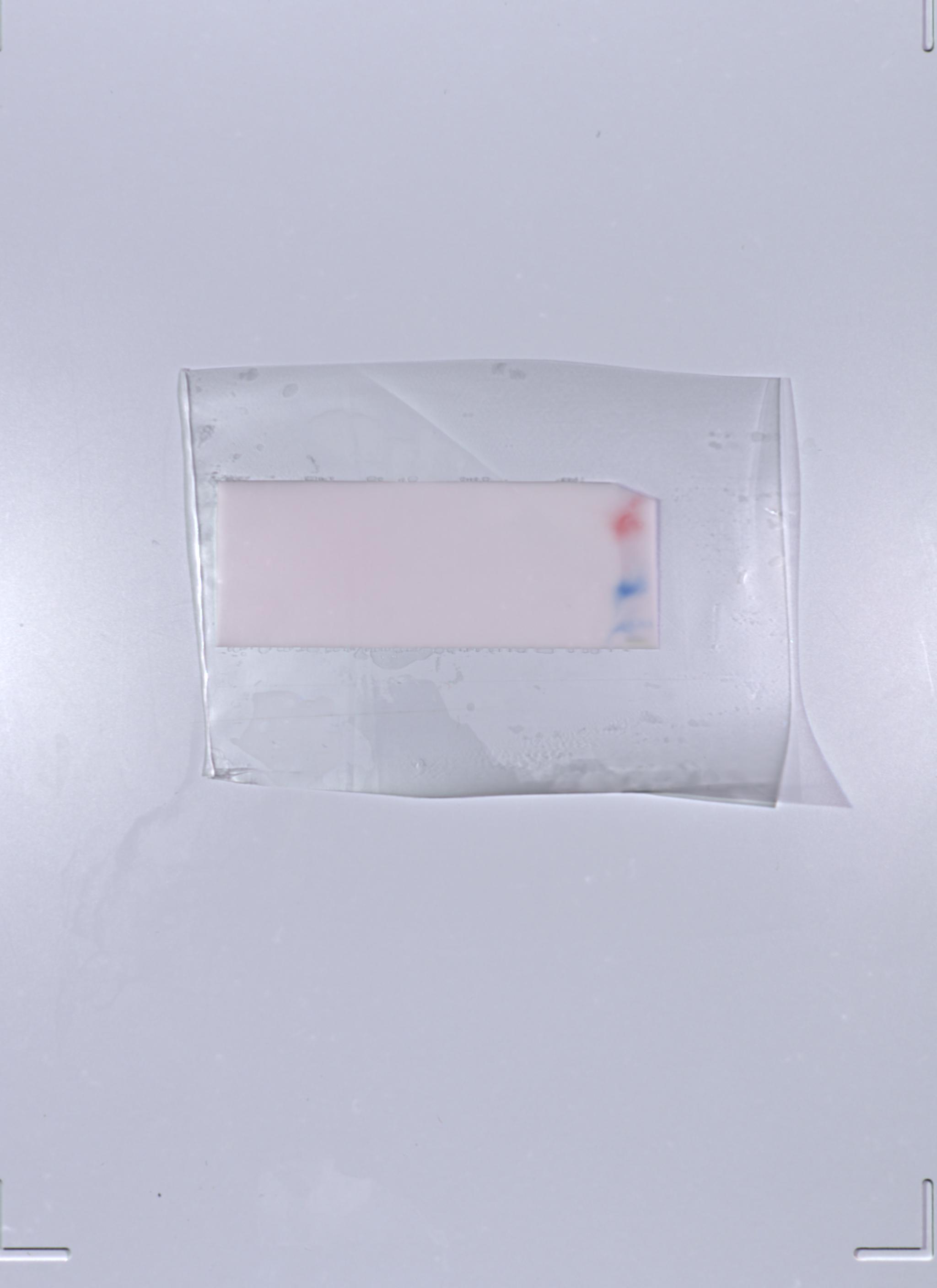

Supplement: Supplementary file 12 — EV Figure Source Data [file 44318_2025_453_MOESM12_ESM.zip › Source data EV-2/Figure EV2/EV2D/GapDH_recovery/misBLM3 2024.11.14_15.29.07_Ch-Marker.jpg]

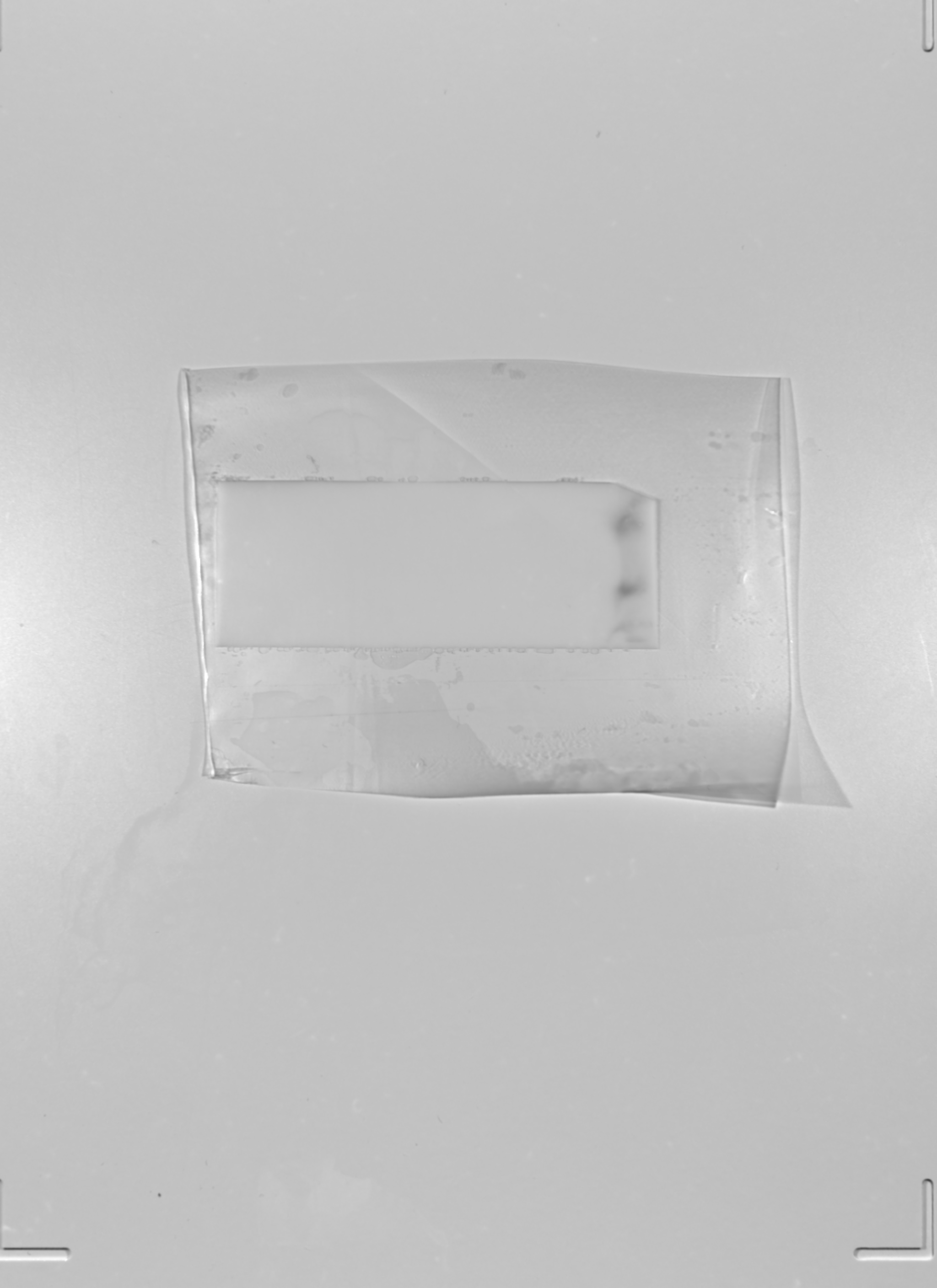

Supplement: Supplementary file 12 — EV Figure Source Data [file 44318_2025_453_MOESM12_ESM.zip › Source data EV-2/Figure EV2/EV2D/GapDH_recovery/misBLM3 2024.11.14_15.29.07_Ch-Marker.tif]

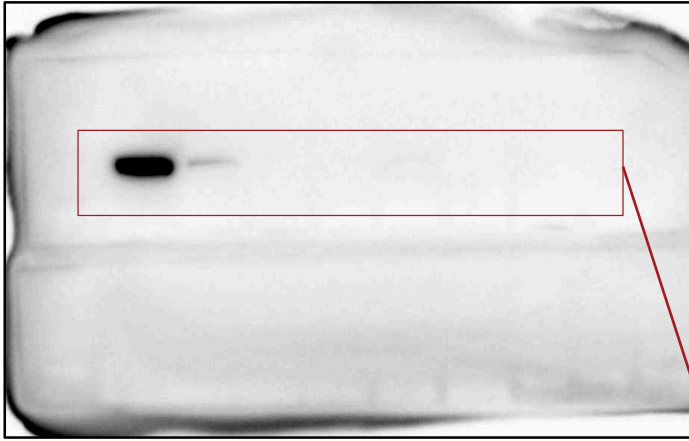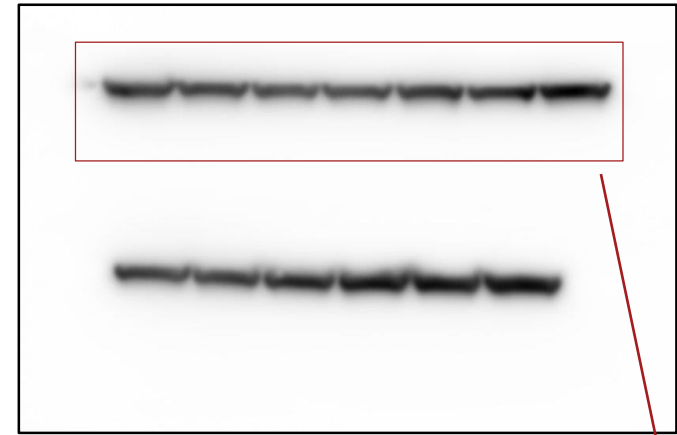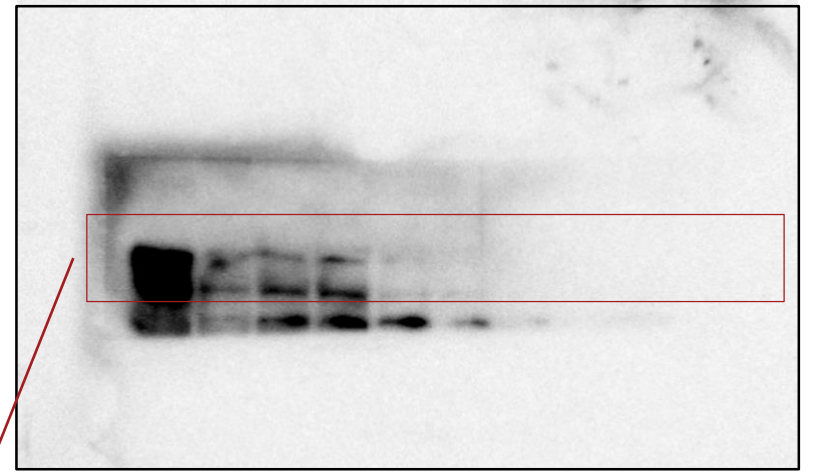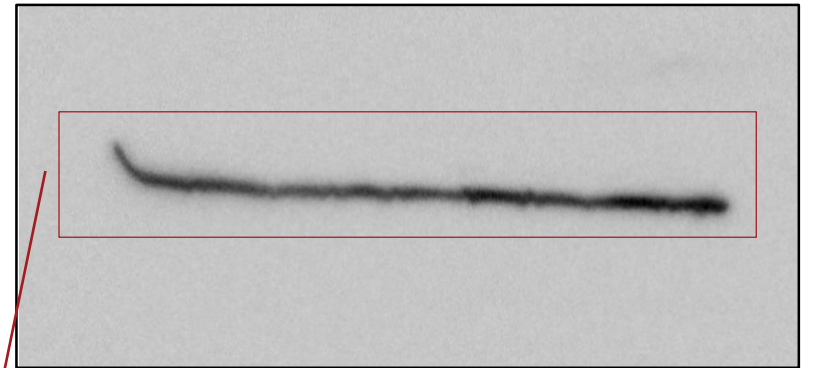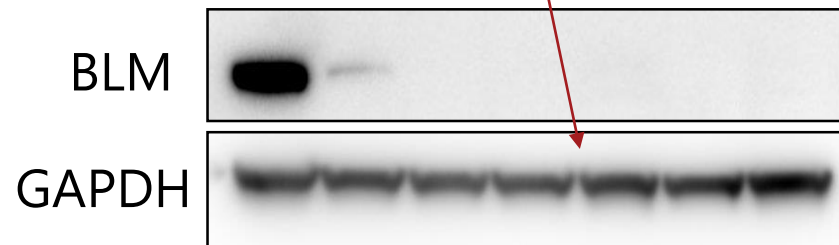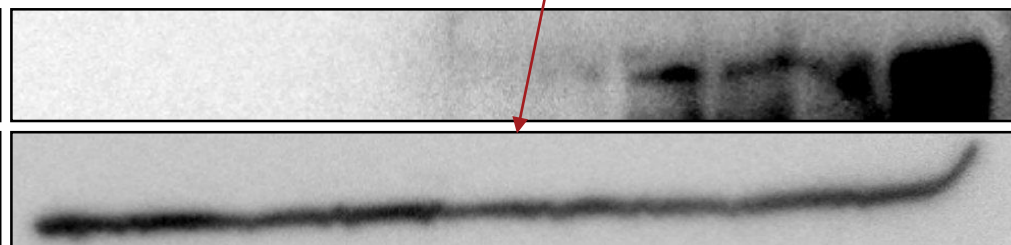

BLM

GAPDH

Supplement: Supplementary file 12 — EV Figure Source Data [file 44318_2025_453_MOESM12_ESM.zip › Source data EV-2/Figure EV2/EV2D/Readme EV2D.pdf]

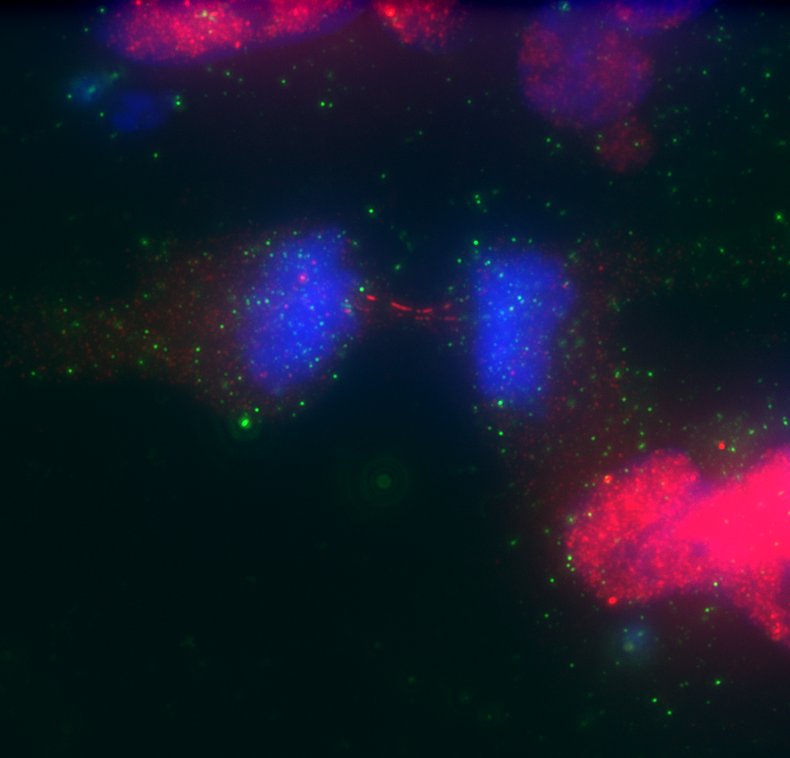

Supplement: Supplementary file 12 — EV Figure Source Data [file 44318_2025_453_MOESM12_ESM.zip › Source data EV-2/Figure EV4/EV4A/bridge wt rpc MB.jpg]

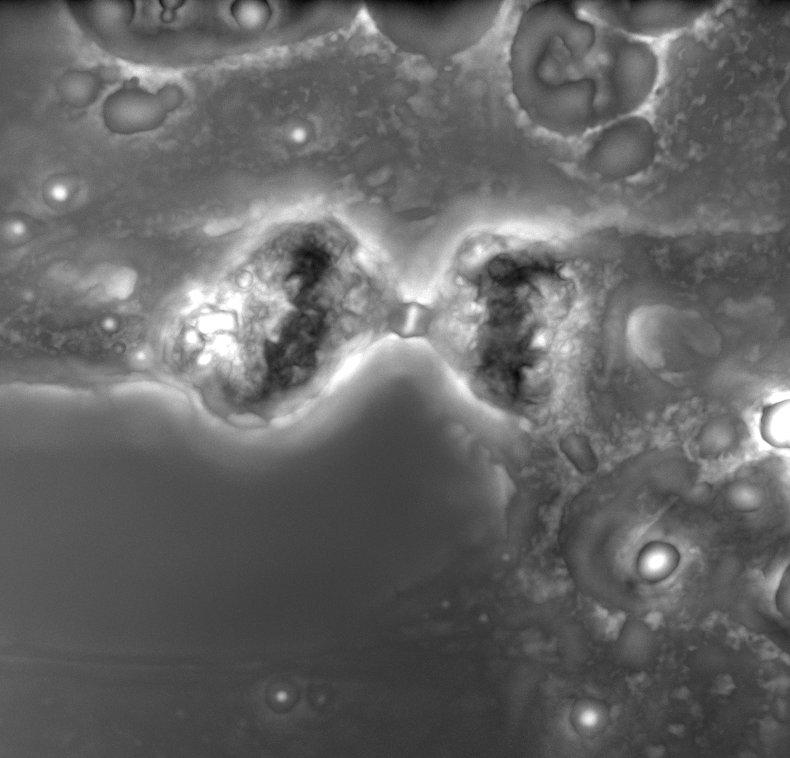

Supplement: Supplementary file 12 — EV Figure Source Data [file 44318_2025_453_MOESM12_ESM.zip › Source data EV-2/Figure EV4/EV4A/bridge wt rpc MB1.jpg]

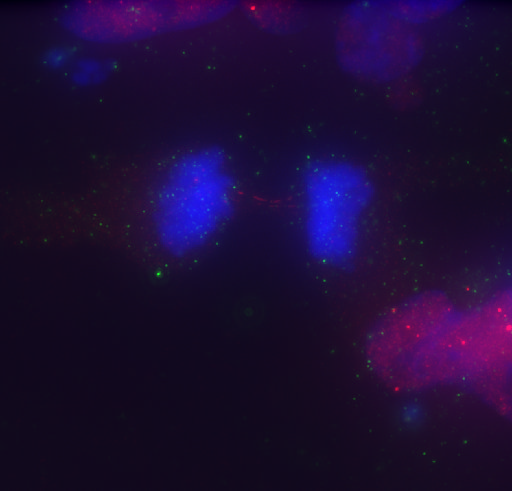

Supplement: Supplementary file 12 — EV Figure Source Data [file 44318_2025_453_MOESM12_ESM.zip › Source data EV-2/Figure EV4/EV4A/WT aph_39_Maximum_Z.vsi]

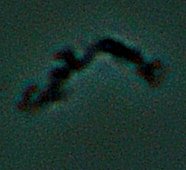

Supplement: Supplementary file 12 — EV Figure Source Data [file 44318_2025_453_MOESM12_ESM.zip › Source data EV-2/Figure EV5/EV5A/BLM KO_1.jpg]

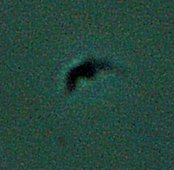

Supplement: Supplementary file 12 — EV Figure Source Data [file 44318_2025_453_MOESM12_ESM.zip › Source data EV-2/Figure EV5/EV5A/BLM KO_2.jpg]

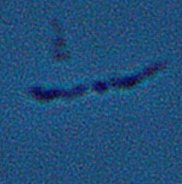

Supplement: Supplementary file 12 — EV Figure Source Data [file 44318_2025_453_MOESM12_ESM.zip › Source data EV-2/Figure EV5/EV5A/BLM WT_1.jpg]

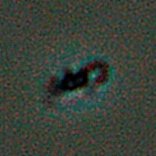

Supplement: Supplementary file 12 — EV Figure Source Data [file 44318_2025_453_MOESM12_ESM.zip › Source data EV-2/Figure EV5/EV5A/BLM WT_2.jpg]

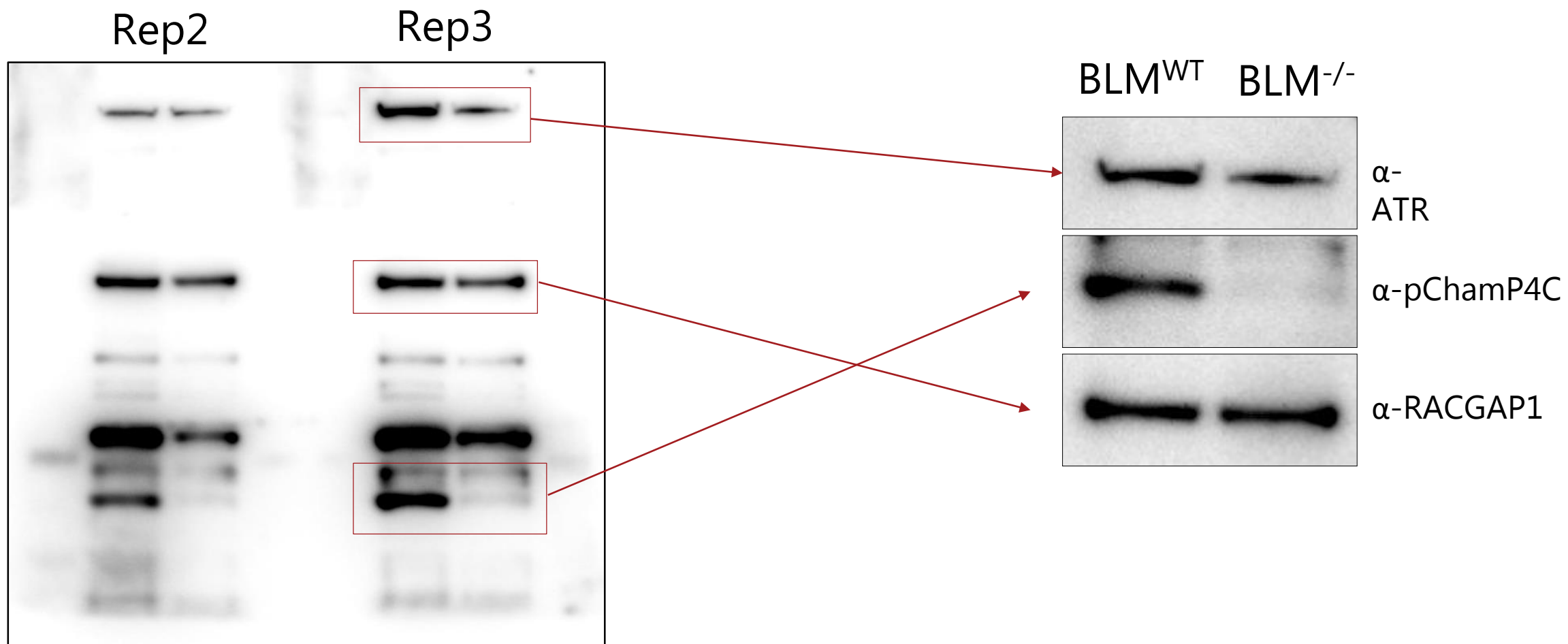

Supplement: Supplementary file 12 — EV Figure Source Data [file 44318_2025_453_MOESM12_ESM.zip › Source data EV-2/Figure EV5/EV5B/Read me EV5B.pdf]

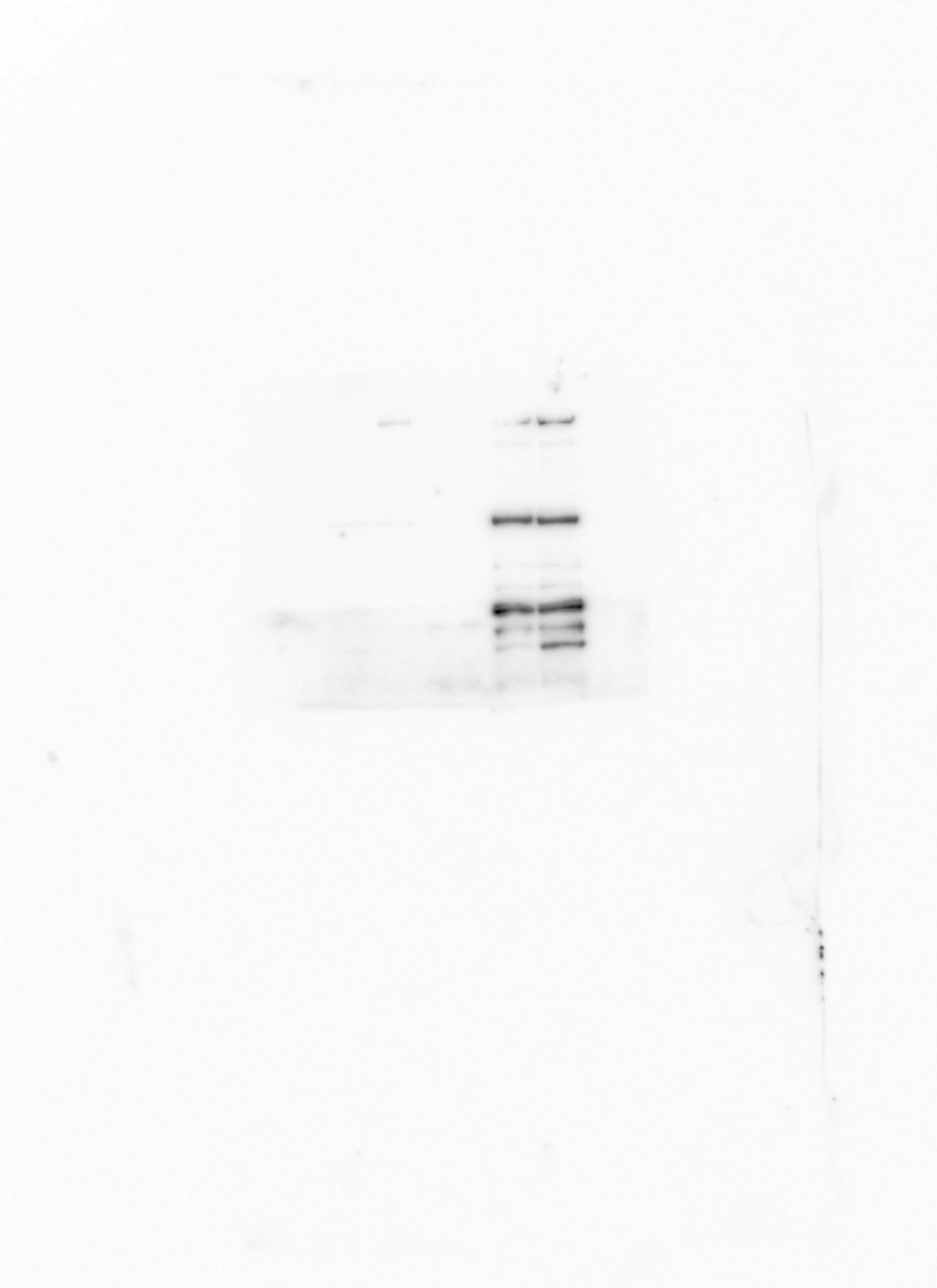

Supplement: Supplementary file 12 — EV Figure Source Data [file 44318_2025_453_MOESM12_ESM.zip › Source data EV-2/Figure EV5/EV5B/Rep1/manikachamp4c gel3b 2024.05.17_15.06.04_Ch/manikachamp4c gel3b 2024.05.17_15.06.04_Ch.tif]

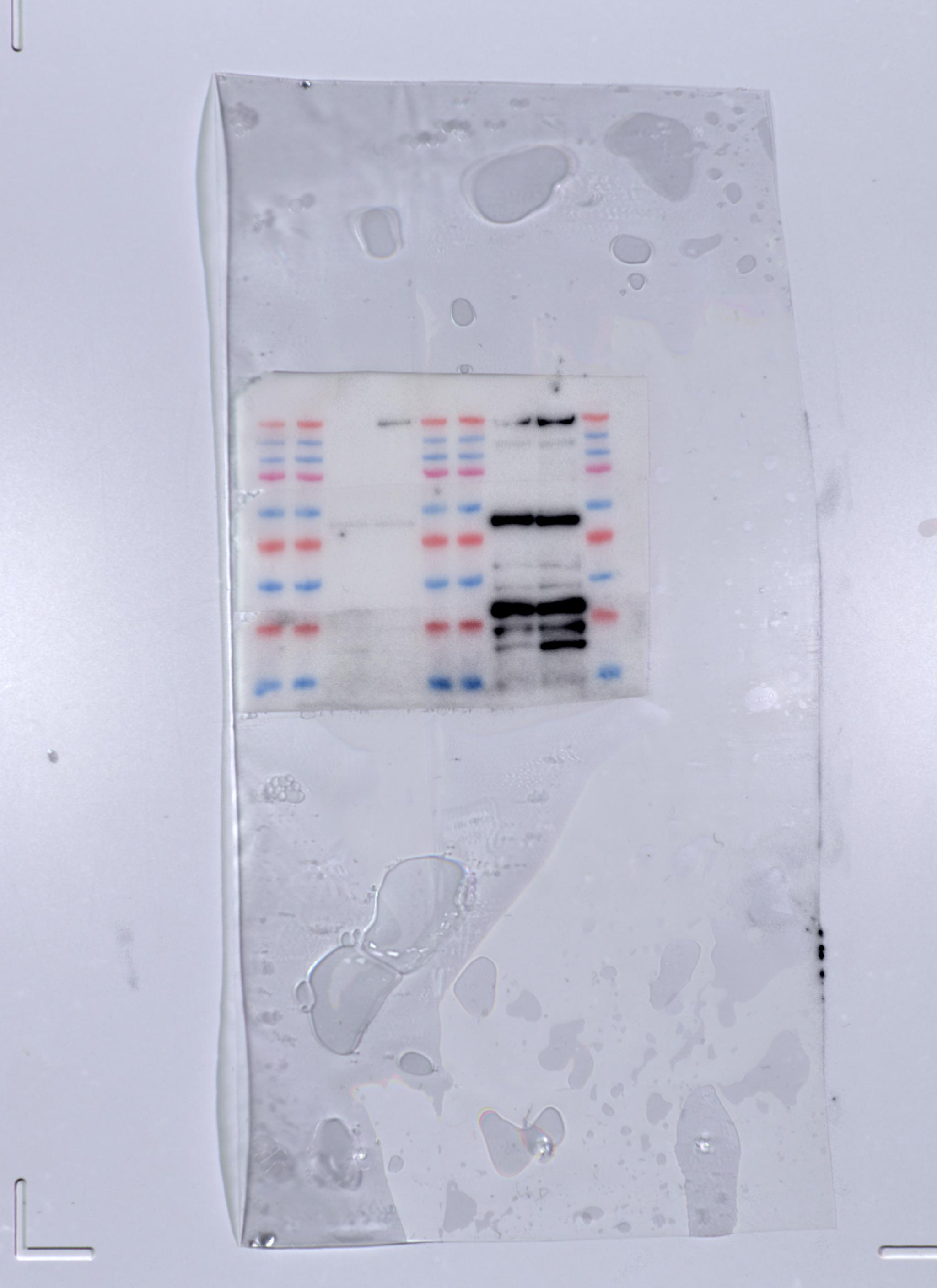

Supplement: Supplementary file 12 — EV Figure Source Data [file 44318_2025_453_MOESM12_ESM.zip › Source data EV-2/Figure EV5/EV5B/Rep1/manikachamp4c gel3b 2024.05.17_15.06.04_Ch/manikachamp4c gel3b 2024.05.17_15.06.04_Ch+Marker.jpg]

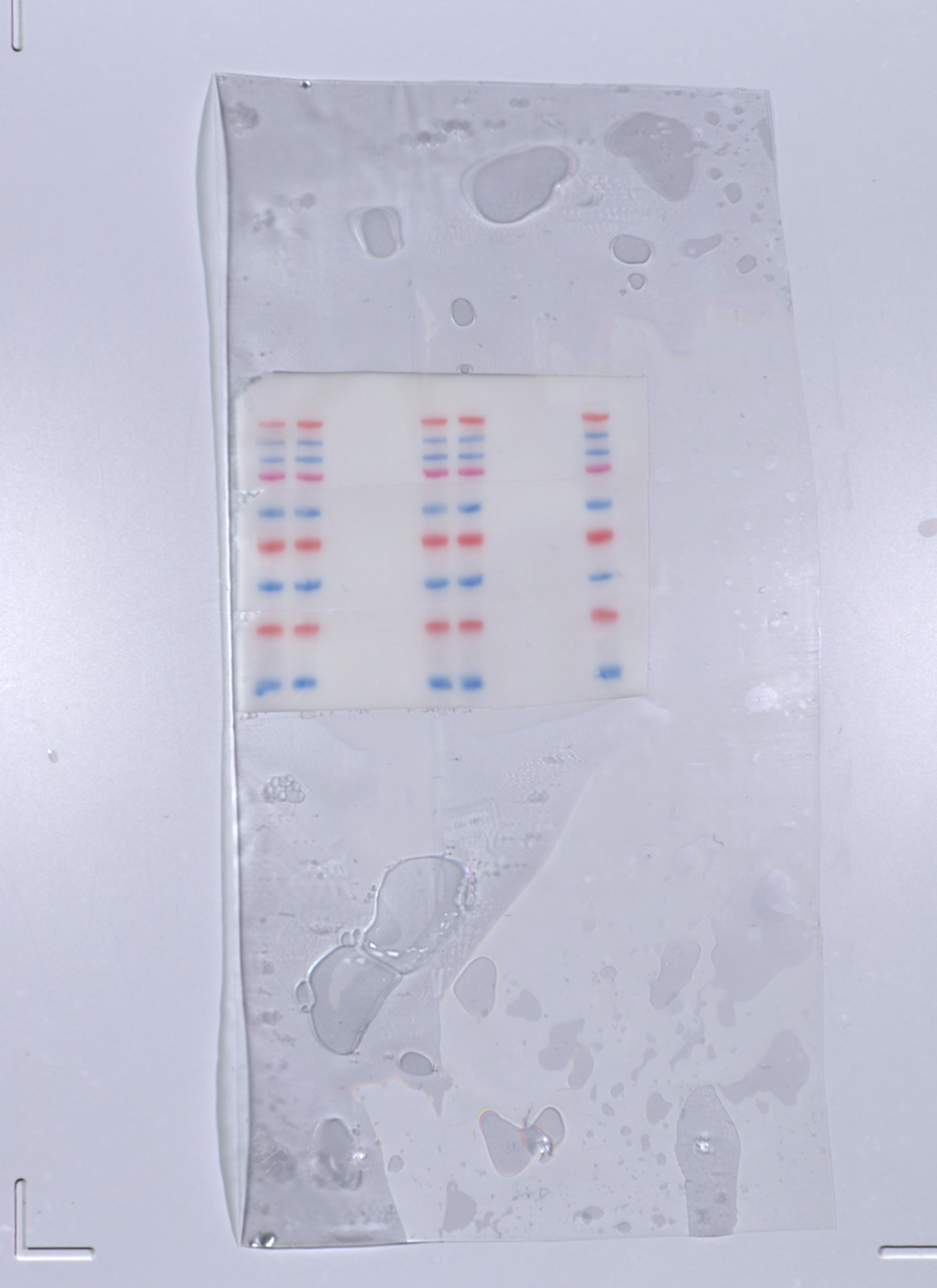

Supplement: Supplementary file 12 — EV Figure Source Data [file 44318_2025_453_MOESM12_ESM.zip › Source data EV-2/Figure EV5/EV5B/Rep1/manikachamp4c gel3b 2024.05.17_15.06.04_Ch/manikachamp4c gel3b 2024.05.17_15.06.04_Ch-Marker.jpg]

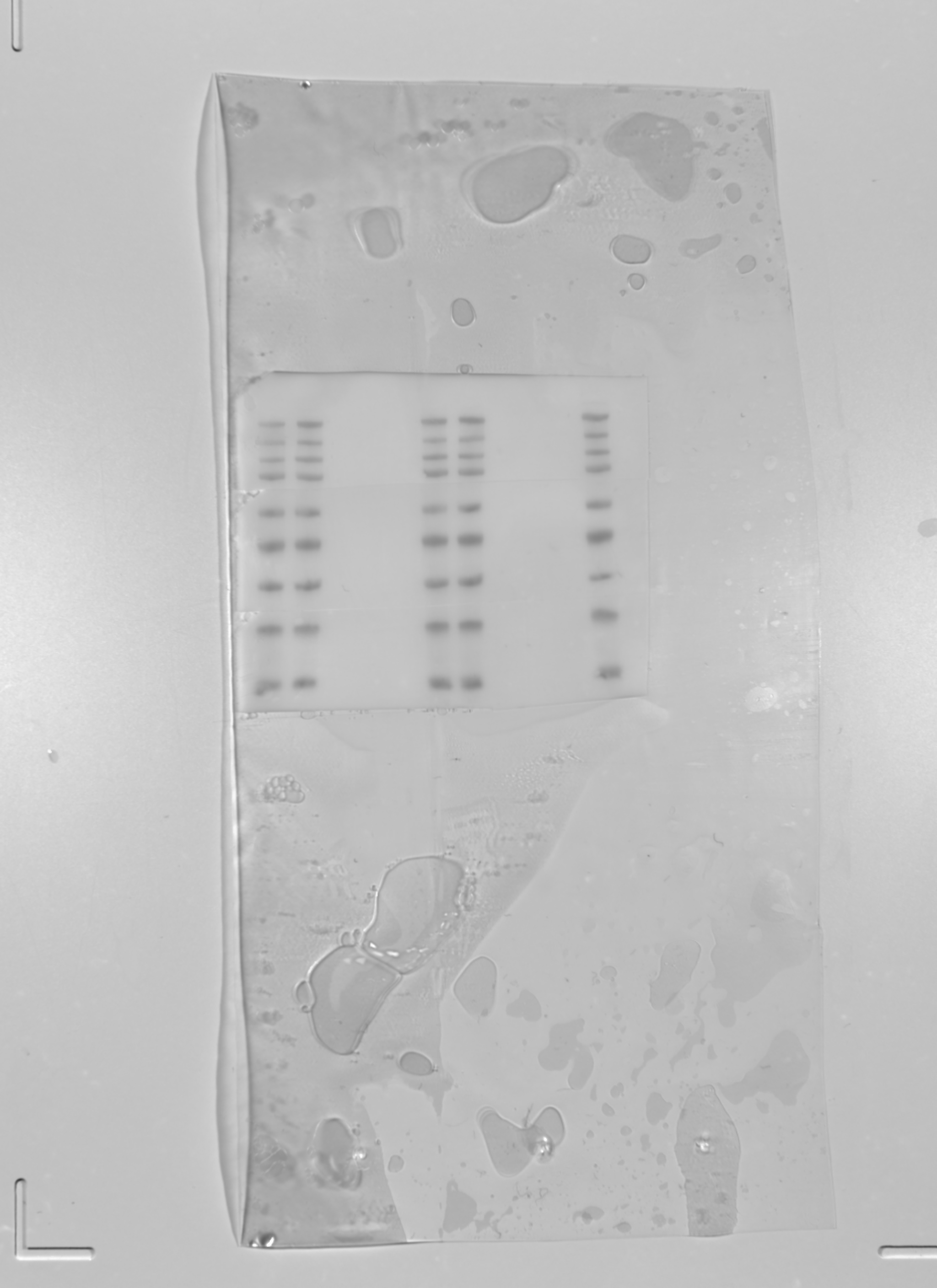

Supplement: Supplementary file 12 — EV Figure Source Data [file 44318_2025_453_MOESM12_ESM.zip › Source data EV-2/Figure EV5/EV5B/Rep1/manikachamp4c gel3b 2024.05.17_15.06.04_Ch/manikachamp4c gel3b 2024.05.17_15.06.04_Ch-Marker.tif]

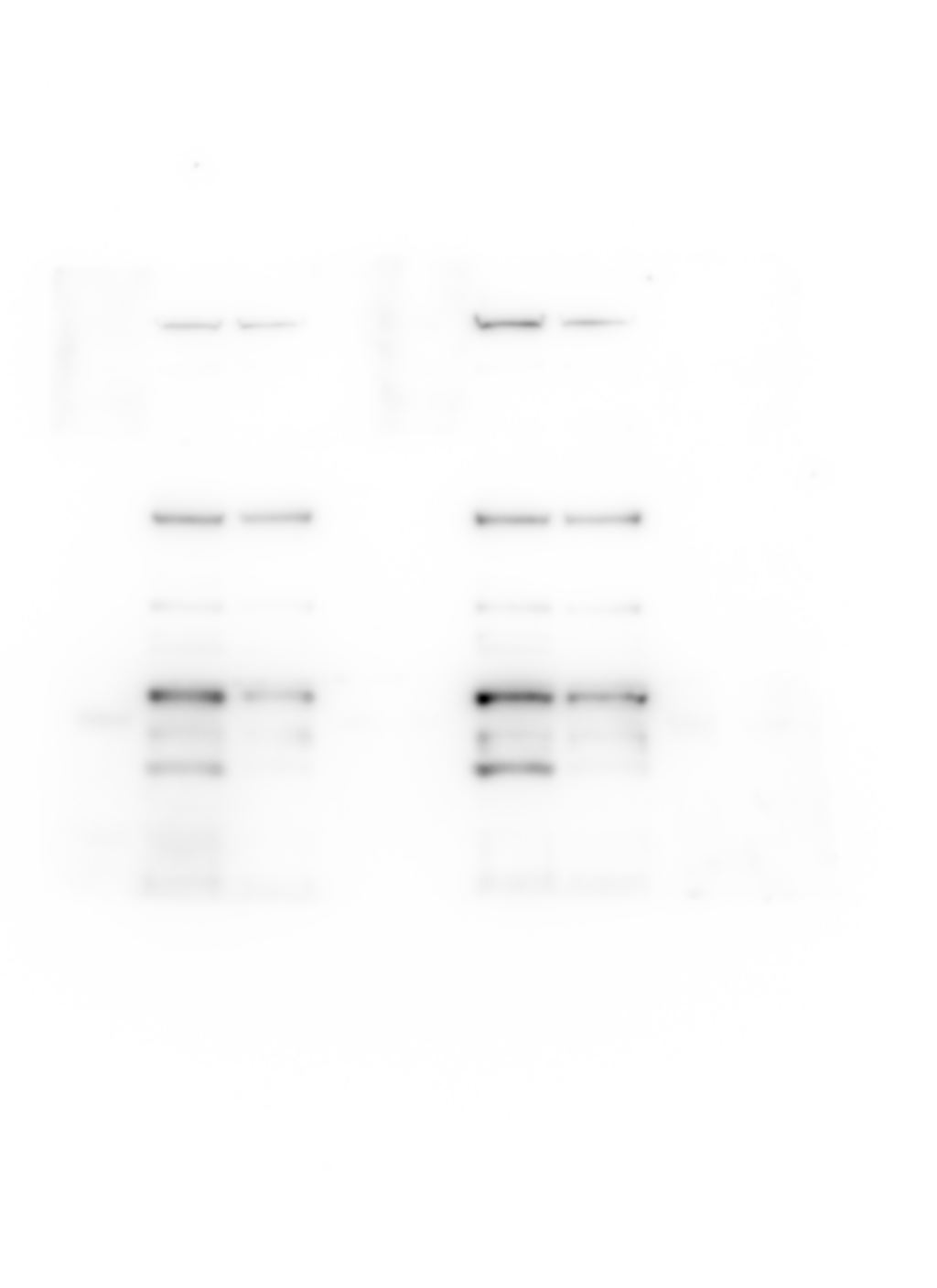

Supplement: Supplementary file 12 — EV Figure Source Data [file 44318_2025_453_MOESM12_ESM.zip › Source data EV-2/Figure EV5/EV5B/Rep2 and 3/manika gel4 f 2024.05.23_16.36.21_Ch/manika gel4 f 2024.05.23_16.36.21_Ch.tif]

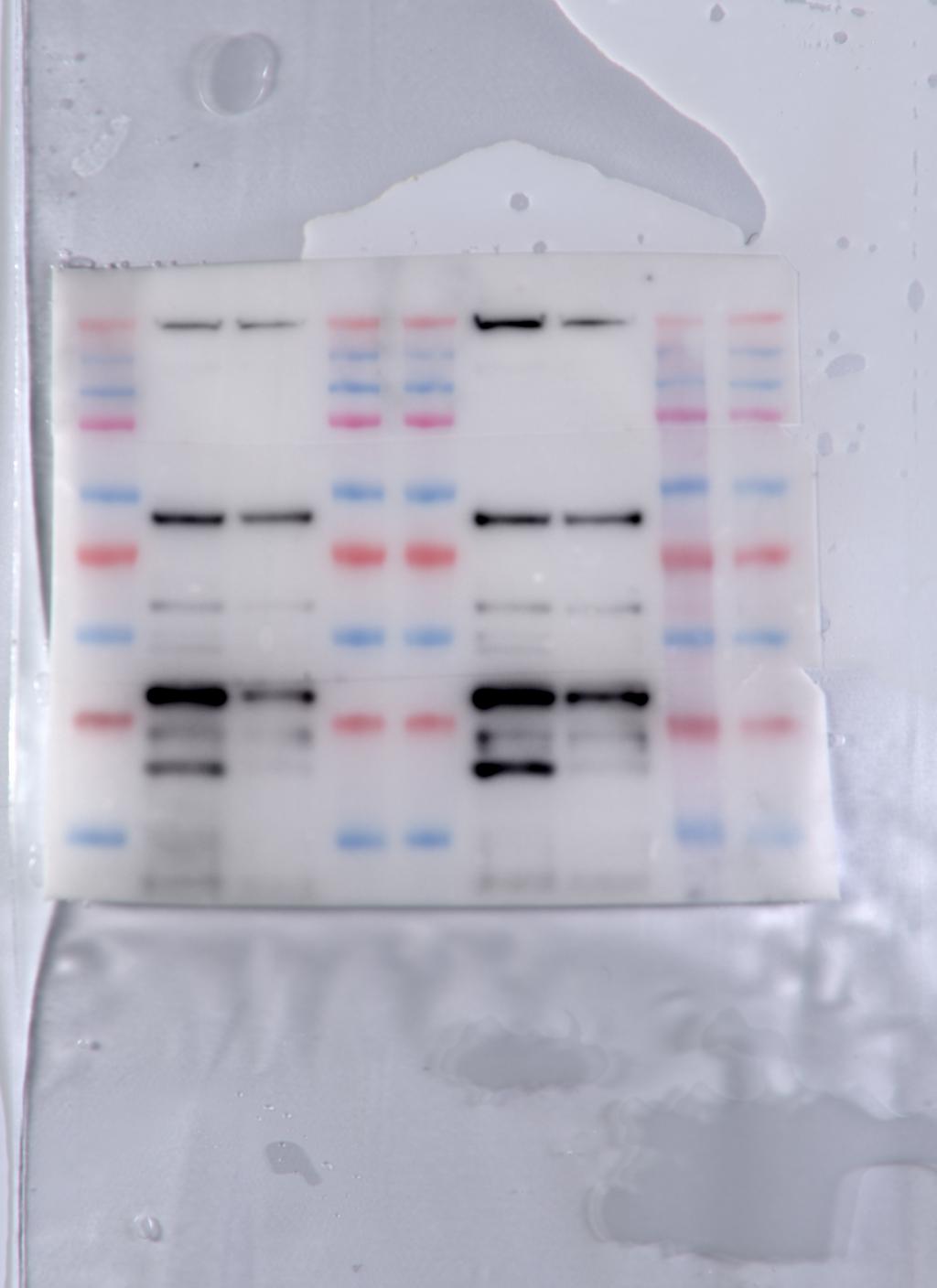

Supplement: Supplementary file 12 — EV Figure Source Data [file 44318_2025_453_MOESM12_ESM.zip › Source data EV-2/Figure EV5/EV5B/Rep2 and 3/manika gel4 f 2024.05.23_16.36.21_Ch/manika gel4 f 2024.05.23_16.36.21_Ch+Marker.jpg]

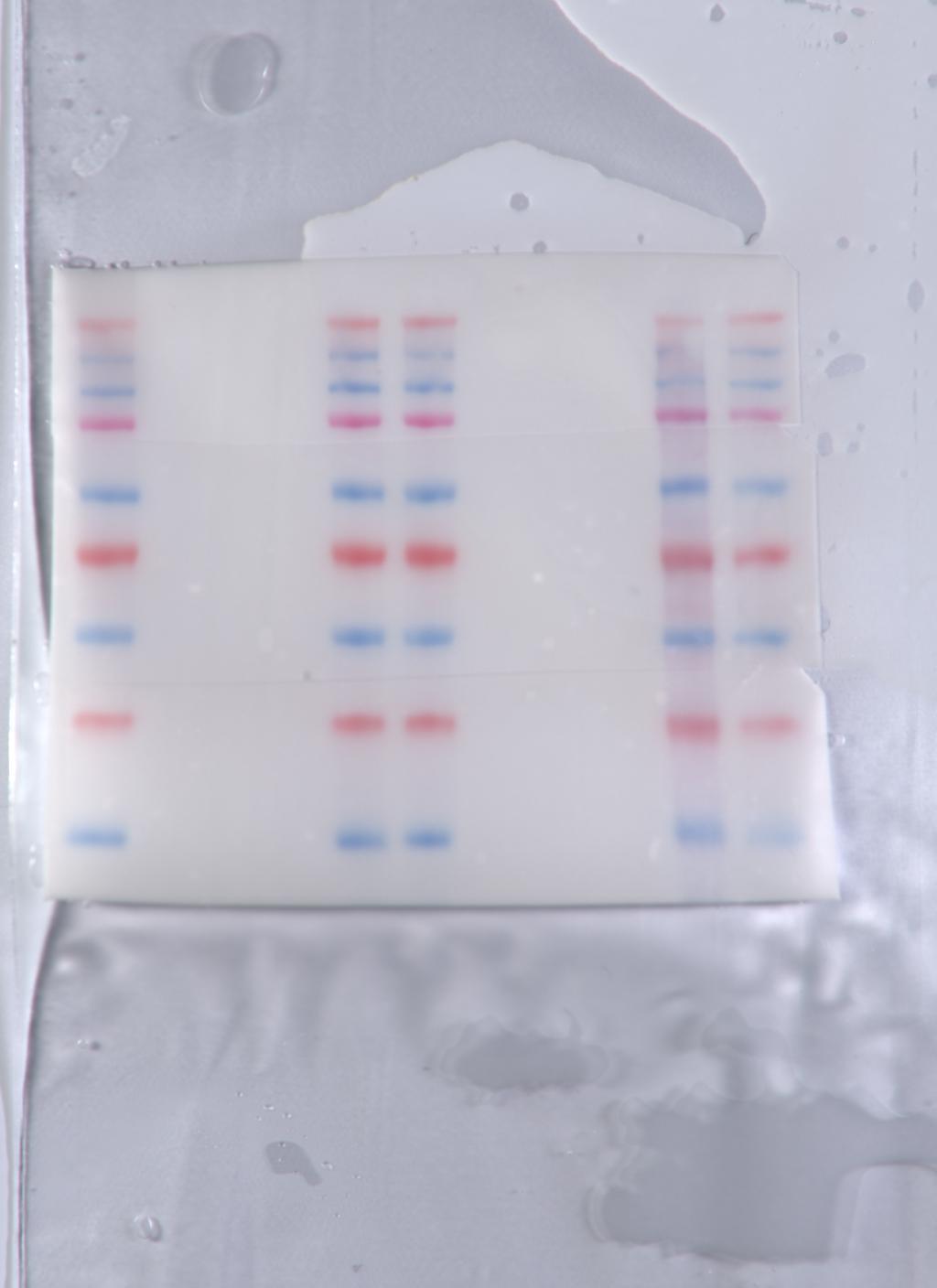

Supplement: Supplementary file 12 — EV Figure Source Data [file 44318_2025_453_MOESM12_ESM.zip › Source data EV-2/Figure EV5/EV5B/Rep2 and 3/manika gel4 f 2024.05.23_16.36.21_Ch/manika gel4 f 2024.05.23_16.36.21_Ch-Marker.jpg]

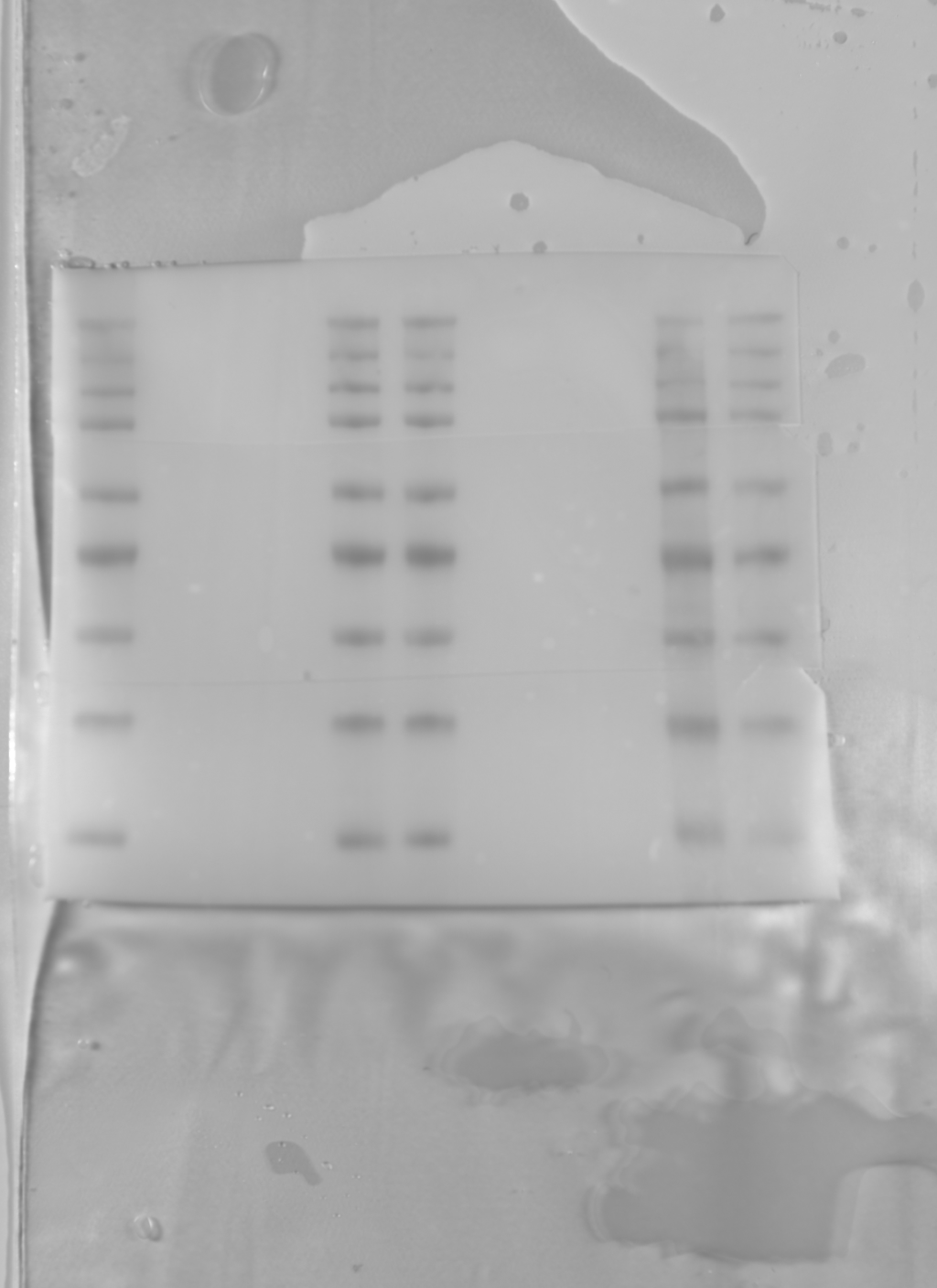

Supplement: Supplementary file 12 — EV Figure Source Data [file 44318_2025_453_MOESM12_ESM.zip › Source data EV-2/Figure EV5/EV5B/Rep2 and 3/manika gel4 f 2024.05.23_16.36.21_Ch/manika gel4 f 2024.05.23_16.36.21_Ch-Marker.tif]

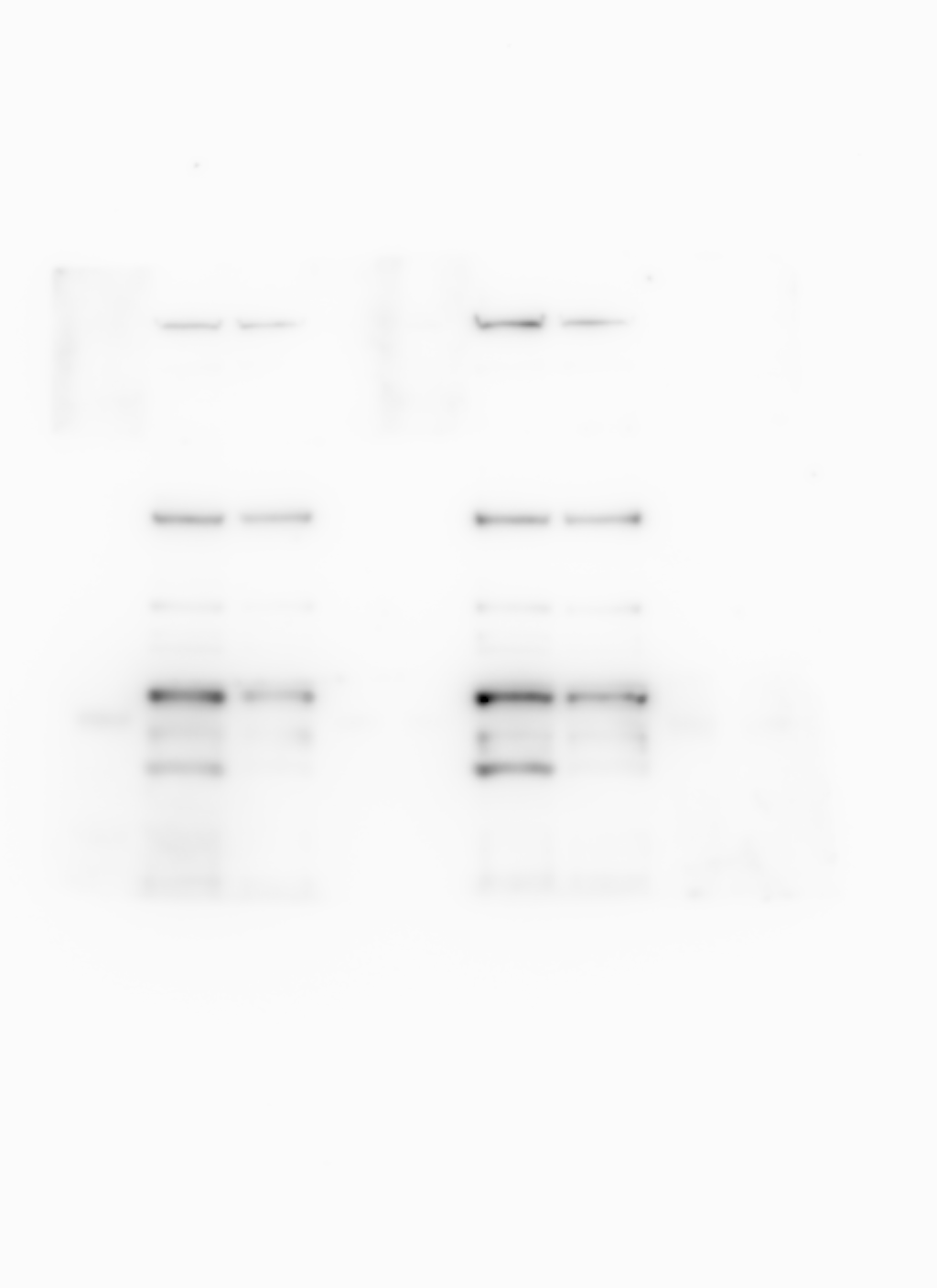

Supplement: Supplementary file 12 — EV Figure Source Data [file 44318_2025_453_MOESM12_ESM.zip › Source data EV-2/Figure EV5/EV5B/Rep2 and 3/manika gel4 g 2024.05.23_16.39.51_Ch/manika gel4 g 2024.05.23_16.39.51_Ch.tif]

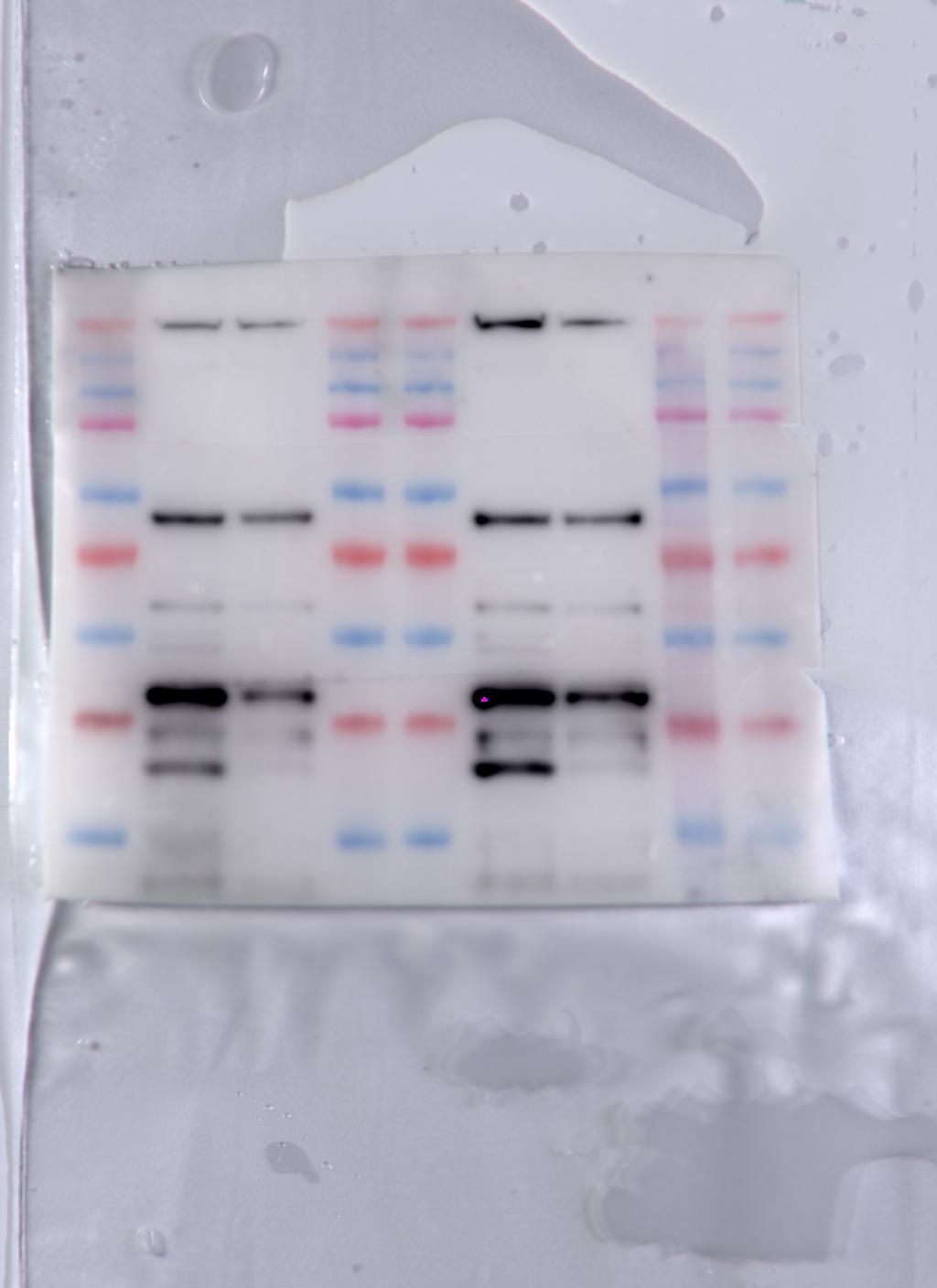

Supplement: Supplementary file 12 — EV Figure Source Data [file 44318_2025_453_MOESM12_ESM.zip › Source data EV-2/Figure EV5/EV5B/Rep2 and 3/manika gel4 g 2024.05.23_16.39.51_Ch/manika gel4 g 2024.05.23_16.39.51_Ch+Marker.jpg]

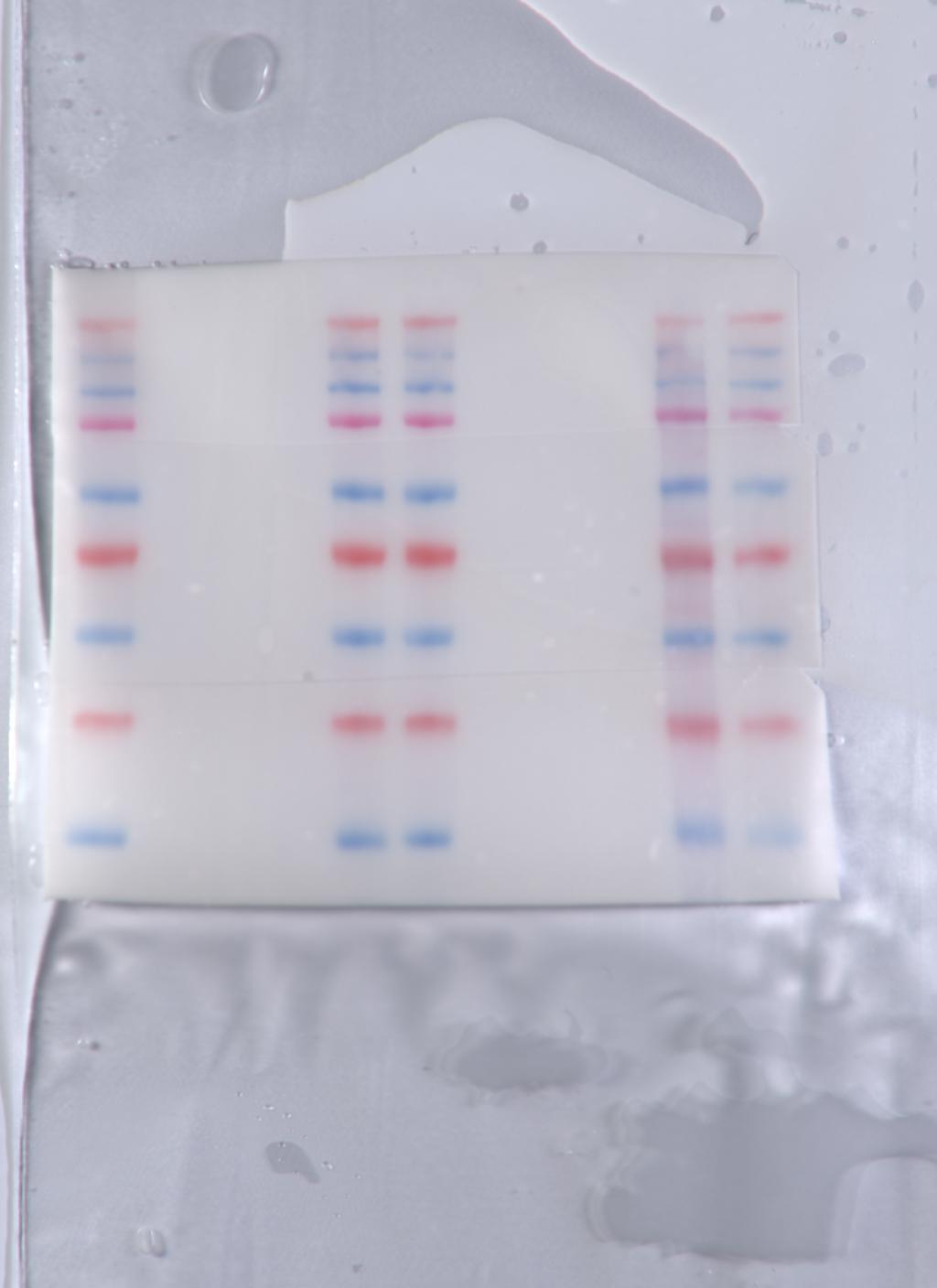

Supplement: Supplementary file 12 — EV Figure Source Data [file 44318_2025_453_MOESM12_ESM.zip › Source data EV-2/Figure EV5/EV5B/Rep2 and 3/manika gel4 g 2024.05.23_16.39.51_Ch/manika gel4 g 2024.05.23_16.39.51_Ch-Marker.jpg]

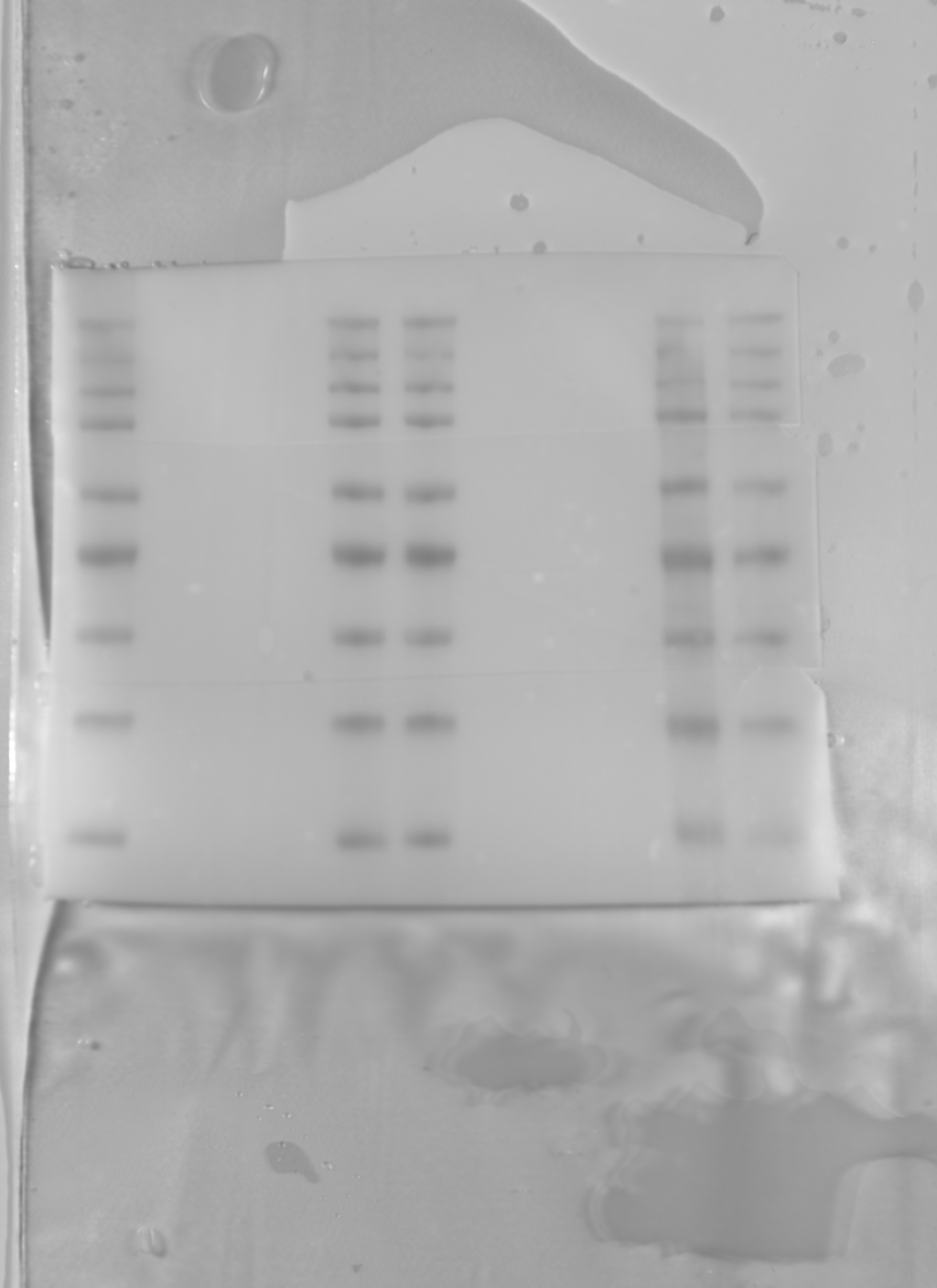

Supplement: Supplementary file 12 — EV Figure Source Data [file 44318_2025_453_MOESM12_ESM.zip › Source data EV-2/Figure EV5/EV5B/Rep2 and 3/manika gel4 g 2024.05.23_16.39.51_Ch/manika gel4 g 2024.05.23_16.39.51_Ch-Marker.tif]

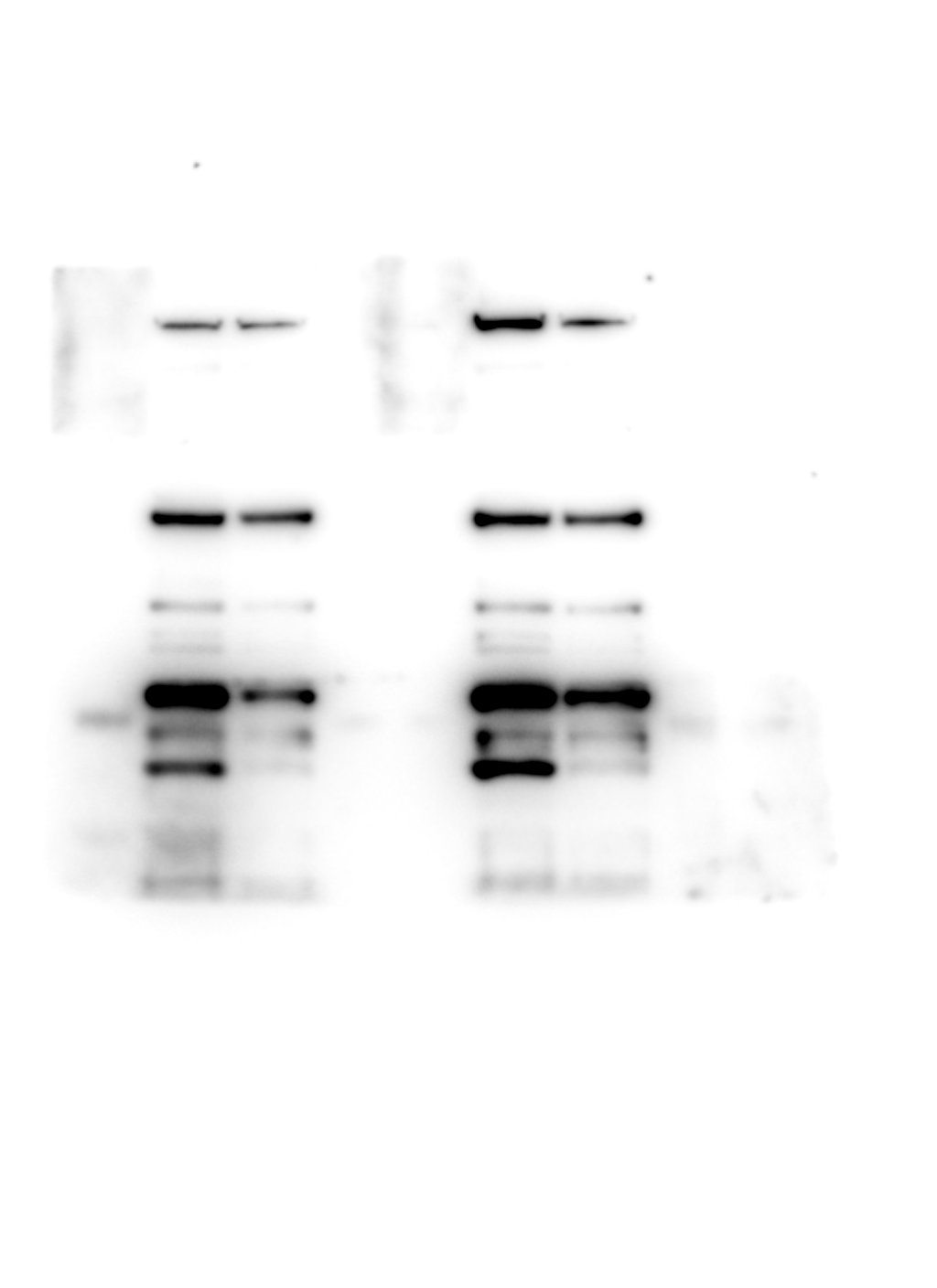

Supplement: Supplementary file 12 — EV Figure Source Data [file 44318_2025_453_MOESM12_ESM.zip › Source data EV-2/Figure EV5/EV5B/Western blot image.jpg]

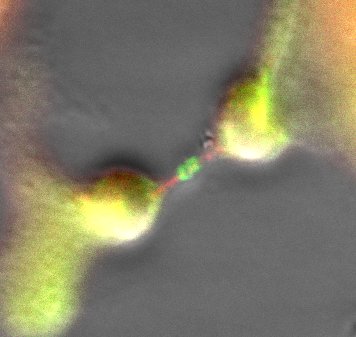

Supplement: Supplementary file 12 — EV Figure Source Data [file 44318_2025_453_MOESM12_ESM.zip › Source data EV-2/Figure EV5/EV5C/merge-dapi_1.jpg]

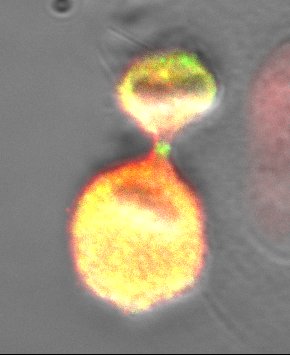

Supplement: Supplementary file 12 — EV Figure Source Data [file 44318_2025_453_MOESM12_ESM.zip › Source data EV-2/Figure EV5/EV5C/merge-dapi_2.jpg]

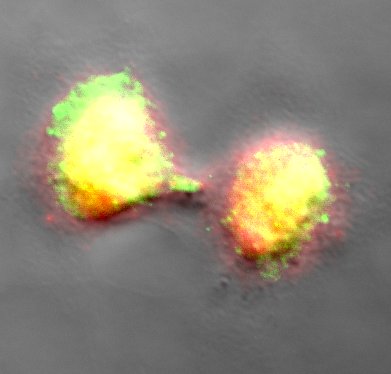

Supplement: Supplementary file 12 — EV Figure Source Data [file 44318_2025_453_MOESM12_ESM.zip › Source data EV-2/Figure EV5/EV5C/merge-dapi_3.jpg]

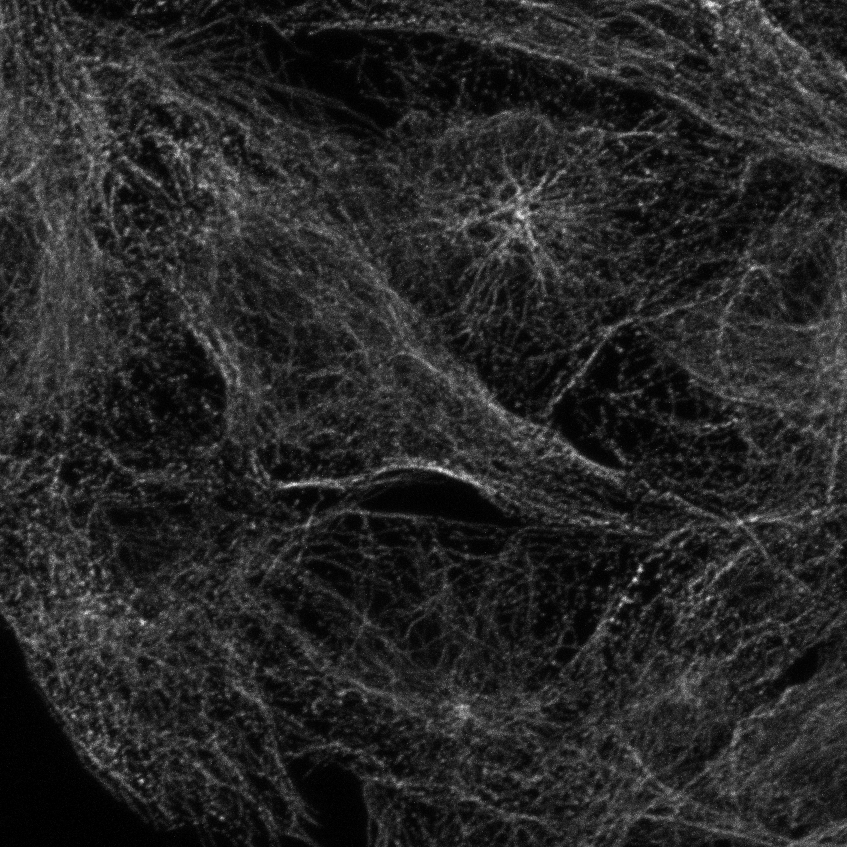

Supplement: Supplementary file 13 — Appendix Figure Source data [file 44318_2025_453_MOESM13_ESM.zip › Source data Appendix/Figure S1/S1A/Composite 29 cropped mch-tub+ total tub -1.tif]

## Slide 1
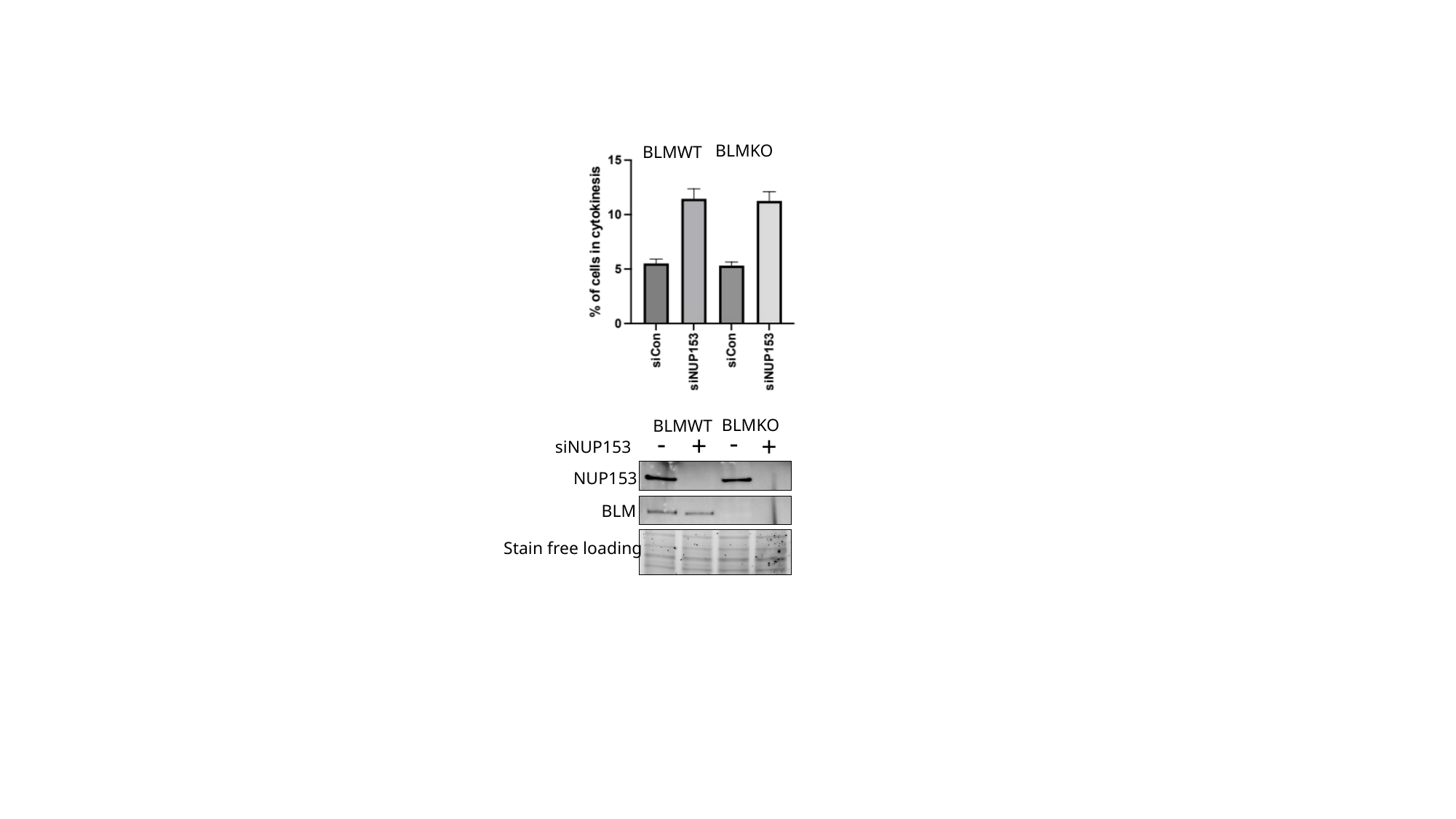

BLMKO
BLMWT
BLMKO
BLMWT
-
-
+
+
siNUP153
NUP153
BLM
Stain free loading

Supplement: Supplementary file 13 — Appendix Figure Source data [file 44318_2025_453_MOESM13_ESM.zip › Source data Appendix/Figure S1/S1B/Presentation1.pptx]

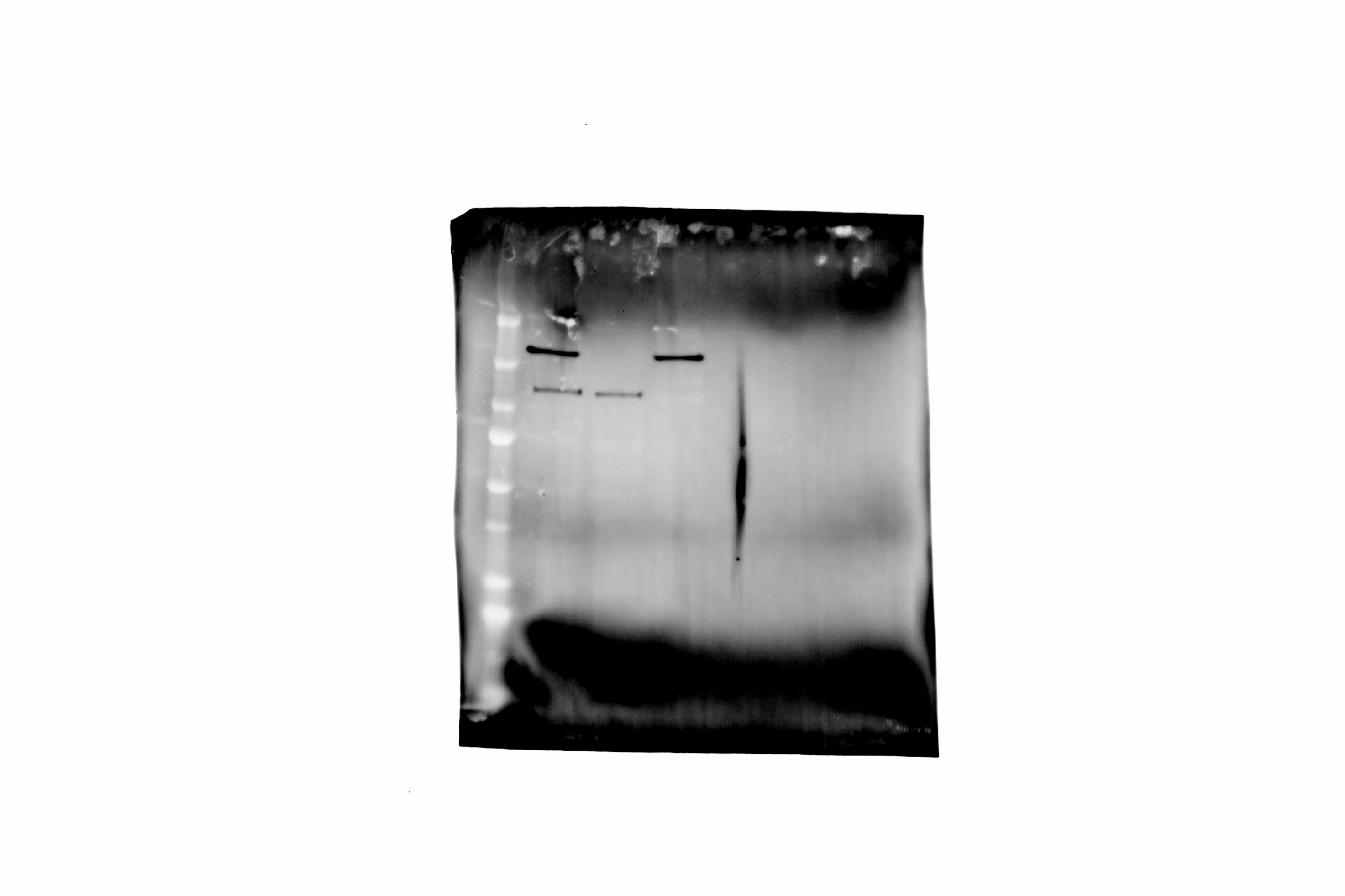

Supplement: Supplementary file 13 — Appendix Figure Source data [file 44318_2025_453_MOESM13_ESM.zip › Source data Appendix/Figure S1/S1B/WB/BIORAD 2025-04-14 09h29m23s(StarBright B700).jpg]

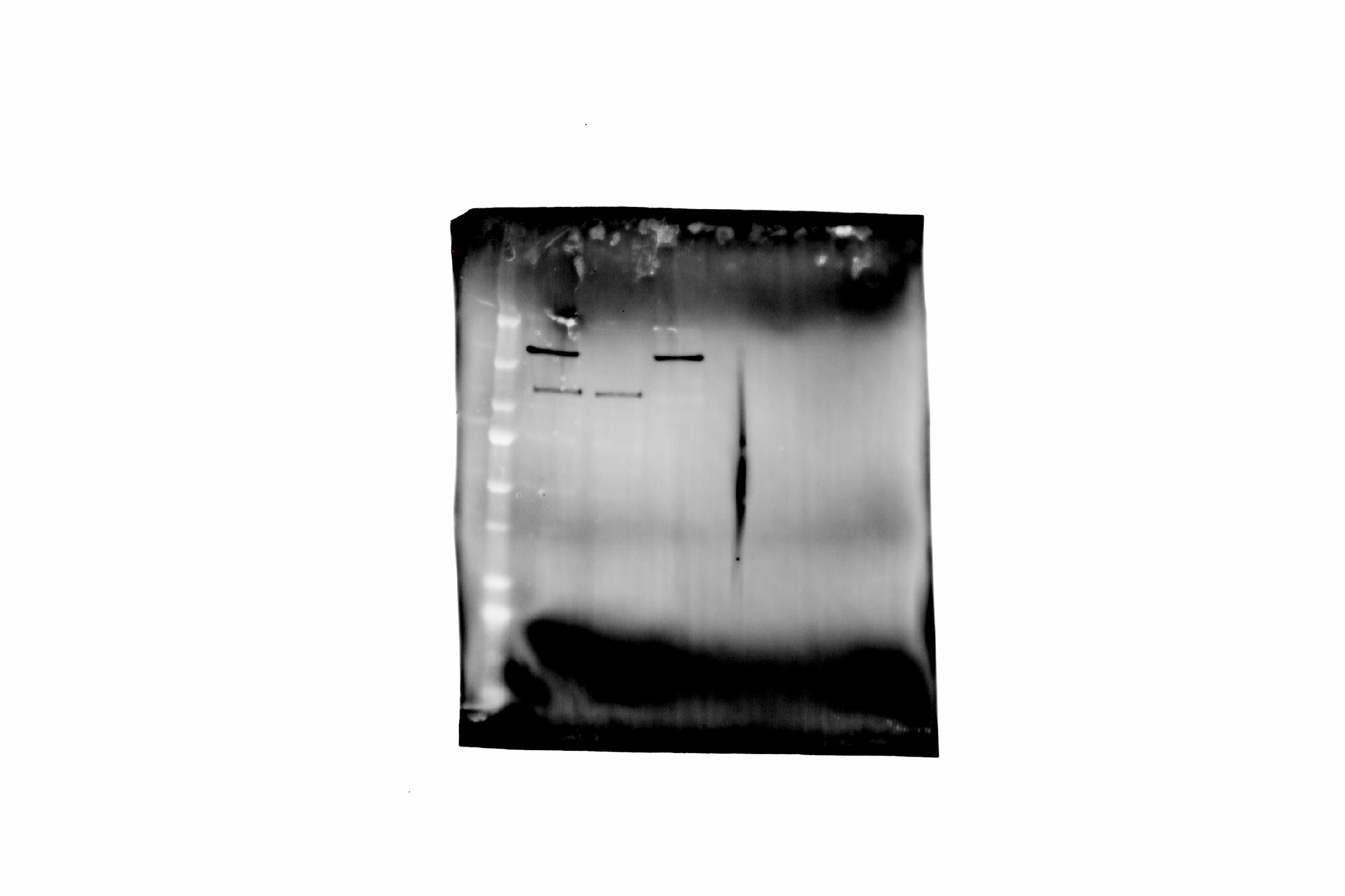

Supplement: Supplementary file 13 — Appendix Figure Source data [file 44318_2025_453_MOESM13_ESM.zip › Source data Appendix/Figure S1/S1B/WB/BIORAD 2025-04-14 09h29m23s(StarBright B700).tif]

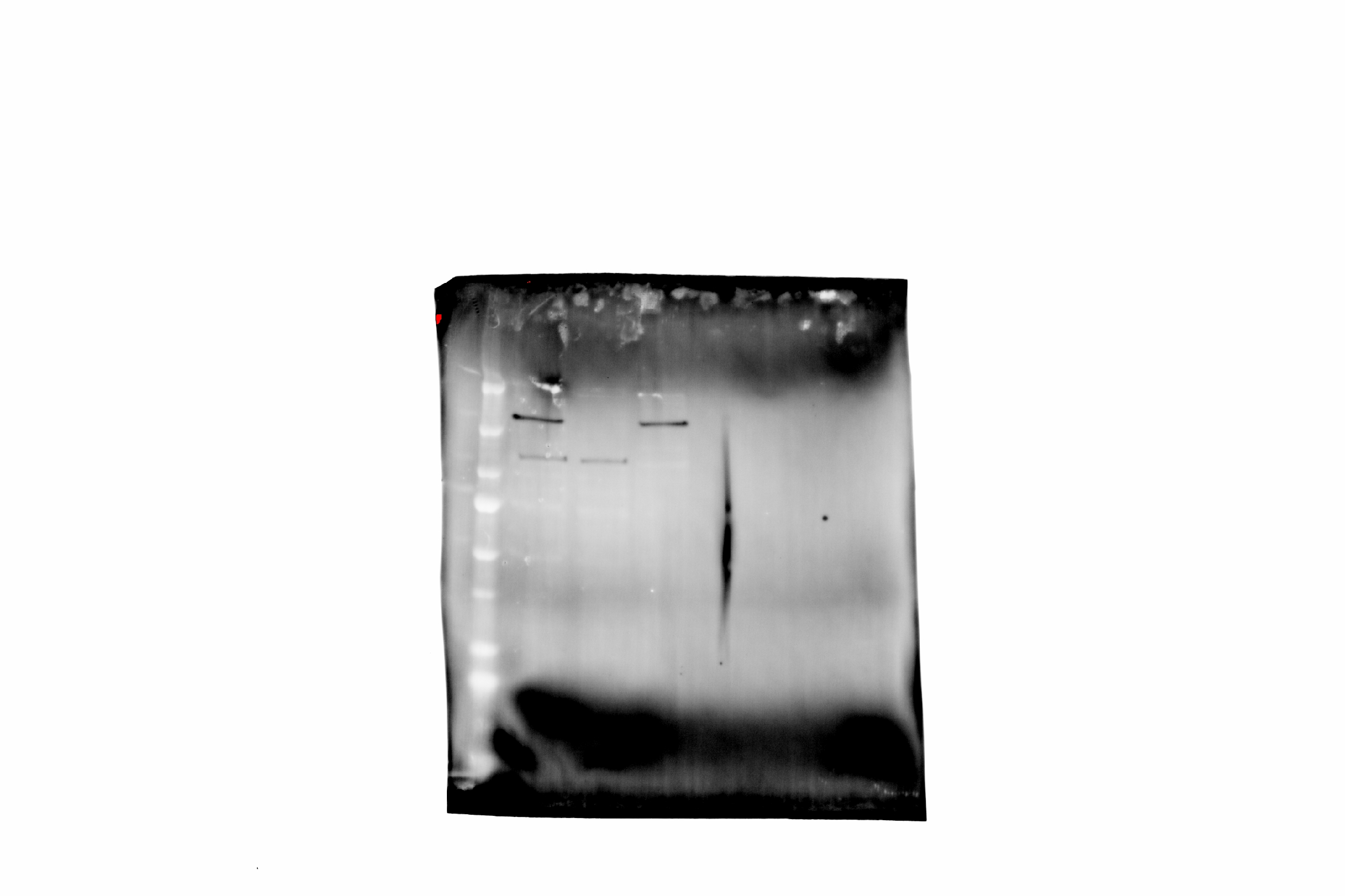

Supplement: Supplementary file 13 — Appendix Figure Source data [file 44318_2025_453_MOESM13_ESM.zip › Source data Appendix/Figure S1/S1B/WB/BIORAD 2025-04-15 06h39m15s(StarBright B700).jpg]

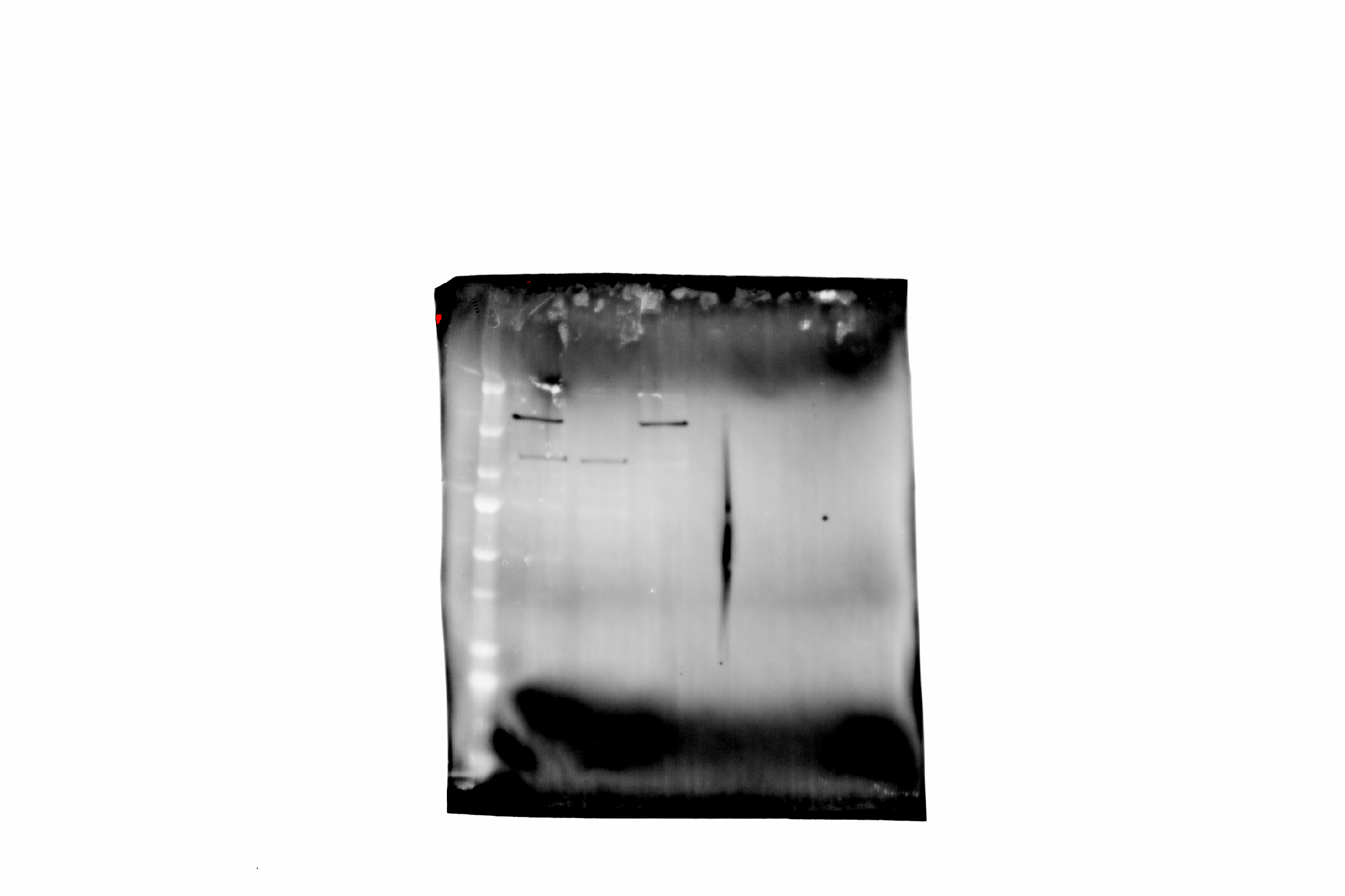

Supplement: Supplementary file 13 — Appendix Figure Source data [file 44318_2025_453_MOESM13_ESM.zip › Source data Appendix/Figure S1/S1B/WB/BIORAD 2025-04-15 06h39m15s(StarBright B700).tif]

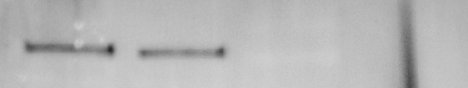

Supplement: Supplementary file 13 — Appendix Figure Source data [file 44318_2025_453_MOESM13_ESM.zip › Source data Appendix/Figure S1/S1B/WB/BLM BIORAD 2025-04-14 09h29m23s(StarBright B700)-1.jpBLMg.jpg]

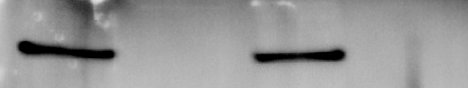

Supplement: Supplementary file 13 — Appendix Figure Source data [file 44318_2025_453_MOESM13_ESM.zip › Source data Appendix/Figure S1/S1B/WB/NUP153 BIORAD 2025-04-14 09h29m23s(StarBright B700)-1.jpg]

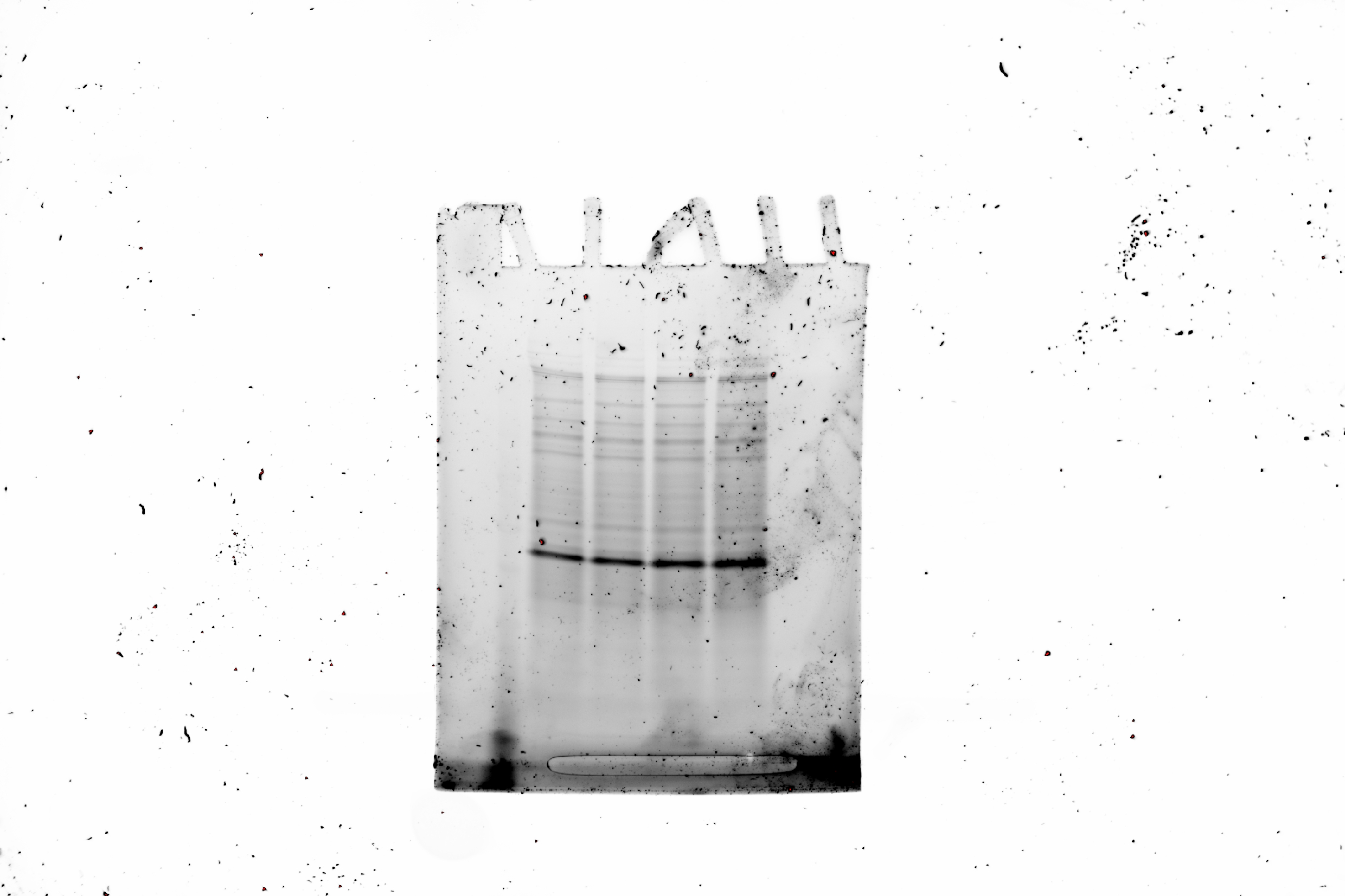

Supplement: Supplementary file 13 — Appendix Figure Source data [file 44318_2025_453_MOESM13_ESM.zip › Source data Appendix/Figure S1/S1B/WB/RB EMBO 2025-04-13 13h26m52s(Stain Free Gel).jpg]

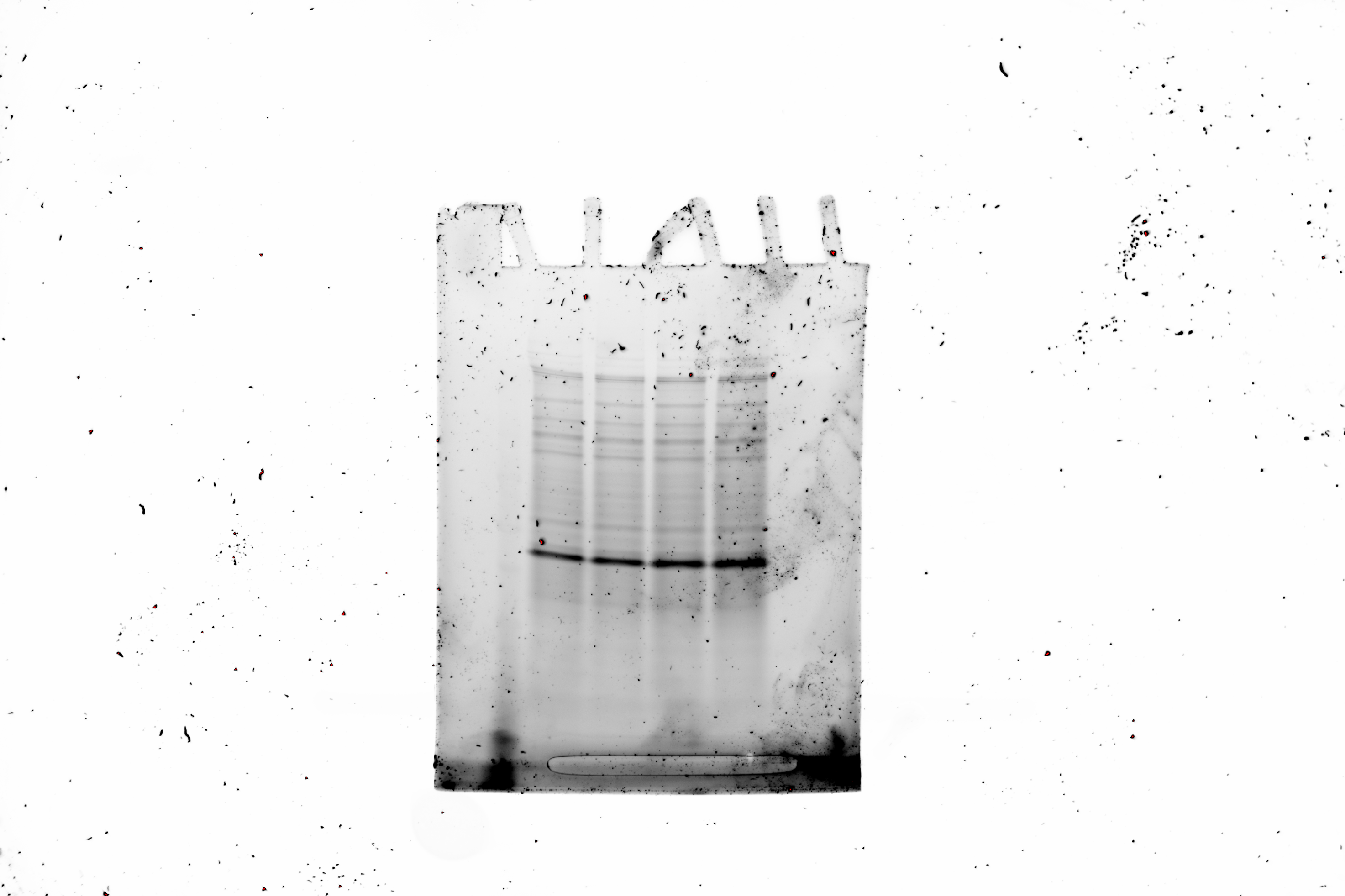

Supplement: Supplementary file 13 — Appendix Figure Source data [file 44318_2025_453_MOESM13_ESM.zip › Source data Appendix/Figure S1/S1B/WB/RB EMBO 2025-04-13 13h26m52s(Stain Free Gel).tif]

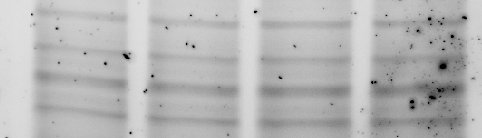

Supplement: Supplementary file 13 — Appendix Figure Source data [file 44318_2025_453_MOESM13_ESM.zip › Source data Appendix/Figure S1/S1B/WB/Stain free EMBO 2025-04-13 13h26m52s(Stain Free Gel)-1.jpg]

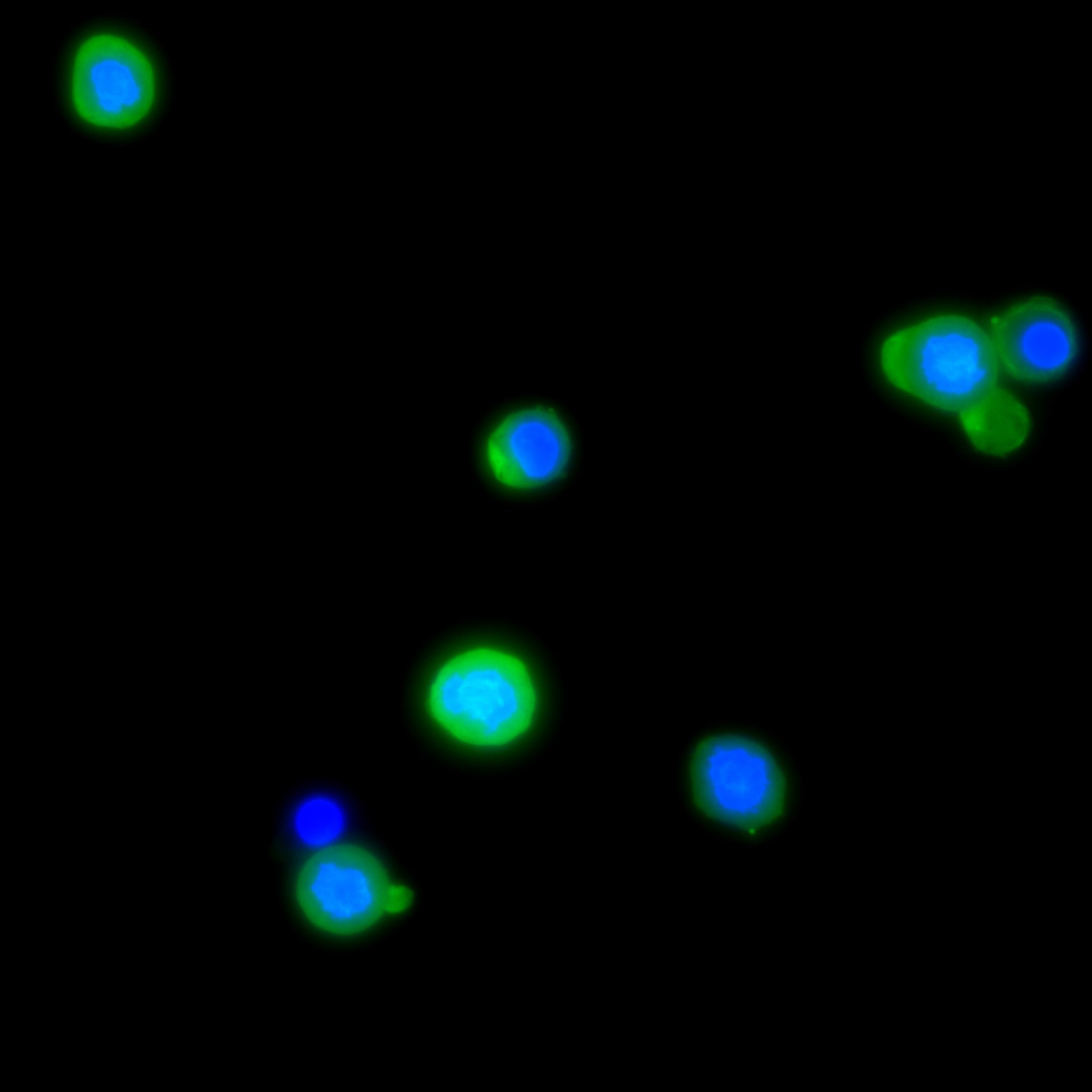

Supplement: Supplementary file 13 — Appendix Figure Source data [file 44318_2025_453_MOESM13_ESM.zip › Source data Appendix/Figure S3/S3A/BLM KO 0.jpg]

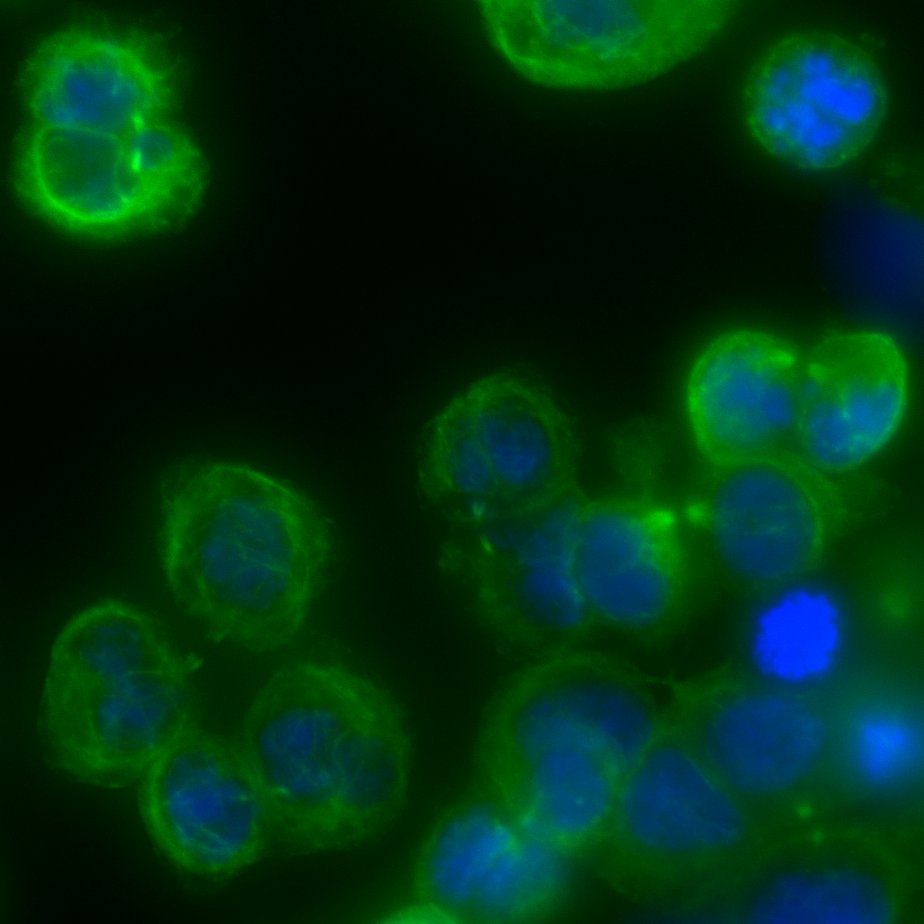

Supplement: Supplementary file 13 — Appendix Figure Source data [file 44318_2025_453_MOESM13_ESM.zip › Source data Appendix/Figure S3/S3A/BLM KO 2.jpg]

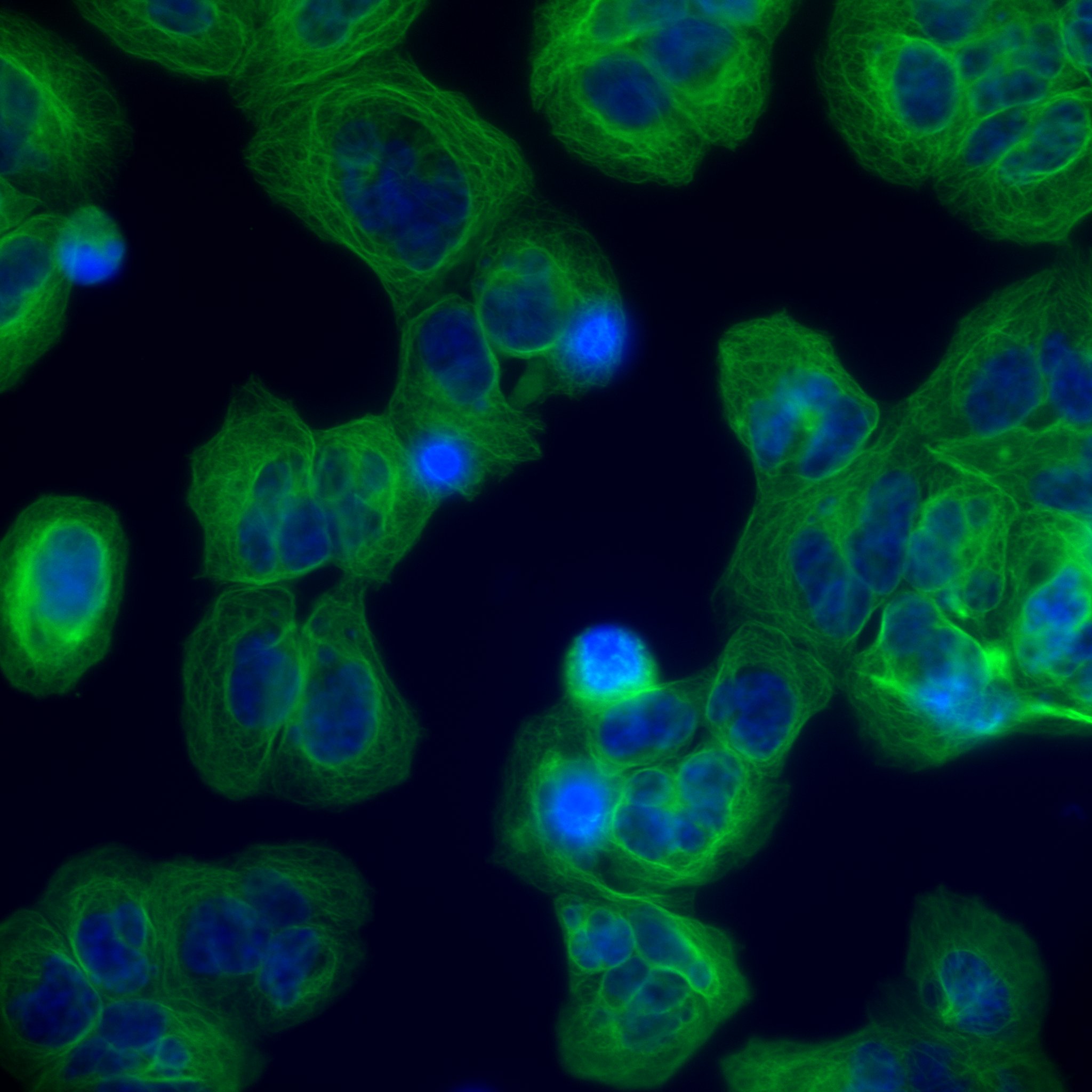

Supplement: Supplementary file 13 — Appendix Figure Source data [file 44318_2025_453_MOESM13_ESM.zip › Source data Appendix/Figure S3/S3A/BLM KO 4.jpg]

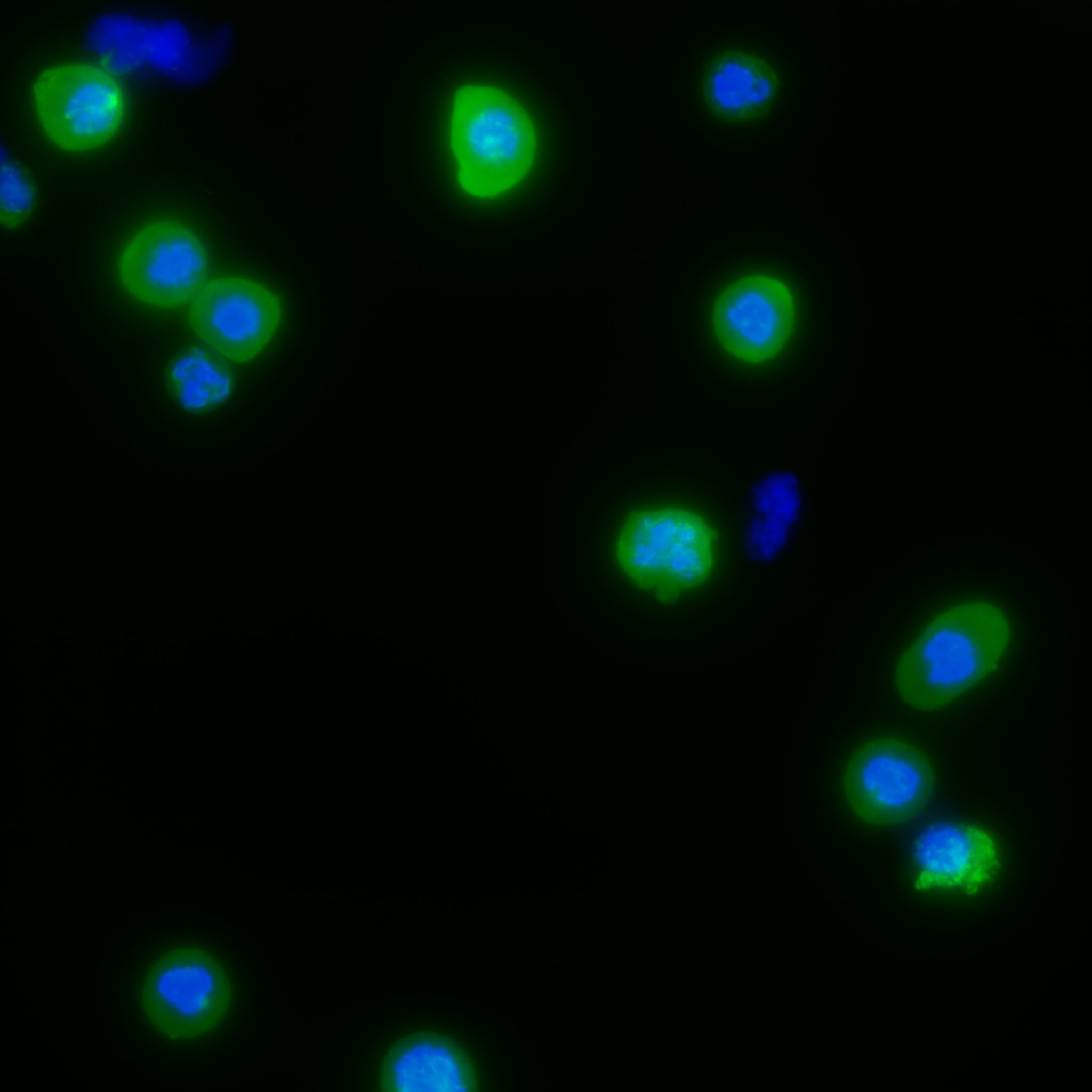

Supplement: Supplementary file 13 — Appendix Figure Source data [file 44318_2025_453_MOESM13_ESM.zip › Source data Appendix/Figure S3/S3A/BLM WT 0.jpg]

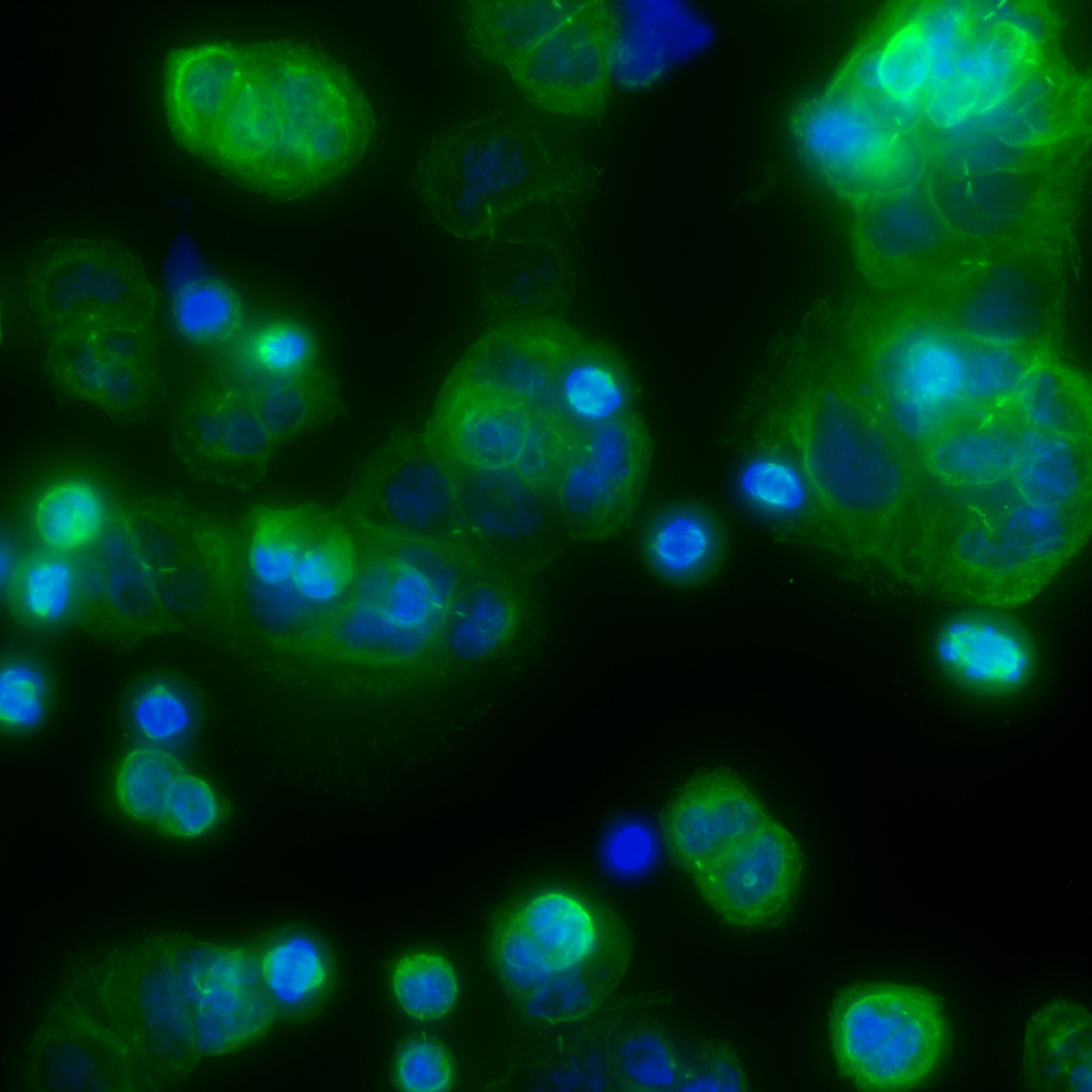

Supplement: Supplementary file 13 — Appendix Figure Source data [file 44318_2025_453_MOESM13_ESM.zip › Source data Appendix/Figure S3/S3A/BLM WT 2.jpg]

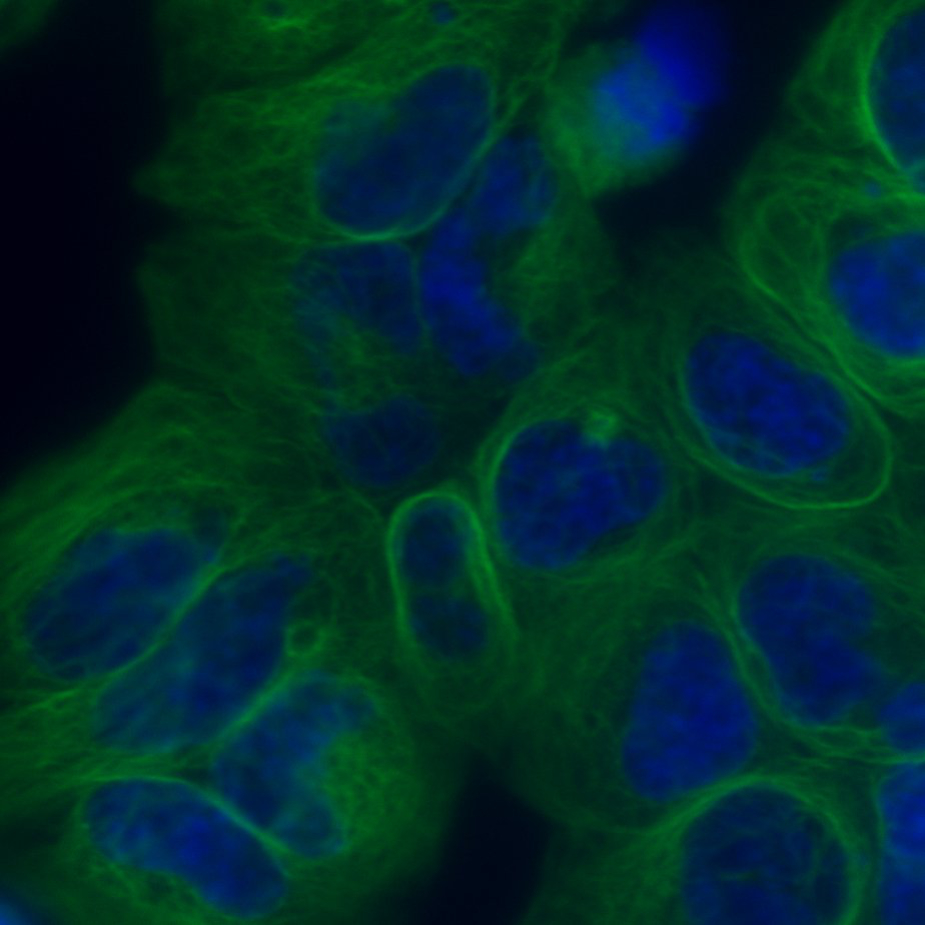

Supplement: Supplementary file 13 — Appendix Figure Source data [file 44318_2025_453_MOESM13_ESM.zip › Source data Appendix/Figure S3/S3A/BLM WT 4.jpg]

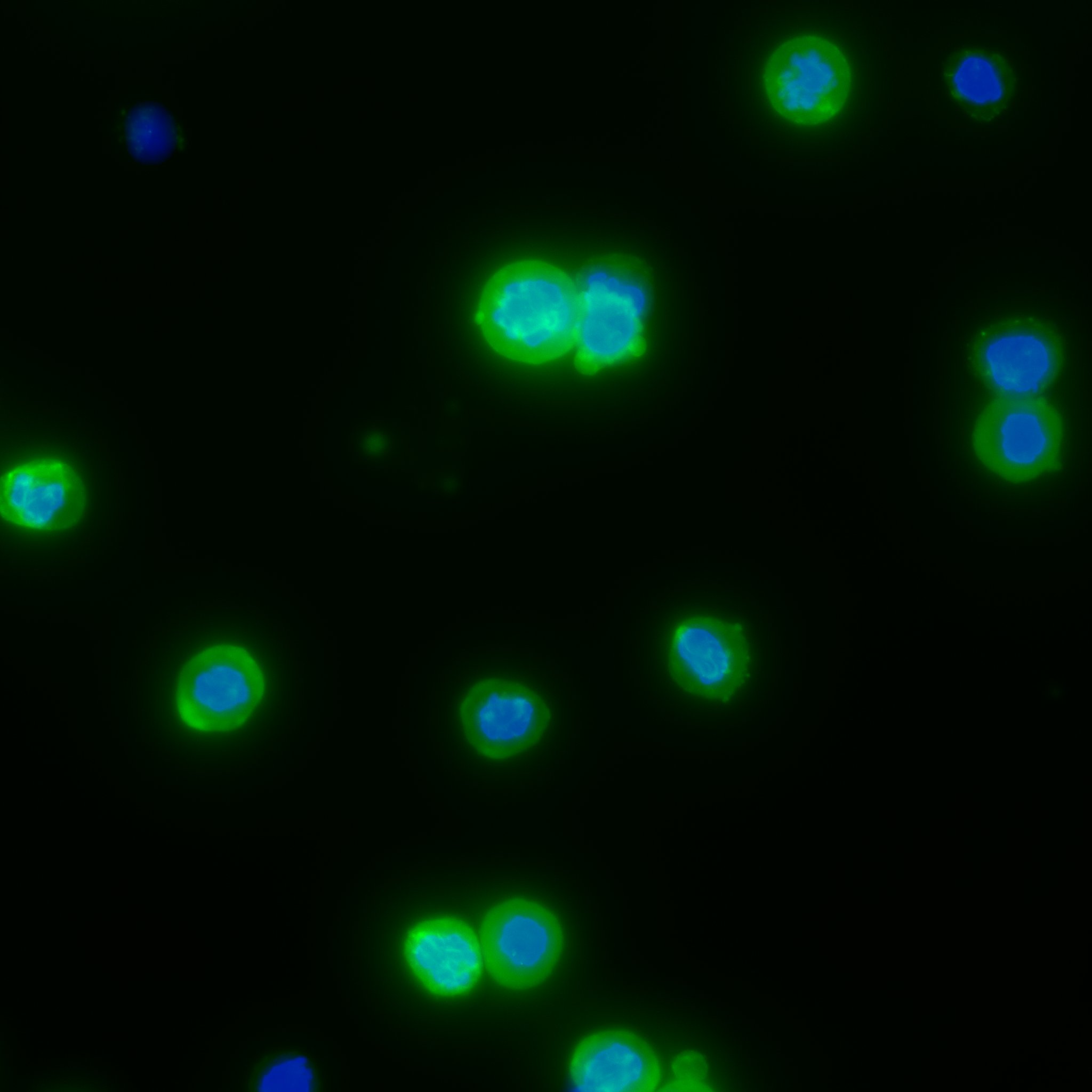

Supplement: Supplementary file 13 — Appendix Figure Source data [file 44318_2025_453_MOESM13_ESM.zip › Source data Appendix/Figure S3/S3B/U2OS DMSO 0.jpg]

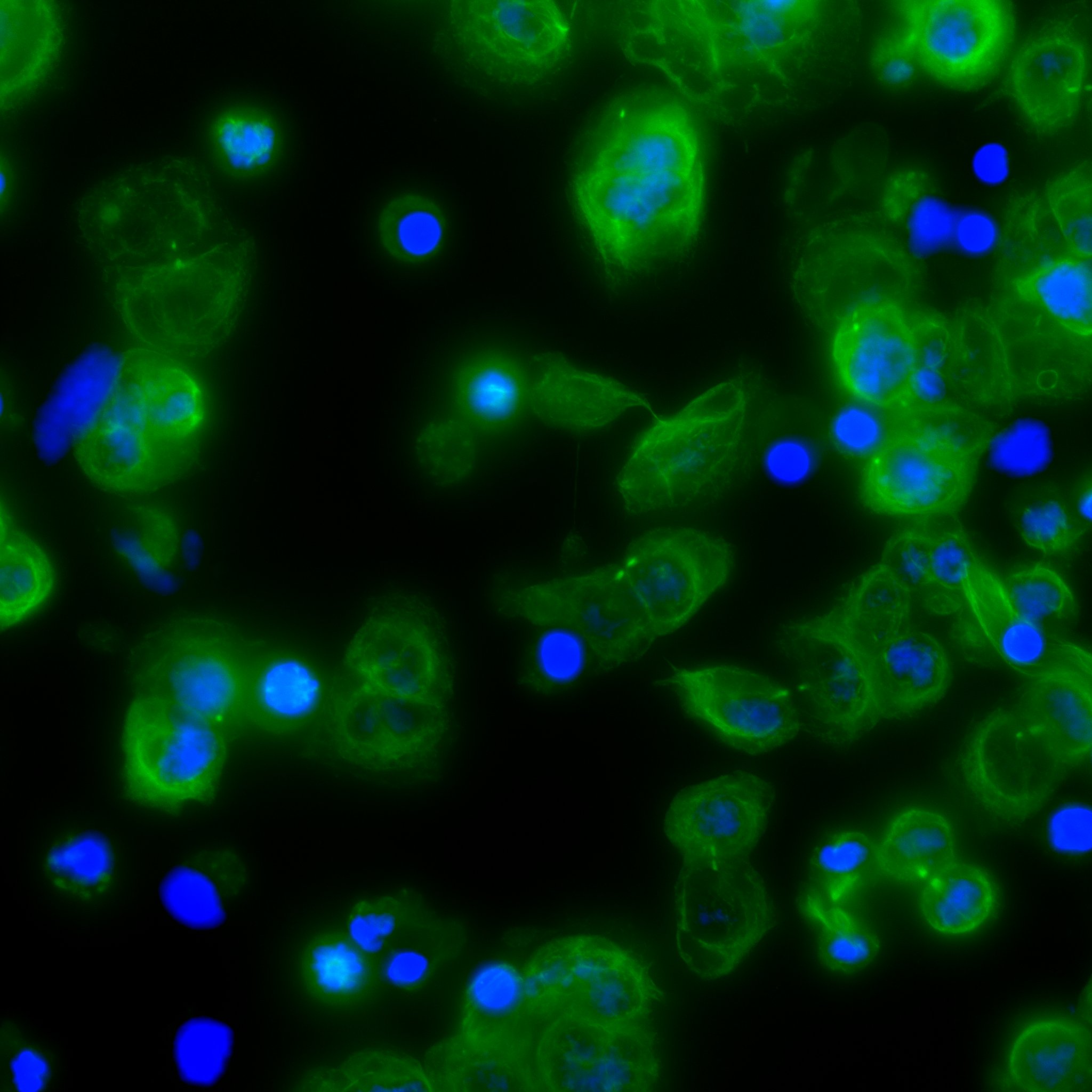

Supplement: Supplementary file 13 — Appendix Figure Source data [file 44318_2025_453_MOESM13_ESM.zip › Source data Appendix/Figure S3/S3B/U2OS DMSO 2.jpg]

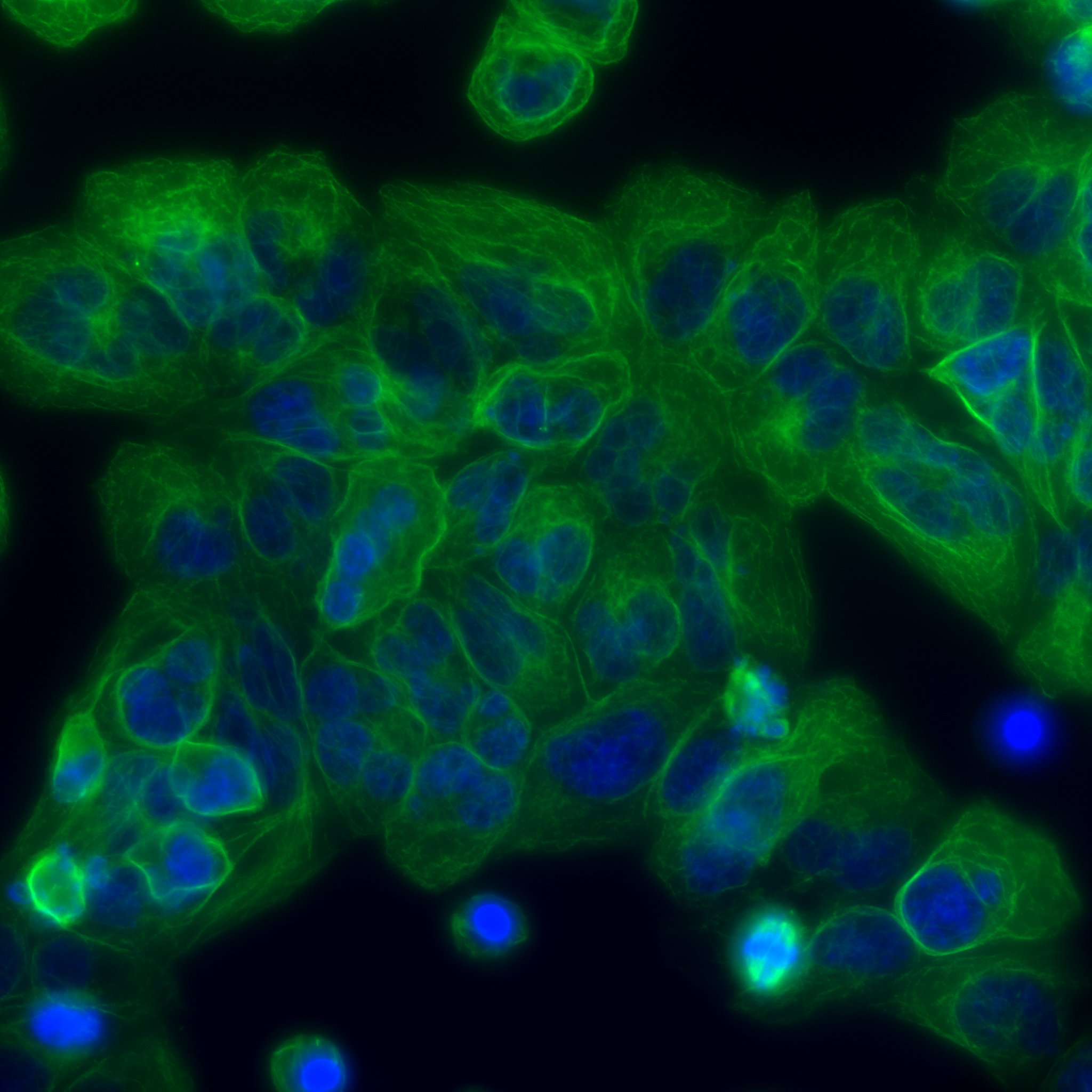

Supplement: Supplementary file 13 — Appendix Figure Source data [file 44318_2025_453_MOESM13_ESM.zip › Source data Appendix/Figure S3/S3B/U2OS DMSO 4.jpg]

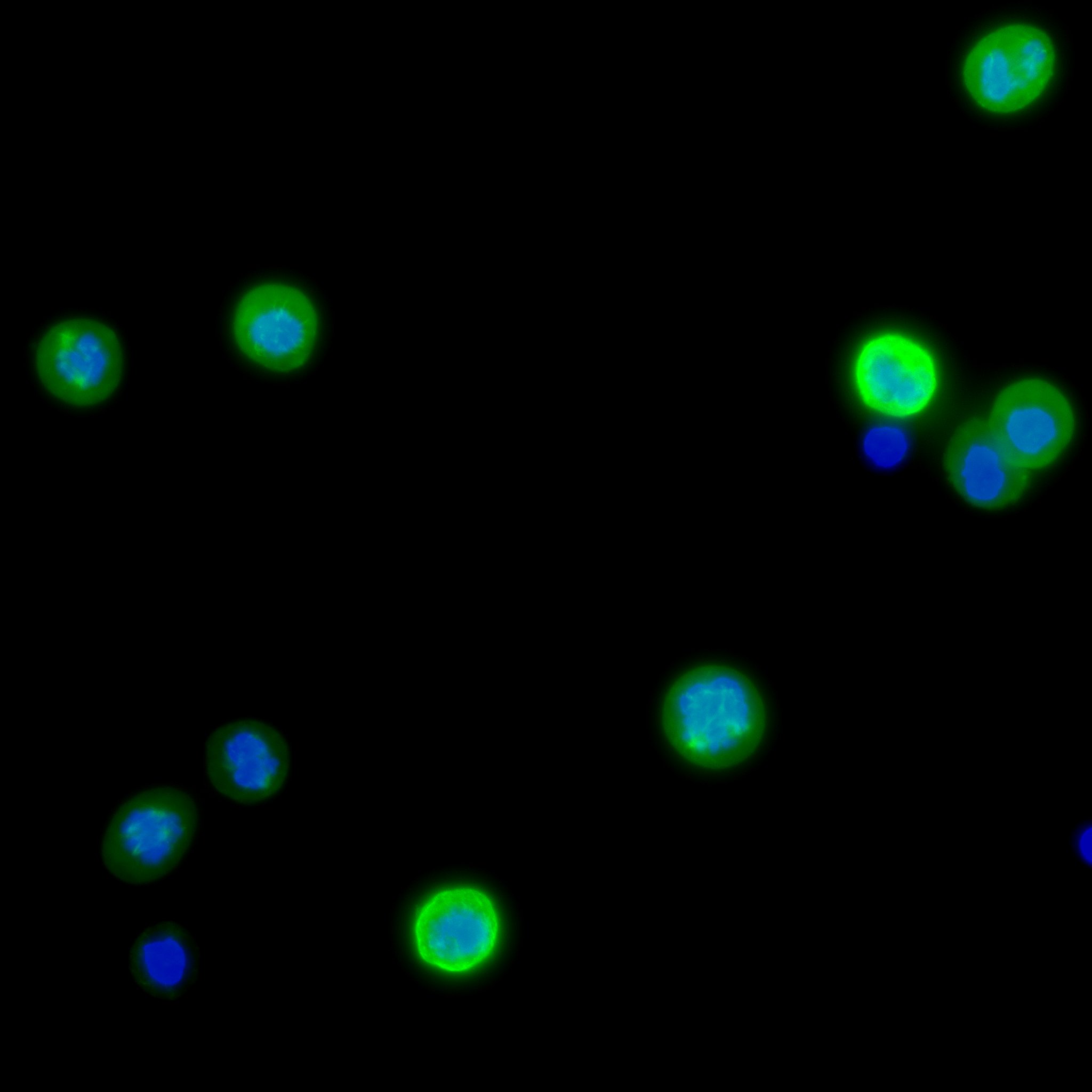

Supplement: Supplementary file 13 — Appendix Figure Source data [file 44318_2025_453_MOESM13_ESM.zip › Source data Appendix/Figure S3/S3B/U2OS HAMNO 0.jpg]

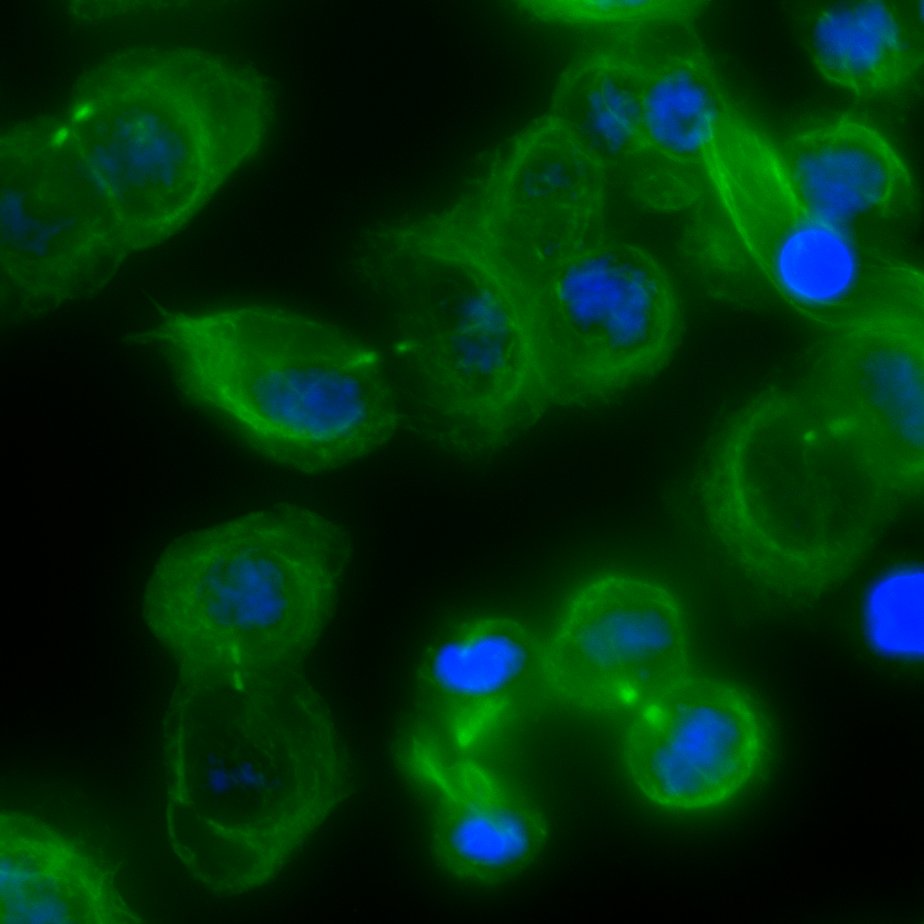

Supplement: Supplementary file 13 — Appendix Figure Source data [file 44318_2025_453_MOESM13_ESM.zip › Source data Appendix/Figure S3/S3B/U2OS HAMNO 2.jpg]

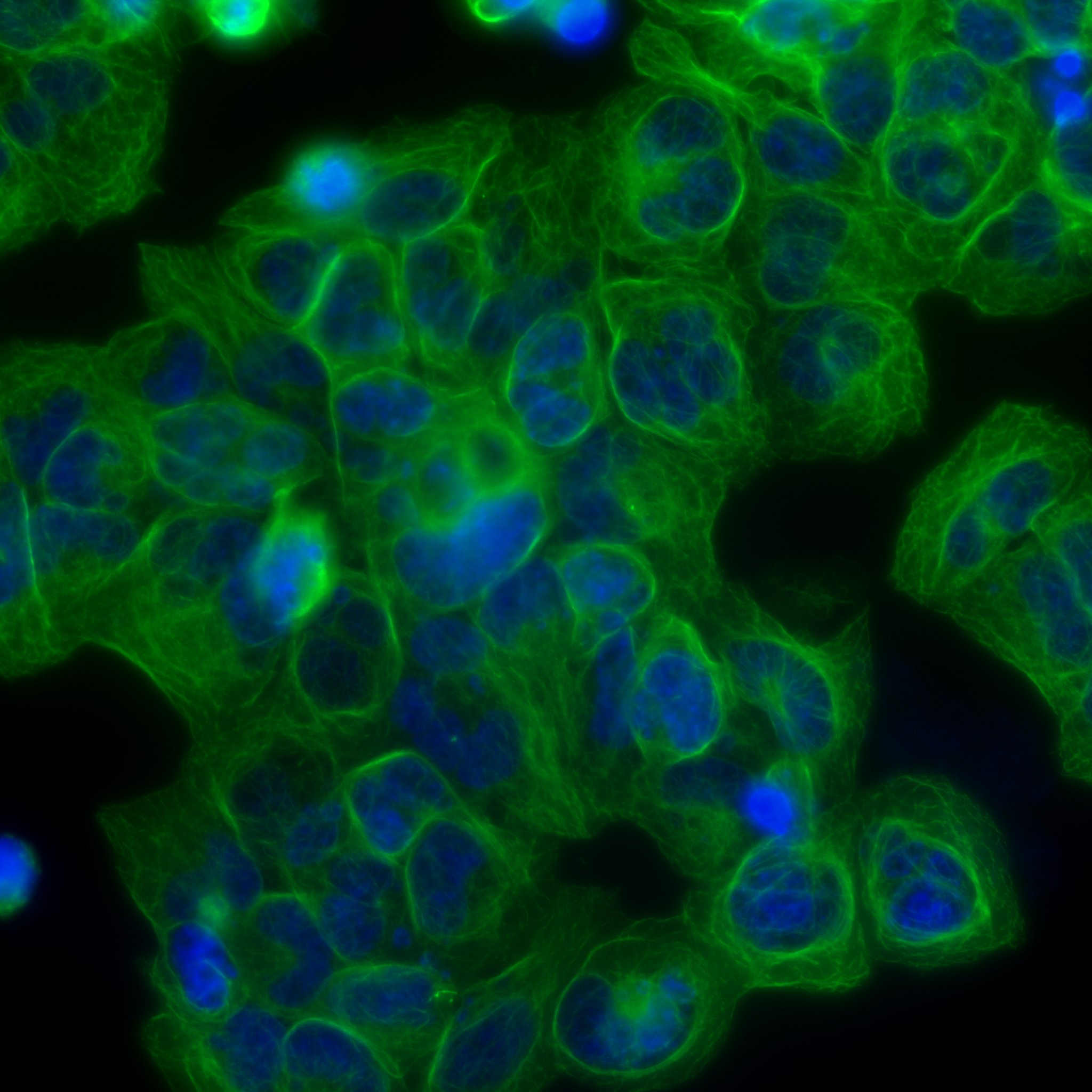

Supplement: Supplementary file 13 — Appendix Figure Source data [file 44318_2025_453_MOESM13_ESM.zip › Source data Appendix/Figure S3/S3B/U2OS HAMNO 4.jpg]

## Slide 1
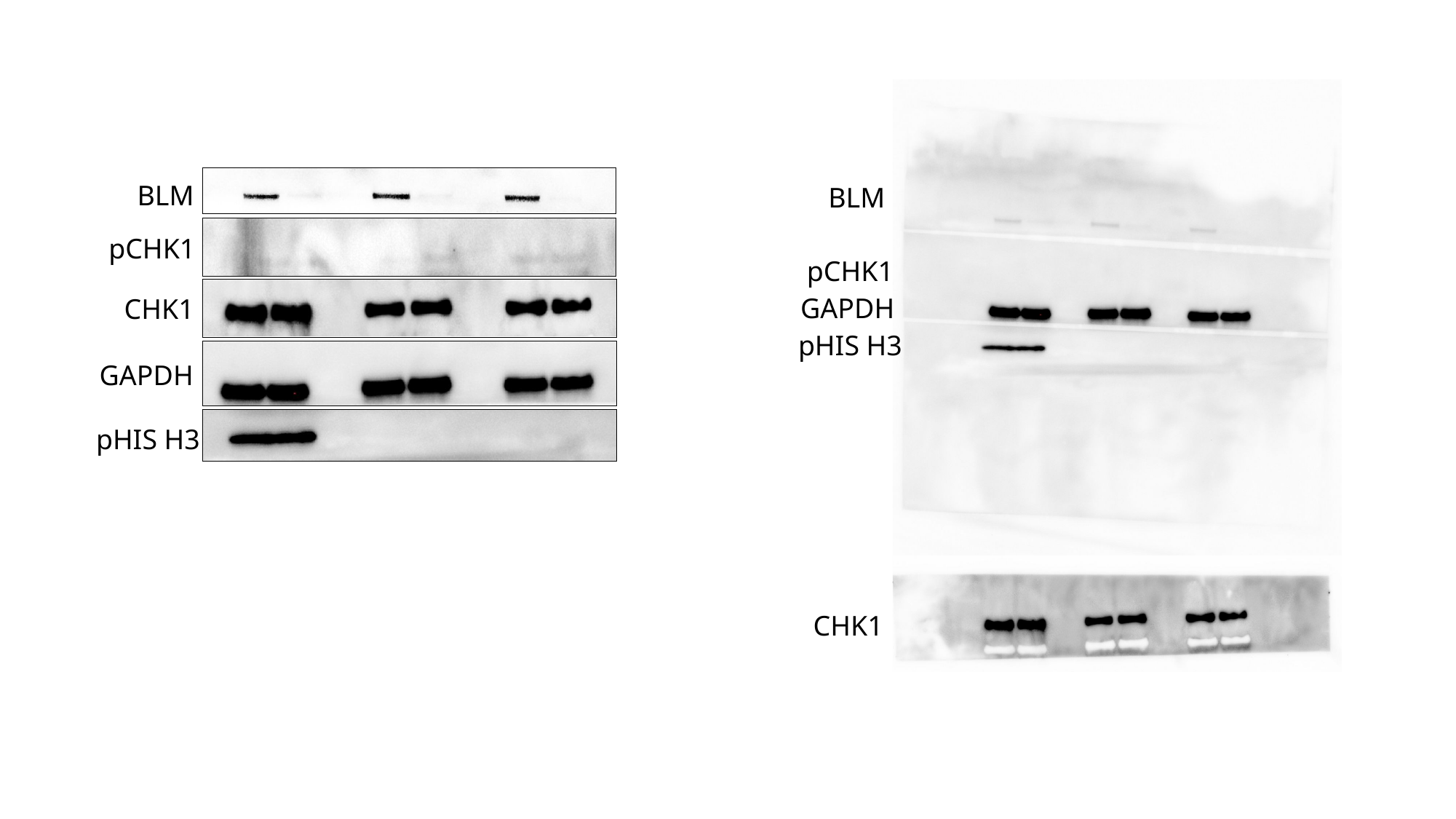

BLM
BLM
pCHK1
pCHK1
GAPDH
CHK1
pHIS H3
GAPDH
pHIS H3
CHK1

Supplement: Supplementary file 13 — Appendix Figure Source data [file 44318_2025_453_MOESM13_ESM.zip › Source data Appendix/Figure S3/S3C/-Aph pCHK1.pptx]

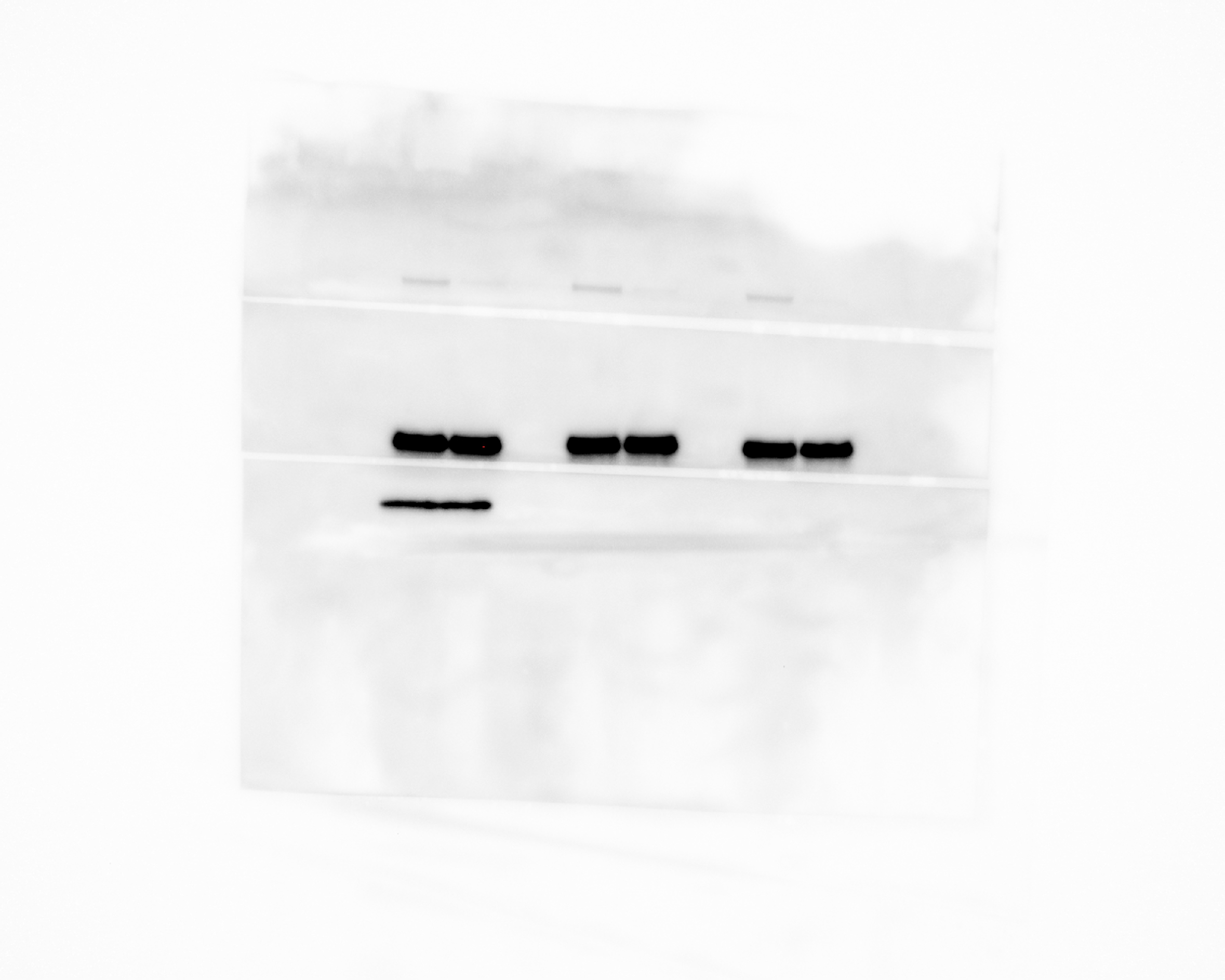

Supplement: Supplementary file 13 — Appendix Figure Source data [file 44318_2025_453_MOESM13_ESM.zip › Source data Appendix/Figure S3/S3C/ChemiDoc Images 2024-11-23_17.51.06/Rahul 2024-11-19 13h45m27s(Chemiluminescence).jpg]

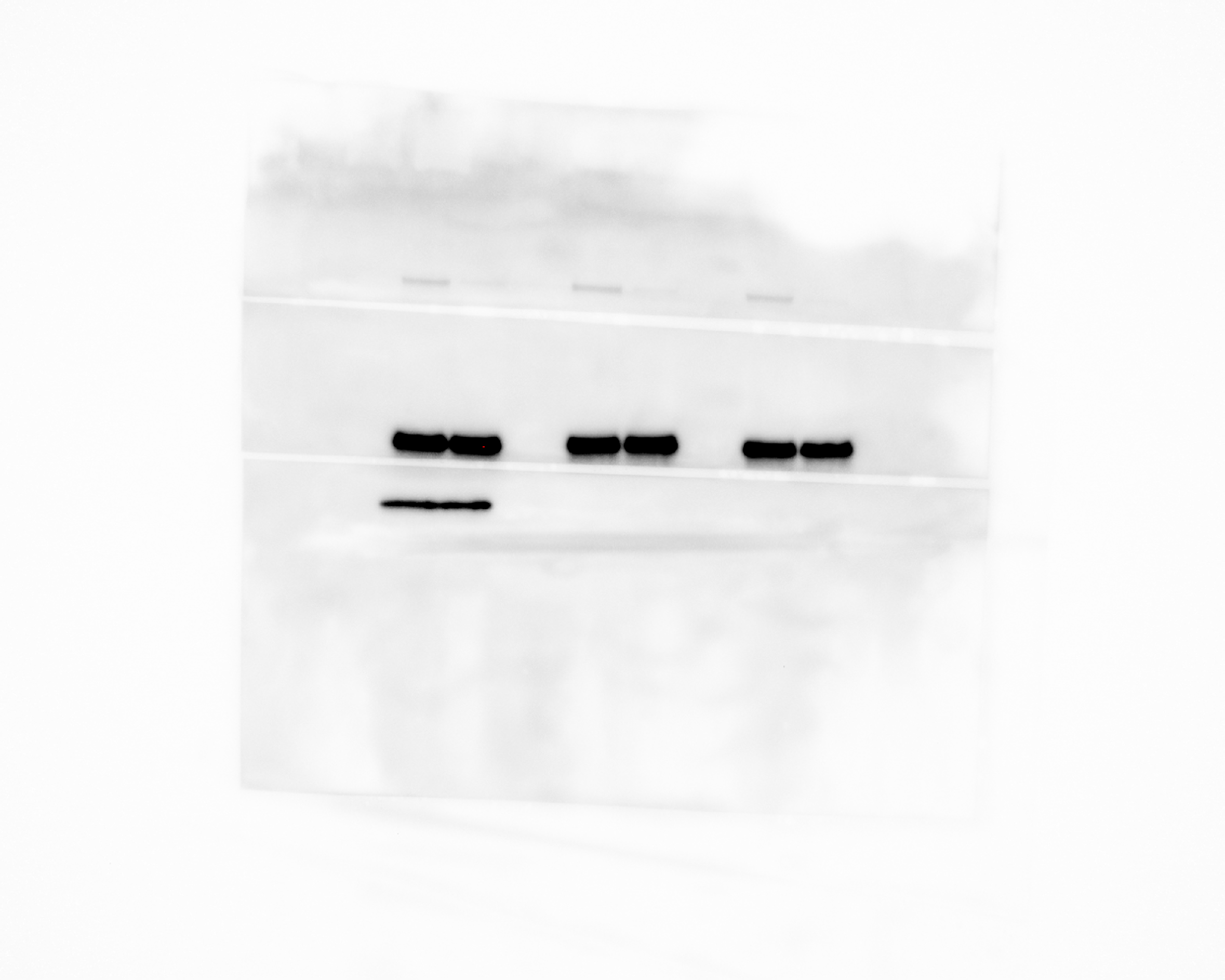

Supplement: Supplementary file 13 — Appendix Figure Source data [file 44318_2025_453_MOESM13_ESM.zip › Source data Appendix/Figure S3/S3C/ChemiDoc Images 2024-11-23_17.51.06/Rahul 2024-11-19 13h45m27s(Chemiluminescence).tif]

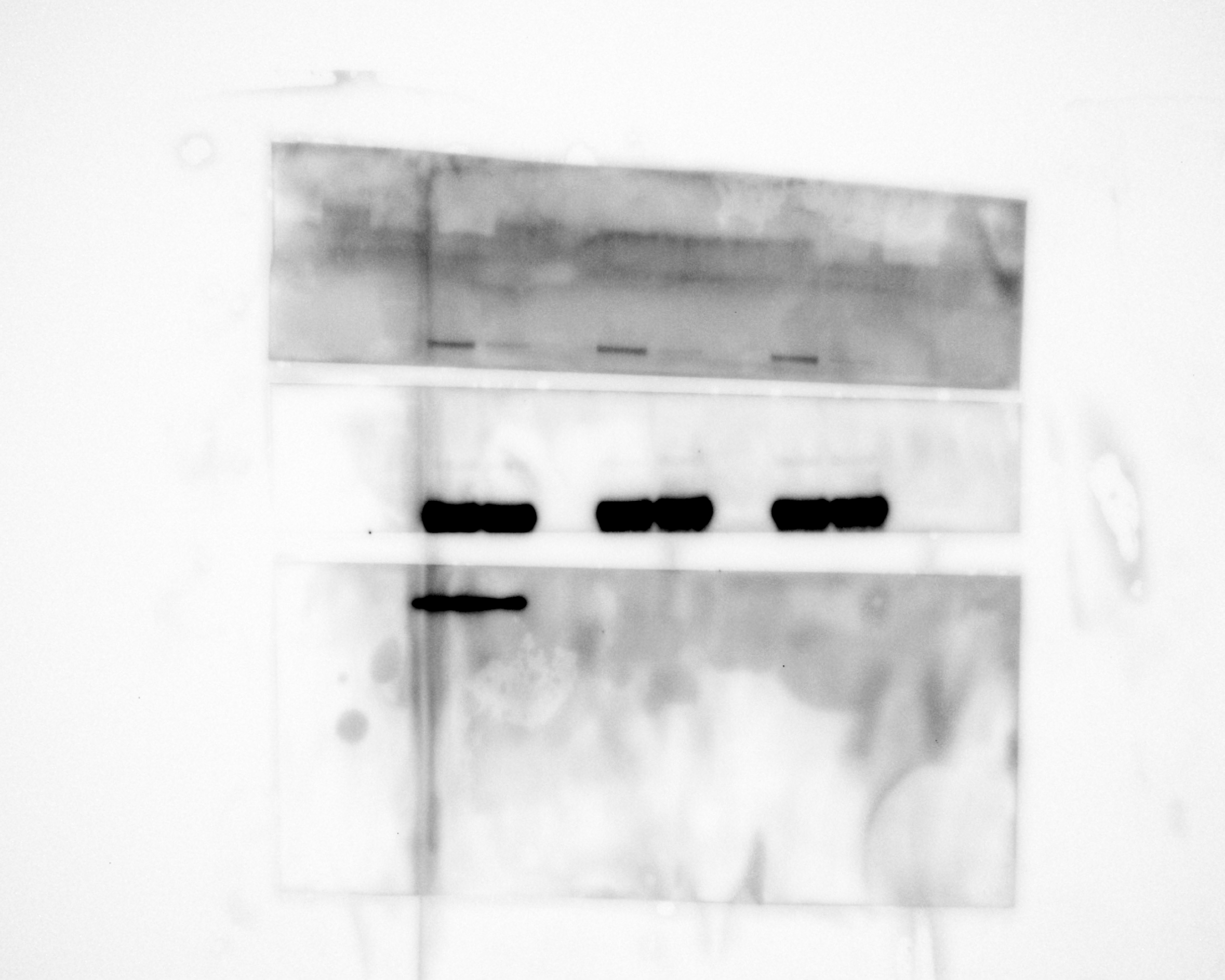

Supplement: Supplementary file 13 — Appendix Figure Source data [file 44318_2025_453_MOESM13_ESM.zip › Source data Appendix/Figure S3/S3C/ChemiDoc Images 2024-11-23_17.51.49/Rahul 2024-11-20 16h41m15s(Chemiluminescence).jpg]

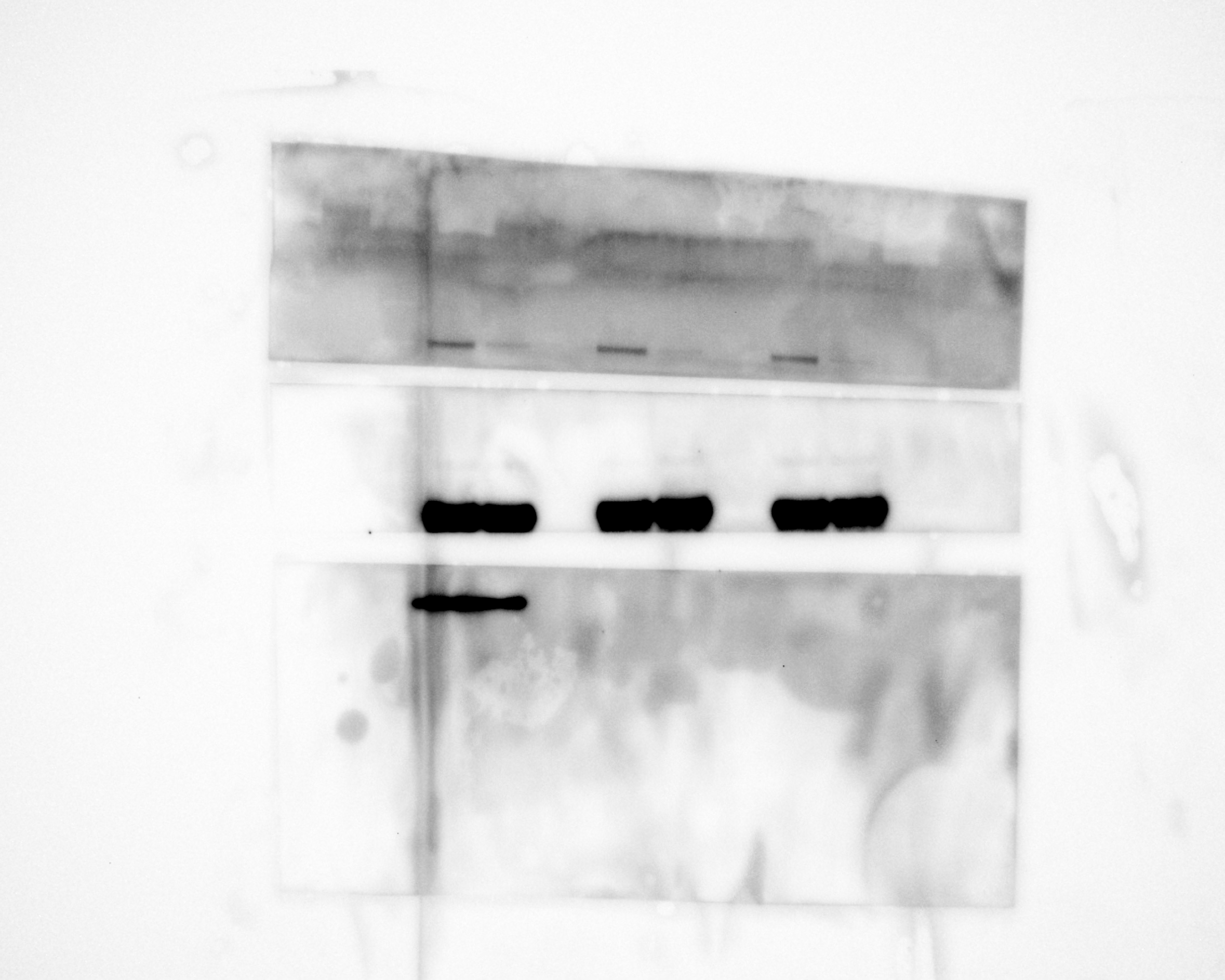

Supplement: Supplementary file 13 — Appendix Figure Source data [file 44318_2025_453_MOESM13_ESM.zip › Source data Appendix/Figure S3/S3C/ChemiDoc Images 2024-11-23_17.51.49/Rahul 2024-11-20 16h41m15s(Chemiluminescence).tif]

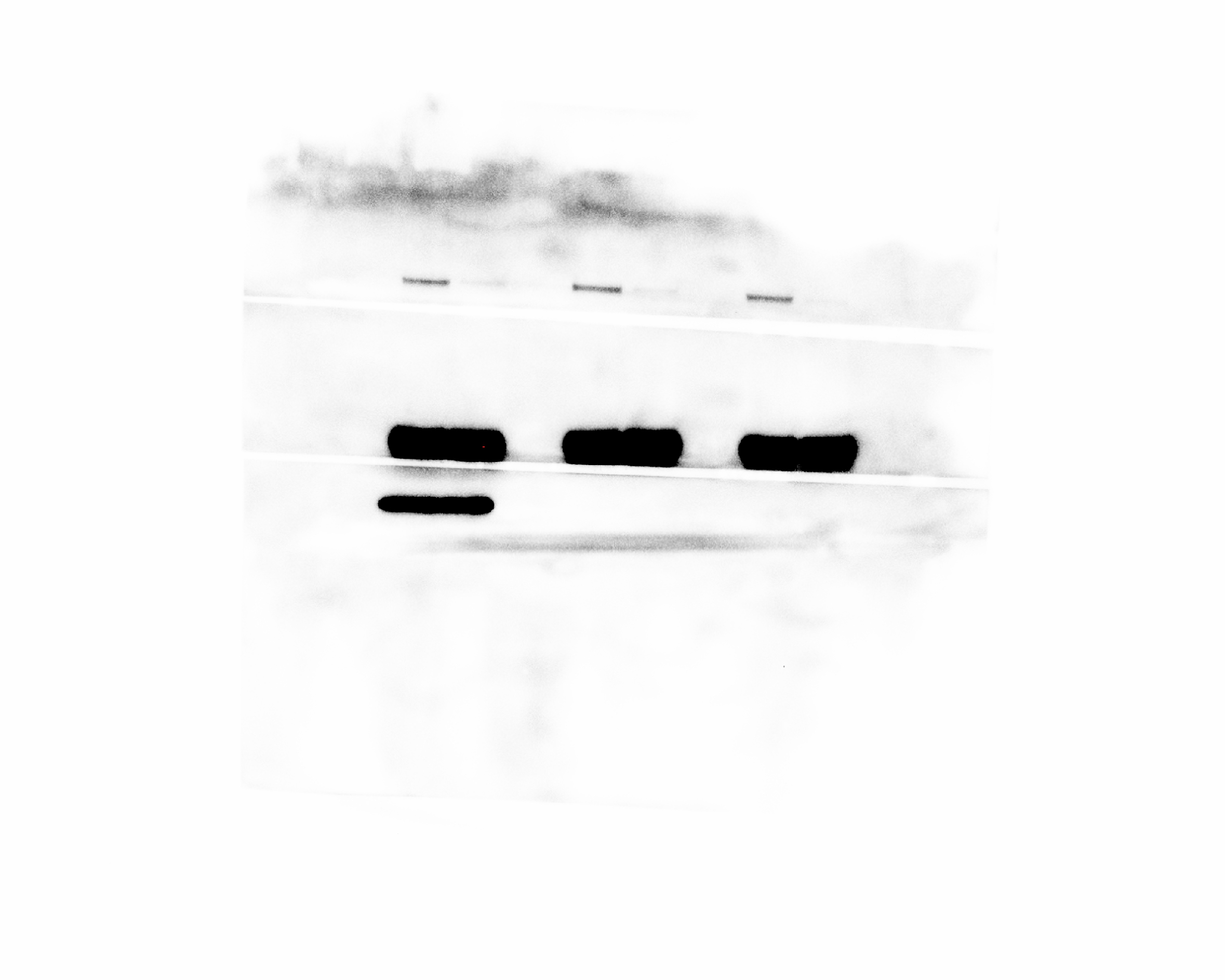

Supplement: Supplementary file 13 — Appendix Figure Source data [file 44318_2025_453_MOESM13_ESM.zip › Source data Appendix/Figure S3/S3C/ChemiDoc Images 2024-11-23_17.52.16/Rahul 2024-11-19 13h45m27s(Chemiluminescence).jpg]

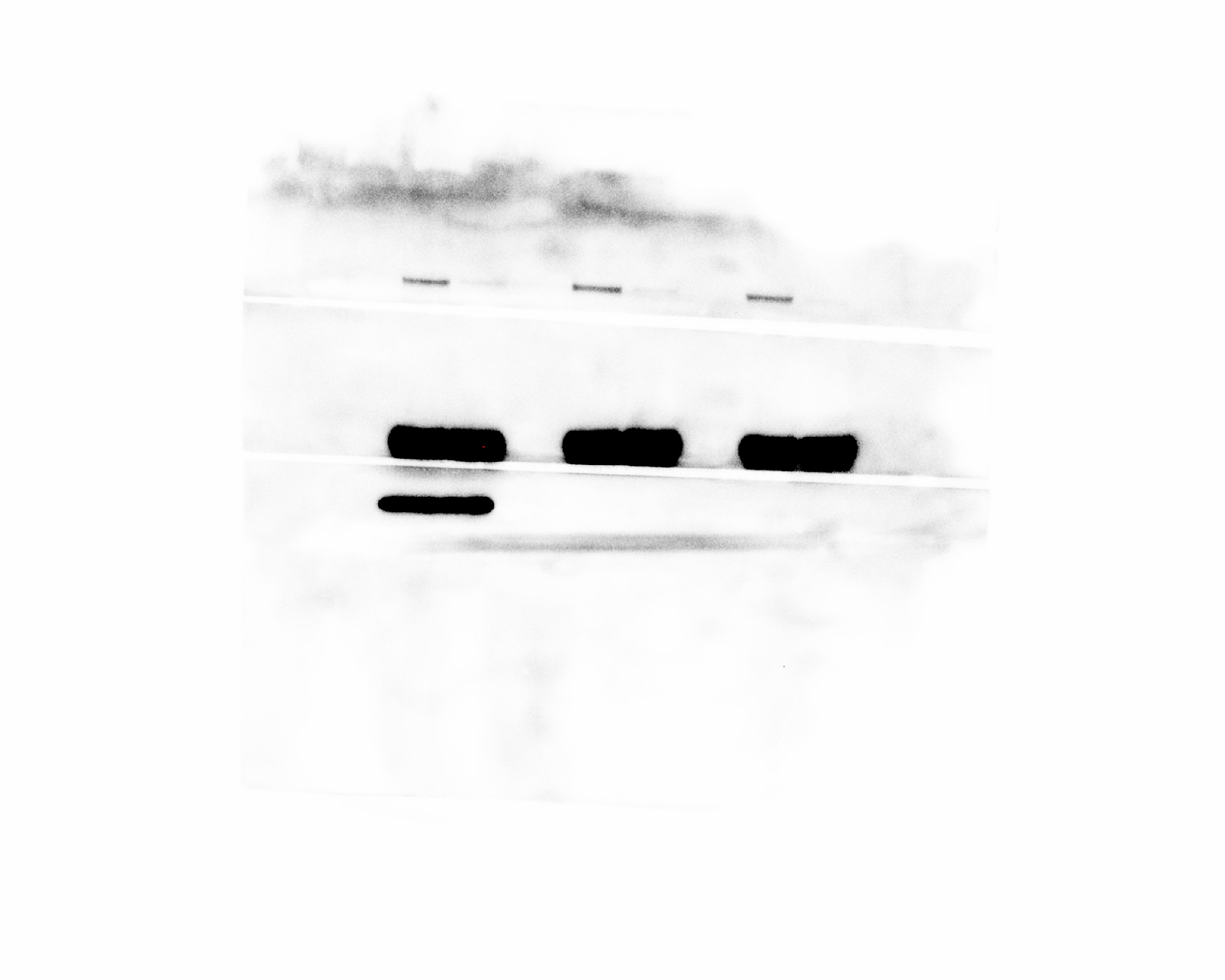

Supplement: Supplementary file 13 — Appendix Figure Source data [file 44318_2025_453_MOESM13_ESM.zip › Source data Appendix/Figure S3/S3C/ChemiDoc Images 2024-11-23_17.52.16/Rahul 2024-11-19 13h45m27s(Chemiluminescence).tif]

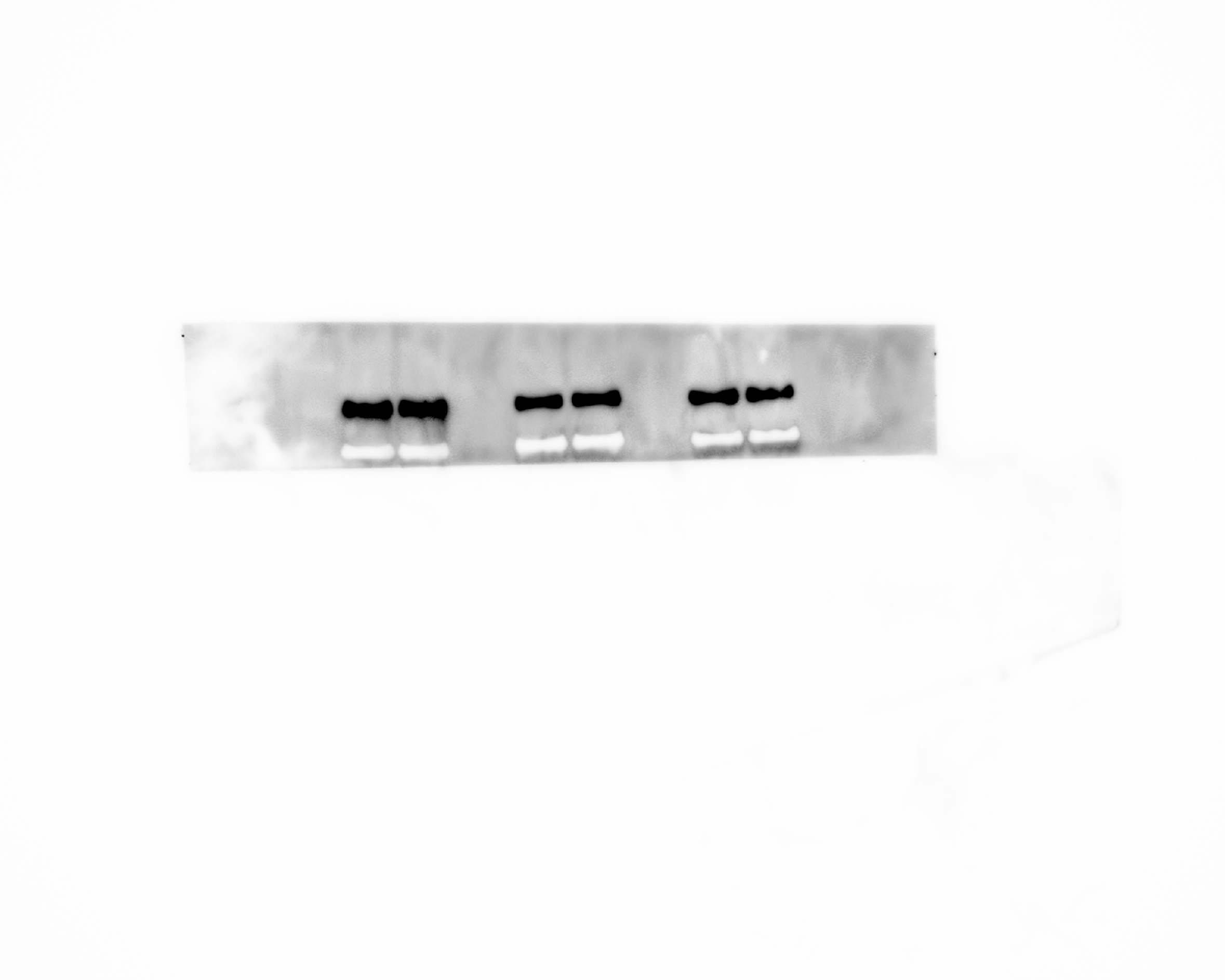

Supplement: Supplementary file 13 — Appendix Figure Source data [file 44318_2025_453_MOESM13_ESM.zip › Source data Appendix/Figure S3/S3C/ChemiDoc Images 2024-11-23_17.52.35/Rahul 2024-11-23 17h50m09s(Chemiluminescence).jpg]

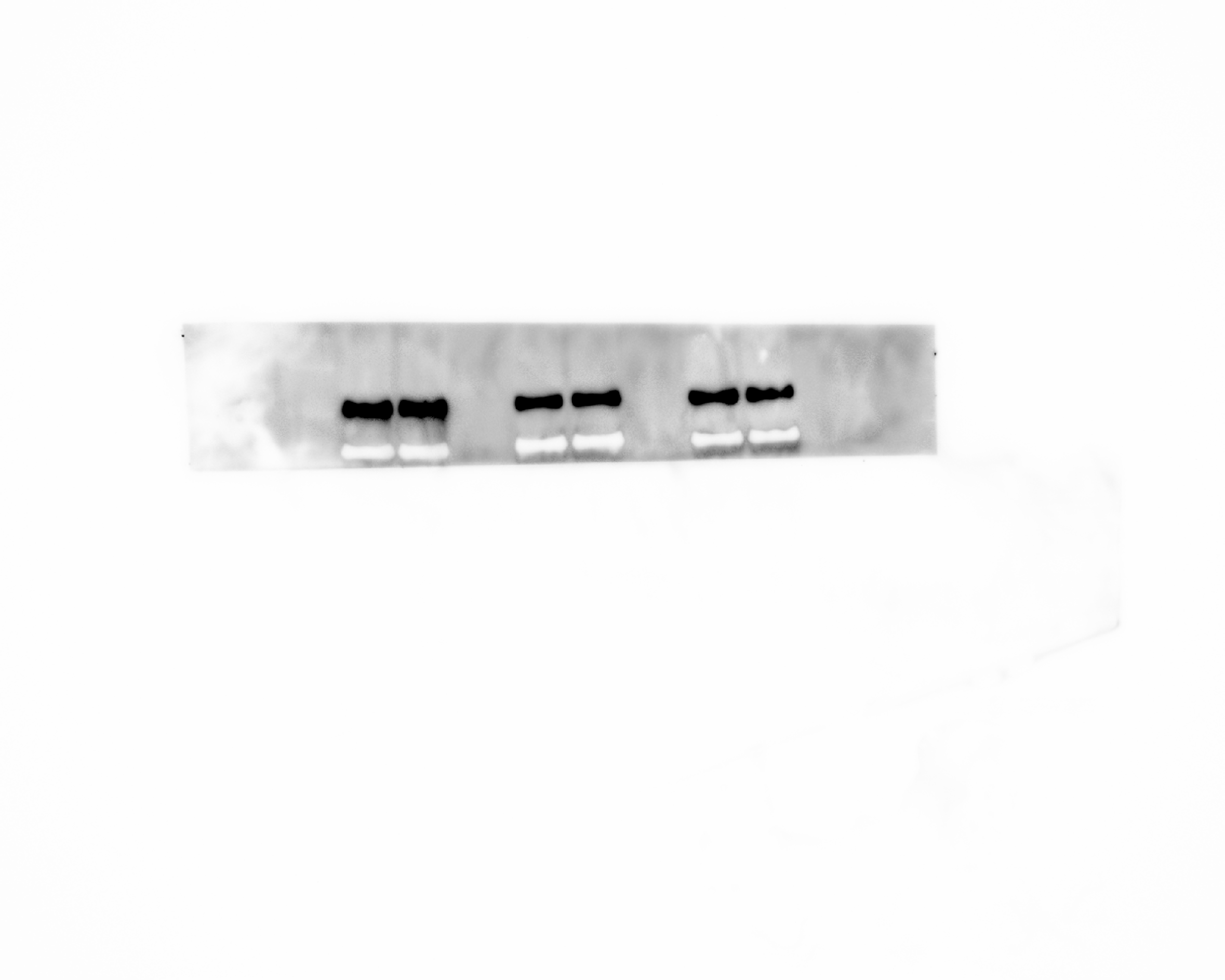

Supplement: Supplementary file 13 — Appendix Figure Source data [file 44318_2025_453_MOESM13_ESM.zip › Source data Appendix/Figure S3/S3C/ChemiDoc Images 2024-11-23_17.52.35/Rahul 2024-11-23 17h50m09s(Chemiluminescence).tif]

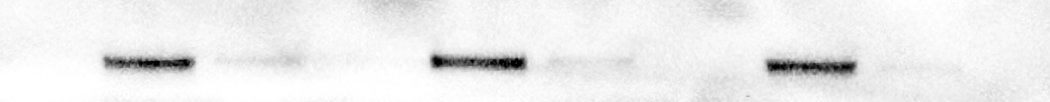

Supplement: Supplementary file 13 — Appendix Figure Source data [file 44318_2025_453_MOESM13_ESM.zip › Source data Appendix/Figure S3/S3C/CROPPED/blm.jpg]

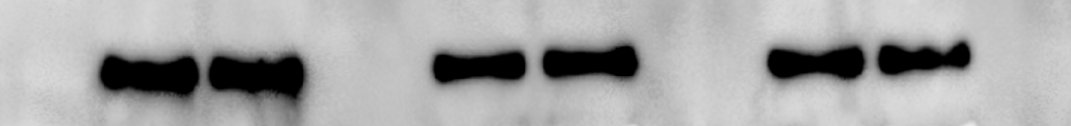

Supplement: Supplementary file 13 — Appendix Figure Source data [file 44318_2025_453_MOESM13_ESM.zip › Source data Appendix/Figure S3/S3C/CROPPED/chk1.jpg]

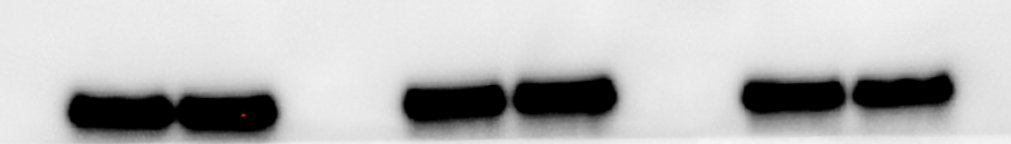

Supplement: Supplementary file 13 — Appendix Figure Source data [file 44318_2025_453_MOESM13_ESM.zip › Source data Appendix/Figure S3/S3C/CROPPED/gapdh.jpg]

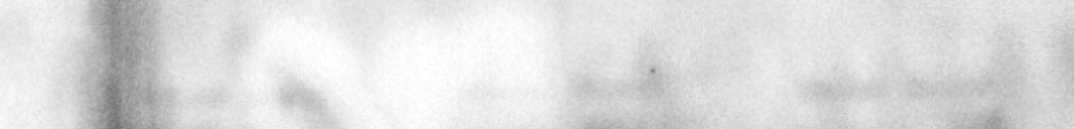

Supplement: Supplementary file 13 — Appendix Figure Source data [file 44318_2025_453_MOESM13_ESM.zip › Source data Appendix/Figure S3/S3C/CROPPED/pchk1.jpg]

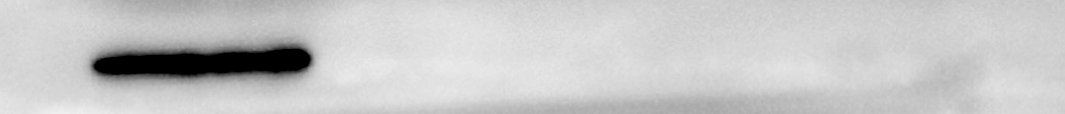

Supplement: Supplementary file 13 — Appendix Figure Source data [file 44318_2025_453_MOESM13_ESM.zip › Source data Appendix/Figure S3/S3C/CROPPED/phish3.jpg]
