# Supplementary material for: Mechanistic insight into anaphase bridge signaling to the abscission checkpoint
Source: EMBO J. 2025 May 12;44(13):3824–52. doi: 10.1038/s44318-025-00453-w (PMC12217976; doi:10.1038/s44318-025-00453-w)
Supplement: Supplementary file 14 — Expanded View Figures [file 44318_2025_453_MOESM14_ESM.pdf]

Expanded View Figures

Figure EV1. The absence of PICH induces a delay in the timing of abscission.

(**A**) Representative western blot of a whole cell lysate derived from U2OS cells treated with siControl, siBLM or siPICH for 48 h. (**B**) Experimental workflow for live-cell imaging experiments depicted in Fig. 1A–D. (**C**, **D**) Representative stills (**C**) and quantification (**D**) derived from live-cell imaging of U2OS cells (with no visible chromatin bridges) stably expressing fluorescently tagged histone H2B (red) and α-tubulin (green), treated with either siControl or siPICH for 48 h ($n = >60$). The yellow arrows indicate the site of abscission. Scale bar, 10 µm. The data are an average of three independent biological replicates with error bars representing the standard deviation. A Mann–Whitney test was performed to derive significance. Exact p value is <0.0001 (****). The data used for siCon in (**D**) are the same as used in Fig. 1B, as these experiments were performed together. (**E**) Representative immunofluorescence images and quantification of the total number of UFBs in U2OS cells during anaphase after treatment with siControl or siBLM for 48 h ($n = >50$). Scale bar, 5 µm. Significance was derived using a t-test. Exact p values are 0.0062 (**) between siCon/sicon+Aph, 0.012 (*) between siCon/siBLM, 0.008 (**) between siBLM/siBLM+Aph and 0.0002 (***) between siCon/siBLM+Aph. Source data are available online for this figure.

© The Author(s)

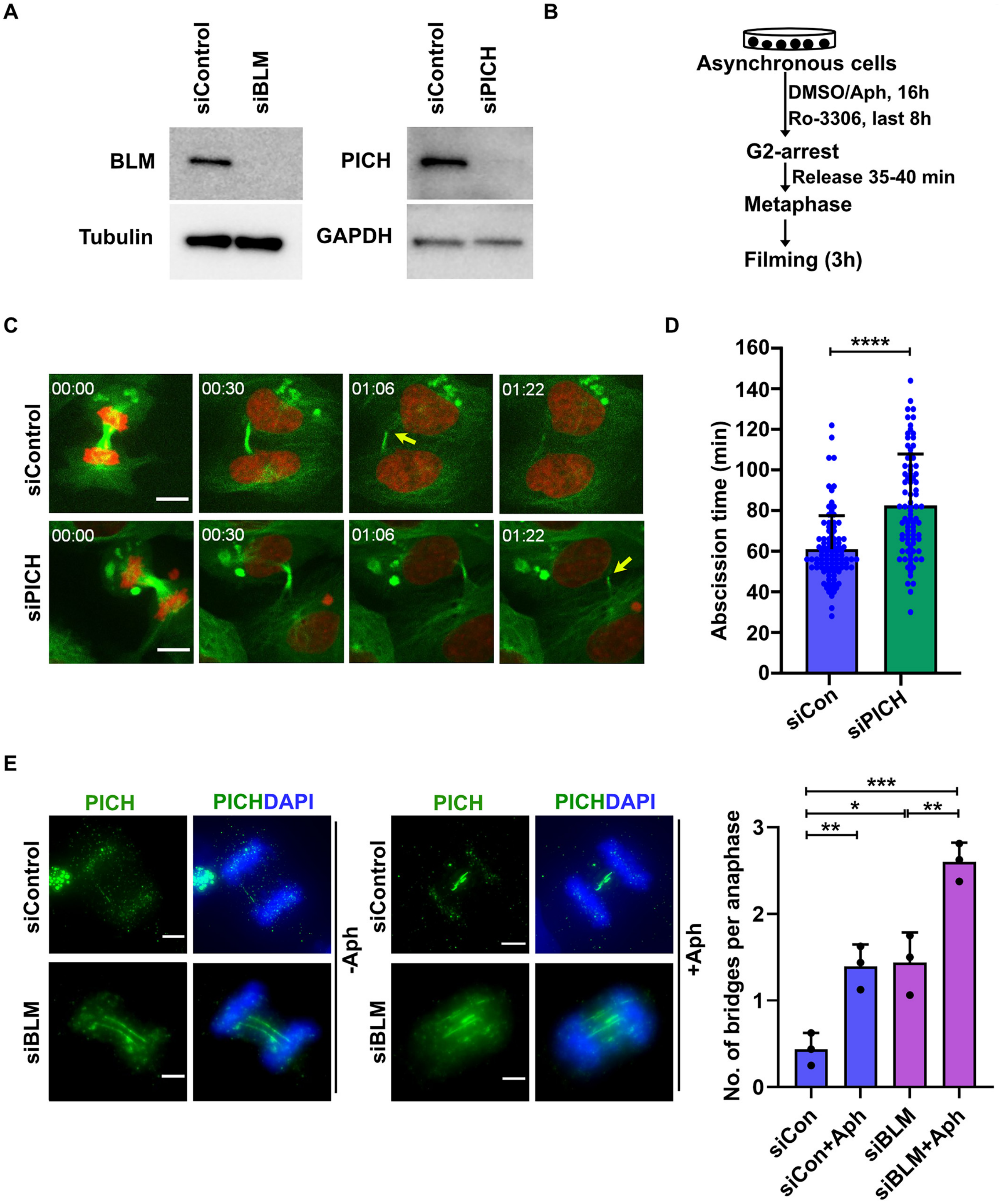
A
siControl
siBLM
BLM
Tubulin
siControl
siPICH
PICH
GAPDH
B
Asynchronous cells
DMSO/Aph, 16h
Ro-3306, last 8h
G2-arrest
Release 35-40 min
Metaphase
Filming (3h)
C
siControl
siPICH
00:00
00:30
01:06
01:22
D
Abscission time (min)
****
siCon
siPICH
E
PICH
PICHDAPI
siControl
siBLM
-Aph
+Aph
No. of bridges per anaphase
***
*
**
siCon
siCon+Aph
siBLM
siBLM+Aph

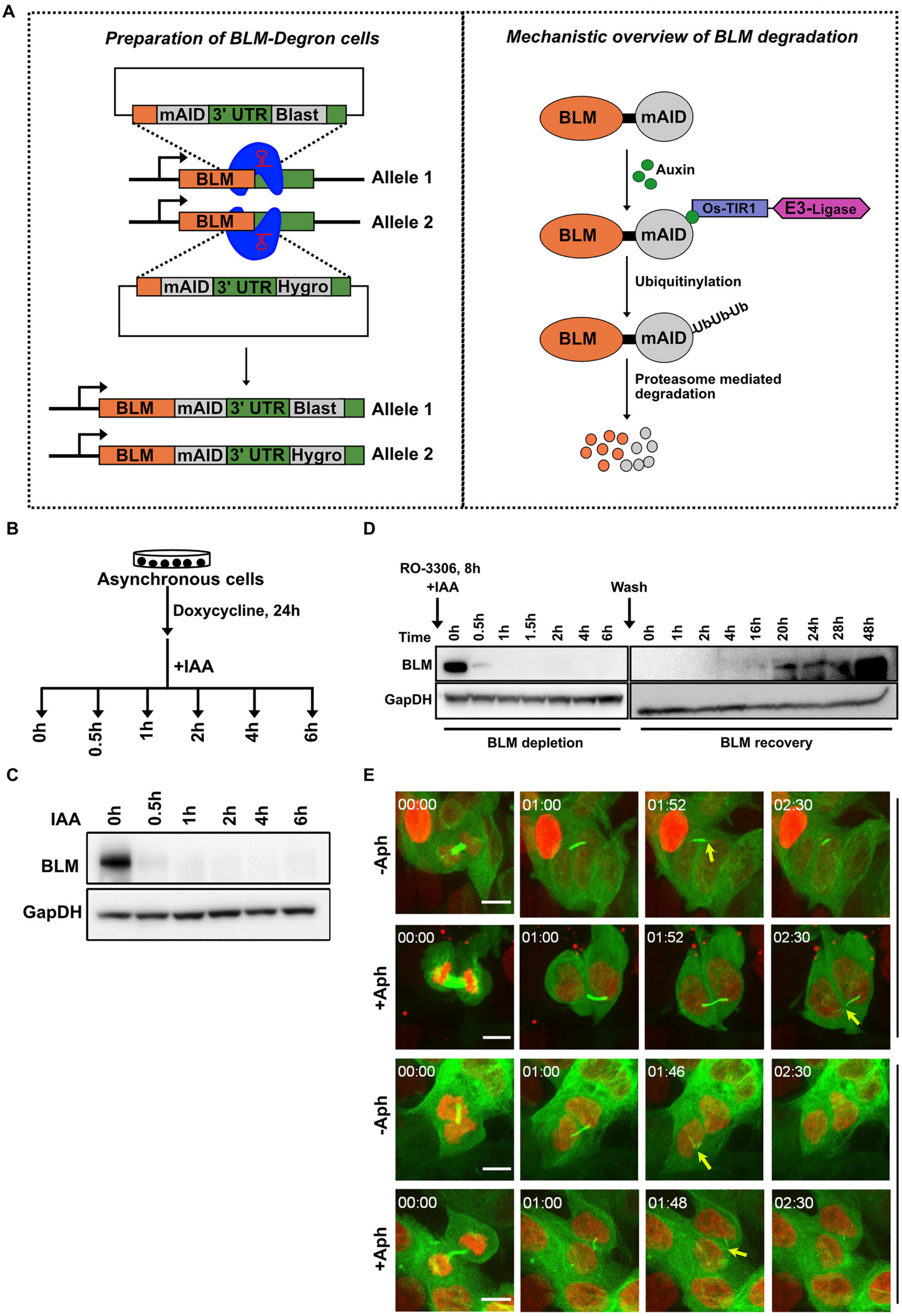
A
Preparation of BLM-Degron cells
mAID 3' UTR Blast
BLM
Allele 1
BLM
Allele 2
mAID 3' UTR Hygro
BLM mAID 3' UTR Blast Allele 1
BLM mAID 3' UTR Hygro Allele 2
Mechanistic overview of BLM degradation
BLM mAID
Auxin
Os-TIR1
E3-Ligase
BLM mAID
Ubiquitinylation
BLM mAID Ub-Ub-Ub
Proteasome mediated degradation
B
Asynchronous cells
Doxycycline, 24h
+IAA
0h 0.5h 1h 2h 4h 6h
C
IAA 0h 0.5h 1h 2h 4h 6h
BLM
GapDH
D
RO-3306, 8h
+IAA
Wash
Time 0h 0.5h 1h 1.5h 2h 4h 6h 0h 1h 2h 4h 16h 20h 24h 28h 48h
BLM
GapDH
BLM depletion
BLM recovery
E
00:00 01:00 01:52 02:30
-Aph
00:00 01:00 01:52 02:30
+Aph
-IAA
00:00 01:00 01:46 02:30
-Aph
00:00 01:00 01:48 02:30
+Aph
+IAA

Figure EV2. Mitotic degradation of BLM prevents replication stress-induced abscission delay.

(**A**) Depiction of the procedure for generating a BLM degron in DLD1 cells (left) and a graphical overview of the mechanism by which BLM undergoes degradation (right). (**B**) Experimental workflow for assessing BLM degradation over time in BLM degron cells upon IAA addition. (**C**) A representative western blot of whole cell lysates derived from BLM degron cells treated with IAA for the indicated times. The data are representative of two independent biological replicates. (**D**) A representative western blot of BLM degron cells, arrested in G2-M checkpoint with RO-3306 for 8 h before degrading the BLM by adding IAA (auxin). BLM depletion was monitored for up to 6 h after adding IAA. Cells were then washed and released with fresh warm media and BLM recovery was monitored for up to 48 h. (**E**) Representative still pictures derived from live-cell imaging of BLM degron cells (with no visible chromatin bridges) stably expressing fluorescently tagged α-tubulin (green) and histone H2B (red) treated with (+IAA) or without (-IAA). BLM degradation was induced exclusively in mitosis following exposure or not of cells to RS (0.3 μM Aph), as indicated in Fig. 1E. The yellow arrows indicate the site of abscission. Scale bar, 10 μm. Source data are available online for this figure.

 © The Author(s)

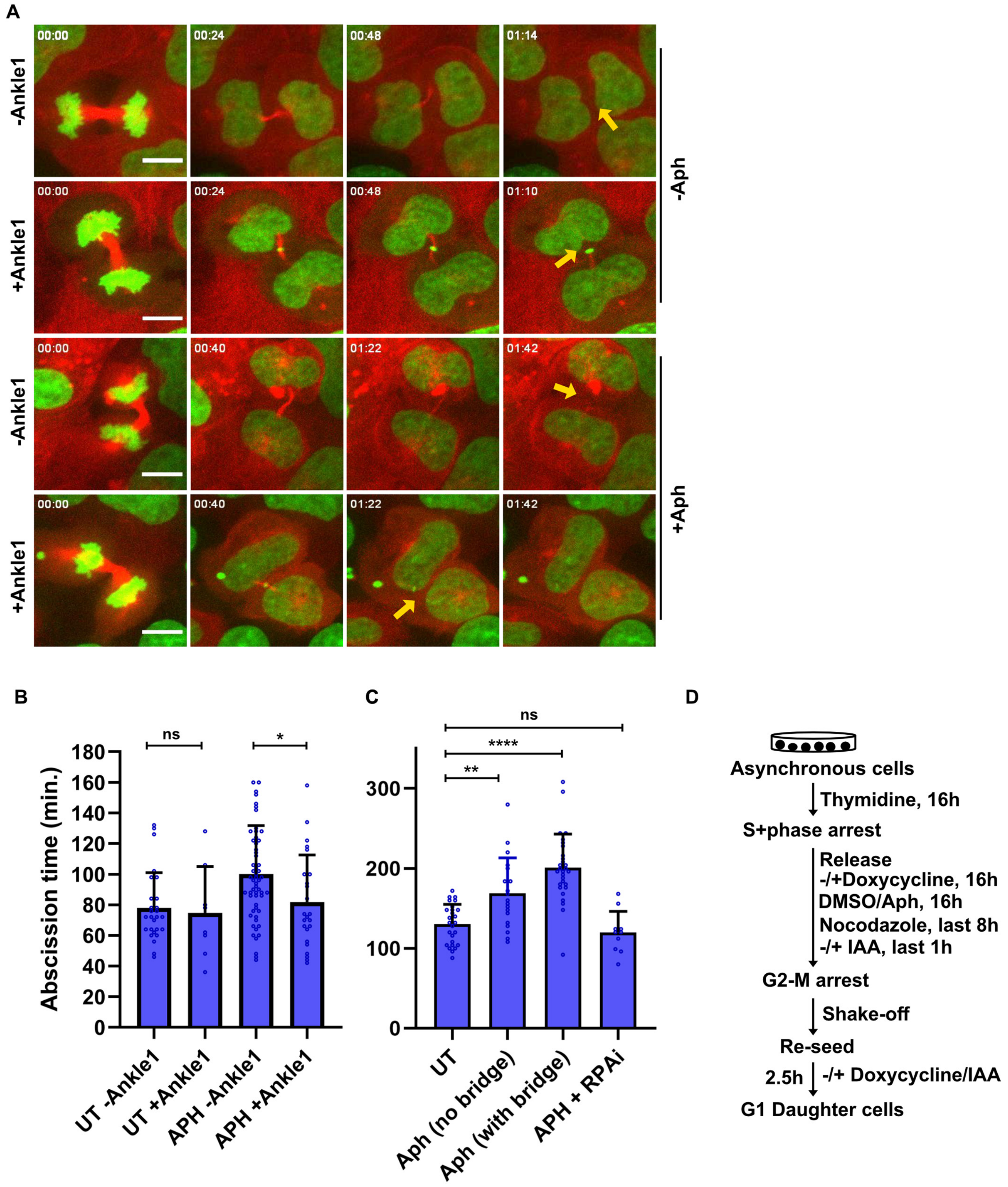
A
-Ankle1
+Ankle1
-Ankle1
+Ankle1
-Aph
+Aph
00:00
00:24
00:48
01:14
00:00
00:24
00:48
01:10
00:00
00:40
01:22
01:42
00:00
00:40
01:22
01:42
B
Abscission time (min.)
ns
*
UT -Ankle1
UT +Ankle1
APH -Ankle1
APH +Ankle1
C
ns
****
**
UT
Aph (no bridge)
Aph (with bridge)
APH + RPAi
D
Asynchronous cells
Thymidine, 16h
S+phase arrest
Release
-/+Doxycycline, 16h
DMSO/Aph, 16h
Nocodazole, last 8h
-/+ IAA, last 1h
G2-M arrest
Shake-off
Re-seed
2.5h
-/+ Doxycycline/IAA
G1 Daughter cells

Figure EV3. Expression of Ankle1 nuclease reduces the incidence of DNA bridges and prevents RS-induced abscission delay.

(**A**) Representative still pictures derived from live-cell imaging of U2OS cells (with no visible chromatin bridges) stably expressing fluorescently tagged histone H2B (green) and α-tubulin (red) and transiently expressing GFP-Ankel protein (which can be seen to localize to the midbody; in panels +Ankle1), in the presence (+Aph) or absence (-Aph) of RS (0.3 μM Aph). The yellow arrows indicate the site of abscission. Scale bar, 10 μm. (**B**) Quantification of abscission time derived from the live-cell imaging described in (**A**) (*n* => 10). The data are an average of two independent biological replicates with error bars representing the standard deviation. Mann-Whitney test was performed to derive significance. Exact *p* values are 0.89 (n.s.) and 0.014 (*). (**C**) Quantification of abscission time derived from the live-cell imaging of DLD1 cells expressing neon-PICH and mCherry-Tubulin along with SiR-DNA (*n* => 10). Mitotic cells under RS (APH), but having no visible PICH bridge, were analyzed separately from mitotic cells with visible PICH bridges and cells treated with RPAi (discussed later in the text). The data are an average of three independent biological replicates with error bars representing the standard deviation. Mann-Whitney test was performed to derive significance. Exact *p* values are 0.0016 (**) between UT/Aph (no bridge), <0.0001 (****) between UT/Aph (with bridge) and 0.289 (ns) between UT/Aph+RPAi. (**D**) Experimental workflow for assessing bi-nucleation and micronuclei formation with BLM degron cells with (+IAA) or without (-IAA) BLM degradation exclusively in mitosis in presence or absence of RS (0.3 μM Aph). Source data are available online for this figure.

 © The Author(s)

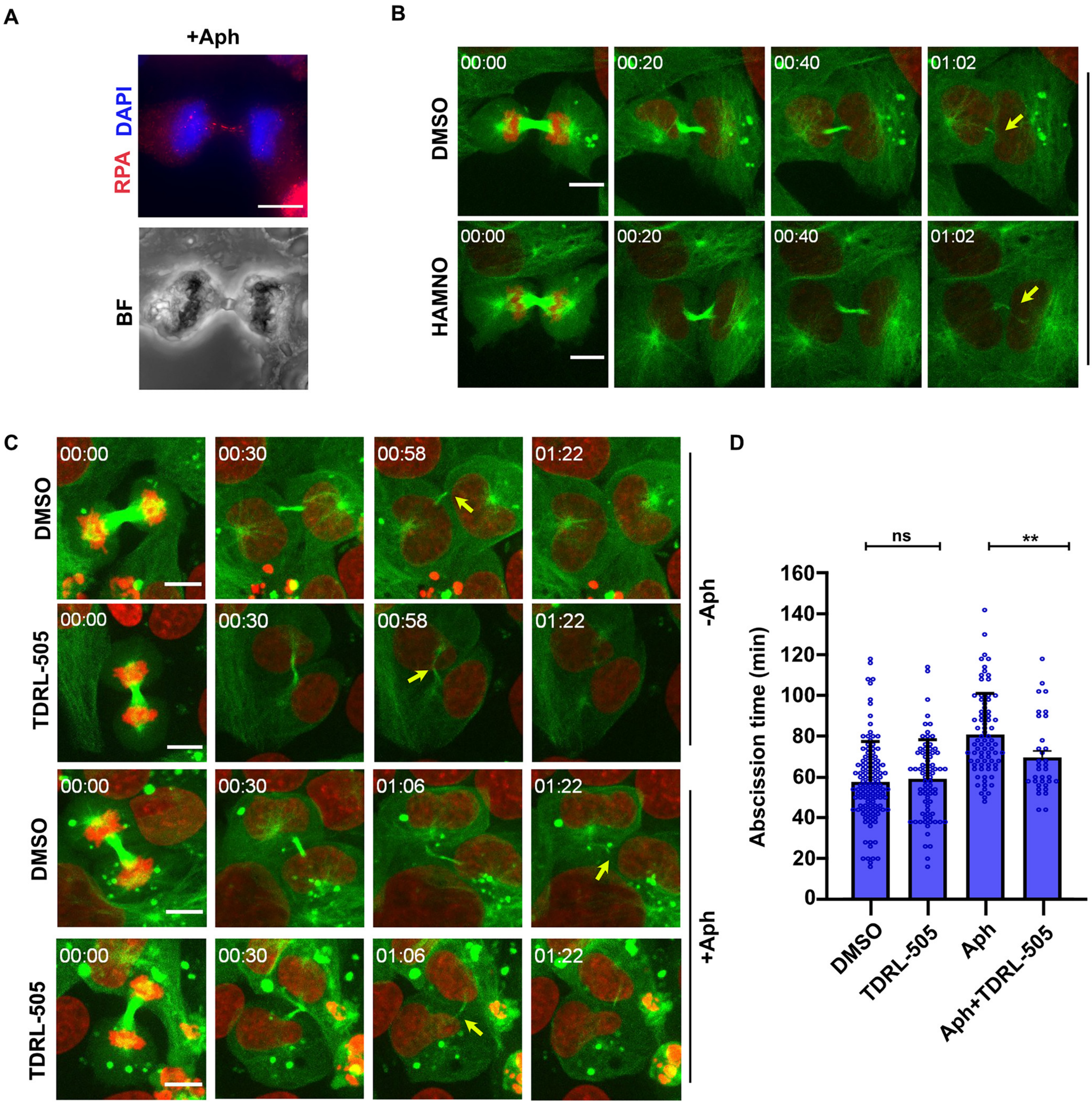

Figure EV4. Mitotic inhibition of RPA prevents replication stress-induced abscission delay.

(**A**) Representative immunofluorescence images (top) and bright field images (bottom) of the same U2OS cell (with visible midbody formation), treated with low dose Aph (0.3 μM during interphase) and stained with an anti-RPA antibody and DAPI. Scale bar, 10 μm. (**B**) Representative still pictures of live-cell imaging (n = >60) of U2OS cells (with no visible chromatin bridges) stably expressing fluorescently tagged histone H2B (red) and α-tubulin (green) treated with either DMSO or an RPA inhibitor (HAMNO, 50 μM) during mitosis in the absence of RS. The yellow arrows indicate the site of abscission. Scale bar, 10 μm. (**C**, **D**) Representative still pictures (**C**) and quantification (**D**) of live-cell imaging (n = >30) of U2OS cells (with no visible chromatin bridges) stably expressing fluorescently tagged histone H2B (red) and α-tubulin (green) treated with either DMSO or an RPA inhibitor (TDRL-505, 50 μM) during mitosis in the presence (+Aph) or absence (-Aph) of RS (0.3 μM Aph). The yellow arrows in (**C**), indicate the site of abscission. Scale bar, 10 μm. The data are an average of two independent biological replicates with error bars representing the standard deviation. A Mann-Whitney test was performed to derive significance. Exact p values are 0.490 (ns) and 0.0028 (**). Source data are available online for this figure.

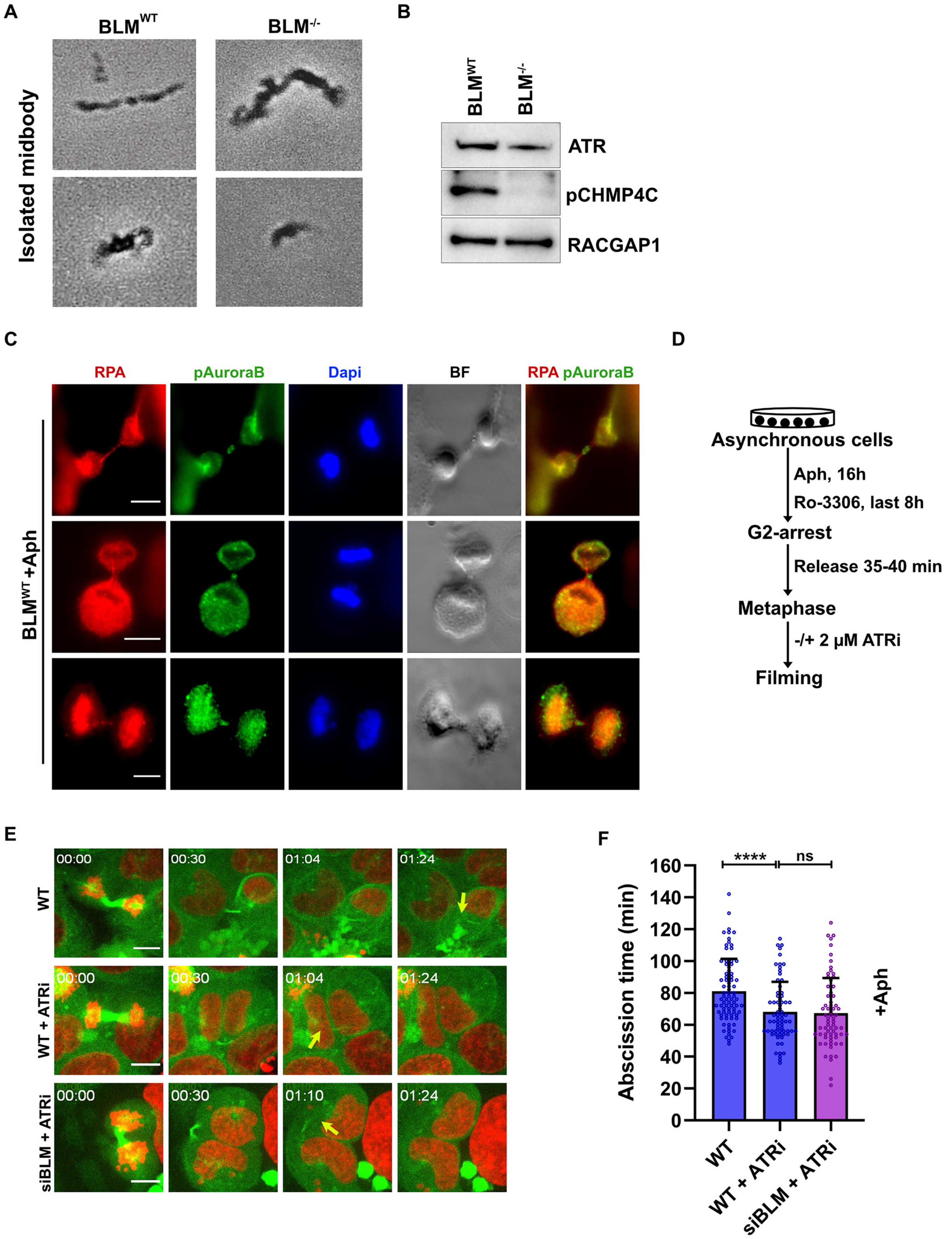
A
BLMWT
BLM-/-
Isolated midbody
B
BLMWT
BLM-/-
ATR
pCHMP4C
RACGAP1
C
RPA
pAuroraB
Dapi
BF
RPA pAuroraB
BLMWT +Aph
D
Asynchronous cells
Aph, 16h
Ro-3306, last 8h
G2-arrest
Release 35-40 min
Metaphase
-/+ 2 μM ATRi
Filming
E
WT
WT + ATRi
siBLM + ATRi
00:00
00:30
01:04
01:24
01:10
F
Abscission time (min)
0
20
40
60
80
100
120
140
160
****
ns
+Aph
WT
WT + ATRi
siBLM + ATRi

 © The Author(s)

Figure EV5. Mitotic inhibition of ATR prevents replication stress-induced abscission delay.

(**A**) Images of purified midbodies from BLM^{WT} and $BLM^{-/-}$ cells. (**B**) A representative western blot of midbody lysates derived from BLM^{WT} and $BLM^{-/-}$ U2OS cells with the indicated antibodies. The data are an average of two independent experiments. (**C**) Representative immunofluorescence images of BLM^{WT} cells showing an RPA coated persistent bridge passing through the midbody depicted through bright field (BF) microscopy, with active Aurora B (pThr-232) at the midbody and following replication stress. Scale bar, 10 µm. (**D–F**) Experimental workflow (**D**), still pictures (**E**) and quantification (**F**) of live-cell imaging ($n = >60$) of U2OS cells (with no visible chromatin bridges) stably expressing fluorescently tagged histone H2B (green) and α-tubulin (red) treated with either DMSO or an ATR inhibitor (2 µM, VE-822) during mitosis following exposure to RS (0.3 µM Aph) in interphase. The yellow arrows in (**E**) indicate the site of abscission. The data are an average of three independent experiments with error bars representing the standard deviation. A Mann-Whitney test was performed to derive significance. Exact p values are <0.0001 (****) and 0.554 (n.s.). Scale bar in (**E**), 10 µm. Source data are available online for this figure.
