# Supplementary material for: Mechanistic insight into anaphase bridge signaling to the abscission checkpoint
Source: EMBO J. 2025 May 12;44(13):3824–52. doi: 10.1038/s44318-025-00453-w (PMC12217976; doi:10.1038/s44318-025-00453-w)
Supplement: Supplementary file 2 — Peer Review File [file 44318_2025_453_MOESM2_ESM.pdf]

THE EMBO JOURNAL

MECHANISTIC INSIGHT INTO ANAPHASE BRIDGE SIGNALING TO THE ABSCISSION CHECKPOINT

Manika Singh, Girish Rajendraprasad, Vasileios Katopodis, Rui Cui, Marin Barisic, Rahul Bhowmick, and Ian Hickson

Corresponding author(s): Ian Hickson (iandh@sund.ku.dk) , Rahul Bhowmick (rahul.bhowmick@vanderbilt.edu)

Review Timeline:

Submission Date:	5th Feb 24
Editorial Decision:	8th Mar 24
Revision Received:	2nd Dec 24
Editorial Decision:	16th Jan 25
Revision Received:	16th Apr 25
Accepted:	24th Apr 25

Editor: Hartmut Vodermaier

Transaction Report:

(Note: With the exception of the correction of typographical or spelling errors that could be a source of ambiguity, letters and reports are not edited. Depending on transfer agreements, referee reports obtained elsewhere may or may not be included in this compilation. Referee reports are anonymous unless the Referee chooses to sign their reports.)

Prof. Ian D Hickson
University of Copenhagen
Center for Chromosome Stability, Department of Cellular and Molecular Medicine
Panum Institute
Blegdamsvej 3b
Copenhagen DK-2200
Denmark

8th Mar 2024

Re: EMBOJ-2024-116883
MECHANISTIC INSIGHT INTO ANAPHASE BRIDGE SIGNALING TO THE ABSCISSION CHECKPOINT

Dear Ian,

Thank you for submitting your study on BLM helicase signaling to the abscission checkpoint machinery for our consideration. Three expert referees have now assessed it and provided the below-copied reports. As you will see, all referees acknowledge the interest and potential importance of your findings, but also raise a number of conceptual and technical queries that would need to be addressed in order to make the study sufficiently conclusive. Recurrent key concerns are the concrete roles of BLM (and other players like PICH, RPA) in signaling to the abscission checkpoint components; the temporal order of events; and the apparent discrepancies with previous reports regarding requirements of particular factors/activities in different aspects of anaphase bridge and cytokinesis processes.

Should you be able to satisfactorily answer these questions, we would be happy to consider a revised version of the study further for The EMBO Journal. Since our single-major-revision-round policy makes it important to adequately clarify all raised issues at the time of resubmission, I would in this case invite you to get back to me with a tentative response letter and revision plan already during the early stages of the revision work; this would allow us to discuss (happily also via online call) how key concerns might best be addressed, and which points would be be the most relevant ones within the scope of the present study. I should add that we could also offer extension of the default three-months revision period if needed, with our 'scooping protection' (meaning that competing work appearing elsewhere in the meantime will not affect our considerations of your study) remaining of course valid also throughout such an extension.

Detailed information on preparing, formatting and uploading a revised manuscript can be found below and in our Guide to Authors. Thank you again for the opportunity to consider this work for The EMBO Journal, and I look forward to hearing from you in due time.

With kind regards,

Hartmut

Hartmut Vodermaier, PhD
Senior Editor, The EMBO Journal
h.vodermaier@embojournal.org

*** PLEASE NOTE: All revised manuscript are subject to initial checks for completeness and adherence to our formatting guidelines. Revisions may be returned to the authors and delayed in their editorial re-evaluation if they fail to comply to the following requirements (see also our Guide to Authors for further information):

1) Every manuscript requires a Data Availability section (even if only stating that no deposited datasets are included). Primary datasets or computer code produced in the current study have to be deposited in appropriate public repositories prior to resubmission, and reviewer access details provided in case that public access is not yet allowed. Further information: embopress.org/page/journal/14602075/authorguide#dataavailability

2) Each figure legend must specify
- size of the scale bars that are mandatory for all micrograph panels
- the statistical test used to generate error bars and P-values
- the type error bars (e.g., S.E.M., S.D.)
- the number (n) and nature (biological or technical replicate) of independent experiments underlying each data point
- Figures may not include error bars for experiments with n<3; scatter plots showing individual data points should be used

instead.

3) Revised manuscript text (including main tables, and figure legends for main and EV figures) has to be submitted as editable text file (e.g., .docx format). We encourage highlighting of changes (e.g., via text color) for the referees' reference.

4) Each main and each Expanded View (EV) figure should be uploaded as individual production-quality files (preferably in .eps, .tif, .jpg formats). For suggestions on figure preparation/layout, please refer to our Figure Preparation Guidelines: http://bit.ly/EMBOPressFigurePreparationGuideline

5) Point-by-point response letters should include the original referee comments in full together with your detailed responses to them (and to specific editor requests if applicable), and also be uploaded as editable (e.g., .docx) text files.

6) Please complete our Author Checklist, and make sure that information entered into the checklist is also reflected in the manuscript; the checklist will be available to readers as part of the Review Process File. A download link is found at the top of our Guide to Authors: embopress.org/page/journal/14602075/authorguide

7) All authors listed as (co-)corresponding need to deposit, in their respective author profiles in our submission system, a unique ORCiD identifier linked to their name. Please see our Guide to Authors for detailed instructions.

8) Please note that supplementary information at EMBO Press has been superseded by the 'Expanded View' for inclusion of additional figures, tables, movies or datasets; with up to five EV Figures being typeset and directly accessible in the HTML version of the article. For details and guidance, please refer to: embopress.org/page/journal/14602075/authorguide#expandedview

9) Digital image enhancement is acceptable practice, as long as it accurately represents the original data and conforms to community standards. If a figure has been subjected to significant electronic manipulation, this must be clearly noted in the figure legend and/or the 'Materials and Methods' section. The editors reserve the right to request original versions of figures and the original images that were used to assemble the figure. Finally, we generally encourage uploading of numerical as well as gel/blot image source data; for details see: embopress.org/page/journal/14602075/authorguide#sourcedata

At EMBO Press, we ask authors to provide source data for the main manuscript figures. Our source data coordinator will contact you to discuss which figure panels we would need source data for and will also provide you with helpful tips on how to upload and organize the files.

Further information is available in our Guide For Authors: https://www.embopress.org/page/journal/14602075/authorguide

In the interest of ensuring the conceptual advance provided by the work, we recommend submitting a revision within 3 months (6th Jun 2024). Please discuss the revision progress ahead of this time with the editor if you require more time to complete the revisions. Use the link below to submit your revision:

Link Not Available

Referee #1:

General summary:
In the manuscript entitled "Mechanistic Insight into Anaphase Bridge Signaling to the Abscission Checkpoint", the authors investigate a novel role and molecular mechanism of BLM, a key factor in preventing genome instability and a UFB-processing protein, in regulating the abscission checkpoint. The authors demonstrate that cells depleted of BLM, but not those depleted of other UFB-binding proteins (PICH or RIF1), exhibit a reduced abscission time, implying that BLM is uniquely necessary for abscission checkpoint activation. Furthermore, the authors reveal that BLM acts on double-stranded UFBs to generate single-stranded UFBs coated with RPA proteins. These RPA-coated UFBs lead to ATR-CHK1 activation, which functions upstream of Aurora B, subsequently governing the activation of the abscission checkpoint. Lastly, the authors present evidence that increased aneuploidy/polyploidy in BLM-deficient cells promotes anchorage-independent growth of RPE1 cells. Overall, the manuscript presents a compelling narrative on how BLM processes UFBs to activate the abscission checkpoint, thereby preventing genome instability. However, several concerns need to be addressed before the manuscript can be considered for publication.

Major concerns:

1. The authors focus on RS-induced UFBs in this study and conclude that BLM generates RPA-coated UFBs that induce CHK1 activation. However, aphidicolin has also been shown to induce chromatin bridges and lagging chromosomes (e.g., in PMID: 31395887 and PMID: 23811686). Similarly, BLM deficiency also leads to the induction of chromatin bridges (e.g., PMID: 17599064). According to the authors' model, the abscission checkpoint induced by trapped chromatin should not be affected by BLM depletion. It is therefore somewhat surprising to observe that, with aphidicolin treatment, depletion of BLM results in a significant reduction of abscission time compared to mock-depleted cells (e.g., Fig 1D). Is it possible that BLM is also involved in processing chromatin bridges to prevent their activation of the abscission checkpoint? Or there were only a limited number of chromatin bridges/lagging chromosomes induced in the authors' cell model upon Aph treatment? The authors should quantify abscission time by dividing the cells into two groups: those without chromatin bridges (CBs) and those with CBs. This means that Figure 1D should display the abscission time of (1) siCon without CBs; (2) siCon with CBs; (3) siBLM without CBs, and (4) siBLM with CBs. Presenting these results will help clarify the above issue.
2. In the first paragraph of the Results and Discussion section, the authors state that "we did not observe any reduction in abscission timing in BLM-depleted cells with persistent bridges compared to control cells having no persistent bridges." It is unclear whether "bridges" in this context refers to UFBs or chromatin bridges. Additionally, this statement is not supported by any data. The authors should provide the relevant data, as it represents an important result in the context of their findings.
3. The unexpected result that PICH depletion does not show reduced abscission time as BLM depletion requires further investigation. One possible explanation is that the depletion is incomplete, allowing for the presence of RPA-coated UFBs. In this case, BLM-coated UFBs would still be expected to appear in PICH-depleted cells, albeit at a reduced number. The authors should provide quantification for the experiment in Figure EV4E, comparing the number of both BLM-coated and RPA2-coated UFBs in PICH-depleted cells versus mock-depleted cells.
4. In Figure 3, the authors induce RPA-coated UFBs with a high dose of Aph and demonstrate that this treatment can induce abscission delay even when BLM is depleted. However, a key question arises: does a high dose of Aph also induce chromatin bridges or lagging chromosomes? The authors should address this to provide a comprehensive understanding of the effects of high-dose Aph treatment on abscission delay in BLM-depleted cells.
5. The results presented in Figures 5 and 6 may not be directly relevant to the abscission function of BLM. While Figure 5 concludes that BLM's role in cytokinesis prevents aneuploidy, it does not necessarily imply that only its role in cytokinesis is responsible for preventing aneuploidy. BLM has multiple functions in preventing genome instability, so the anchorage-independent growth of RPE1 p53-/- BLM-/- may also be due to other functions of BLM. Knocking out any gene that induces genome instability could be expected to yield similar results. The authors acknowledge that they cannot rule out the interphase role of BLM. To provide further support for the authors' hypothesis, I suggest including RPE1 p53-/- PICH-/- or RPE1 p53-/- RIF1-/- (with or without MYC expression) in the soft agar assays. If only BLM knockout promotes anchorage-independent growth, but not PICH-/- or RIF1-/- cells, it would strengthen the authors' argument regarding the specific role of BLM in this context.
6. A crucial control experiment that should be included is the complementation of BLM-deficient cells with a catalytically inactive BLM mutant, in order to assess whether it can rescue the observed reduction in abscission time. This will help confirm the essential role of BLM's helicase activity in the observed phenotypes, and effectively eliminate the possibility that other UFB proteins recruited by BLM might be accountable for the observed effects.
7. The discussion on the comparison of chromatin bridges and UFBs is indeed brief, and it would be beneficial to expand upon it. The authors cite and discuss Petsalaki et al. 2023 (PMID: 37638884), but they should also consider citing and discussing the previous paper from the same group, Petsalaki et al. 2021 (PMID: 33355621), which demonstrates that the ATM-CHK2 pathway activates the abscission checkpoint. This raises the question of whether ATR-CHK1 and ATM-CHK2 sense RPA-coated UFBs and chromatin bridges, respectively. It is important to clarify if BLM is also involved in chromatin bridge processing (as mentioned in point 1) and other types of UFBs (e.g., C-UFBs). It would be interesting for the authors to investigate whether BLM depletion also reduces abscission time when cells are treated with a low dose of ICRF-193, which induces both chromatin bridges and C-UFBs. Additionally, in Figures 4B and 4E, the authors should consider detecting the phosphorylation of CHK2 to provide a more comprehensive understanding of the pathways involved in sensing chromatin bridges and UFBs.

Minor concerns:
1. For Figure 1E, the authors should provide Western blotting results demonstrating that a short incubation of IAA after RO-3306 arrest and release can induce clear degradation of BLM. The Western blot in Figure EV2C is only for asynchronous cells, which may not accurately represent the situation when cells are arrested in the G2 phase.
2. When discussing Ankle1 nuclease migrating to the midbody during cytokinesis and its potential role in cutting unresolved or partially resolved DNA junctions during late mitosis (Brachner et al, 2012; Song et al, 2020), the authors should also cite a more relevant and recent cell biology paper about Ankle1: PMID: 36825683. Additionally, the authors should correct instances where Ankle1 is misspelled as Ankel1.
3. On page 12, the authors state that "Since removal of auxin allows restoration of BLM, it is expected that BLM would be functional when cells enter the subsequent S-phase...". The authors should provide a Western blot experiment to demonstrate that the removal of auxin indeed allows the restoration of BLM after nocodazole release.
4. In the Methods section, the authors mention using either a t-test or a Mann-Whitney test (type 1, 2, or 3) to calculate p-values. They should clarify whether they used a one-tailed or two-tailed test (presumably a two-tailed test). Additionally, the figure legends should indicate the specific statistical test used for each graph. It is also important to address the use of paired t-tests (type 1) in the study, as it appears that all the quantified data should be using unpaired tests. Ensuring that the correct statistical tests are employed for all quantified data is crucial.

Referee #2:

Singh et al submit a manuscript that proposes the role of BLM and RPA in the abscission checkpoint, the authors argue that these factors play a specific role in sensing the missegragated DNA derived from unresolved UFBs. An intriguing ramification of this work is that genetic deficiencies in BLM give rise to the Bloom's syndrome, that is characterized by genomic instability and increased susceptibility to cancer. This connection raises the possibility that defects in the abscission checkpoint could contribute to the pathologies associated with the Bloom's syndrome. The current work shows some significant weaknesses as it fails to connect the role of BLM to known components of the abscission checkpoint. Moreover, the key evidence connecting BLM to abscission regulation in response to persisting UFBs is indirect. The following issues need to be addressed to support the main conclusions:

Figure 1 and Figure 2 determine abscission time by live cell analysis of cells expressing fluorescently tagged tubulin. This is the right experimental approach but meaningful measures of abscission time rely on expressing reasonable labels of the tubulin constructs, as overexpression of tubulin perturbs abscission time. In this context, it is concerning to see large aggregates of fluorescent tubulin in the images and movies shown in these figures. Cell expressing appropriate levels of fluorescent tubulin should be used to repeat this analysis.

The authors conclude that BLM specifically delays abscission in those mitotic events where UFBs persist into cytokinesis, but this point is not unequivocally demonstrated in the manuscript as the authors mostly rely on indirect correlations. The authors need to show that UFBs persist until the time of cytokinetic abscission when BLM is depleted, and that abscission is not delayed in these particular events when BLM is depleted. In other words, a marker for missegredated DNA, such as the NE component Lap2B, should be used to monitor persistent DNA bridges, and determine by live cell microscopy that BLM is required specifically to regulate abscission in cells that have missegregated DNA.

Besides delaying abscission in response to missegregated DNA, the abscission checkpoint also delays abscission upon partial depletion of certain nucleoporins. If the model proposed by the authors is correct, BLM should have a specific role to delay abscission in response to unresolved UFBs while the response to Nup depletion should remain intact. This functional specificity needs to be addressed.

Figure 2E. The validation of the RPA inhibitors is simply not convincing. To show the specific effect of these inhibitors on cells with unresolved UFBs, the authors use cells stably expressing fluorescently tagged histone H2B to mark these DNA bridges. UFBs are thought to contain non-chromatinized DNA, thus the use of histone markers to label unresolved UFBs is not adequate.

Figure 4A. The authors argue that CHK1 activation by replication stress requires BLM, but a control without aphidicolin is needed in this experiment to show that CHK1 activation is a specific response to replication stress/missegregated DNA.

Figure 4E. The reduction of the Aurora B pT232 signal at the midbody in BLM deficient cells needs to be quantified properly, including a reasonable number of events. At the very least, aphidicolin-treated cells should be compared to a negative control to show the specificity of this phenotype. It is also important to establish in this experiment whether the Aurora B pT232 signal in the midbody is associated with missegregated DNA.

On a related issue, the connection between BLM/RPA with known components of the abscission checkpoint needs to be consolidated, as this part of the work is not convincing. For example, the authors should address whether RPA/BLM colocalize with active Aurora B (or other components of the abscission checkpoint) on UFB-related structures. It would also be important to determine whether CHMP4C is still phosphorylated in BLM-depleted cells treated with aphidicolin, as this type of evidence would establish a firm connection between BLM and downstream components of the abscission checkpoint.

EV Figure 5. The authors conclude that generation of RPA-coated UFBs is sufficient to induce abscission delay, but this is not supported by the data presented here. The use of RPA inhibitor suggests that RPA is needed for abscission delays, but this is not evidence that coating by RPA is sufficient to engage the abscission checkpoint.

other issues:

Figure 1 and others. Abscission time is represented as the average of three experiments with error bars representing the standard deviation. It is not clear from these graphs whether the increases in abscission time are driven by a small number of abscission events or the delays are observed across the entire population. This is important as one would expect that UFBs would persist in a subset of cells and these would be the events where abscission is delayed. The individual vales abscission should be represented in these graphs, or at least a graph showing the distribution of the values should be used.

The text states that CHMP4C is a member of the chromosome passenger complex. This is incorrect, as CHMP4C is an ESCRT-

III subunit that interacts with the chromosome passenger complex. In fact, the references on CHMP4C need to be revised to make sure the relevant papers are cited. For example, the citation of Petselaki et al 2018 in the introduction is misleading as this work does not describe the role of CHMP4C on abscission regulation.

Referee #3:

Singh et al perform a series of experiments leading to the conclusion that BLM helicase can regulate the activation of the abscission checkpoint, by generating the ssDNA necessary for RPA->ATR->Chk1 activation at mitosis. It is observed that BLM deficiency promotes tetraploidisation during cellular transformation, which promotes anchorage independent growth in presence of c-Myc oncogene. Tetraploid cells are more tumour like in this crude assay. Overall the manuscript is interesting, and links various previously described aspects of BLM biology, with a biological outcome that is relevant to the cancer predisposition phenotype of Bloom syndrome patients. It builds on the strength of previous studies by this lab on ultrafine anaphase bridges (UFAB), by mechanistically describing how a helicase could be linked to abscission checkpoint activation. There are only a couple things that require a small amount of additional investigation to support the main conclusions of the paper:

1) The main hypothesis is that BLM helicase activity is responsible for converting dsDNA at bridges to ssDNA, which goes on to allow RPA loading. However this has not been experimentally tested, and relies on the known function of RPA in binding ssDNA and BLM in unwinding DNA. One or more of the BLMsi experiments should be complemented with BLM-ATPase dead mutant. Additional data (although not essential, but would improve the impact) could be contributed by including additional BLM mutants at the same time: such as those recently described to separate the function of BLM in resection, TRR binding and dHJ dissolution (Shorrocks et al Nature Comms 2021, Hodson et al PNAS 2022, Xue et al Nature Comms 2022). not essential, but would increase
2) RPA coated bridges arise when PICH is depleted, even though BLM requires PICH for recruitment to UFAB. A potential explanation for this is that RPA is loaded because PICH is removed by BLM which exposes ssDNA, rather than BLM generating ssDNA directly (PICH accumulates on UFABs in absence of BLM, which supports this idea). One way to investigate this would be to see whether RPA still coats bridges when BLM degradation (with IAA or siRNA) is performed in absence of PICH. The results will support the hypothesis that it is direct ssDNA generation by BLM, or alternatively the unloading of PICH, that supports activation of the abscission checkpoint.
3) RO-3306 is a CDK1 inhibitor used to arrest cells in G2 so that the effects of BLM can be analysed in mitosis. But CDK1 phosphorylation of BLM is essential for its role in dissolution of catalytically interlinked DNA molecules (Pogliano, et al Science Advances 2022). Some discussion of this point should be included, and a Western blot showing BLM phosphorylation status during the "release" phase would indicate that the protein becomes "reactivated/rephosphorylated" after RO-3306 is removed (similar to Fig 1F of Pogliano et al). In other experiments, where Nocodazole is instead used to arrest the cells, the effects observed appear stronger than when RO-3306 is used - is there a direct side-by-side data for any result comparing the two methods?
4) Several figures contain bar graphs with rather underwhelming changes (less than 2-fold with large error bars) are then used to justify conclusions. Some are listed as "representative of three independent experiments" but does this mean it is 1) the result of a single experiment that is similar to other experiments, or 2) the average of three independent experiments. It should be (2), but the language is ambiguous. Furthermore, given the conclusions being drawn, the authors should include the individual values of each experiment (for example similar to what is shown in Fig 3C) for all the bar graphs of the manuscript, so the reader can see the spread of data.

Minor points to be addressed:
1) The methods section states that the statistical test used is indicated in each relevant figure legend, however none of the figure legends contain this information. Please address.
2) Figure 4E shows ATR and ATRIP blots that are blank. How do we know these antibodies worked in this experiment? The absence of bands does not indicate the absence of ATR and ATRIP, just that they can't be detected and this could be because the antibodies don't work. A control extract that is positive for these antibodies should be included, which is on the same gel as the samples investigated.
3) If possible, the data of figure 5 should be shown as a 2 dimensional plot of the primary FACS data with area vs width measurements of the channel used to collect PI stained cells. This would help discriminate tetraploid G1 from diploid G2 cells (which overlap in a single peak when viewed only in one direction), particularly in the +IAA sample.
4) Several studies have shown that BLM itself is a substrate of Chk1 kinase activity (eg Petsalaki JCB 2014, Kharat Oncogene 2016). Please include some discussion on what this might mean for the proposed model.

Dear Hartmut,

We would like to thank you for your editorial remarks and the referees for their very helpful comments on the manuscript. We have addressed all the comments that we are able to address experimentally and believe that the new analyses have helped considerably in improving the manuscript.

In the response below, the original comments of the referees are given in **bold** text, and our responses are given in blue text.

Best wishes,

Ian

Referee #1:

General summary:

In the manuscript entitled "Mechanistic Insight into Anaphase Bridge Signaling to the Abscission Checkpoint", the authors investigate a novel role and molecular mechanism of BLM, a key factor in preventing genome instability and a UFB-processing protein, in regulating the abscission checkpoint. The authors demonstrate that cells depleted of BLM, but not those depleted of other UFB-binding proteins (PICH or RIF1), exhibit a reduced abscission time, implying that BLM is uniquely necessary for abscission checkpoint activation. Furthermore, the authors reveal that BLM acts on double-stranded UFBs to generate single-stranded UFBs coated with RPA proteins. These RPA-coated UFBs lead to ATR-CHK1 activation, which functions upstream of Aurora B, subsequently governing the activation of the abscission checkpoint. Lastly, the authors present evidence that increased aneuploidy/polyploidy in BLM-deficient cells promotes anchorage-independent growth of RPE1 cells. Overall, the manuscript presents a compelling narrative on how BLM processes UFBs to activate the abscission checkpoint, thereby preventing genome instability. However, several concerns need to be addressed before the manuscript can be considered for publication.

We thank the reviewer for his/her insightful comments on our manuscript. We have carefully addressed the feedback provided, which we believe has significantly enhanced the clarity and impact of our findings regarding the unique role of BLM in activating the abscission checkpoint.

Major concerns:

1. The authors focus on RS-induced UFBs in this study and conclude that BLM generates RPA-coated UFBs that induce CHK1 activation. However, aphidicolin has also been shown to induce chromatin bridges and lagging chromosomes (e.g., in PMID: 31395887 and PMID: 23811686). Similarly, BLM deficiency also leads to the induction of chromatin bridges (e.g., PMID: 17599064). According to the authors' model, the abscission

checkpoint induced by trapped chromatin should not be affected by BLM depletion. It is therefore somewhat surprising to observe that, with aphidicolin treatment, depletion of BLM results in a significant reduction of abscission time compared to mock-depleted cells (e.g., Fig 1D). Is it possible that BLM is also involved in processing chromatin bridges to prevent their activation of the abscission checkpoint? Or there were only a limited number of chromatin bridges/lagging chromosomes induced in the authors' cell model upon Aph treatment? The authors should quantify abscission time by dividing the cells into two groups: those without chromatin bridges (CBs) and those with CBs. This means that Figure 1D should display the abscission time of (1) siCon without CBs; (2) siCon with CBs; (3) siBLM without CBs, and (4) siBLM with CBs. Presenting these results will help clarify the above issue.

We agree with the reviewer that treatment with Aphidicolin (Aph) induces chromatin bridges and lagging chromatin in a small subset of cells, and that depletion of BLM exacerbates this effect. However, we specifically focused on ultrafine bridges (UFBs). In our live cell experiments where we were measuring abscission time, we excluded any cell that exhibited a chromatin bridge, a point that we have emphasize further in the revised text in the results section. Previously, it was only apparent from reading the Methods section.

The main reason for excluding chromatin bridges from our quantification was that in a percentage of chromatin bridge-containing cells, these bridges eventually start to partially lose histones (perhaps due to increased tension) as anaphase progresses, resulting in the formation of stretches of non-chromatinized (UFB-like) DNA within an otherwise chromatin bridge. This complicates the overall scenario and scoring regime. We acknowledge the potential role of BLM in processing chromatin bridges and agree that exploring this aspect in future studies would be valuable, especially considering that the mechanisms underlying the dissolution of chromatin bridges remain elusive (and could be very different from those operating on UFBs).

We have tried to calculate the abscission timing of the cells containing chromatin bridges and plotted them as asked by the reviewer. This is a reviewer-only image. Indeed, chromatin bridges also delay abscission timing in our hands.

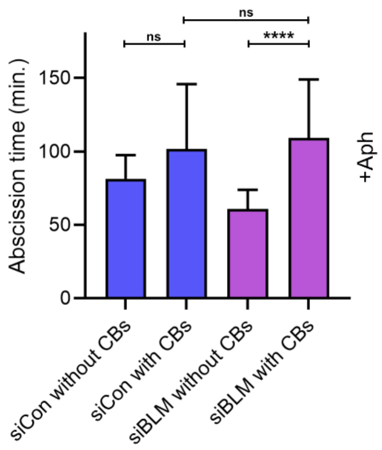

2. In the first paragraph of the Results and Discussion section, the authors state that "we did not observe any reduction in abscission timing in BLM-depleted cells with persistent bridges compared to control cells having no persistent bridges." It is unclear whether "bridges" in this context refers to UFBs or chromatin bridges. Additionally, this statement is not supported by any data. The authors should provide the relevant data, as it represents an important result in the context of their findings.

As discussed earlier, in this study we only focused on cells with UFBs and excluded any cell with a chromatin bridge. We have amended the text in the revised manuscript to clarify this point.

3. The unexpected result that PICH depletion does not show reduced abscission time as BLM depletion requires further investigation. One possible explanation is that the depletion is incomplete, allowing for the presence of RPA-coated UFBs. In this case, BLM-coated UFBs would still be expected to appear in PICH-depleted cells, albeit at a reduced number. The authors should provide quantification for the experiment in Figure EV4E, comparing the number of both BLM-coated and RPA2-coated UFBs in PICH-depleted cells versus mock-depleted cells.

We agree with the reviewer that these results seem unexpected. We believe that the difference in phenotype between BLM- and PICH-depleted cells results from the fact that PICH-depleted cells have been shown before to have small fraction of BLM bridges (PMID: 22563370). We have now explained the plausible cause of RPA bridges present in the PICH-depleted background in the revised manuscript.

4. In Figure 3, the authors induce RPA-coated UFBs with a high dose of Aph and demonstrate that this treatment can induce abscission delay even when BLM is depleted. However, a key question arises: does a high dose of Aph also induce chromatin bridges or lagging chromosomes? The authors should address this to provide a comprehensive understanding of the effects of high-dose Aph treatment on abscission delay in BLM-depleted cells.

As the reviewer correctly pointed out, high doses of aphidicolin do induce chromatin bridges and lagging chromatin in a small number of cells. Nevertheless, the formation of chromatin bridges or lagging chromosomes is inherently a rare event, unlike the formation of UFBs. Similar to all other live-cell experiments reported in this manuscript, when quantifying abscission timing for this experiment, we included only the cells that do not show any chromatin bridges. We have clearly mentioned this in the revised manuscript.

5. The results presented in Figures 5 and 6 may not be directly relevant to the abscission function of BLM. While Figure 5 concludes that BLM's role in cytokinesis prevents aneuploidy, it does not necessarily imply that only its role in cytokinesis is responsible for preventing aneuploidy. BLM has multiple functions in preventing genome instability, so the anchorage-independent growth of RPE1 p53-/- BLM-/- may also be due to other functions of BLM. Knocking out any gene that induces genome instability could be expected to yield similar results. The authors acknowledge that they cannot rule out the interphase role of BLM. To provide further support for the authors' hypothesis, I suggest

including RPE1 p53-/- PICH-/- or RPE1 p53-/- RIF1-/- (with or without MYC expression) in the soft agar assays. If only BLM knockout promotes anchorage-independent growth, but not PICH-/- or RIF1-/- cells, it would strengthen the authors' argument regarding the specific role of BLM in this context.

We appreciate the reviewer's suggestion and acknowledge the critical role of BLM in maintaining genome stability during interphase. In our experiments presented in Figure 5, we specifically focused on BLM's role during mitosis by degrading BLM exclusively at this stage. However, as noted in the manuscript, we cannot dismiss BLM's interphase role since the cells do not recover wild-type levels of BLM expression by S phase following mitotic degradation.

Currently, we are investigating this aspect to better understand the distinct roles of BLM in both mitosis and interphase, particularly concerning genome instability. As we are still in the early stages of this investigation, a more comprehensive discussion is beyond the scope of this manuscript. In the revised version, we have clarified that the effects we observed may relate to BLM's overall role in maintaining genome stability, which could be associated with or independent of its role during interphase.

6. A crucial control experiment that should be included is the complementation of BLM-deficient cells with a catalytically inactive BLM mutant, in order to assess whether it can rescue the observed reduction in abscission time. This will help confirm the essential role of BLM's helicase activity in the observed phenotypes, and effectively eliminate the possibility that other UFB proteins recruited by BLM might be accountable for the observed effects.

As recommended by the reviewer, we have tested the effect of expressing a BLM ATPase-dead variant of BLM in the $BLM^{-/-}$ cells and analyzed abscission timing. In the revised manuscript, we show that the BLM ATPase-dead variant does not induce an abscission delay (Figure 2G/H), confirming the role of BLM's helicase activity in triggering abscission delay.

7. The discussion on the comparison of chromatin bridges and UFBs is indeed brief, and it would be beneficial to expand upon it. The authors cite and discuss Petsalaki et al. 2023 (PMID: 37638884), but they should also consider citing and discussing the previous paper from the same group, Petsalaki et al. 2021 (PMID: 33355621), which demonstrates that the ATM-CHK2 pathway activates the abscission checkpoint. This raises the question of whether ATR-CHK1 and ATM-CHK2 sense RPA-coated UFBs and chromatin bridges, respectively. It is important to clarify if BLM is also involved in chromatin bridge processing (as mentioned in point 1) and other types of UFBs (e.g., C-UFBs). It would be interesting for the authors to investigate whether BLM depletion also reduces abscission time when cells are treated with a low dose of ICRF-193, which induces both chromatin bridges and C-UFBs. Additionally, in Figures 4B and 4E, the authors should consider detecting the phosphorylation of CHK2 to provide a more comprehensive understanding of the pathways involved in sensing chromatin bridges and UFBs.

In the revised manuscript, we have expanded the discussion regarding the comparison of chromatin bridges and ultrafine bridges (UFBs). We thank the reviewer for mentioning Petsalaki et al. (2021) (PMID: 33355621), which demonstrates that the ATM-CHK2 pathway activates the

abscission checkpoint. As hypothesized by the reviewer, it is possible that the BLM-RPA-ATR axis controls the UFB-induced checkpoint, whereas ATM-CHK2 induces the chromatin bridge-induced checkpoint. The low dose aphidicolin treatment used in our manuscript primarily induces UFBs, while chromatin bridges are seen in only a small subset of cells. Mackay et al. (PMID: 25904336) previously described that low-dose aphidicolin-induced abscission delay is not dependent on ATM; rather, it is dependent on ATR-CHK1. In the revised manuscript, we have discussed all these findings in the discussion section.

Minor concerns:

1. For Figure 1E, the authors should provide Western blotting results demonstrating that a short incubation of IAA after RO-3306 arrest and release can induce clear degradation of BLM. The Western blot in Figure EV2C is only for asynchronous cells, which may not accurately represent the situation when cells are arrested in the G2 phase.

In the revised manuscript, we have included a western blot indicating degradation of BLM in G2 arrested cells (Figure EV2D).

2. When discussing Ankle1 nuclease migrating to the midbody during cytokinesis and its potential role in cutting unresolved or partially resolved DNA junctions during late mitosis (Brachner et al, 2012; Song et al, 2020), the authors should also cite a more relevant and recent cell biology paper about Ankle1: PMID: 36825683. Additionally, the authors should correct instances where Ankle1 is misspelled as Ankel1.

We thank the reviewer for pointing out a more appropriate reference. In the revised manuscript we have included PMID: 36825683 and also amended the spelling of Ankle1.

3. On page 12, the authors state that "Since removal of auxin allows restoration of BLM, it is expected that BLM would be functional when cells enter the subsequent S-phase...". The authors should provide a Western blot experiment to demonstrate that the removal of auxin indeed allows the restoration of BLM after nocodazole release.

In the revised manuscript, we have included a western blot demonstrating the rate of recovery of BLM after removal of auxin (Supplementary EV2D).

4. In the Methods section, the authors mention using either a t-test or a Mann-Whitney test (type 1, 2, or 3) to calculate p-values. They should clarify whether they used a one-tailed or two-tailed test (presumably a two-tailed test). Additionally, the figure legends should indicate the specific statistical test used for each graph. It is also important to address the use of paired t-tests (type 1) in the study, as it appears that all the quantified data should be using unpaired tests. Ensuring that the correct statistical tests are employed for all quantified data is crucial.

As recommended by the reviewer, we have now included the statistical methods used for each experiment in the figure legends.

Referee #2:

Singh et al submit a manuscript that proposes the role of BLM and RPA in the abscission checkpoint, the authors argue that these factors play a specific role in sensing the missegragated DNA derived from unresolved UFBs. An intriguing ramification of this work is that genetic deficiencies in BLM give rise to the Bloom's syndrome, that is characterized by genomic instability and increased susceptibility to cancer. This connection raises the possibility that defects in the abscission checkpoint could contribute to the pathologies associated with the Bloom's syndrome. The current work shows some significant weaknesses as it fails to connect the role of BLM to known components of the abscission checkpoint. Moreover, the key evidence connecting BLM to abscission regulation in response to persisting UFBs is indirect. The following issues need to be addressed to support the main conclusions:

Figure 1 and Figure 2 determine abscission time by live cell analysis of cells expressing fluorescently tagged tubulin. This is the right experimental approach but meaningful measures of abscission time rely on expressing reasonable labels of the tubulin constructs, as overexpression of tubulin perturbs abscission time. In this context, it is concerning to see large aggregates of fluorescent tubulin in the images and movies shown in these figures. Cell expressing appropriate levels of fluorescent tubulin should be used to repeat this analysis.

We agree with the reviewer that differences in tubulin expression could potentially cause variations in abscission timing. However, as demonstrated in figure below (reviewer-only figure), the level of mCherry-tagged tubulin is significantly less than that of the un-tagged endogenous tubulin. Since we used the same U2OS cell lines, with and without siBLM or +/- RPAi, the observed differences in abscission time are very unlikely to be attributable to tubulin expression.

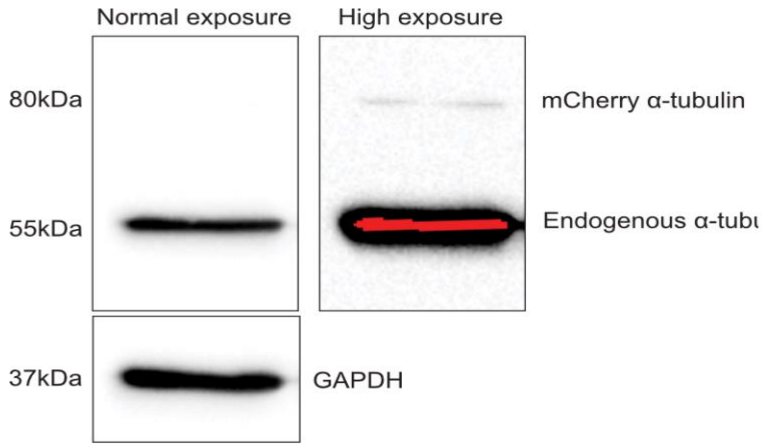

The authors conclude that BLM specifically delays abscission in those mitotic events where UFBs persist into cytokinesis, but this point is not unequivocally demonstrated in the manuscript as the authors mostly rely on indirect correlations. The authors need to show that UFBs persist until the time of cytokinetic abscission when BLM is depleted, and that abscission is not delayed in these particular events when BLM is depleted. In other words, a marker for missegredated DNA, such as the NE component Lap2B, should be used to monitor persistent DNA bridges, and determine by live cell microscopy that BLM is required specifically to regulate abscission in cells that have missegregated DNA.

Although Lap2b has been previously used to monitor chromatin and cytoplasmic bridges, it is not suitable for distinguishing these bridges from ultra-fine DNA bridges (UFBs). The use of Lap2b staining could complicate the analysis, as it does not specifically differentiate between UFBs and other types of bridges. In our study, we attempted to overcome this limitation by tagging PICH with a fluorescent protein, mNeon, to observe the fate of PICH-coated UFBs in BLM knockout (BLM-KO) cells. However, we encountered a challenge with Neon fluorescence, which tends to bleach significantly before cells reach telophase. This bleaching effect prevented us from directly correlating the persistence of UFBs with delays in abscission. Even with RPE $BLM^{-/-}$ GFP BLMwt cells (new data added; Figure 2H), the BLM bridges get resolved before or around telophase to single-stranded DNA, which makes it impossible to locate bridges at the midbody. However, our Immunofluorescence data (Figure EV4A) shows that RPA bridges persist until midbody formation.

Besides delaying abscission in response to missegregated DNA, the abscission checkpoint also delays abscission upon partial depletion of certain nucleoporins. If the model proposed by the authors is correct, BLM should have a specific role to delay abscission in response to unresolved UFBs while the response to Nup depletion should

We appreciate the reviewer for bringing up this interesting aspect. However, thoroughly dissecting the impact of persistent ultrafine bridges (UFB) and nucleoporin depletion on abscission timing would require a substantial amount of experimentation. Given that the main focus of our study is on UFBs, we believe that analyzing this in detail would be beyond the scope of the current manuscript.

Figure 2E. The validation of the RPA inhibitors is simply not convincing. To show the specific effect of these inhibitors on cells with unresolved UFBs, the authors use cells stably expressing fluorescently tagged histone H2B to mark these DNA bridges. UFBs are thought to contain non-chromatinized DNA, thus the use of histone markers to label unresolved UFBs is not adequate.

Fig 2E is not depicting the validation of RPA inhibitors. It represents frozen frames from live cell imaging, showing the different stages of mitosis used to calculate abscission timing upon treatment with RPA inhibitors. Thes inhibitors have been extensively validated (PMID: 25070753, PMID: 20395205). We didn’t use fluorescently tagged histone H2B to mark UFBs in any of our experiments. We have clarified in several places in the text that we only studied chromatin-free UFBs and excluded chromatinized bridges.

Figure 4A. The authors argue that CHK1 activation by replication stress requires BLM, but a control without aphidicolin is needed in this experiment to show that CHK1 activation is a specific response to replication stress/missegregated DNA.

In the revised manuscript, we have included the suggested control (Appendix Figure S2C).

Figure 4E. The reduction of the Aurora B pT232 signal at the midbody in BLM deficient cells needs to be quantified properly, including a reasonable number of events. At the very least, aphidicolin-treated cells should be compared to a negative control to show the specificity of this phenotype. It is also important to establish in this experiment whether the Aurora B pT232 signal in the midbody is associated with missegregated DNA.

On a related issue, the connection between BLM/RPA with known components of the abscission checkpoint needs to be consolidated, as this part of the work is not convincing. For example, the authors should address whether RPA/BLM colocalize with active Aurora B (or other components of the abscission checkpoint) on UFB-related structures. It would also be important to determine whether CHMP4C is still phosphorylated in BLM-depleted cells treated with aphidicolin, as this type of evidence would establish a firm connection between BLM and downstream components of the abscission checkpoint.

We agree with the reviewer that a quantification seems necessary, but as explained in the manuscript, the pThr-232 signal was present on the arms of the midbody and not at its center, it was difficult to quantify this difference by reference to a control protein (such as RACGAP1) that lies at the midbody center.

We agree that there isn’t direct evidence of RPA colocalizing with the Aurora B at the midbody. However, Figure EV4A shows that the RPA bridge passes through the midbody. It is reasonable to assume, therefore, that RPA bridge could activate ATR-Chk1-AuroraB at the midbody.

We have also added the CHMP4C phosphorylation data from the isolated midbody after RS, from BLM^{WT} and $BLM^{-/-}$ cells (Figure EV5B). We also quantified the percentage of cells that have a pCHMP4C signal at the midbody in $BLM^{-/-}$ cells. The data below are reviewer-only data. These data are an average of two independent experiments.

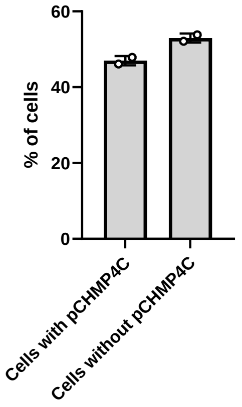

EV Figure 5. The authors conclude that generation of RPA-coated UFBs is sufficient to induce abscission delay, but this is not supported by the data presented here. The use of RPA inhibitor suggests that RPA is needed for abscission delays, but this is not evidence that coating by RPA is sufficient to engage the abscission checkpoint.

We acknowledge the reviewer's point that we do not have direct evidence demonstrating that coating by RPA alone is enough to activate the abscission checkpoint. However, we have demonstrated that RPA-mediated Chk1 activation plays a crucial role in activating the abscission checkpoint. There is extensive literature supporting the idea that the coating of RPA on DNA is necessary to trigger ATR-Chk1 activation, which aligns with our hypothesis. We have discussed this in the revised manuscript.

other issues:

Figure 1 and others. Abscission time is represented as the average of three experiments with error bars representing the standard deviation. It is not clear from these graphs whether the increases in abscission time are driven by a small number of abscission events or the delays are observed across the entire population. This is important as one would expect that UFBs would persist in a subset of cells and these would be the events where abscission is delayed. The individual vales abscission should be represented in these graphs, or at least a graph showing the distribution of the values should be used.

All the individual abscission time values are represented in the source data file.

The text states that CHMP4C is a member of the chromosome passenger complex. This is incorrect, as CHMP4C is an ESCRT-III subunit that interacts with the chromosome passenger complex. In fact, the references on CHMP4C need to be revised to make sure the relevant papers are cited. For example, the citation of Petselaki et al 2018 in the introduction is misleading as this work does not describe the role of CHMP4C on abscission regulation.

We have amended the text and excluded the suggested references.

Referee #3:

Singh et al perform a series of experiments leading to the conclusion that BLM helicase can regulate the activation of the abscission checkpoint, by generating the ssDNA necessary for RPA->ATR->Chk1 activation at mitosis. It is observed that BLM deficiency promotes tetraploidisation during cellular transformation, which promotes anchorage independent growth in presence of c-Myc oncogene. Tetraploid cells are more tumour like in this crude assay. Overall the manuscript is interesting, and links various previously described aspects of BLM biology, with a biological outcome that is relevant to the cancer predisposition phenotype of Bloom syndrome patients. It builds on the strength of previous studies by this lab on ultrafine anaphase bridges (UFAB), by mechanistically describing how a helicase could be linked to abscission checkpoint activation. There are only a couple things that require a small amount of additional investigation to support the main conclusions of the paper:

We thank the reviewer for the encouraging comments.

1) The main hypothesis is that BLM helicase activity is responsible for converting dsDNA at bridges to ssDNA, which goes on to allow RPA loading. However, this has not been experimentally tested, and relies on the known function of RPA in binding ssDNA and BLM in unwinding DNA. One or more of the siBLM experiments should be complemented with BLM-ATPase dead mutant. Additional data (although not essential, but would improve the impact) could be contributed by including additional BLM mutants at the same time: such as those recently described to separate the function of BLM in resection, TRR binding and dHJ dissolution (Shorrocks et al Nature Comms 2021, Hodson et al PNAS 2022, Xue et al Nature Comms 2022). not essential, but would increase

We thank the reviewer for pointing out this weakness of our manuscript. In the revised manuscript, we have tested the effect of overexpressing a BLM ATPase-dead variant in BLM-/- cells. We found that the ATPase-dead BLM mutant does not induce abscission delay (Figure 2G/H), confirming the role of BLM's helicase activity in triggering abscission delay.

2) RPA coated bridges arise when PICH is depleted, even though BLM requires PICH for recruitment to UFB. A potential explanation for this is that RPA is loaded because PICH is removed by BLM which exposes ssDNA, rather than BLM generating ssDNA directly (PICH accumulates on UFBs in the absence of BLM, which supports this idea). One way to investigate this would be to see whether RPA still coats bridges when BLM degradation (with IAA or siRNA) is performed in absence of PICH. The results will support the hypothesis that it is direct ssDNA generation by BLM, or alternatively the unloading of PICH, that supports activation of the abscission checkpoint.

BLM-depleted cells lack RPA bridges, indicating that BLM is essential for their formation. We propose that the distinct phenotypes observed in BLM- and PICH-depleted cells stem from the fact that PICH-depleted cells have been shown to still contain a small fraction of BLM bridges. These Aph-induced bridges seem to be different from double stranded centromeric bridges, which require PICH for the loading of BLM. Our findings demonstrate that PICH-depleted cells

accumulate RPA-coated UFBs (Appendix Figure S1C). This observation could explain the phenotypic differences between BLM- and PICH-depleted cells. We have now added this explanation in the manuscript text.

3) RO-3306 is a CDK1 inhibitor used to arrest cells in G2 so that the effects of BLM can be analysed in mitosis. But CDK1 phosphorylation of BLM is essential for its role in dissolution of catalytically interlinked DNA molecules (Pogliano, et al Science Advances 2022). Some discussion of this point should be included, and a Western blot showing BLM phosphorylation status during the "release" phase would indicate that the protein becomes "reactivated/rephosphorylated" after RO-3306 is removed (similar to Fig 1F of Pogliano et al). In other experiments, where Nocodazole is instead used to arrest the cells, the effects observed appear stronger than when RO-3306 is used - is there a direct side-by-side data for any result comparing the two methods?

We thank the reviewer for raising this point. In the revised manuscript we have discussed the Pogliano et al Science Advances, 2022 and also tested BLM phosphorylation in the RO3306 released cells.

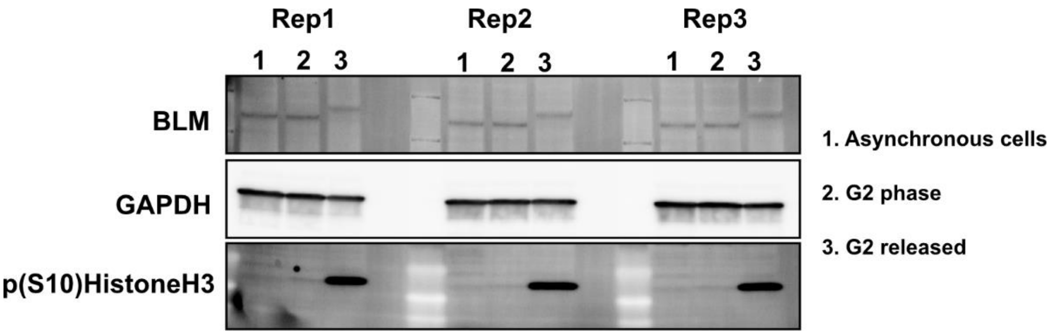

Nocodazole arrest cells at Prometaphase, where cells become more rounded have minimal surface adherence, this gives an advantage when harvesting only prometaphase cells using mitotic shake-off. In cases where we need all cells in mitosis for analysis, such as for western blotting etc, we therefore preferred to arrest cells using Nocodazole instead of RO-3306.

4) Several figures contain bar graphs with rather underwhelming changes (less than 2-fold with large error bars) are then used to justify conclusions. Some are listed as "representative of three independent experiments" but does this mean it is 1) the result of a single experiment that is similar to other experiments, or 2) the average of three independent experiments. It should be (2), but the language is ambiguous. Furthermore, given the conclusions being drawn, the authors should include the individual values of each experiment (for example similar to what is shown in Fig 3C) for all the bar graphs of the manuscript, so the reader can see the spread of data.

All the bar graph represents the average of three independent experiments. In the revised manuscript, we have clearly mentioned that point in the figure legends and also included the individual values from each replicate in the bar graph. The spread of data is represented in the source file.

Minor points to be addressed:

1) The methods section states that the statistical test used is indicated in each relevant figure legend, however none of the figure legends contain this information. Please address.

In the revised manuscript, we have included information about the statistical tests used in the figure legends.

2) Figure 4E shows ATR and ATRIP blots that are blank. How do we know these antibodies worked in this experiment? The absence of bands does not indicate the absence of ATR and ATRIP, just that they can't be detected and this could be because the antibodies don't work. A control extract that is positive for these antibodies should be included, which is on the same gel as the samples investigated.

We agree with the reviewer and in the revised version we have included a positive control for these antibodies in the supplementary figure EV6D.

3) If possible, the data of figure 5 should be shown as a 2 dimensional plot of the primary FACS data with area vs width measurements of the channel used to collect PI stained cells. This would help discriminate tetraploid G1 from diploid G2 cells (which overlap in a single peak when viewed only in one direction), particularly in the +IAA sample.

We thank reviewer for this comment. We have added the additional graphs in the source data. .

4) Several studies have shown that BLM itself is a substrate of Chk1 kinase activity (eg Petsalaki JCB 2014, Kharat Oncogene 2016). Please include some discussion on what this might mean for the proposed model.

We thank the reviewer for raising this point. In the revised version we have discussed this issue.

Prof. Ian D Hickson
University of Copenhagen
Center for Chromosome Stability, Department of Cellular and Molecular Medicine
Panum Institute
Blegdamsvej 3b
Copenhagen DK-2200
Denmark

16th Jan 2025

Re: EMBOJ-2024-116883R
MECHANISTIC INSIGHT INTO ANAPHASE BRIDGE SIGNALING TO THE ABSCISSION CHECKPOINT

Dear Ian,

Thank you for submitting your revised manuscript to The EMBO Journal, and my sincere apologies for the delay in getting it re-reviewed, due to limited availability of the original referees in the pre-Christmas period. I have now finally received the feedback of two of them, copied below for your information. While referee 3 is now satisfied with the revisions, I am afraid that referee 2 retains several substantive concerns that they feel have been insufficiently addressed during the revision.

As I mentioned, we normally allow only a single round of major revision, to avoid repeated rounds of (often incremental) improvements, and to give authors a decisive commitment from our side. Given that the issues raised by referee 2 continue to preclude publication of the study at this stage, but that there also have already been significant revision efforts as acknowledged by referee 3, I would in this case allow an exceptional second round of experimental revision to allow you to clarify the remaining open points. Should you be able to address them to the reviewer's satisfaction, we would be happy to eventually accept this work for EMBO Journal publication. As always, I would be open to considering possible options for revising based on a preliminary response letter, which I might in this case discuss with the critical referee to get their views on how promising they would seem.

I am therefore returning the manuscript to you for a second, final round of experimental revision, with the link below for eventual resubmission. When preparing a re-revised manuscript addressing the persistent issues, please also take care of the following points raised by our data editors with regard to the Figures and legends:

1. Please define the annotated p values ****/***/**/* as well as provide the exact p-values for the same in the legend of figure 1B, D, F, I, J; 2F, G; 3D, 5D, G, H; 6C, D; EV1 D, E; EV3 B, C, EV4 D, EV5 E as appropriate.
2. Please indicate what */ **/ ***/ **** represents; if this represents p value, please specify the exact p value in the legend of figure 3C.
3. Please indicate the statistical test used for data analysis in the legends of figure 3C.
4. Please note that the measure of center for the error bars needs to be defined in the legends of figures 5D, G, H; 6C, D
5. Please note that the scale bar needs to be defined for figure EV1 A
6. Please note that scale bar and its definition are missing for figure EV5 D.
7. Please note that the yellow arrows are not defined in the legend of figures 1G, H; 5C. This needs to be rectified.
8. Please note that the dotted white border is not defined in the legend of figure 5C. This needs to be rectified.

Once again, sorry for the delays with this re-review process, and I look forward to hearing back from you in due time.

With kind regards,

Hartmut

Hartmut Vodermaier, PhD
Senior Editor, The EMBO Journal
h.vodermaier@embojournal.org

*** PLEASE NOTE: All revised manuscript are subject to initial checks for completeness and adherence to our formatting guidelines. Revisions may be returned to the authors and delayed in their editorial re-evaluation if they fail to comply to the following requirements (see also our Guide to Authors for further information):

1) Every manuscript requires a Data Availability section (even if only stating that no deposited datasets are included). Primary datasets or computer code produced in the current study have to be deposited in appropriate public repositories prior to resubmission, and reviewer access details provided in case that public access is not yet allowed. Further information: embopress.org/page/journal/14602075/authorguide#dataavailability

2) Each figure legend must specify
- size of the scale bars that are mandatory for all micrograph panels
- the statistical test used to generate error bars and P-values
- the type error bars (e.g., S.E.M., S.D.)
- the number (n) and nature (biological or technical replicate) of independent experiments underlying each data point
- Figures may not include error bars for experiments with n<3; scatter plots showing individual data points should be used instead.

3) Revised manuscript text (including main tables, and figure legends for main and EV figures) has to be submitted as editable text file (e.g., .docx format). We encourage highlighting of changes (e.g., via text color) for the referees' reference.

4) Each main and each Expanded View (EV) figure should be uploaded as individual production-quality files (preferably in .eps, .tif, .jpg formats). For suggestions on figure preparation/layout, please refer to our Figure Preparation Guidelines: http://bit.ly/EMBOPressFigurePreparationGuideline

5) Point-by-point response letters should include the original referee comments in full together with your detailed responses to them (and to specific editor requests if applicable), and also be uploaded as editable (e.g., .docx) text files.

6) Please complete our Author Checklist, and make sure that information entered into the checklist is also reflected in the manuscript; the checklist will be available to readers as part of the Review Process File. A download link is found at the top of our Guide to Authors: embopress.org/page/journal/14602075/authorguide

7) All authors listed as (co-)corresponding need to deposit, in their respective author profiles in our submission system, a unique ORCiD identifier linked to their name. Please see our Guide to Authors for detailed instructions.

8) Please note that supplementary information at EMBO Press has been superseded by the 'Expanded View' for inclusion of additional figures, tables, movies or datasets; with up to five EV Figures being typeset and directly accessible in the HTML version of the article. For details and guidance, please refer to: embopress.org/page/journal/14602075/authorguide#expandedview

9) To facilitate reproducibilty and cross-laboratory adoption of methodologies, please structure the Materials & Methods section as outlined in our guide to authors, including a completed Reagents and Tools Table that can be downloaded from our author guidelines as well (https://www.embopress.org/page/journal/14602075/authorguide#structuredmethods).

10) Digital image enhancement is acceptable practice, as long as it accurately represents the original data and conforms to community standards. If a figure has been subjected to significant electronic manipulation, this must be clearly noted in the figure legend and/or the 'Materials and Methods' section. The editors reserve the right to request original versions of figures and the original images that were used to assemble the figure. Finally, we generally encourage uploading of numerical as well as gel/blot image source data; for details see: embopress.org/page/journal/14602075/authorguide#sourcedata

At EMBO Press, we ask authors to provide source data for the main manuscript figures. Our source data coordinator will contact you to discuss which figure panels we would need source data for and will also provide you with helpful tips on how to upload and organize the files.

Further information is available in our Guide For Authors:
https://www.embopress.org/page/journal/14602075/authorguide

Revision to The EMBO Journal should be submitted online within 90 days, unless an extension has been requested and approved by the editor; please click on the link below to submit the revision online before 16th Apr 2025:
Link Not Available

If you choose to alternatively have this study further considered by another EMBO Press publication, please use the following hyperlink to directly transfer the manuscript, optionally with inclusion of referee reports and identities:
Link Not Available

Referee #2:

Singh et al submit a revised manuscript that largely fails to address key concerns raised during revision. The specific issues that remain problematic are:

Abscission time in mCherry-tubulin cells. The authors provide new data to argue that the expression of the mCherry-tubulin construct is lower that the endogenous tubulin. This new data doesn't address the main concern as the fact remains that the localization of the mCherry-tubulin in this manuscript doesn't reflect the localization of endogenous tubulin, which never exhibits those disturbing aggregates. It is not acceptable to publish poor quality data like this. This aberrant localization could be due to a non-functional mCherry-tubulin construct that interferes with normal function. Alternatively, the expression levels of mCherry-tubulin may also be highly variable within these cells and the authors could be selecting high expressing cells for their live-cell analysis. In this context, the provided western blot simply reflects the average expression level in this cell line, a small proportion of cells expressing high levels of mCherry-tubulin would be entirely consistent with this western blot analysis.

Addressing abscission delays in cells with persistent UFBs. This critical issue remains unresolved. There is evidence in the literature that abscission can be regulated by the ATR and CHK1 pathway independently of missegregated DNA (PMID: 25904336). It is therefore critical to address this issue to support the main model proposed in this manuscript. The authors are correct and Lap2b won't differentiate between UFBs and chromatin bridges, but this marker will label UFBs that are selectively induced under the experimental conditions used in this paper. Alternatively, the authors could follow RPA bridges by live-cell imaging as fluorescent markers for the RPA complex are available.

Selective role of BLM in abscission regulation. The authors argue that addressing the role of BLM in the response to nucleoporin depletion requires a substantial amount of experimentation, this is simply not the case. As shown by multiple papers in this field, this assay requires a simple siRNA transfection against Nups followed by staining of fixed cells with anti-tubulin antibodies. Given the gaps in this manuscript regarding the specificity of the BLM phenotypes (see above), the manuscript would be way more convincing with the suggested analysis.

Connection between BLM/RPA with known components of the abscission checkpoint. This important issue remains unresolved. It is totally unclear to this reviewer why the co-localization of RPA/BLM with active AuroraB (or other components of the abscission checkpoint) cannot be addressed experimentally. If Aurora b pThr-232 localizes to the midbody arms, its co-localization with the RPA coating the persistent UFB should be fairly straightforward, a simple staining of tubulin will mark the midbody arms as an area that is clearly distinct from the Flemming body and the cytoplasm of the connected cells.

Referee #3:

The authors have sufficiently addressed the concerns of my original review. A very nice work.

Dear Hartmut,

We would like to thank you for giving us the opportunity to submit a second round of revisions and also wish to thank the referees for their very helpful comments on the manuscript. We have addressed all the comments and hope that the new analyses will make the manuscript more compelling.

In the response below, the original comments of the referees are given in **bold** text, and our responses are given in blue text.

Best wishes,

Ian

Referee #2:

Singh et al submit a revised manuscript that largely fails to address key concerns raised during revision. The specific issues that remain problematic are:

1. Abscission time in mCherry-tubulin cells. The authors provide new data to argue that the expression of the mCherry-tubulin construct is lower that the endogenous tubulin. This new data doesn't address the main concern as the fact remains that the localization of the mCherry-tubulin in this manuscript doesn't reflect the localization of endogenous tubulin, which never exhibits those disturbing aggregates. It is not acceptable to publish poor quality data like this. This aberrant localization could be due to a non-functional mCherry-tubulin construct that interferes with normal function. Alternatively, the expression levels of mCherry-tubulin may also be highly variable within these cells and the authors could be selecting high expressing cells for their live-cell analysis. In this context, the provided western blot simply reflects the average expression level in this cell line, a small proportion of cells expressing high levels of mCherry-tubulin would be entirely consistent with this western blot analysis.

We thank the reviewer for the constructive feedback. To address the concern regarding the nature of the aggregates observed in the mCherry-tubulin images, we performed immunofluorescence staining using an anti-tubulin antibody in U2OS-H2BGFP-mCherry-tubulin cells. The results show clearly that the aggregates do not co-localize with tubulin, indicating they are mCherry fluorescence artifacts rather than mCherry-tubulin aggregates.

Additionally, the U2OS-H2BGFP-mCherry-tubulin cells used in this study are a clonal population, exhibiting consistent expression levels of the mCherry-tubulin construct across the population. We have updated the supplementary data to include the new immunofluorescence images for clarity (Appendix Figure S1A). We have added the figure below too.

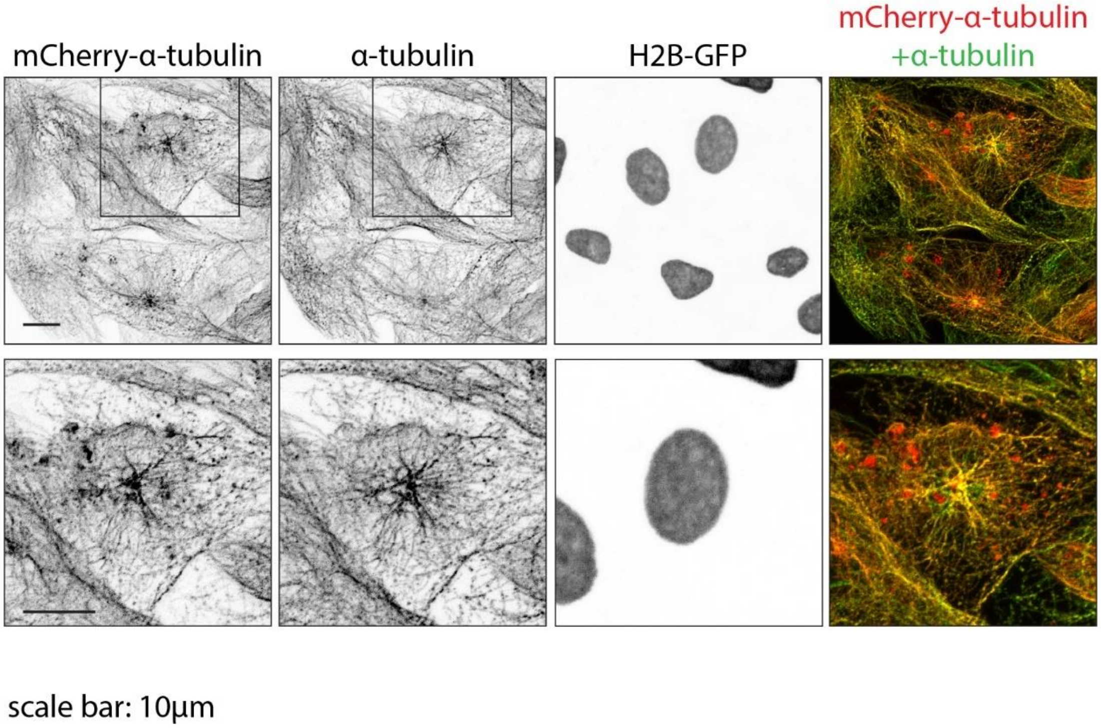

Figure: The aggregates seen in U2OS cells are mCherry aggregates. The square box in top panel is zoomed as lower panel. The big aggregates seen in mCherry-α-tubulin are not seen in anti-tubulin channel.

2. Addressing abscission delays in cells with persistent UFBs. This critical issue remains unresolved. There is evidence in the literature that abscission can be regulated by the ATR and CHK1 pathway independently of missegregated DNA (PMID: 25904336). It is therefore critical to address this issue to support the main model proposed in this manuscript. The authors are correct and Lap2b won't differentiate between UFBs and chromatin bridges, but this marker will label UFBs that are selectively induced under the experimental conditions used in this paper. Alternatively, the authors could follow RPA bridges by live-cell imaging as fluorescent markers for the RPA complex are available.

Similar to the above cited manuscript, we have shown in the manuscript that replication stress induced DNA lesions can activate a cell cycle delay. However, it is clear from our data (Figure EV3C) that this effect is much less pronounced than in cases where a visible UFB is present in the cell (Figure EV3C). Thus, we would like to emphasize that replication stress in general keeps the abscission checkpoint activated causing a delay in abscission, but in cells where there is still an unresolved ultra-fine bridge, there is an additional abscission delay.

3. Selective role of BLM in abscission regulation. The authors argue that addressing the role of BLM in the response to nucleoporin depletion requires a substantial amount of experimentation,

this is simply not the case. As shown by multiple papers in this field, this assay requires a simple siRNA transfection against Nups followed by staining of fixed cells with anti-tubulin antibodies. Given the gaps in this manuscript regarding the specificity of the BLM phenotypes (see above), the manuscript would be way more convincing with the suggested analysis.

We thank the reviewer for this insightful comment and fully agree that the assay to assess the impact of nucleoporin depletion on cytokinesis is both feasible and informative. In response, we performed the suggested experiment by depleting NUP153 via siRNA and subsequently analyzed cells in cytokinesis using anti-tubulin staining. Our quantification shows that NUP153 depletion, as expected, led to an increase in the percentage of cells undergoing cytokinesis. However, this effect was seen whether or not the cells expressed BLM. These results indicate that BLM does not play any obvious role in nucleoporin-triggered cytokinesis regulation. We have now included these new data in the revised manuscript (Appendix Figure S1B) and updated the Results and Discussion sections accordingly.

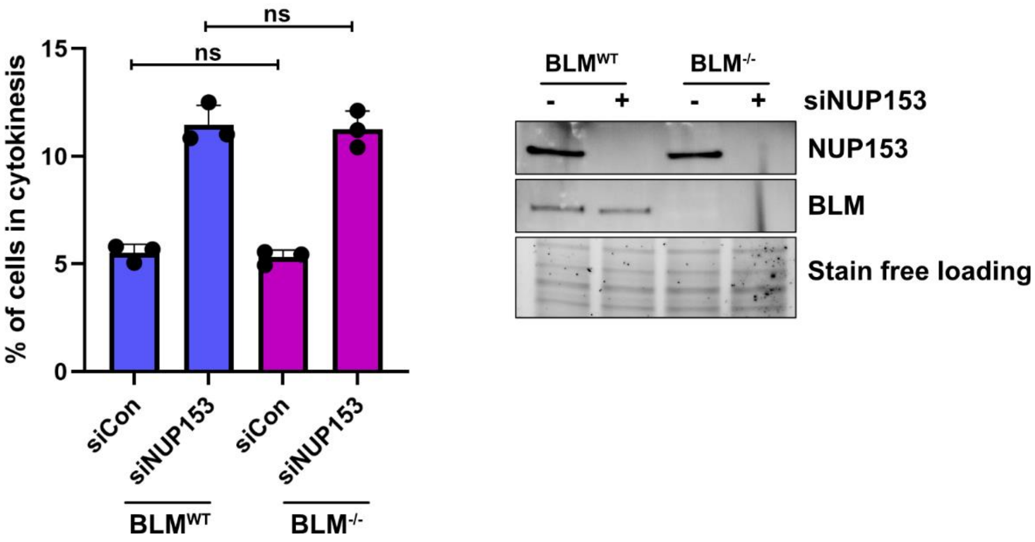

4. Connection between BLM/RPA with known components of the abscission checkpoint. This important issue remains unresolved. It is totally unclear to this reviewer why the co-localization of RPA/BLM with active AuroraB (or other components of the abscission checkpoint) cannot be addressed experimentally. If Aurora b pThr-232 localizes to the midbody arms, its co-localization with the RPA coating the persistent UFB should be fairly straightforward, a simple staining of tubulin will mark the midbody arms as an area that is clearly distinct from the Flemming body and the cytoplasm of the connected cells.

We thank the reviewer for this valuable suggestion. In our earlier attempts, we encountered technical difficulties in obtaining high-quality images using triple immunofluorescence due to signal bleed-through between the channels, which compromised co-localization analysis. However, we have now optimized our imaging conditions and successfully acquired the required images using bright field (BF) microscopy to clearly delineate the midbody structure. We provide representative images below,

showing co-localization of RPA with active Aurora B (pThr232) at the midbody. These images support the spatial proximity of RPA with abscission checkpoint components. These new data have been included in the revised manuscript (Figure EV5C).

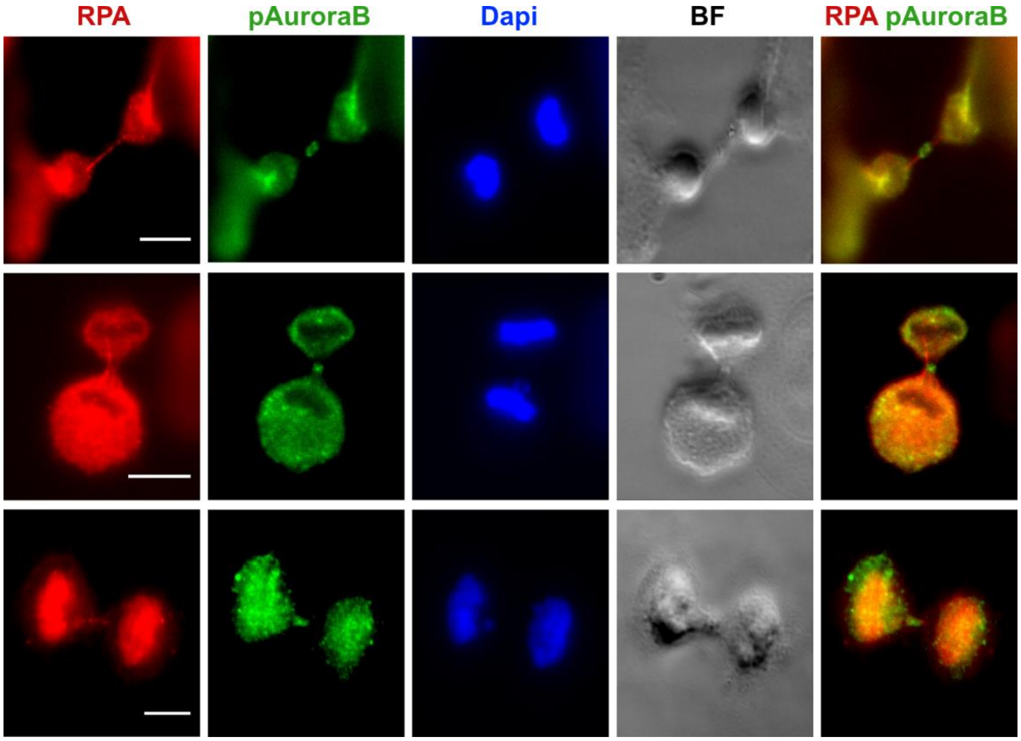

Figure: Three examples to show co-localization of persistent RPA bridges passing through the midbody and pAurora B Thr-232 at midbody upon replication stress in BLM^{WT} cells.

Prof. Ian D Hickson
University of Copenhagen
Center for Chromosome Stability, Department of Cellular and Molecular Medicine
Panum Institute
Blegdamsvej 3b
Copenhagen DK-2200
Denmark

24th Apr 2025

Re: EMBOJ-2024-116883R1
MECHANISTIC INSIGHT INTO ANAPHASE BRIDGE SIGNALING TO THE ABSCISSION CHECKPOINT

Dear Ian,

Thank you for submitting your re-revised manuscript for our consideration. I have now carefully gone through your response and added data, and found them to satisfactorily address then remaining key issues that had been raised in the previous round. In this light, I am pleased to now accept the study for publication in The EMBO Journal.

Your manuscript will be processed for publication by EMBO Press. It will be copy edited and you will receive page proofs prior to publication. Please note that you will be contacted by Springer Nature Author Services to complete licensing and payment information.

You may qualify for financial assistance for your publication charges - either via a Springer Nature fully open access agreement or an EMBO initiative. Check your eligibility: https://www.embopress.org/page/journal/14602075/authorguide#chargesguide

Should you be planning a Press Release on your article, please get in contact with embo_production@springernature.com as early as possible in order to coordinate publication and release dates.

Thank you again for this contribution to The EMBO Journal and congratulations on a successful publication! Please consider us again in the future for your most exciting work.

With kind regards,

Hartmut

Hartmut Vodermaier, PhD
Senior Editor, The EMBO Journal
h.vodermaier@embojournal.org

EMBO Press Author Checklist

Corresponding Author Name: Ian D. Hickson	
Journal Submitted to: The EMBO Journal	
Manuscript Number: EMBOJ-2024-116883	

USEFUL LINKS FOR COMPLETING THIS FORM
The EMBO Journal - Author Guidelines
EMBO Reports - Author Guidelines
Molecular Systems Biology - Author Guidelines
EMBO Molecular Medicine - Author Guidelines

Reporting Checklist for Life Science Articles (updated January
This checklist is adapted from Materials Design Analysis Reporting (MDAR) Checklist for Authors. MDAR establishes a minimum set of requirements in transparent reporting in the life sciences (see Statement of Task: 10.31222/osf.io/9sm4x). Please follow the journal's guidelines in preparing your
Please note that a copy of this checklist will be published alongside your article.

Abridged guidelines for figures
1. Data
The data shown in figures should satisfy the following conditions:
- the data were obtained and processed according to the field's best practice and are presented to reflect the results of the experiments in an accurate and unbiased manner.
- ideally, figure panels should include only measurements that are directly comparable to each other and obtained with the same assay.
- plots include clearly labeled error bars for independent experiments and sample sizes. Unless justified, error bars should not be shown for technical
- if n<5, the individual data points from each experiment should be plotted. Any statistical test employed should be justified.
- Source Data should be included to report the data underlying figures according to the guidelines set out in the authorship guidelines on Data

2. Captions
Each figure caption should contain the following information, for each panel where they are relevant:
- a specification of the experimental system investigated (eg cell line, species name).
- the assay(s) and method(s) used to carry out the reported observations and measurements.
- an explicit mention of the biological and chemical entity(ies) that are being measured.
- an explicit mention of the biological and chemical entity(ies) that are altered/varied/perturbed in a controlled manner.
- the exact sample size (n) for each experimental group/condition, given as a number, not a range;
- a description of the sample collection allowing the reader to understand whether the samples represent technical or biological replicates (including how many animals, litters, cultures, etc.).
- a statement of how many times the experiment shown was independently replicated in the laboratory.
- definitions of statistical methods and measures:
  - common tests, such as t-test (please specify whether paired vs. unpaired), simple χ2 tests, Wilcoxon and Mann-Whitney tests, can be unambiguously identified by name only, but more complex techniques should be described in the methods section;
  - are tests one-sided or two-sided?
  - are there adjustments for multiple comparisons?
  - exact statistical test results, e.g., P values = x but not P values < x;
  - definition of 'center values' as median or average;
  - definition of error bars as s.d. or s.e.m.

Please complete ALL of the questions below.
Select "Not Applicable" only when the requested information is not relevant for your study.

Materials

Newly Created Materials	Information included in the manuscript?	In which section is the information available? (Reagents and Tools Table, Materials and Methods, Figures, Data Availability Section)
New materials and reagents need to be available; do any restrictions apply?	Yes	Materials and Methods

Antibodies	Information included in the manuscript?	In which section is the information available? (Reagents and Tools Table, Materials and Methods, Figures, Data Availability Section)
For **antibodies** provide the following information:<br>- Commercial antibodies: RRID (if possible) or supplier name, catalogue number and or/clone number<br>- Non-commercial: RRID or citation	Yes	Appendix Table S2

DNA and RNA sequences	Information included in the manuscript?	In which section is the information available? (Reagents and Tools Table, Materials and Methods, Figures, Data Availability Section)
**Short novel DNA or RNA including primers, probes**: provide the sequences.	Yes	Materials and Methods and Appendix Table S1

Cell materials	Information included in the manuscript?	In which section is the information available? (Reagents and Tools Table, Materials and Methods, Figures, Data Availability Section)
**Cell lines:** Provide species information, strain. Provide accession number in repository **OR** supplier name, catalog number, clone number, and/**OR** RRID.	Yes	Materials and Methods
**Primary cultures:** Provide species, strain, sex of origin, genetic modification status.	Not Applicable	
Report if the cell lines were recently **authenticated** (e.g., by STR profiling) and tested for mycoplasma contamination.	Yes	Materials and Methods

Experimental animals	Information included in the manuscript?	In which section is the information available? (Reagents and Tools Table, Materials and Methods, Figures, Data Availability Section)
**Laboratory animals or Model organisms:** Provide species, strain, sex, age, genetic modification status. Provide accession number in repository **OR** supplier name, catalog number, clone number, **OR** RRID.	Not Applicable	
**Animal observed in or captured from the field:** Provide species, sex, and age where possible.	Not Applicable	
Please detail **housing and husbandry conditions**.	Not Applicable	

Plants and microbes	Information included in the manuscript?	In which section is the information available? (Reagents and Tools Table, Materials and Methods, Figures, Data Availability Section)
**Plants:** provide species and strain, ecotype and cultivar where relevant, unique accession number if available, and source (including location for collected wild specimens).	Not Applicable	
**Microbes:** provide species and strain, unique accession number if available, and source.	Not Applicable	

Human research participants	Information included in the manuscript?	In which section is the information available? (Reagents and Tools Table, Materials and Methods, Figures, Data Availability Section)
If collected and within the bounds of privacy constraints report on age, sex and gender or ethnicity for all study participants.	Not Applicable	

Core facilities	Information included in the manuscript?	In which section is the information available? (Reagents and Tools Table, Materials and Methods, Figures, Data Availability Section)
If your work benefited from core facilities, was their service mentioned in the acknowledgments section?	Not Applicable	

Design

Study protocol	Information included in the manuscript?	In which section is the information available? (Reagents and Tools Table, Materials and Methods, Figures, Data Availability Section)
If study protocol has been **pre-registered, provide DOI in the manuscript**. For clinical trials, provide the trial registration number **OR** cite DOI.	Not Applicable	
Report the **clinical trial registration number** (at ClinicalTrials.gov or equivalent), where applicable.	Not Applicable	

Laboratory protocol	Information included in the manuscript?	In which section is the information available? (Reagents and Tools Table, Materials and Methods, Figures, Data Availability Section)
Provide DOI OR other citation details if **external detailed step-by-step protocols** are available.	Not Applicable	

Experimental study design and statistics	Information included in the manuscript?	In which section is the information available? (Reagents and Tools Table, Materials and Methods, Figures, Data Availability Section)
Include a statement about **sample size** estimate even if no statistical methods were used.	Not Applicable	
Were any steps taken to minimize the effects of subjective bias when allocating animals/samples to treatment (e.g. **randomization procedure**)? If yes, have they been described?	Not Applicable	
Include a statement about **blinding** even if no blinding was done.	Not Applicable	
Describe **inclusion/exclusion criteria** if samples or animals were excluded from the analysis. Were the criteria pre-established?<br><br>If sample or data points were omitted from analysis, report if this was due to attrition or intentional exclusion and provide justification.	Not Applicable	
For every figure, are **statistical tests** justified as appropriate? Do the data meet the assumptions of the tests (e.g., normal distribution)? Describe any methods used to assess it. Is there an estimate of variation within each group of data? Is the variance similar between the groups that are being statistically compared?	Yes	Materials and Methods and Figures

Sample definition and in-laboratory replication	Information included in the manuscript?	In which section is the information available? (Reagents and Tools Table, Materials and Methods, Figures, Data Availability Section)
In the figure legends: state number of times the experiment was **replicated** in laboratory.	Yes	Figures
In the figure legends: define whether data describe **technical or biological replicates**.	Yes	Figures

Ethics

Ethics	Information included in the manuscript?	In which section is the information available? (Reagents and Tools Table, Materials and Methods, Figures, Data Availability Section)
Studies involving **human participants**: State details of **authority granting ethics approval** (IRB or equivalent committee(s), provide reference number for approval.	Not Applicable	
Studies involving **human participants**: Include a statement confirming that **informed consent** was obtained from all subjects and that the experiments conformed to the principles set out in the WMA Declaration of Helsinki and the Department of Health and Human Services Belmont Report.	Not Applicable	
Studies involving **human participants:** For publication of **patient photos**, include a statement confirming that consent to publish was obtained.	Not Applicable	
Studies involving experimental **animals**: State details of **authority granting ethics approval** (IRB or equivalent committee(s), provide reference number for approval. Include a statement of compliance with ethical regulations.	Not Applicable	
Studies involving **specimen and field samples:** State if relevant **permits** obtained, provide details of authority approving study; if none were required, explain why.	Not Applicable	

Dual Use Research of Concern (DURC)	Information included in the manuscript?	In which section is the information available? (Reagents and Tools Table, Materials and Methods, Figures, Data Availability Section)
Could your study fall under dual use research restrictions? Please check biosecurity documents and list of **select agents and toxins** (CDC): https://www.selectagents.gov/sat/list.htm	Not Applicable	
If you used a select agent, is the security level of the lab appropriate and reported in the manuscript?	Not Applicable	
If a study is subject to dual use research of concern regulations, is the name of the **authority granting approval and reference number** for the regulatory approval provided in the manuscript?	Not Applicable	

Reporting

The MDAR framework recommends adoption of discipline-specific guidelines, established and endorsed through community initiatives. Journals have their own policy about requiring specific guidelines and recommendations to complement MDAR.

Adherence to community standards	Information included in the manuscript?	In which section is the information available? (Reagents and Tools Table, Materials and Methods, Figures, Data Availability Section)
State if relevant guidelines or checklists (e.g., **ICMJE, MIBBI, ARRIVE, PRISMA**) have been followed or provided.	Not Applicable	
For **tumor marker prognostic studies,** we recommend that you follow the **REMARK** reporting guidelines (see link list at top right). See author guidelines, under 'Reporting Guidelines'. Please confirm you have followed these guidelines.	Not Applicable	
For **phase II and III randomized controlled trials**, please refer to the **CONSORT** flow diagram (see link list at top right) and submit the CONSORT checklist (see link list at top right) with your submission. See author guidelines, under 'Reporting Guidelines'. Please confirm you have submitted this list.	Not Applicable	

Data Availability

Data availability	Information included in the manuscript?	In which section is the information available? (Reagents and Tools Table, Materials and Methods, Figures, Data Availability Section)
Have **primary datasets** been deposited according to the journal's guidelines (see 'Data Deposition' section) and the respective accession numbers provided in the Data Availability Section?	Not Applicable	
Were **human clinical and genomic datasets** deposited in a public access-controlled repository in accordance to ethical obligations to the patients and to the applicable consent agreement?	Not Applicable	
Are **computational models** that are central and integral to a study available without restrictions in a machine-readable form? Were the relevant accession numbers or links provided?	Not Applicable	
If publicly available data were reused, provide the respective **data citations in the reference list**.	Not Applicable	
